# Prioritization of novel anti-infective stilbene derivatives by combining metabolomic data organization and a stringent 3R-infection model in a knowledge graph†

**DOI:** 10.1039/d4ra08421g

**Published:** 2025-04-23

**Authors:** Olivier Auguste Kirchhoffer, Luis Quirós-Guerrero, Jahn Nitschke, Louis-Félix Nothias, Frédéric Burdet, Laurence Marcourt, Nabil Hanna, Florence Mehl, Bruno David, Antonio Grondin, Emerson Ferreira Queiroz, Marco Pagni, Thierry Soldati, Jean-Luc Wolfender

**Affiliations:** a Institute of Pharmaceutical Sciences of Western Switzerland, University of Geneva, CMU 1211 Geneva Switzerland Jean-Luc.Wolfender@unige.ch; b School of Pharmaceutical Sciences, University of Geneva, CMU 1211 Geneva Switzerland; c Department of Biochemistry, Faculty of Sciences, University of Geneva Quai Ernest-Ansermet 30 1205 Geneva Switzerland; d Université Côte d’Azur, CNRS, ICN France; e Vital-IT, SIB Swiss Institute of Bioinformatics 1015 Lausanne Switzerland; f Green Mission Department, Herbal Products Laboratory, Pierre Fabre Research Institute Toulouse France

## Abstract

The rising threat of multidrug-resistant tuberculosis, caused by *Mycobacterium tuberculosis*, underscores the urgent need for new therapeutic solutions to tackle the challenge of antibiotic resistance. The current study utilized an innovative 3R infection model featuring the amoeba *Dictyostelium discoideum* infected with *Mycobacterium marinum*, serving as stand-ins for macrophages and *M. tuberculosis*, respectively. This high-throughput phenotypic assay allowed for the evaluation of more specific anti-infective activities that may be less prone to resistance mechanisms. To discover novel anti-infective compounds, a diverse collection of 1600 plant NEs from the Pierre Fabre Library was screened using the latter assay. Concurrently, these NEs underwent untargeted UHPLC-HRMS/MS analysis. The biological screening flagged the NE from *Stauntonia brunoniana* as one of the anti-infective hit NEs. High-resolution HPLC micro-fractionation coupled with bioactivity profiling was employed to highlight the natural products driving this bioactivity. Stilbenes were eventually identified as the primary active compounds in the bioactive fractions. A knowledge graph was then used to leverage the heterogeneous data integrated into it to make a rational selection of stilbene-rich NEs. Using both CANOPUS chemical classes and Jaccard similarity indices to compare features within the metabolome of the 1600 plant NEs collection, 14 NEs rich in stilbenes were retrieved. Among those, the roots of *Gnetum edule* were flagged as possessing broader chemo-diversity in their stilbene content, along with the corresponding NE also being a strict anti-infective. Eventually, a total of 11 stilbene oligomers were isolated from *G. edule* and fully characterized by NMR with their absolute stereochemistry established through electronic circular dichroism. Six of these compounds are new since they possess a stereochemistry which was never described in the literature to the best of our knowledge. All of them were assessed for their anti-infective activity and (−)-gnetuhainin M was reported as having the highest anti-infective activity with an IC_50_ of 22.22 μM.

## Abstract 2

Knowledge graph organization of metabolomic data provides a powerful tool to accelerate the discovery of new therapeutic solutions against multidrug-resistant tuberculosis.

## Graphical abstract

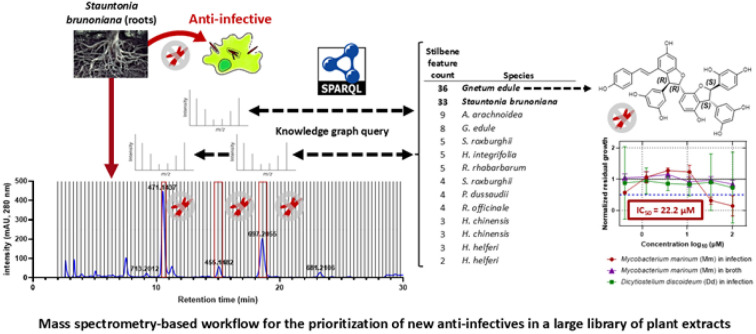

## Introduction

Multidrug-resistant tuberculosis (MDR-TB) and other resistant strains of Mycobacteria (Q194309 (https://www.wikidata.org/wiki/Q194309)) with the ability to resist conventional antibiotic treatments are a growing threat to human health. As of 2022, no less than 1.3 million people died from *Mycobacterium tuberculosis* (Mtb, Q130971 (https://www.wikidata.org/wiki/Q130971)), while an estimated 410 000 people developed multidrug-resistant or rifampicin-resistant TB (MDR/RR-TB)^1^ underlining the need for new and more specific therapeutic solutions in this disease area. To address these problems, natural extracts (NEs) from plant origin remain a largely untapped reservoir for bioactive natural products (NPs) discovery. To date less than 10% of plant species have been evaluated for medicinal properties.^1,2^

In this context, the chemical diversity of a registered plant NEs collection (containing 1600 samples) was leveraged. This set covers about 30% of all known botanical families and was systematically analysed by mass spectrometry-based metabolite profiling,^3^ thereby generating consequential amounts of spectral data from HRMS/MS analyses.

Spectral organization through molecular networking (MN) along with the GNPS platform have established themselves as tools to organize, visualize and compare HRMS/MS data since their inception in 2014.^4,5^ Yet it has been shown that their use can become challenging with the increasing size of datasets. Furthermore, large datasets tend to be more prone to chromatographic misalignments due to experimental factors. The iterative nature of the data acquisition processes and the fact that samples are likely analysed over large periods of time increases the risks for potential batch-effects.^6^ The 1600 NEs collection used in this study was no exception in that matter as was described by Allard *et al.* (2023).^3^ Although aligning UHPLC-HRMS/MS data does not generally pose any major problem when working with MNs in a *dataset-centric* manner, problems arise when it comes to incrementing data in an existing dataset as it would require re-processing the entire dataset. This problem changed the paradigm from *dataset-centric* approaches to *sample-centric* approaches relying on unaligned datasets organized in knowledge graph (KG) frameworks.^7^

The present study will establish a method to navigate large unaligned spectral spaces based on plant extracts content. As a proof-of-concept, chemically informed queries will be established to identify extracts likely to contain bioactive NPs. In the frame of the TB disease area, increasing specificity of therapeutic solutions can be addressed with more stringent phenotypic assays. As opposed to screening drug candidates on mycobacteria grown *in broth*, screening approaches that utilize infection models have been elaborated to better capture the specificities of Mtb's intracellular behavior.^8,9^ In this study, we employed an innovative 3R infection model using *Mycobacterium marinum* (Mm, ATCC BAA-535 (https://www.atcc.org/products/baa-535)) alongside the amoeba and professional phagocyte *Dictyostelium discoideum* (Dd, ATCC MYA-4120 (https://www.atcc.org/products/mya-4120)) as stand-ins for Mtb and macrophages, respectively. We used this model in a high-throughput phenotypic assay which enabled simultaneous assessment of a sample's impact on both the host and the pathogen, making it a particularly stringent assay.^10^

Combined with the above-mentioned assay, the biological screening of the 1600 NEs library provided an unbiased method for targeting NEs of interest. The current study focuses on one of the hit NEs identified during the bioactivity screening of the above-mentioned 1600 NEs collection. Based on the chemistry of this extract, the available metabolomic data of the KG was then explored to prioritize other potentially bioactive extracts.

In the first step, a selected hit NE was subjected to HPLC bioactivity profiling to identify the LC-peaks containing the bioactivity. MS-based annotation of the corresponding metabolites resulted in the identification of a common scaffold of biological interest. Then, the fragmentation spectra of those metabolites were used as proxies to search for analogues within all species contained in the KG. This strategy aimed at finding NEs containing a maximum number of diverse metabolites derived from a scaffold of interest and guide their isolation.

## Results and discussion

### From biological screening to bioactive NE

The current work was initiated by the biological screening of the 1600 NEs collection used for UHPLC-HRMS/MS metabolite profiling described by Allard *et al.* (2023).^3^ The biological screening was based on an infection model in which the amoeba host Dd is infected by Mm.^11,12^ Two readouts resulted from this screening: samples that reduce the growth of Mm by at least 50% during infection were classified as “anti-infectives”, while those that reduce the growth of Dd by at least 50% were classified as “Dd inhibitors”. A second assay, in which Mm was grown in broth instead of inside Dd host cells was carried out separately with all samples. This second assay aimed at classifying samples that were “anti-biotic”. This approach allowed us to differentiate anti-biotic NEs from anti-infective NEs, the latter acting only on the growth patterns during infection. Therefore, NEs were considered hits if they were both anti-infectives and not anti-biotic. Ideally, they should also not affect the growth of the host. NEs classified as “anti-infective Dd inhibitors” were also considered as hits on the assumption that components responsible for the anti-infective effect in the NE were not necessarily the same as those responsible for inhibiting the growth of the amoeba.

The screening of the 1600 NEs collection eventually yielded 12 anti-infective hits (hit rate: 0.75%), 8 of which were strict anti-infectives and 4 others were anti-infective Dd inhibitors (not covered in this study, described in the work of J. Nitschke (2024)^13^). Among those, the roots ethyl acetate extract of a Lardizabalaceae *Stauntonia brunoniana* (Decne.) Wall. Ex Hemsl. (Q10865526 (https://www.wikidata.org/wiki/Q10865526)) stood out as a strict anti-infective hit with good activity. To our knowledge, no phytochemical investigations were reported for this species at the time of writing (see WikiData query (https://w.wiki/9rLt)), it thus constituted the focus of the current study.

**Fig. 1:**
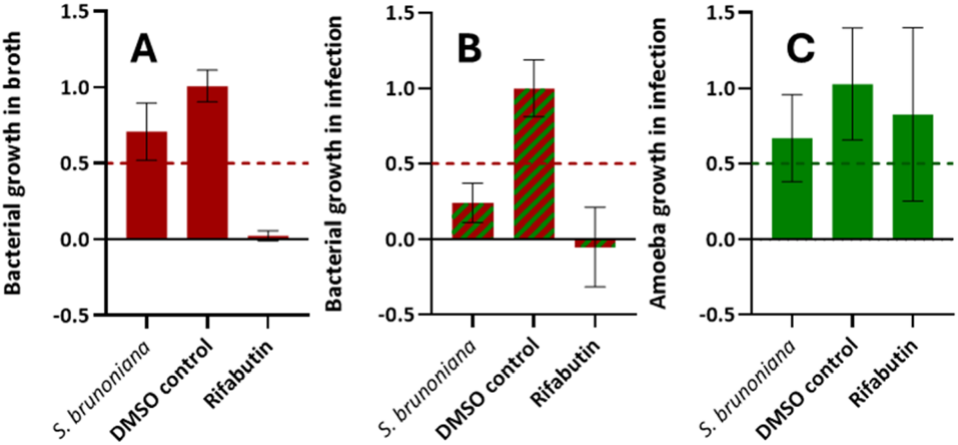
Normalized biological activities of the ethyl acetate extract of *Stauntonia brunoniana* (3 biological replicates, 1 technical replicate). (A) *M. marinum* growth in broth is inhibited by 29%. (B) *M. marinum* growth in infection is inhibited by 76%. (C) *D. discoideum* growth in infection is inhibited by 33%. Rifabutin was used as a positive control and DMSO as a negative control (value normalized to 1.0). 50% growth inhibition is the threshold (represented by a dotted line) set for a NE to be considered as bioactive in each respective category.

This NE was classified as a “strict anti-infective” as it displayed inhibition of intracellular bacterial growth (during infection) by 76% (Fig. 1B) and was only weakly active on the bacteria grown *in vitro* (29% inhibition of growth in Fig. 1A). The biological activity on the host was also assessed and did not show significant toxicity (33% inhibition of growth in Fig. 1C). Hence, the ethyl acetate extract of the roots of *S. brunoniana* was chosen for subsequent HPLC bioactivity profiling in order to highlight the LC-peaks responsible for this activity.

### Metabolite profiling of the active NE

**Fig. 2:**
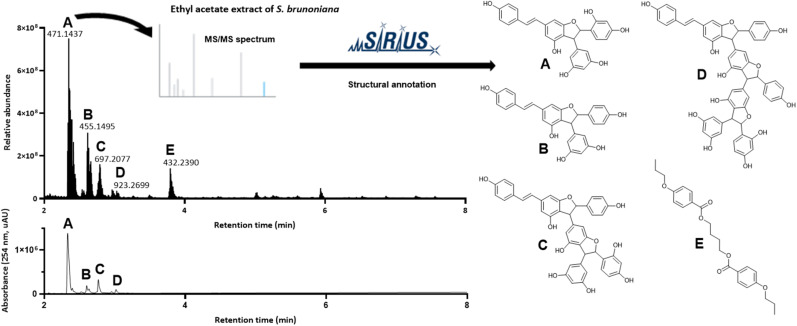
UHPLC-PDA-HRMS metabolite profile of the ethyl acetate extract of *S. brunoniana* roots acquired within the collection of 1600 NEs. Positive ion mode UHPLC-HRMS trace (base peak chromatogram) and UV trace at 254 nm are displayed in the range of 2–8 min (10 min run in total). Annotated compounds A–E were dereplicated using SIRIUS (with structures obtained *via* the CSI:FingerID module) based on the MS/MS spectra recorded on the associated most intense ions for which *m*/*z* are displayed.

To obtain an overview of the chemical composition of the active NE, we evaluated its corresponding UHPLC-PDA-HRMS/MS data in positive mode acquired on the entire collection of 1600 NEs to dereplicate some of the most intense LC-MS-peaks.^3^ For this, we made use of the dereplication results obtained on the 1600 NEs collection^3^ with GNPS, TimaR^14^ and SIRIUS^15^ through the CSI:FingerID^16^ and CANOPUS modules.^17,18^ This allowed for the determination of high-confidence molecular formulae (MF)^19^ and the annotation of the chemical classes of the detected features.^17,18^ All these tools allowed for the annotation of components observed in selected NE (Fig. 2).

This process highlighted the most intense LC-MS-peaks in the MS trace as being stilbene derivatives. This was also corroborated by online-UV-PDA data for those peaks showing characteristic bands reported for stilbenes at 310 nm and 327 nm.^20^ The analyses revealed compound A with positive ion (PI) [M + H]^+^*m*/*z* of 471.1437 for a MF C_28_H_22_O_7_ (*Δ* = −0.21 ppm), compound B with a PI [M + H]^+^*m*/*z* of 455.1495 (MF C_28_H_22_O_6_, *Δ* = 1.32 ppm), compound C with a PI [M + H]^+^*m*/*z* 697.2077 (MF C_42_H_32_O_10_, *Δ* = 1.29 ppm) and compound D with PI [M + H]^+^*m*/*z* of 923.2699 (MF C_56_H_42_O_13_, *Δ* = 0.11 ppm). Dereplication of these ions based on their MS/MS spectra indicated that compounds A and B corresponded to stilbene dimers, while compound C corresponded to a trimer and compound D to a tetramer. The putative annotated structures obtained are displayed in Fig. 2 and corresponded to the best candidate structures proposed by the CSI:FingerID module of SIRIUS.^15,16^ Compound E on the other hand seemed to be a structural outlier which, unlike stilbenes, was not detectable in the UV trace.

To assess the bioactivity of these compounds putatively annotated as stilbenes in the historical collection of 1600 NEs, a new NE was prepared by Accelerated Solvent Extraction (ASE). The procedure used consisted in three successive extraction methods with solvents of increasing polarity (hexane, ethyl acetate and methanol respectively). It should be noted that historical ethyl acetate extracts (as reported in Allard *et al.*^3^) all underwent a pre-treatment aimed at reducing their content down to medium-polarity compounds, by removing very non-polar components through a DCM rinsing step on silica pads.^3^ For better scalability, this process was replaced by a hexane-extraction step before the ethyl acetate extraction in the current study to provide a similar removal of non-polar compounds.

The metabolite profile of the new NE matched well with the historical one (ESI Fig. 1†) and confirmed that compound E (absent in the new NE) was a contaminant. Structure E was reported as a component of meltable ink used in printing^21^ that was a contaminant common to the 20 adjacent samples in the series of 1600 NEs analysed.

### HPLC-PDA based bioactivity profiling of the new NE

To identify if the annotated stilbenes were responsible for the biological activities observed at the NE level, an HPLC-based bioactivity profiling^22^ was carried out. The HPLC-PDA chromatographic conditions were optimized to maximize the separation of all stilbenes (ESI Fig. 1B and C†). These conditions were transferred^23^ to semi-preparative HPLC-PDA on a 10 mm i.d. column that allowed micro-fractions to be collected in a single 96-deepwell plate for further biological testing.

**Fig. 3:**
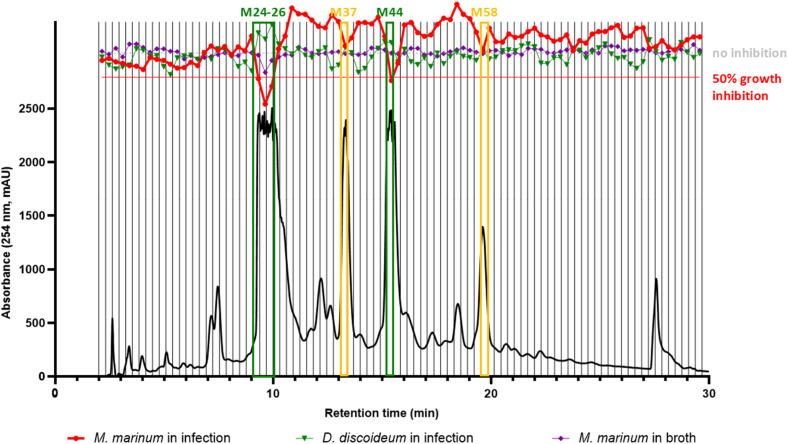
Semi-preparative HPLC-PDA microfractionation of the ethyl acetate extract of *S. brunoniana* roots. Vertical lines correspond to the 92 micro-fractions that were tested for their anti-infective activities in infection (in red), their effect on the host in infection (in green) as well as anti-biotic activities in broth (in purple) with 3 biological replicates, 1 technical replicate for each experiment. Two zones with inhibitory activities above 50% can be observed (in green M24-26 and M44) with M37 and M58 highlighted in yellow also showing some reduction of growth relative to neighbouring fractions.

The dried micro-fractions were subjected to biological testing with the same strategy as for screening the 1600 NEs collection. As previously, growth inhibition data from Mm grown in broth or infecting Dd, and on the growth of Dd itself were obtained for all micro-fractions generated. This enabled the generation of an extensive bioactivity profile across the whole chromatogram as displayed in Fig. 3. Results clearly indicated that micro-fractions M24-26 and M44 held most of the bioactivity measured at the NE level (red trace, Mm in infection, Fig. 3). They corresponded to the main UV-active peaks, which were also linked to the major metabolites of the NE according to the ELSD trace (ESI Fig. 1C,† ELSD chromatogram).

Additionally, micro-fractions M37 and M58 also showed moderate anti-infective activities relative to neighbouring fractions. The overlay of all three single-dose assay readouts also demonstrated that none of the fractions seemed to have any major effect on the growth of Dd (in green) nor on Mm in broth (in purple), thus confirming our observations of bioactivity on the plant NE (Fig. 1).

These bioactive micro-fractions were then subjected to UHPLC-PDA-CAD-HRMS/MS measurements to assess their content and purity. The analyses revealed that both M24-26 and M44 contained pure compounds (according to the Charged Aerosol Detector (CAD) semi-quantitative trace) and corresponded to compounds A with PI [M + H]^+^*m*/*z* of 471.1434 and C with PI [M + H]^+^*m*/*z* 697.2067 respectively. Meanwhile M37 corresponded to B, with a PI [M + H]^+^*m*/*z* of 455.1487, while M58 corresponded to D with PI [M + H]^+^*m*/*z* of 923.2673. This process clearly demonstrated that the activity of the NE was directly linked to its major stilbene constituents (Fig. 2).

The analyses of the micro-fractions revealed that the HPLC bioactivity profiling chromatographic resolution was sufficient to isolate the main constituents of the NE in amounts sufficient for preliminary biological testing. One drawback though was that it did not allow for precise weight measurements. The real potency of such stilbenes in terms of anti-infective activities was consequently hard to assess at this stage.

These results on *S. brunoniana* pushed for the evaluation of the 1600 NEs collection to find plants containing a large diversity of stilbenes, while including those highlighted in *S. brunoniana*. To do so, the chemical- and spectral space of the 1600 NEs collection was explored with dedicated computational approaches. The aim was to efficiently identify NEs rich in stilbene for targeted isolation at a scale compatible with detailed biological assessment and unambiguous structural identification of the largest possible number of stilbene derivatives.

### Search for stilbenes within the 1600 NEs collection

Navigating through the metabolomics data of the entire 1600 NEs collection was made possible thanks to the integration of this data into a KG.^7^ As explained in a previous publication,^7^ for such a large MS dataset, it was crucial to work in a *sample-centric* manner through the Experimental Natural Products Knowledge Graph (ENPKG (https://enpkg.commons-lab.org/)) workflow.^7^ Indeed, working with independent series over long periods of time is prone to batch effects. The proper alignment of features across such type of data can become challenging when not impossible.^6^

**Fig. 4:**
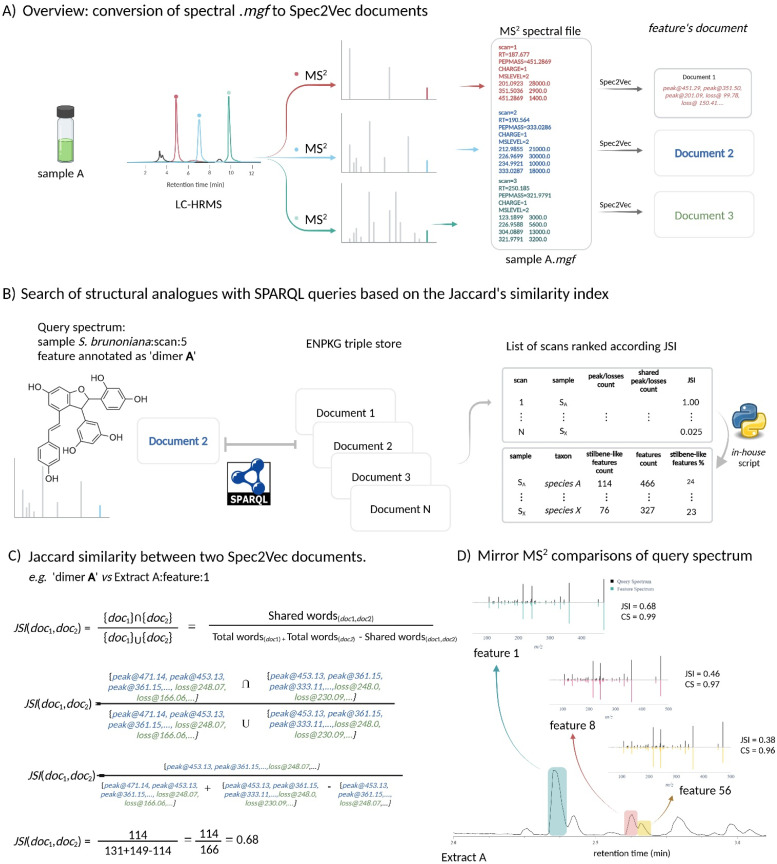
Overview of the workflow using Jaccard Similarity Indices (JSI). (A) Spectrum to document: each feature's spectral information was transformed into a document of peaks and losses using Spec2Vec within the ENPKG workflow. The peaks in the MS/MS spectra were represented as words (*e.g.*, ‘peak@xxx.xx’) and the neutral losses as ‘loss@xxx.xx’, corresponding to the mass differences between two peaks.^25^ (B) Search for structural analogues: the search for structural analogues using Jaccard Similarity Indices (JSI) began with the definition of a proxy MS/MS scan of the molecule of interest contained in the Knowledge Graph (KG). In this example, the proxy scan was the feature annotated as ‘dimer A’, focusing on stilbene-like structures. This information was used in a SPARQL query to calculate the JSI and retrieve all features within the KG that met the defined JSI threshold (JSI %3e 0.25). The results table listed each feature, with an *in-house* script grouping the features sample-wise to obtain the total count of features tagged as stilbene-like structures. (C) JSI calculation: illustration of the JSI calculation between two documents (proxy and a given scan) in the KG through the SPARQL query. (D) MS/MS spectra comparison: MS/MS spectra mirror comparison between the MS/MS of the proxy feature and three different scans in a given sample. The cosine similarity (CS) value was shown as well [link scan 5 *vs.* scan 1, mirror plot] [link scan 5 *vs.* scan 8, mirror plot].

For this, ENPKG organizes and stores all unaligned HRMS and HRMS/MS data along with associated information such as retention time, peak area, annotations (GNPS/ISDB/SIRIUS structural annotations and CANOPUS chemical class annotations), and more, on a sample-by-sample basis. The data is stored as semantic *Resource Description Framework* (RDF)-type triples (*subject-predicate-object*), allowing for incremental data storage from independent metabolite profiling series. Additionally, ENPKG can incorporate several data types like biological readouts (such as anti-infective activities) and previous phytochemical knowledge. The recovery of meaningful information from the KG is performed through federated SPARQL queries (https://www.w3.org/TR/2008/REC-rdf-sparql-query-20080115/)^24^ and in the frame of this study, is entirely reliant data from the publicly available and previously published instance of ENPKG.^7^ They allow for the retrieval and combination of data from the ENPKG triple store. In the case of the 1600 NE-collection, this corresponded to over 200 million triples that were stored (features, different types of annotations, biological readouts, …). For example, the structure and chemical class annotations from SIRIUS could be retrieved and linked to specific NEs. Of special interest in this study was the conversion of the MS/MS data of each feature into documents (*i.e*. lists) of peaks and losses through Spec2Vec^25^ as part of the ENPKG workflow. Each peak in an MS/MS spectrum was represented by the word ‘*peak@xxx.xx*’, and the neutral losses were calculated as precursor_*m*/*z*_ minus peak_*m*/*z*_ and represented as ‘*loss@xxx.xx*’ (see Fig. 4A). The peaks and losses of each MS/MS of each feature were stored as triples (*e.g*. document of feature 62 → *has_spec2vec_peak* → peak of value 923.27, …) in the KG (ESI Fig. 2†). This enabled the search and comparison of spectral fingerprints (documents) through SPARQL queries. In the context of this study and to identify NEs rich in stilbenes, a two-step strategy was envisioned, based on annotations at the chemical class level and spectral similarity. First, a SPARQL query (ESI query 1†) that counted the features annotated as ‘*oligomeric stilbenes*’ by CANOPUS^17^ was applied for each sample over the whole collection of 1600 NEs. This chemical class was chosen as the structures of compounds A–D all fell into this category according to NPClassifier.^18^ This query produced a list of 163 NEs with at least one feature annotated as ‘*oligomeric stilbene*’ (ESI Table 1†).

Then, to complement the NE selection process, a method was implemented to directly navigate the spectral space and search for stilbene fragmentation patterns based on peaks and losses in all the spectral documents stored in the ENPKG. For this, a SPARQL query applying Jaccard similarity index (JSI)^26^ was developed (see Fig. 4B). JSIs measured the similarity between two MS/MS spectra (stored as ‘*text*’ documents in the KG) by comparing their intersection with their union. The JSI is determined by dividing the number of unique common observations in two sets by the total number of unique observations in either set (see Fig. 4C). The results ranged from 0 to 1, with 0 meaning that two features are completely dissimilar and 1 meaning that they are identical features. To perform this calculation in the ENPKG, a “proxy” or “feature query” (a reference MS/MS spectrum) included in the KG had to be selected.

The spectral documents of the features dereplicated as stilbenes (dimer A, trimer C, and tetramer D) in *S. brunoniana* mentioned above were used individually as proxies in the SPARQL query. Fig. 4C provided an overview of the calculations for ‘Compound A’ as a proxy against one of the features (feature 1) of one of the samples (extract A) in the ENPKG. The Spec2Vec document of the proxy was automatically compared to that of ‘feature 1’ in sample ‘Extract A’. The intersection and union were computed, and the ratio corresponded to the Jaccard similarity index (JSI = 0.68). This calculation was repeated for all features in the ENPKG (*ca.* 1 million features). The results returned by the query included the sample codes, the scan number, retention time, and parent mass of each ‘similar’ feature in addition to the JSI. This provided a list of features that could be sorted according to JSI values. The top feature candidates were those most similar to the proxy in the whole dataset. This procedure was repeated for the other proxies of *S. brunoniana* (ESI query 2, 3, and 4†).

An in-house script (https://github.com/luigiquiros/Anti-infective-stilbenes-publication) (available on github (https://github.com/luigiquiros/Anti-infective-stilbenes-publication)) was used to combine the results of the three different queries for compounds A, C, and D, to express the results as the number of stilbene-like features per sample. Features were only considered if they had a JSI higher than the established threshold of 0.25. This value was established based on the iterative application of the same type of query over several types of compounds, as well as an observation of the statistical distribution of JSI indices for a set query (see ESI Fig. 3†). An acceptable match between the proxy and the features present in other NEs containing it, as well as structural analogues, was obtained with JSI > 0.25. To prove the acceptability of this threshold, an additional query that added the information of chemical classes for each feature when calculating JSI scores was implemented. Among the 1000 features with the highest JSI scores compared to compound A, 137 features had a JSI ≥ 0.25 (ESI Fig. 3†). Of those 137, 43 features were annotated as “oligomeric stilbenes”, 2 features were annotated as “unknown”, and the remainder (92 features) were not annotated by CANOPUS. No other chemical class than the correct “oligomeric stilbenes” appeared within the features with JSI ≥ 0.25, which suggested that the rate of “false positives” arising using this threshold is likely low.

This information was added to the list of the previously obtained CANOPUS chemical class count (ESI Table 1†). From this combined list, only the top 10% of NEs (according to their CANOPUS chemical class count), with enough dry plant material available, were considered for further analyses. This reduced the list to 14 NEs presented in Table 1. The best candidates according to the JSI query results were used to select NEs for *in depth* phytochemical analysis as shown in the following section.

**Table 1:** Table of the 14 top stilbene-containing NEs, ranked from the highest to lowest Jaccard similarity index (JSI) feature count, with the CANOPUS class count and biological readouts associated with each NE. These readouts are displayed in percentage of growth inhibition, bacterial in infection, amoeba in infection and bacterial in broth, with values reaching above 50% (corresponding to hits in the respective category) displayed in bold. *Number of entire MS/MS spectra (*i.e.* features) for which the calculated JSI > 0.25 compared to MS/MS spectra of proxy molecules

Species	Organ	CANOPUS class count	JSI feature count*	Bacterial growth inhibition in infection (%)	Amoebal growth inhibition in infection (%)	Bacterial growth inhibition in broth (%)
*Gnetum edule*	Roots	60	**36**	**52**	0	18
*Stauntonia brunoniana*	Roots	48	**33**	**76**	33	29
*Ampelocissus arachnoidea*	Multiple	95	9	3	0	0
*Gnetum edule*	Stems	36	8	20	13	1
*Shorea roxburghii*	Bark	68	5	46	0	8
*Holoptelea integrifolia*	Roots	48	5	**67**	**53**	22
*Rheum rhabarbarum*	Roots	37	5	41	10	25
*Shorea roxburghii*	Leaves	33	4	0	0	0
*Parashorea dussaudii*	Stems	32	4	0	0	13
*Rheum officinale*	Roots	35	4	34	11	27
*Hopea chinensis*	Roots	56	3	37	17	14
*Hopea chinensis*	Stems	36	3	0	0	9
*Hopea helferi*	Stems	29	3	12	8	0
*Hopea helferi*	Roots	35	2	16	3	0

### Selection of 14 stilbene-rich NEs

Together with the stilbene-related information, Table 1 also contains readouts from the biological screening for the 14 stilbene-rich NEs. Interestingly, among the 14 stilbene-rich extracts, three NEs (*Gnetum edule*, *Stauntonia brunoniana*, *Holoptelea integrifolia*) qualified as “hit NEs” (*i.e.* bacterial growth inhibition ≥50% in infection, bacterial growth inhibition <50% in broth). Besides, anti-biotic activities (Mm in broth) were generally low to non-existent, suggesting that stilbene bioactivity may be selective towards inhibiting the bacterial growth in the infection model.

*Gnetum edule* (Willd.) Blume (roots) stood out as the most promising candidate with the highest number of stilbene features and an improved bioactivity profile with lower toxicity on the host in infection (although it also has lower anti-infective activity than *S. brunoniana*). Interestingly, the richest plant in terms of CANOPUS stilbene class count was *Ampelocissus arachnoidea* with 95 ions, but this did not translate in a significant number of stilbenes flagged by the JSI metric (highlighting only 9 stilbene-like features). It can be explained by the fact that stilbenes within *A. arachnoidea* were structurally different from the stilbene proxies used to calculate JSI (according to annotations made, differing notably in the way stilbene monomers were branched together). This NE was also barely bioactive, which suggested that not all ‘*oligomeric stilbenes*’ flagged in this manner were equal in terms of biological activities. Those results thus highlighted the importance of using the second filter, which targeted the stilbene sub-structure. To explore potential differences among stilbenes themselves, a feature-based molecular network (FBMN^27^) was created using only the LC-HRMS/MS data of the 14 selected NEs.

### Exploration of the stilbene content in selected NEs

**Fig. 5:**
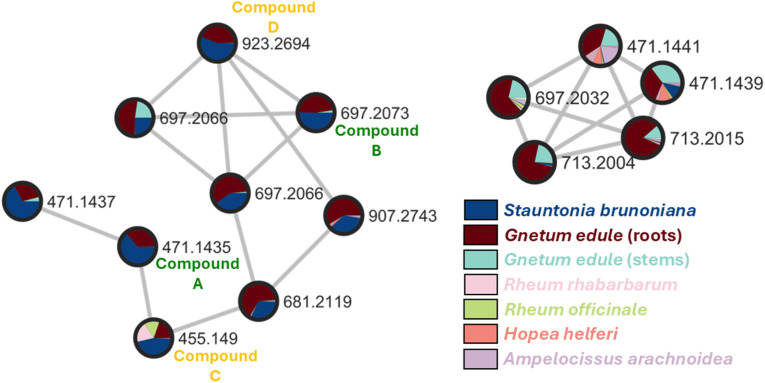
Stilbene clusters selected from the Molecular Network (MN) of the 14 stilbene-containing extracts. All four compounds annotated in Fig. 2 are found in the left-hand-side cluster (compounds A–D). Pie charts inside each node represent the relative intensity of the corresponding ion within each of the extracts they are contained in. Nodes of the left-hand-side cluster show ions most intense in two extracts: *Stauntonia brunoniana* and *Gnetum edule*. The right-hand-side cluster shows other nodes annotated as stilbenes which were mostly found in the extract of *G. edule* but were not found in *S. brunoniana*.

In this FBMN, stilbenes clustered together according to their MS/MS similarity in terms of cosine score (>0.7) and all 4 compounds flagged in *S. brunoniana* were localized in a single cluster (cluster I, Fig. 5). It appeared that these nodes were most abundant in *S. brunoniana* (represented in blue) and in *G. edule* (represented in red). This also indicated that the JSI-based spectral similarity provided similar results to the modified cosine score used in FBMN, with the advantage of being able to make comparisons over a very large collection of unaligned samples.

Interestingly, another feature (with positive *m*/*z* of 713.2004) appearing abundantly in *G. edule* was not previously identified and additional stilbene isomers, not present in *S. brunoniana*, were spotted in the same cluster (cluster II, Fig. 5). These observations were in line with the data in Table 1, suggesting that *G. edule* may have higher chemical diversity when it comes to stilbene derivatives, with 60 features identified by CANOPUS as ‘*oligomeric stilbenes*’ against only 48 in *S. brunoniana*. For all those reasons, it was decided that *G. edule* would be the best candidate to undergo an in-depth phytochemical investigation and obtain a representative set of stilbenes for bioactivity assessments.

### Isolation, structural characterization and biological evaluation of stilbene derivatives from *Gnetum edule*

**Fig. 6:**
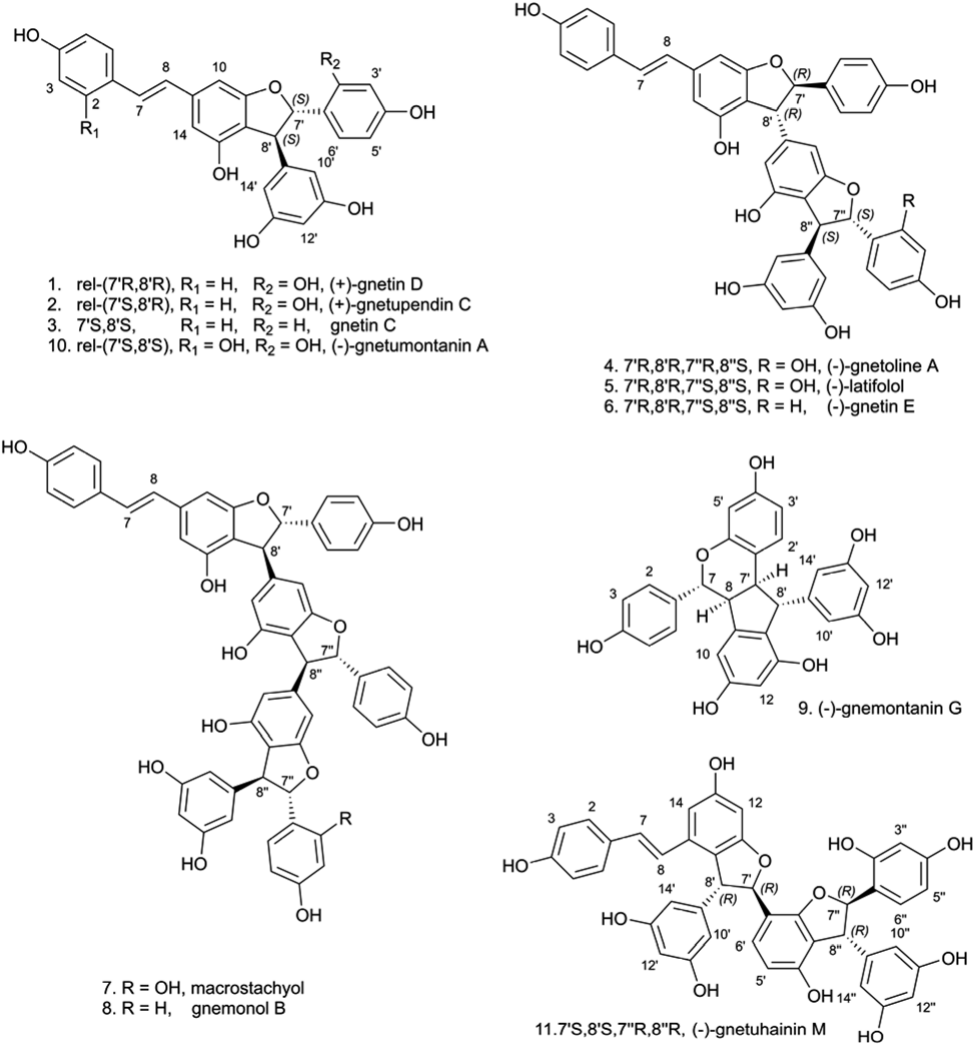
Compounds isolated from the ethyl acetate NE of *G. edule* (roots).

A thorough phytochemical study of the ethyl acetate extract of the Gnetaceae *Gnetum edule* (Willd.) Blume (roots) was carried out to corroborate the presence of stilbene-like NPs and evaluate their biological activities. The NE was separated at the gram-scale allowing for the obtention of pure compounds with enough material for full *de novo* identification and complete biological characterization. For this, fractionation was performed with flash-UV chromatography and similarly to the previous HPLC micro-fractionation process, the chromatographic conditions used were optimized at the HPLC scale and then adapted to the flash chromatography scale using a geometrical gradient transfer.^23^ This process was effective in isolating compounds as it yielded 8 pure stilbenes (1–8) out of a total of 72 fractions. Only fraction F12 needed to be further separated by semi-preparative HPLC using dry load injection^28^ and yielded 3 additional stilbenes (9–11). All isolated stilbenes were characterized by 1D and 2D NMR. Their relative stereochemistry was established using ROESY NMR. Subsequent ECD measurements/calculations were employed to determine their absolute stereochemistry (see structures in Fig. 6).

A total of six structures among those in Fig. 6 were known (1, 3, 5, 7–9) and matched previously reported ^1^H and ^13^C NMR data. Gnetin D (1, Q104666844 (https://www.wikidata.org/wiki/Q104666844)) and gnetin C (3, Q11300131 (https://www.wikidata.org/wiki/Q11300131)) were previously isolated from the woods of *Gnetum leyboldii* lianas (Q15049867 (https://www.wikidata.org/wiki/Q15049867)),^29^ latifolol (5, Q105143065 (https://www.wikidata.org/wiki/Q105143065)) was isolated from the stem of *Gnetum latifolium* (Q15050020 (https://www.wikidata.org/wiki/Q15050020)),^30^ macrostachyol A (7) was isolated from the roots of *Gnetum macrostachyum* (Q17013764 (https://www.wikidata.org/wiki/Q17013764)),^31^ gnemonol B (8, Q105384666 (https://www.wikidata.org/wiki/Q105384666)) was isolated from the roots of *Gnetum gnemon* (Q72368 (https://www.wikidata.org/wiki/Q72368))^32^ and gnemontanin G (9) was isolated from the caulis of *Gnetum montanum* Markgr. (Q10879811 (https://www.wikidata.org/wiki/Q10879811)).^33^

Among these stilbenes isolated from *G. edule*, four of them matched the four peaks annotated as stilbenes in *S. brunoniana* and serving as proxies (A–D) for this type of compounds in the whole 1600 NEs dataset. These stilbenes were gnetin D (1, compound A), gnetin C (3, compound C), latifolol (5, compound B) and macrostachyol A (7, compound D). The final structures obtained were in good agreement with the core skeleton dereplicated from the micro-fractions. The full *de novo* identification process enabled the unambiguous establishment of the stereochemistry and ascertainment of the hydroxylation pattern on this type of molecules. Comparison of the metabolite profiling on high-resolution profiles between *G. edule* and *S. brunoniana* also indicated that the retention times (RTs) and MS/MS spectra for all four reference compounds were matching between both NEs (as was shown on the FBMN, Fig. 5). All this evidence therefore confirmed that the biological activity in *S. brunoniana* was indeed linked to these four stilbenes.

Additionally, five of the isolated stilbenes were found to be isomers of known compounds, yet their stereochemistry did not match any reported structures. One of them was identified as a new stilbene and named gnetoline A (4) and four of them were enantiomers of known compounds (2, 6, 10 and 11). The absolute stereochemistry of latifolol (5) was also established here, whereas previous reports had only established its relative stereochemistry.

Compound 2 was isolated as a brown amorphous powder with an [M + H]^+^ of *m*/*z* 471.1433 corresponding to a MF of C_28_H_22_O_7_ (*Δ* = −1.06 ppm). The ^1^H and ^13^C NMR data (Table 2, Fig. S2.4. and S2.5., ESI†) showed that it corresponded to gnetupendin C,^20^ the 7′-8′ *cis* isomer of gnetin D. Both the ^3^J_H-7′-H-8′_ value of 7.7 Hz and the dipolar correlation from H-7′ to H-8′ and from H-6′ and H-10′/14′ confirmed the *cis* configuration of H-7′ and H-8′. However, the specific rotation value ([*α*]^20^_D_ +69.6 for 2 against [*α*]^20^_D_ −220.0 reported for gnetupendin C^20^) indicated that 2 was the enantiomer of (−)-gnetupendin C. The absolute configuration of the latter had not been defined previously and the Electronic Circular Dichroism (ECD) measurements carried out on compound 2 did not provide concluding information. Therefore compound 2 was identified as (+)-gnetupendin C with a *cis*-relative stereochemistry (7′*S*,8′*R* or 7′*R*,8′*S*).

**Table 2:** ^1^H and ^13^C NMR data of dimeric compounds 2 and 10

Position	2		10	
	^1^H	^13^C	^1^H	^13^C
1		128.2		115.3
2	7.41 d (8.6)	127.9		156.1
3	6.77 d (8.6)	115.5	6.33 d (2.5)	102.6
4		157.2		158.1
5	6.77 d (8.6)	115.5	6.25 dd (8.5, 2.5)	107.2
6	7.41 d (8.6)	127.9	7.35 d (8.5)	127.2
7	7.03 d (16.3)	127.9	7.19 d (16.4)	123.3
8	6.91 d (16.3)	125.7	6.85 d (16.4)	124.7
9		139.0		140.1
10	6.70 d (1.3)	98.1	6.54 d (1.3)	97.8
11		161.3		161.7
12		116.8		114.3
13		154.2		154.6
14	6.49 d (1.3)	107.3	6.44 d (1.3)	106.1
1′		114.5		118.7
2′		154.4		155.3
3′	6.16 d (2.3)	101.6	6.32 d (2.4)	102.5
4′		156.9		157.9
5′	5.94 dd (8.3, 2.3)	105.4	6.14 dd (8.4, 2.4)	105.9
6′	6.75 d (8.3)	127.4	6.84 d (8.4)	126.5
7′	5.84 d (7.7)	84.8	5.53 d (3.4)	87.6
8′	4.45 d (7.7)	48.6	4.18 d (3.4)	52.8
9′		141.6		145.7
10′	5.63 d (2.2)	106.8	6.04 d (2.2)	105.5
11′		157.1		158.1
12′	5.78 t (2.2)	100.6	6.02 t (2.2)	100.6
13′		157.1		158.1
14′	5.63 d (2.2)	106.8	6.04 d (2.2)	105.5
2-OH	—		9.59 s	
4-OH	9.55 s		9.40 s	
13-OH	9.23 s		9.20 s	
2′-OH	9.37 s		9.54 s	
4′-OH	8.97 s		9.24 s	
11′-OH	8.71 s		9.04 s	
13′-OH	8.71 s		9.04 s	

Compound 10 was also a dimer of stilbene isolated as a brown amorphous powder with an [M + H]^+^ of *m*/*z* 487.1382 corresponding to a MF of C_28_H_22_O_8_ (*Δ* = −1.03 ppm). The ^1^H and ^13^C NMR data (Table 2, Fig. S10.4. and S10.5.†) followed patterns reported for (+)-gnetumontanin A.^34^ Additional measurements distinguished 10 from (+)-gnetumontanin A: opposing specific rotation values ([*α*]^20^_D_ −13.4 for 10 against [*α*]^22^_D_ +17 reported for (+)-gnetumontanin A^34^). Compound 10 was thus identified as (−)-gnetumontanin A.

Compound 4 was a trimer of stilbene isolated as brown amorphous powder with [M + H]^+^ of *m*/*z* 697.2068 corresponding to MF of C_42_H_32_O_10_ (*Δ* = 0 ppm). Its NMR data (Table 3 and Fig. S.4.4†) showed similarities with that of latifolol (5),^30^ leading to the conclusion that they share the same planar structure. For latifolol, the relative configuration of each dihydrofurane ring was identified as *trans* based on the ^3^*J*_H-7′–H-8′_ and ^3^*J*_H-7′′–H-8′′_ values (4.0 and 4.2 Hz, respectively) and from the ROESY correlations from H-7′ (or H-7′′) to H-10′/H-14′ (or H-10′′/14′′) and from H-8′ (or H-8′′) to H-2′/6′ (or H-2′′). For compound 4, the relative configuration was defined as *trans* for H-7′ and H-8′ (^3^*J*_H-7′–H-8′_ = 3.9 Hz; and same ROESY as those described for latifolol), and *cis* for H-7′′ and H-8′′ (^3^*J*_H-7′–H-8′_ = 7.9 Hz; and ROESY correlations from H-6′′ to H-10′′/14′′). As the absolute configuration of latifolol (5) did not seem to have been defined previously, ECD calculations were carried out for the 4 possible isomers and compared with the experimental data (S5.1†). The best fit was observed for the 7′*R*,8′*R*,7′′*S*,8′′*S* isomer, for which the specific rotation value −12.4 (*c* 0.12, MeOH) was measured (literature:^30^ [*α*]^20^_D_ −42 (*c* 0.15, MeOH)). The absolute configuration of latifolol (5) was thus defined here as (*E*)-7′*R*,8′*R*,7′′*S*,8′′*S* for the first time. For compound 4, the presence of an unidentified stilbene dimer mixed with it meant that potential distortions in the ECD spectrum could be expected. The experimental ECD (S4.1†) trace did match that of the 7′*R*,8′*R*,7′′*R*,8′′*S*-configuration and compound 4 was thus identified as a new (*E*)-7′*R*,8′*R*,7′′*R*,8′′*S*-isomer of latifolol (5) and was named gnetoline A (4).

**Table 3:** ^1^H and ^13^C NMR data of trimeric compounds 4, 6 and 11

Position	4		6		11	
	^1^H	^13^C	^1^H	^13^C	^1^H	^13^C
1		128.1		128.1		128.0
2	7.42 d (8.6)	127.9	7.41 d (8.7)	127.9	7.09 d (8.7)	127.7
3	6.76 d (8.6)	115.5	6.76 m	115.5	6.68 d (8.7)	115.5
4		157.3		157.3		157.3
5	6.76 d (8.6)	115.5	6.6 m	115.5	6.68 d (8.7)	115.5
6	7.42 d (8.6)	127.9	7.41 d (8.7)	127.9	7.09 d (8.7)	127.7
7	7.03 d (16.3)	128.2	7.03 d (16.3)	128.2	6.83 d (16.3)	128.8
8	6.92 d (16.3)	125.5	6.91 d (16.3)	125.5	6.58 d (16.3)	122.0
9		139.7		139.7		134.7
10	6.68 d (1.1)	97.7	6.68 d (0.9)	97.8		118.9
11		161.5		161.5		160.8
12		113.9		113.9	6.23 d (2.0)	96.0
13		154.6		154.6		158.4
14	6.50 d (1.1)	107.3	6.50 d (0.9)	107.3	6.59 d (2.0)	102.9
1′		132.3		132.2		112.8
2′	7.16 d (8.6)	126.7	7.15 d (8.6)	126.8		158.9
3′	6.76 d (8.6)	115.3	6.76 m	115.3		115.1
4′		157.2		157.3		154.9
5′	6.76 d (8.6)	115.3	6.76 m	115.3	6.31 d (8.5)	108.6
6′	7.16 d (8.6)	126.7	7.15 d (8.6)	126.8	7.05 d (8.5)	127.6
7′	5.40 d (3.9)	91.9	5.40 d (4.0)	91.9	5.61 d (6.6)	88.2
8′	4.35 d (3.9)	54.2	4.34 d (4.0)	54.2	4.65 d (6.6)	53.9
9′		144.5		145.1		145.5
10′	6.19 s	99.7	6.16 d (1.3)	99.4	6.10 d (2.2)	105.7
11′		160.9		161.0		158.6
12′		115.8		113.1	6.07 t (2.2)	101.1
13′		154.2		154.5		158.6
14′	6.15 s	107.3	6.13 d (1.3)	107.1	6.10 d (2.2)	105.7
1′′		114.6		131.7		118.6
2′′		154.3	7.10 d (8.6)	127.2		154.8
3′′	6.13 d (2.3)	101.6	6.76 m	115.3	6.32 d (2.5)	102.4
4′′		156.8		157.2		157.6
5′′	5.93 dd (8.4, 2.3)	105.4	6.76 m	115.3	6.07 dd (8.4, 2.5)	105.9
6′′	6.75 d (8.4)	127.4	7.10 d (8.6)	127.2	6.55 d (8.4)	125.8
7′′	5.82 d (7.9)	85.0	5.27 d (5.7)	92.4	5.54 d (2.7)	88.2
8′′	4.42 d (7.9)	48.5	4.22 d (5.7)	54.3	4.10 d (2.7)	53.3
9′′		141.6		144.7		145.5
10′′	5.63 d (2.2)	106.8	5.97 d (2.2)	105.4	6.08 d (2.2)	105.6
11′′		157.1		158.3		158.1
12′′	5.78 t (2.2)	100.5	6.03 t (2.2)	100.9	6.04 t (2.2)	100.7
13′′		157.1		158.3		158.1
14′′	5.63 d (2.2)	106.8	5.97 d (2.2)	105.4	6.08 d (2.2)	105.6
4-OH	9.55 s		9.49 s or 9.56 s		9.57 s	
13-OH	9.40 s		9.40 s		9.37 s	
4′-OH	9.48 s		9.49 s or 9.56 s		9.37 s	
11′-OH	—		—		9.15 s	
13′-OH	9.18 s		9.23 s		9.15 s	
2′′-OH	9.33 s		—		9.53 s	
4′′-OH	8.95 s		9.49 s or 9.56 s		9.17 s	
11′′-OH	8.72 s		9.10 s		9.07 s	
13′′-OH	8.72 s		9.10 s		9.07 s	

Compound 6 was also a trimer of stilbene isolated as brown amorphous powder with [M + H]^+^ of *m*/*z* 681.2120 corresponding respectively to a MF of C_42_H_33_O_9_ (*Δ* = 0.15 ppm). The ^1^H and ^13^C NMR data (Table 3, Fig. S6.4. and S6.5.†) followed patterns of *trans*,*trans*-configuration reported for gnetin E,^29^ with no absolute configuration established in previous reports. ECD measurements (S6.1†) were therefore carried out to establish the absolute configuration 7′*R*,8′*R*,7′′*S*,8′′*S* for (−)-gnetin E (6) for the first time.

**Fig. 7:**
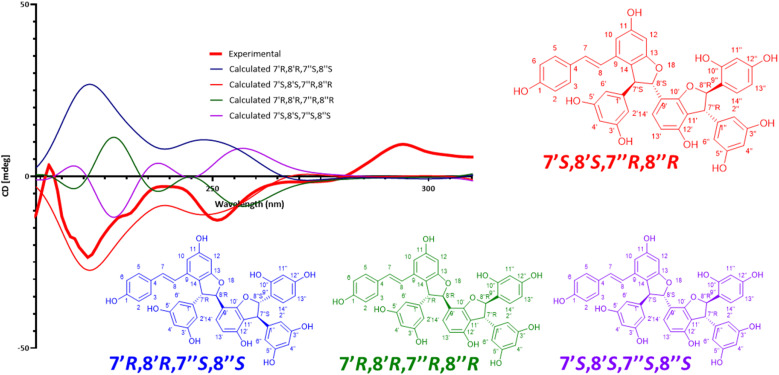
Measured and calculated ECD traces for (−)-gnetuhainin M. Experimental ECD trace measured for compound 11, compared to traces calculated for the four possible absolute stereoisomers using time-dependent density functional theory (TD-DFT) calculations. The final absolute configuration established for (−)-gnetuhainin M is (*E*)-7′*S*,8′*S*,7′′*R*,8′′*R*.

Compound 11 is a trimer of stilbene with an [M + H]^+^ of *m*/*z* 713.2018 corresponding to a MF of C_42_H_32_O_11_ (*Δ* = 0.14 ppm). The NMR data (Table 3, Fig. S11.4. and S11.5.†) was similar to that reported for (−)-gnetuhainin M.^35^ Measurements of specific rotation values ([*α*]^20^_D_−44.9 for 11 against [*α*]^25^_D_ −32.9 reported for (−)-gnetuhainin M) also suggested that the isolated compound matched with the reported (−)-gnetuhainin M.^35^ Only the *trans*,*trans* relative stereochemistry was established in that report however, there were still four different absolute stereoisomers possible (Fig. 7). The experimental ECD trace was compared to the four traces of the possible absolute stereoisomers calculated using time-dependant density functional theory (TD-DFT).^36–38^ The experimental trace matched that of isomer 7′*S*,8′*S*,7′′*R*,8′′*R*, allowing for the assignment of the absolute stereochemistry (*E*)-7′*S*,8′*S*,7′′*R*,8′′*R* for (−)-gnetuhainin M (Fig. 7) for the first time.

### Biological activity assessment of isolated compounds

**Fig. 8:**
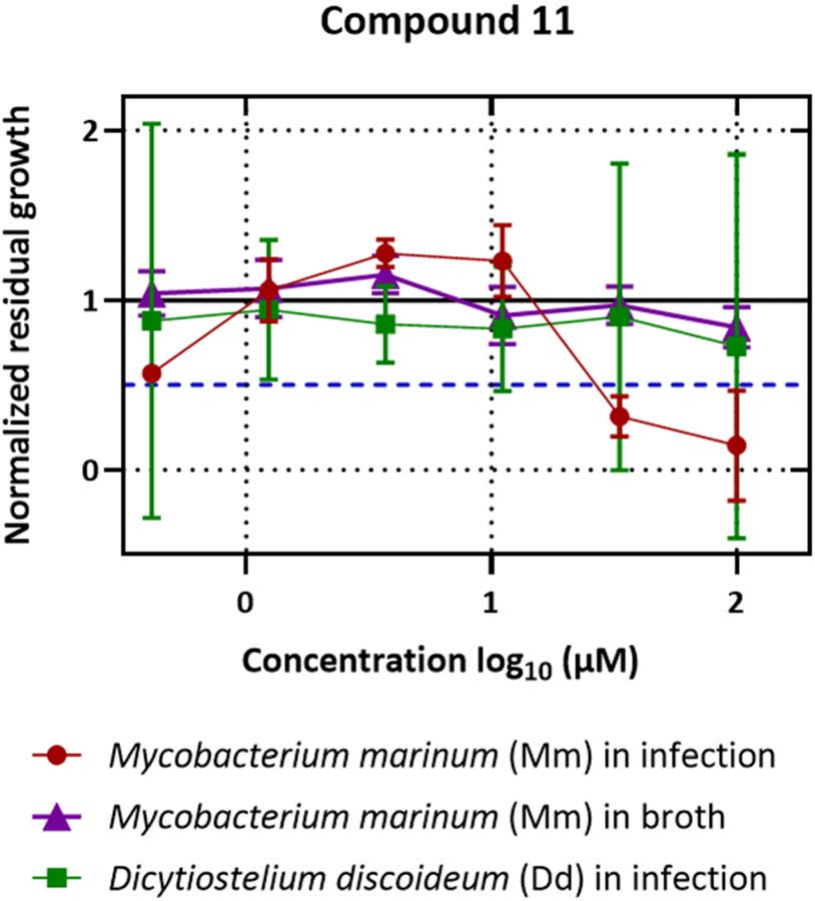
Dose–response curves for anti-infective and anti-biotic assays on compound 11 ((−)-gnetuhainin M) with median values. In red: bacterial growth in infection; in green: amoeba growth in infection; concentrations recorded (from left to right): 0.41 μM, 1.23 μM, 3.70 μM, 11.1 μM, 33.3 μM and 100 μM.

Biological activity assessment on individual compounds confirmed the expected anti-infective activities observed at the NE level. Most of the dose–response curves of these compounds followed a similar pattern as for compound 11 (Fig. 8): while they displayed anti-infective activity at high concentrations (100 μM), at intermediate concentrations (11.1 μM and 3.7 μM) a slight pro-infective effect was observed, which was not visible at the lowest concentration (0.4 μM). IC_50_ values for these compounds ranged from >100 μM to 22.22 μM (Table 4).

**Table 4:** IC_50_ (Mm in infection, Dd in infection and Mm in broth) of the 11 stilbenes isolated from *G. edule*

Compound	IC_50_ Mm in infection (μM)	IC_50_ Dd in infection (μM)	IC_50_ Mm in broth (μM)
1	>100	>100	>100
2	66.67	>100	>100
3	>100	>100	>100
4	66.67	66.67	>100
5	66.67	66.67	>100
6	66.67	>100	>100
7	66.67	66.67	>100
8	66.67	66.67	>100
9	>100	>100	>100
10	>100	>100	>100
11	22.22	>100	>100

The biological readouts of single compounds did align well with the biological readout expected at the NE level. In fact, none of these compounds showed any activity on Mm in broth and their effect on the growth of the host Dd remained largely limited, qualifying most of them as strict anti-infectives, as was the case for the NE. IC_50_ values of isolated compounds were generally high (*i.e.* lower activity), compared to the strong activities observed in fractions from the HPLC bioactivity profiling. This can be attributed to the inherent drawback of the latter approach, in that it doesn't allow for precise weight measurements of micro-fractions. In that process, all micro-fractions are tested at a single concentration according to an average weight (calculated as the total amount of extract injected divided by the number of micro-fractions). This induced increased uncertainties, resulting in bioactivities being likely overestimated for major compounds of an extract.

**Fig. 9:**
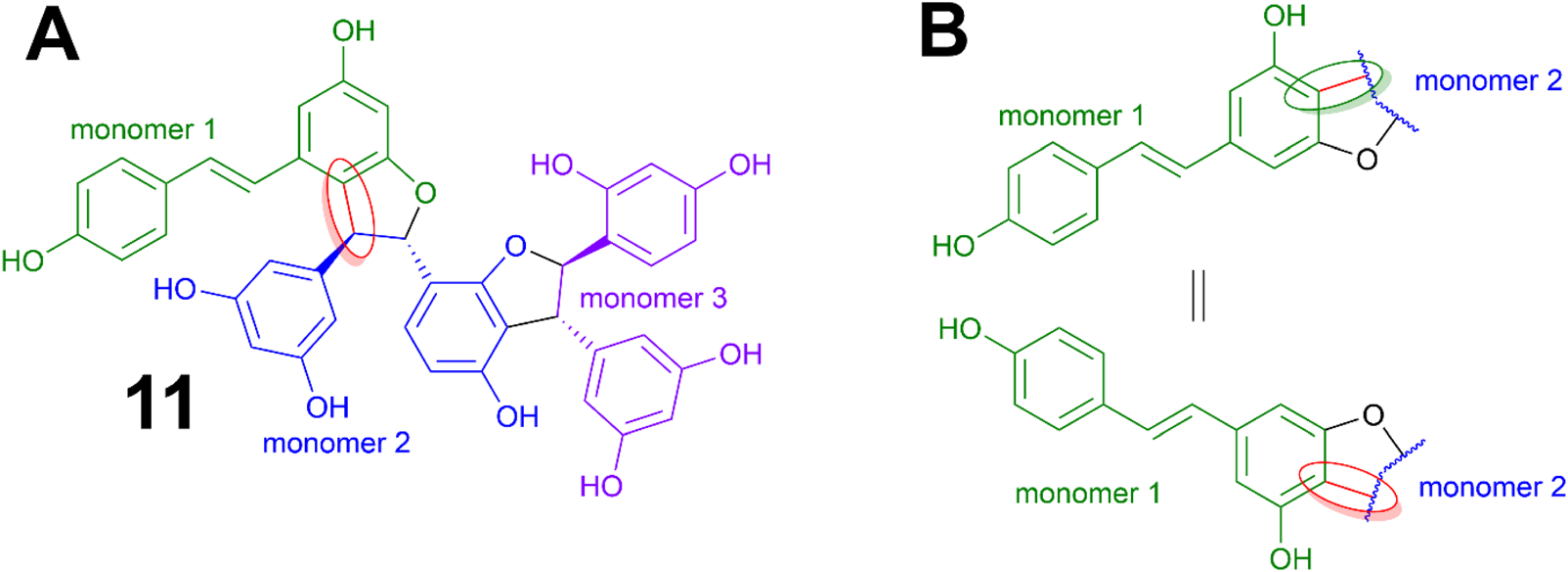
Difference in linkage between monomers in the isolated structures. (A) Linkage between monomers 1 and 2 in compound 11. (B) Linkage between monomers 1 and 2 in other compounds isolated (except for compound 9).

In absolute terms, compound 11 was the best candidate for anti-infective activities with an IC_50_ of 22.22 μM. In terms of its chemical structure, it was also an outlier compared to other compounds, which was observable in the form of its linkage between monomers 1 and 2 (Fig. 9). It was fair to assume therefore that this structural difference may have had an impact on the activity levels observed. Additionally, in spite of their appearance as complex three-dimensional entities, these compounds are in fact fairly rigid in terms of conformational movements.

### Stilbenes in other stilbene-rich NEs

**Fig. 10:**
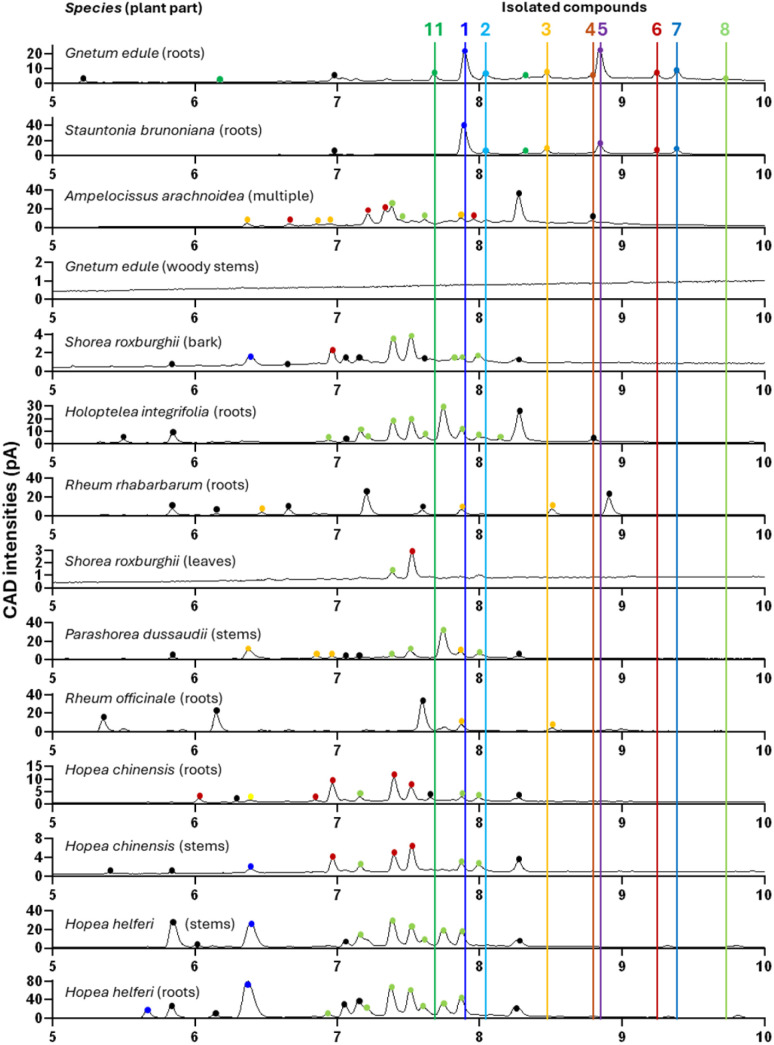
Abundances of stilbene oligomers in the selection of 14 stilbene-rich plant NE UHPLC-Charged Aerosol Detection (CAD) traces for each NE injected at 5 mg mL^−1^. *Y*-Axis represents CAD intensities (in pA), *x*-axis represents the retention time (26 min runs, zoom on the zone of retention of stilbenes 5–10 min). Each dot represents an ion corresponding to an isolated compound (or an isomer thereof) according to its colour. Black dots represent *m*/*z* that did not correspond to any isolated compounds but corresponded to features dereplicated as stilbene.

The various isolated stilbenes served as references to map the stilbene content in the other selected NEs (Table 4) labelled as stilbene-rich within the 1600 NEs (see Fig. 10). For this purpose, the 14 NEs were re-analysed by UHPLC-PDA-CAD-HRMS/MS using a profiling method with longer gradient time to increase the chromatographic peak capacity and facilitate accurate comparisons. Fig. 10 presents the semi-quantitative Charged Aerosol Detection (CAD) traces for each of the selected NEs, with a focus on the chromatographic window of stilbene elution (5–10 min). Major peaks found in each NE were marked by a dot coloured according to whether their *m*/*z* corresponded to any of the isolated compounds (tagged by a vertical line). These colored dots highlighted isomers of isolated compounds, while black dots corresponded to features dereplicated as stilbene derivatives, without their *m*/*z* matching any of the isolated compounds.

Clearly, all 14 NEs contained an abundance of stilbenes. In the case of *G. edule*, they were clearly abundant in the roots, in the woody stems however they were not detectable on the CAD trace, but the MS detection revealed their presence. The CAD semi-quantitative analysis revealed that there seemed to be no direct correlation between the general abundance of stilbenes and the biological activity of the NE. In fact, this was also reflected in the isolated stilbenes, exhibiting very variable levels of anti-infective activity. The alignment of all CAD traces revealed that most of the compounds isolated from *G. edule* (roots) and those flagged in *S. brunoniana* seemed to be specific to these two NEs, with many stilbenes not appearing in any other NE (2, 4, 5, 7, 11). The other stilbene-rich plants highlighted by the JSI search in the KG were likely to contain other forms of stilbene (generally more polar), eluting before 8 minutes.

## Conclusion

This study has shown how through organizing data into a KG framework and using adequate SPARQL queries, it was possible to distinguish stilbene features within over 1 million features of a 1600 NEs collection. Using tools as simple as Jaccard indices along with the MS/MS data stored in the KG, it was possible to make a rational selection of a few stilbene-rich NEs out of a large library. In fact, even when dealing with uncertain annotations or an unknown class of compounds, this approach should work for any type of compounds of interest as it relies directly on experimental MS/MS data.

Considering the significant size of the dataset and unlike in MNs approaches, the exploration of the spectral space was based on MS/MS data stored through the Spec2Vec pipeline. This is computationally friendly format, which comes with an inherent drawback being the absence of intensity values in the resulting data format (documents). The JSI score developed in this study was indeed effective in handling such format. One major resulting pitfall, highlighted by low JSI value, was that the noise in the MS/MS spectra could not be filtered out based on intensity values in the current KG. Future developments of the KG will aim to overcome this problem by integrating the MS/MS peaks intensities information during raw data processing. Such adaptation should allow direct filtering based on spectral intensities, a critical parameter that was lost in spectral document conversion. As shown however, this did not impede the relevance of JSI scores to successfully highlight chemical similarity across samples. This enabled the prioritization of extracts similar in composition to a reference extract with annotated bioactive LC-peaks as proxies.

This approach also yielded chemical novelty, with 6 newly characterized isomers out of 11 chemical entities isolated from *G. edule*. (−)-Gnetuhainin M (11) in particular stood out from all other compounds, not only due to its specific structural features, but also for its anti-infective activity being significantly higher, with an IC_50_ of 22.22 μM for the inhibition of bacterial growth in infection. These structures were reminiscent of *trans*-δ-viniferins, which are another type of stilbene dimers widely reported for various biological activities,^39^ including anti-infective effects. Furthermore, studies on *trans*-δ-viniferins obtained by biotransformation showed that *O*-methylation on some of the phenol groups appeared to have a beneficial effect on the anti-infective activity, confirming interest for this type of scaffold.^11^ Selective *O*-methylation of (−)-gnetuhainin M (11) might therefore lead to further improvement of its anti-infective activity.

An additional observation concerning biological activities was the pro-infective effect observed with individual compounds at mid-level concentrations, which remained largely unexplained. This pro-infective effect was reminiscent of the behaviour displayed by rapamycin (ESI Fig. 4†), a known autophagy inducer (inhibitor of the mTOR pathway which negatively regulates autophagy).^40^ Further mechanism of action studies on this class of compounds might provide additional information to explain such a phenomenon. Altogether, the combination of metabolomic data in a KG with stringent *in cellulo* anti-infective assays holds promises to highlight other scaffolds of interest in the chemical/spectral space of large metabolomic datasets.

## Experimental section

### General experimental procedures

UV spectra were recorded on a JASCO J-815 spectrometer (Loveland, CO, USA) in MeOH, using a 1 cm cell. The scan speed was set at 200 nm min^−1^ in continuous mode between 500 and 205 nm, with a bandwidth of 1 nm, a data pitch of 1 nm and 3 accumulations. NMR data were collected on a Bruker Avance III HD 600 MHz NMR spectrometer equipped with a QCI 5 mm cryoprobe and a SampleJet automated sample changer (Bruker BioSpin, Rheinstetten, Germany). Chemical shifts are presented in parts per million (*δ*), referencing the residual DMSO-d_6_ signal (*δ*_H_ 2.50; *δ*_C_ 39.5) as internal standards for ^1^H and ^13^C NMR, respectively, with coupling constants (*J*) reported in Hz. Full assignments were determined through 2D NMR experiments (COSY, ROESY, HSQC, and HMBC). HRMS data were acquired using a Orbitrap Exploris 120 mass spectrometer (Thermo Scientific, Germany) with a heated electrospray (H-ESI) source. Fraction contents were monitored using a multi-detection UHPLC-PDA-ELSD-MS platform (Waters) equipped with a single quadrupole detector and heated electrospray ionization. Analytical HPLC utilized an HP 1260 Agilent system with a photodiode array detector (Agilent Technologies, Santa Clara, CA, USA). Semipreparative HPLC was conducted on a modular system (Puriflash-MS 4250, Interchim, Montluçon, France) equipped with a quaternary pump, a UV detector module and a fraction collector.

### Plant material

The plants containing the compounds of interest were *Stauntonia brunoniana* (Decne.) Wall. ex Hemsl. (Lardizabalaceae) and *Gnetum edule* (Willd.) Blume (Gnetaceae). These plants belong to the Pierre Fabre Laboratories (PFL) collection with over 17 000 unique samples collected worldwide. The PFL collection was registered at the European Commission under the accession number 03-FR-2020. This registration certifies that the collection meets the criteria set out in the EU ABS Regulation which implements at EU level the requirements of the Nagoya Protocol regarding access to genetic resources and the fair and equitable sharing of benefits arising from their utilization (Sharing nature, 2022). PFL supplied all the vegetal material (ground dry material). Plant materials were dried for 3 days at 55 °C in an oven; then the material was ground and stored in plastic pots at controlled temperature and humidity in PFL facilities. All references also exist as intact samples for later identification purposes when needed. For both plants used in this study, their roots were the chosen plant part used, with their following unique ID within the PFL collection: V113270 (*S. brunoniana* roots) and V113056 (*G. edule* roots).

### Plant extraction

The plant material was extracted in a Thermo Scientific Dionex ASE 350 Accelerated Solvent Extractor. The roots of *Stauntonia brunoniana* (37.81 g) and *G. edule* (71.04 g) were extracted in a 100 mL pressure-resistant stainless steel extraction cell using the ASE system. At the bottom and the top of the cell, a cellulose filter (Dionex™) was added to prevent solid particles from reaching the system. The cell was loaded in the tray then pressurized and extracted with hexane, ethyl acetate and methanol respectively. The rinse volume was set at 60% and the temperature at 40 °C, with 3 cycles for each solvent (6 cycles for the ethyl acetate extracts) and a static time set at 5 min. The resulting NEs were collected in round bottom flasks, combined and evaporated to dryness on a rotary evaporator (Büchi Rotavapor R114™ Labortechnik AG, Switzerland) for each solvent to constitute the final NEs. The following extraction yields were obtained: *S. brunoniana* hexane 81 mg (0.21%), ethyl acetate 442.78 mg (1.17%), methanol 2.0928 g (5.54%); *G. edule* hexane 227.2 mg (0.32%), ethyl acetate 1.2782 g (1.80%), methanol 7.3275 mg (10.3%).

### UHPLC-PDA-ELSD-MS analyses of fractions

Aliquots (50 μL) of fractions obtained by HPLC-microfractionation, flash chromatography and semi-preparative HPLC were analysed by UHPLC-PDA-ELSD-MS. The conditions for the ESI (Waters Acquity QDA Detector) were set as follows: capillary voltage 0.8 kV (negative ion mode) or 1.2 kV (positive ion mode), cone voltage 15 V, probe temperature 600 °C, source temperature 120 °C. Detection was performed in negative- (NI) then positive ion mode (PI) with an *m*/*z* range of 150–1250 Da. The separation was done on an Acquity UPLC BEH C18 column (50 × 2.1 mm i.d., 1.7 μm; Waters) at 0.6 mL min^−1^, 40 °C with H_2_O (A) and MeCN (B) both containing 0.1% formic acid (FA). The following gradient was applied for the separation: from 5 to 100% of B from 0 to 7 min, 1 min at 100% B, and a re-equilibration step of 2 min. The ELSD was set at 45 °C, with a gain of 3. The PDA detector (Waters Acquity) was set in the range from 190 to 500 nm, with a resolution of 1.2 nm. Sampling rate was set at 20 points per s.

### UHPLC-DAD-CAD-HRMS/MS of NEs, fractions and pure compounds

Analyses were performed with a Waters Acquity UHPLC system coupled to a Corona Veo RS Charged Aerosol Detector (CAD, Thermo Scientific, Germany) and an Orbitrap Exploris 120 mass spectrometer (Thermo Scientific, Germany). The Orbitrap employed a heated electrospray ionization source (H-ESI) with the following parameters: spray voltage: +3.5 kV; ion transfer tube temperature: 320.00 °C; vaporizer temperature: 320.00 °C; S-lens RF: 45 (arb units); sheath gas flow rate: 35.00 (arb. units); sweep gas (arb.): 1 and auxiliary gas flow rate: 10.00 (arb. units). Control of the instruments was done using Thermo Scientific Xcalibur software v. 4.6.67.17. Full scans were acquired at a resolution of 30 000 fwhm (at *m*/*z* 200) and MS2 scans at 15 000 fwhm in the range of 100–1000 *m*/*z*, with 1 microscan, time (ms): 200, an RF lens (%): 70; AGC target custom (normalized AGC target (%): 300); maximum injection time (ms): 130; microscans: 1; data type: profile; use EASY-IC(TM): ON. The settings for dynamic exclusion mode were customized; exclude after *n* times: 1; exclusion duration (s): 5; mass tolerance: ppm; low: 10, high: 10, exclude isotopes: true. Appex detention: desired apex window (%): 50. Isotope exclusion: assigned and unassigned with an exclusion window (*m*/*z*) for unassigned isotopes: 8. The intensity threshold was set to 2.5 × 10^5^ and a targeted mass exclusion list was used.

The centroid data-dependent MS2 (dd-MS2) scan acquisition events were performed in discovery mode, triggered by apex detection with a trigger detection (%) of 300 with a maximum injection time of 120 ms, performing 1 microscan. The top 3 abundant precursors (charge states 1 and 2) within an isolation window of 1.2 *m*/*z* were considered for MS/MS analysis. For precursor fragmentation in the HCD mode, a normalized collision energy of 15, 30 and 45% was used. Data was recorded in profile mode (use EASY-IC(TM): ON).

The chromatographic separation was done on a Waters BEH C18 column (50 × 2.1 mm i.d., 1.7 μm, Waters, Milford, MA) using the following gradient (time (min), % B): 5% B from 0 to 0.5 min; from 5% B to 100% B between 0.5 and 7 min; 100% B from 7 to 8 min, from 100% B to 5% B from 8 to 8.10 min; 5% B from 8.10 to 10 min. The mobile phases were H_2_O (A) and MeCN (B) both containing 0.1% FA. The flow rate was set to 600 μL min^−1^, the injection volume was 2 μL and the column was kept at 40 °C. The PDA detector was used from 210 to 400 nm with a resolution of 1.2 nm. The CAD detector was kept at 40 °C, with 5 bar N_2_ and power function 1 for a data collection rate of 20 Hz.

### HPLC-PDA analysis of the ethyl acetate extract of *S. brunoniana* prior to microfractionation

The analysis of the ethyl acetate extract of *S. brunoniana* (roots) was carried out using an HP 1260 system equipped with a photodiode-array (PDA) detection unit from Agilent Technologies in Santa Clara, CA, United States. An XBridge BEH C18 column (250 × 4.6 mm i.d., 5 μm, Waters®) was employed. Detection was performed using a PDA, with UV wavelengths set at 214, 254, 280 and 329 nm. UV spectra between 190–500 nm were recorded with a threshold of 10 mAU and setting increments of 2 nm. HPLC conditions involved a mobile phase of H_2_O (A) and MeCN (B), both containing 0.1% FA. The flow rate was set at 1 mL min^−1^, with an injection volume of 10 μL. The separation temperature was maintained at 25 °C, and the sample concentration was 10 mg mL^−1^ dissolved in DMSO. The gradient slope was set as follows: an initial gradient flow of 30–48% of B in 24 min, followed by a gradient flow of 1 min from 48–100% B and a final rinsing hold of 5 min at 100% B.

### Semi-preparative HPLC-PDA microfractionation of the ethyl acetate extract of *S. brunoniana*

The separation of the ethyl acetate extract of *S. brunoniana* (roots) was carried out on 13.5 mg dissolved in 200 μL DMSO using an HPLC 1260 system equipped with a photodiode-array (PDA) detection unit from Agilent Technologies in Santa Clara, CA, United States. An XBridge BEH C18 column (250 × 10 mm i.d., 5 μm, Waters®) was employed. Detection was performed using a PDA, with UV wavelengths set at 214, 254, 280 and 329 nm. UV spectra between 190–500 nm were recorded with a threshold of 10 mAU and setting increments of 2 nm. HPLC conditions involved a mobile phase of H_2_O (A) and MeCN (B), both containing 0.1% FA. The flow rate was set at 4.7 mL min^−1^, with an injection volume of 200 μL. The separation temperature was maintained at 25 °C, and the sample concentration was 135 mg mL^−1^ dissolved in DMSO. The gradient slope was set as follows: an initial gradient flow of 30–48% of B in 24.39 min, followed by a gradient flow of 1 min from 48–100% B and a final rinsing hold of 4.93 min at 100% B. Those conditions were obtained through gradient transfer^23^ based on an optimized analytical run of 30 min. The total separation time was set to about 30 minutes so that the effluent could be effectively collected as 92 micro-fractions of about 1400 μL each that were all dried and individually tested for their biological activity.

### HPLC-PDA gradient optimizations on crude NE for flash chromatography

The analysis of the ethyl acetate extract of *G. edule* (roots) was carried out using an HP 1260 system equipped with a photodiode-array (PDA) detection unit from Agilent Technologies in Santa Clara, CA, United States. An InterChim® Puriflash HQ C18 column (250 × 4.6 mm i.d., 15 μm, Moluçon, France) was employed. Detection was performed using a PDA, with UV wavelengths set at 214, 254, 280 and 329 nm. UV spectra between 190–500 nm were recorded with a threshold of 10 mAU and setting increments of 2 nm. HPLC conditions involved a mobile phase of H_2_O (A) and MeOH (B), both containing 0.1% FA. The flow rate was set at 1 mL min^−1^, with an injection volume of 10 μL. The separation temperature was maintained at 25 °C, and the sample concentration was 10 mg mL^−1^ dissolved in MeOH. The gradient slope was set as follows: an initial hold of 1 min at 40% B, gradient flow of 40–45.5% of B in 9 min, followed by a hold of 2 min at 45.5%, then another gradient flow of 45.5–47% in 3 min, followed by a hold of 2 min at 47% B, then a gradient flow of 47–52% in 10 min, followed by a hold of 2 min at 52%, before another gradient flow from 52–54% in 4 min, followed by a hold of 2 min at 54%, then a gradient flow of 54–70% in 15 min, before another gradient flow of 70–100% in 1 min, ending with a 9 min washing step at 100% B. These optimized HPLC analytical conditions were geometrically transferred by gradient transfer to the flash-LC scale.^23^

### Flash-UV chromatography on the ethyl acetate extract of *G. edule*

The ethyl acetate extract of *G*. *edule* (roots) was purified with a Büchi Flash chromatography system (Büchi Pump Module C-605, UV Photometer C-640, Control Unit C-620, Fraction Collector C-660), using an InterChim® Puriflash HQ C18 column (120 g, 210 × 30 mm i.d., 15 μm, Moluçon, France). 1.01 g of NE were mixed in the stationary phase (C18 Zeoprep® 40–63 μm) and sand (50–70 mesh particle size) in a proportion of 1 : 1 : 1 and then introduced in a dry load cell. The detection was performed by a UV photometer with parameters set as follows: UV wavelengths at 214, 254, 280, 329 nm. The mobile phase was composed of MilliQ H_2_O (A) and technical grade MeOH (B), both containing 0.1% FA (flow rate: 30 mL min^−1^). The gradient slope was set as follows: an initial hold of 1 min at 40% B, gradient flow of 40–45.5% of B in 13 min, followed by a hold of 3 min at 45.5%, then another gradient flow of 45.5–47% in 4 min, followed by a hold of 3 min at 47% B, then a gradient flow of 47–52% in 15 min, followed by a hold of 2 min at 52%, before another gradient flow from 52%–54% in 6 min, followed by a hold of 3 min at 54%, then a gradient flow of 54–70% in 19 min, before another gradient flow of 70–100% in 2 min, ending with a 13 min washing step at 100% B. The separation yielded 72 fractions of 50 mL each (F01–F72) that were dried using a multi-units evaporator (Multivapor™, Büchi Labortechnik AG, Switzerland). The following compounds were identified after HRMS and NMR analyses for confirmations: 1 (F15–18, 158.2 mg, RT 13–19 min), 2 (F21–22, 11 mg, RT 22.5–23.5 min), 3 (F25, 14.1 mg, RT 27–28.5 min), 4 (F28, 6.3 mg, RT 32–32.5 min), 5 (F29–34, 103.2 mg, RT 32.5–40 min), 6 (F41–42, 18.2 mg, RT 51–54 min), 7 (F44–45, 19.4 mg, RT 56–59 min), 8 (F49, 4.6 mg, RT 64–66 min), 9–11 (required further purification, F12, 15.3 mg, RT 10–11 min).

### Semi-preparative HPLC-UV on selected flash fraction

Compound 11 was identified by NMR as the major component in F12 but required further purification. This fraction was subjected to semi-preparative HPLC-UV using a Shimadzu system equipped with an LC-20 A module pumps, an SPD-20 A UV/VIS, a 7725I Rheodyne® valve, and an FRC-40 fraction collector (Shimadzu, Kyoto, Japan). The system was controlled by the LabSolutions software, also from Shimadzu. The fraction was dissolved in 200 μL of MeOH and was mixed with the stationary phase (C18 Zeoprep® 40–63 μm, one spatula), to form uniform slurries. MeOH was evaporated to obtain fine powders which were introduced in a dry-load cell according to our previously published method.^28^ The separation was performed with an XBridge BEH C18 OBD Prep column (250 × 19 mm i.d., 5 μm, Waters®). The mobile phase was composed of MilliQ-grade H_2_O with 0.1% FA (A) and HPLC-grade MeCN with 0.1% FA (B) and the flow rate was set at 17 mL min^−1^. Fractions were collected using 220 and 280 nm UV signals and the gradient slope was set as follows: gradient flow from 15–40% B in 50 min, followed by a gradient from 40–100% B in 1 min, ending with a 9 min washing step at 100% B. The fractions collected (10 mL each) were evaporated to dryness using a Büchi rotary-evaporator system (Büchi Rotavapor R114™ Labortechnik AG, Switzerland). The following compounds were identified after HRMS and NMR analyses for confirmations: 9 (0.9 mg, RT 24.5–25 min), 10 (0.6 mg, RT 29.5–30 min), 11 (1.7 mg, RT 33.5–34.5 min).

### Bioactivity screening

Plant NEs, fractions or isolated compounds were stored at −20 °C and wrapped in aluminum foil if necessary. All manipulations with NEs, fractions or compounds were performed under a sterile hood. NEs, fractions and compounds were throughout resuspended in DMSO, to best solubilise NEs with diverse constituents. NEs, fractions or compounds were added to the assay plate in a 1 : 100 dilution. Assay solutions of NEs, fractions and compounds were prepared in 96 well plates, stored at −20 °C and thawed before the experiment at room temperature or warmer with or without shaking/vortexing to obtain a clear assay solution. NEs were tested at 25 μg mL^−1^ and purified compounds in a dose–response curve of 6 concentrations with 100 μM being the top concentration and a 1 : 3 dilution step between each testing concentration. Since fractions were not weighed individually, the injection mass was used to calculate an average mass per fraction and thus a nominal mass concentration. Fractions were tested at nominal 10 μg mL^−1^.

As described in Nitschke *et al.*^11^ and Mottet *et al.*,^41^*D. discoideum* Ax2(ka) expressing mCherry at the act5 locus^42^ was infected with *M. marinum* M strain expressing the lux operon (luxCDABE)^43,44^ by spinoculation.

Briefly, the day before the experiment *M. marinum* was cultivated in 7H9 broth (Becton Dickinson, Difco Middlebrook 7H9) supplemented with 10% OADC (Becton Dickinson) and 0.05% tyloxapol (Sigma Aldrich) and 50 μg mL^−1^ kanamycin at 32 °C overnight with continuous shaking. Additionally, the day before the experiment 10^7^ amoebae were plated in HL5-C in a 10 cm Petri dish (Falcon). On the day of the experiment, a volume of the *M. marinum* culture corresponding to a multiplicity of infection of 25 with respect to the number of amoeba in the Petri dish was taken and added to the amoeba, subsequently the Petri dishes were centrifuged twice at 500×*g*, as described in Mottet *et al.*^41^ To remove extracellular bacteria, dishes were washed with fresh HL5-C and cells were resuspended in HL5-C with 5 U mL^−1^ penicillin and 5 μg mL^−1^ of streptomycin (Gibco) to inhibit extracellular growth of bacteria during the course of the experiment.

For testing fractions and purified compounds, 20 μL of infected cell suspension was plated into each well of a 384-well plate (Interchim FP-BA8240) to an effective cell number of 10^4^ cells per well. Fractions or compounds including a vehicle control (0.3% DMSO final concentration) and a positive control (rifabutin, 10 μM final concentration) were added using an electronic multipipette (Sartorius). Subsequently, the well plates were sealed with a gas impermeable membrane (H769.1, Carl Roth), briefly centrifuged and intracellular bacterial growth was monitored using an Agilent BioTek H1 plate reader, an Agilent BioTek BioStack plate stacker, by recording luminescence over 72 hours at 25 °C with readings taken every hour. Fluorescence was also recorded to monitor amoeba growth.

For testing bacteria in broth, the pre-culture was diluted to a bacterial density of 3.75 × 10^5^ bacteria per mL in 7H9 medium. Plating bacteria and compounds or fractions was performed analogously to the infection assay described above. Bacteria growth was monitored with the Agilent BioTek H1 plate reader by recording luminescence at 32 °C.

For both assays, growth curves were obtained by measuring the luminescence and fluorescence as a proxy for bacterial growth and host growth, respectively, for 72 hours with time-points taken every hour. The “normalized residual growth” was computed by calculating the area under the curve (AUC, trapezoid method) and normalization to the vehicle control (0.3% DMSO, set at 1) and a baseline curve (set at 0). The baseline curve was calculated by taking the median of the first measurement of all wells in a plate and extrapolating it over the full time course. The threshold for hit detection was arbitrarily fixed at a cut-off of normalized residual growth ≤0.5. Normalized values were averaged over technical and biological replicates (all experiments on isolated compounds have at least *n* = 3 and *N* = 3, whereas the primary extract screening and micro-fractions testing had values of *n* = 1 and *N* = 3).

This procedure was also applied to screening NEs, with slight modifications. The day before the experiment we pre-plated 10 μL HL5-C using a dispenser (Thermo Multidrop), subsequently we pre-plated 2.2 μL of dissolved NEs from 96-well plates into quadrants 1, 2 and 3 of 384-well plates, whereas quadrant 4 was used for positive and vehicle controls. Pre-plating of NEs was performed using a liquid handler (Agilent Bravo). In total, 24 NE plates were distributed in triplicates into eight 384-well plates, amounting to 24 assay plates. The prepared plates were sealed and stored at 4 °C overnight. On the day of the experiment, nine 10 cm Petri dishes were infected (as described before) and grouped into three pools. The cell suspension was adjusted to 10^6^ cells per mL and 10 μL were plated into the 24 assay plates, resulting in 10^4^ cells per well, as used for conventional infection experiments. Plates were sealed and placed in the plate stacker that supplies the plate reader. The same procedure was used to screen the same NEs on Mm in broth. For both assays, the first timepoints and a timepoint after 72 hours was recorded. Subsequently, we normalized the end point, first with the median of the full assay plate at the first time point, and second with the endpoint of the vehicle controls in the respective assay plate.

For IC_50_ estimation, we used a rudimentary approach of interpolating the sample concentration between the two normalized residual growth values which were closest to a value of 0.5.

### HRMS data analysis and generation of a feature-based molecular network (FBMN)

The data used to generate the FBMN comes from the untargeted metabolite profiling analyses carried out in a previously described study on 1600 NEs.^3^ The subset of UHPLC-HRMS/MS data from the 14 extracts according to Table 1 was used to generate an FBMN limited to stilbene-containing extracts. The HRMS/MS data from the entire set of NEs is available on the MassIVE repository under accession number MSV000087728. ThermoRawFileParser (v.1.4.4) was used to convert raw MS files into mzXML formats.^45^ The converted files were treated by MZMine software v.4.0.3.^46^ In positive mode, MS1 and MS2 for each scan were detected at threshold of 1 × 10^6^ and 0, respectively. The ADAP module was used to build chromatograms by connecting data points from mass lists. The chromatograms were deconvoluted into individual peaks by application of the “wavelet” algorithm. In the presence of isotopic patterns, isotopes were grouped to the lowest *m*/*z* ion. The extracted ions were aligned in a table and represented as features that demonstrate *m*/*z*, RT and peak area. The parameters for each mentioned treatment were adjusted according to Rutz *et al.*^47^ FBMNs were built online through the GNPS platform^5^ and visualized by Cytoscape.^48^ The GNPS parameters were adjusted according to Houriet *et al.*^49^ The GNPS job-ID for the molecular network generated is the following: ID = 267d0e36a07e4ff29ffd49a59455a09b.

### Structure and MF annotations using SIRIUS

Annotations were generated using SIRIUS 5.8.6 (ref. 15) for the determination of MF and structures, with the latter being generated through the integrated CSI.FingerID module.^16^ For the MF determination by SIRIUS, the following parameters were set: instrument: Orbitrap; filter by isotope pattern: yes; MS2 mass accuracy: 5 ppm; MS/MS isotope scorer: IGNORE; candidatures stored: 10; min candidates per ion stored: 1; no DB formulas selected; all possible ionizations selected; tree timeout: 0, compound timeout: 0; use heuristic above *m*/*z*: 300; use heuristic only above *m*/*z*: 650. Elements allowed in the MF were auto detected. For CSI:FingerID structural annotations, all possible fallback adducts and structure DB were selected; the score threshold and tag lipids parameters were also checked. CANOPUS^17,18,50^ was also used for compound class annotations (parameter-free).

### Electronic circular dichroism (ECD) and TD-DFT calculations

The absolute configuration assigned for all compounds was based on a comparison between the calculated and experimental ECD (see ESI data†). The calculations were based on the relative configuration determined through NMR 2D ROESY experiments. The structures were used to find the conformers through a random rotor search algorithm (number of conformers, 100) employing the MMFF94s force field in Avogadro v1.2.0.^51^ The conformers were further optimized using PM3 and B3LYP/6-31G (d,p) basis sets in Gaussian 16 software (©2015–2022, Gaussian Inc., Wallingford, CT, United States of America) with the SCRF model in methanol.^36,37^ Default convergence thresholds for self-consistent field (SCF), geometry and excited state optimizations (as Gaussian 16 built-in properties) were used. All optimized conformers were checked for imaginary frequencies. The conformers were subjected to ECD calculations using TD-DFT B3LYP/def2svp as a basis set and an SCRF model in methanol in Gaussian 16 software. The calculated ECD spectrum was generated in SpecDisv1.71 software (Berlin, Germany). The experimental and calculated spectra are available in the ESI.† The ECD calculations were performed on the HPC Baobab cluster at the University of Geneva. Graphs were made using GraphPad Prism (version 10.2.3) and chemical structures were drawn with ChemDraw (version 23.0.1), both licensed to OK by the University of Geneva.

(+)-Gnetin D (1) [*α*]^20^_D_ +15.7 (*c* 0.10, MeOH); UV (MeOH) *λ*_max_ (log *ε*) 226 (4.27), 287 (3.89), 310 (4.02), 328 (4.04), 347 (3.76) nm; ^1^H NMR (DMSO, 600 MHz) *δ* 9.54 (1H, s), 9.24 (2H, s), 9.05 (2H, s), 8.22 (1H, s), 7.41 (2H, d, *J* = 8.7 Hz), 7.02 (1H, d, *J* = 16.3 Hz), 6.90 (1H, d, *J* = 16.2 Hz), 6.85 (1H, d, *J* = 8.4 Hz), 6.76 (2H, d, *J* = 8.6 Hz), 6.66 (1H, s), 6.45 (1H, s), 6.33 (1H, d, *J* = 2.3 Hz), 6.15 (1H, dd, *J* = 8.4, 2.3 Hz), 6.05 (2H, d, *J* = 2.2 Hz), 6.04 (1H, d, *J* = 2.2 Hz), 5.54 (1H, d, *J* = 3.4 Hz), 4.20 (1H, d, *J* = 3.5 Hz); ^13^C NMR (DMSO, 151 MHz) *δ* 161.8, 158.2, 157.9, 157.2, 155.3, 154.6, 145.7, 139.4, 128.2, 127.9, 126.6, 125.6, 118.7, 115.6, 114.8, 107.1, 105.9, 105.5, 102.5, 100.7, 97.7, 87.7, 52.8 (NP-MRD ID: NP0061299 (https://np-mrd.org/natural_products/NP0061299)); HRESIMS *m*/*z* 471.1434 [M + H]^+^ (calcd for C_28_H_23_O_7_^+^ 471.1438 *Δ* = −0.85 ppm), MS/MS spectrum: CCMSLIB00012474988 (https://gnps.ucsd.edu/ProteoSAFe/gnpslibraryspectrum.jsp?SpectrumID=CCMSLIB00012474988), *m*/*z* 469.1291 [M − H]^−^ (calcd for C_28_H_21_O_7_^−^ 469.1293, *Δ* = −0.43 ppm).

(+)-Gnetupendin C (2) [*α*]^20^_D_ +69.6 (*c* 0.16, MeOH); UV (MeOH) *λ*_max_ (log *ε*) 226 (4.57), 287 (4.19), 310 (4.32), 328 (4.34), 347 (4.06) nm; ^1^H NMR (DMSO, 600 MHz) *δ* 9.54 (1H, s), 9.37 (1H, s), 9.24 (1H, s), 8.97 (1H, s), 8.71 (2H, s), 7.41 (2H, d, *J* = 8.7 Hz), 7.03 (1H, d, *J* = 16.3 Hz), 6.91 (1H, d, *J* = 16.2 Hz), 6.79–6.73 (3H, dd, *J* = 11.2, 8.5 Hz), 6.70 (1H, s), 6.49 (1H, d, *J* = 1.3 Hz), 6.16 (1H, d, *J* = 2.3 Hz), 5.94 (1H, dd, *J* = 8.3, 2.3 Hz), 5.84 (1H, d, *J* = 7.7 Hz), 5.78 (1H, t, *J* = 2.2 Hz), 5.63 (2H, d, *J* = 2.2 Hz), 4.45 (1H, d, *J* = 7.7 Hz); ^13^C NMR (DMSO, 151 MHz) *δ* 161.3, 157.2, 157.1, 156.9, 154.4, 154.2, 141.6, 138.9, 128.2, 127.9, 127.9, 127.4, 125.7, 116.8, 115.5, 114.5, 107.3, 106.8, 105.4, 101.6, 100.5, 98.1, 84.8, 48.6 (NP-MRD ID: NP0332862 (https://np-mrd.org/natural_products/NP0332862)); HRESIMS *m*/*z* 471.1433 [M + H]^+^ (calcd for C_28_H_23_O_7_^+^ 471.1438, *Δ* = −1.06 ppm), MS/MS spectrum: CCMSLIB00012474991 (https://gnps.ucsd.edu/ProteoSAFe/gnpslibraryspectrum.jsp?SpectrumID=CCMSLIB00012474991), *m*/*z* 469.1289 [M − H]^−^ (calcd for C_28_H_21_O_7_^−^ 469.1293, *Δ* = −0.85 ppm).

Gnetin C (3) [*α*]^20^_D_ −4.61 (*c* 0.09, MeOH); UV (MeOH) *λ*_max_ (log *ε*) 226 (4.23), 287 (3.74), 310 (3.86), 328 (3.88), 347 (3.61) nm; ^1^H NMR (DMSO, 600 MHz) *δ* 9.48 (1H, s), 9.31 (1H, s), 9.11 (2H, s), 8.22 (1H, s), 7.41 (2H, d, *J* = 8.6 Hz), 7.11 (2H, d, *J* = 8.6 Hz), 7.02 (1H, d, *J* = 16.3 Hz), 6.91 (1H, d, *J* = 16.3 Hz), 6.76 (4H, t, *J* = 8.3 Hz), 6.65 (1H, s), 6.48 (1H, s), 6.05 (1H, s), 5.99 (2H, d, *J* = 2.2 Hz), 5.31 (1H, d, *J* = 4.5 Hz), 4.23 (1H, d, *J* = 4.5 Hz); ^13^C NMR (DMSO, 151 MHz) *δ* 161.5, 158.4, 157.3, 157.2, 154.6, 145.0, 139.6, 132.2, 128.1, 128.1, 127.9, 126.8, 125.5, 115.5, 115.3, 114.0, 107.2, 105.3, 100.9, 97.6, 92.0, 54.3 (NP-MRD ID: NP0061298 (https://www.npmrd.org/natural_products/NP0061298)); HRESIMS *m*/*z* 455.1487 [M + H]^+^ (calcd for C_28_H_23_O_6_^+^ 455.1489, *Δ* = −0.44 ppm), MS/MS spectrum: CCMSLIB00012475006 (https://gnps.ucsd.edu/ProteoSAFe/gnpslibraryspectrum.jsp?SpectrumID=CCMSLIB00012475006), *m*/*z* 453.1338 [M − H]^−^ (calcd for C_28_H_21_O_6_^−^ 453.1344, *Δ* = −1.32 ppm).

(−)-Gnetoline A (4) [*α*]^20^_D_ +19.7 (*c* 0.17, MeOH); UV (MeOH) *λ*_max_ (log *ε*) 226 (4.47), 287 (3.94), 310 (4.02), 328 (4.05), 347 (3.80) nm; ^1^H NMR (DMSO, 600 MHz) *δ* 9.55 (2H, s), 9.40 (1H, s), 9.33 (1H, s), 9.18 (1H, s), 8.95 (1H, s), 8.72 (2H, s), 7.41 (2H, d, *J* = 8.9 Hz), 7.16 (2H, d, *J* = 8.7 Hz), 7.03 (1H, d, *J* = 15.5 Hz), 6.92 (1H, d, *J* = 16.3 Hz), 6.78–6.73 (5H, m), 6.68 (1H, s), 6.50 (2H, s), 6.19 (1H, s), 6.15 (1H, d, *J* = 1.4 Hz), 5.93 (1H, dd, *J* = 8.4, 2.3 Hz), 5.82 (1H, d, *J* = 7.9 Hz), 5.78 (1H, t, *J* = 2.2 Hz), 5.63 (2H, d, *J* = 2.2 Hz), 5.40 (1H, d, *J* = 3.9 Hz), 4.42 (1H, d, *J* = 7.8 Hz), 4.35 (1H, d, *J* = 3.8 Hz); ^13^C NMR (DMSO, 151 MHz) *δ* 161.53, 160.91, 157.26, 157.21, 157.06, 156.85, 154.61, 154.31, 154.20, 144.46, 141.61, 139.74, 132.26, 128.17, 128.09, 127.90, 127.35, 126.72, 125.50, 115.80, 115.53, 115.31, 114.55, 113.90, 107.27, 106.84, 105.56, 101.59, 100.53, 99.72, 97.71, 91.94, 84.98, 54.23, 48.52 (NP-MRD ID: NP0332863 (https://np-mrd.org/natural_products/NP0332863)); HRESIMS *m*/*z* 697.2068 [M + H]^+^ (calcd for C_42_H_33_O_10_^+^ 697.2068, *Δ* = 0 ppm), MS/MS spectrum: CCMSLIB00012475011 (https://gnps.ucsd.edu/ProteoSAFe/gnpslibraryspectrum.jsp?SpectrumID=CCMSLIB00012475011), *m*/*z* 695.1917 [M − H]^−^ (calcd for C_42_H_31_O_10_^−^ 695.1923, *Δ* = −0.86 ppm).

(−)-Latifolol (5) [*α*]^20^_D_ −12.4 (*c* 0.12, MeOH); UV (MeOH) *λ*_max_ (log *ε*) 226 (4.43), 287 (3.93), 310 (4.04), 328 (4.07), 347 (3.80) nm; ^1^H NMR (DMSO, 600 MHz) *δ* 9.57 (1H, s), 9.49 (2H, s), 9.40 (1H, s), 9.27 (1H, s), 9.16 (1H, s), 9.06 (2H, s), 7.42 (2H, d, *J* = 8.6 Hz), 7.16 (2H, d, *J* = 8.1 Hz), 7.04 (1H, d, *J* = 16.2 Hz), 6.92 (1H, d, *J* = 16.2 Hz), 6.87 (1H, d, *J* = 8.3 Hz), 6.77 (4H, d, *J* = 8.2 Hz), 6.68 (1H, s), 6.52 (1H, s), 6.31 (1H, m), 6.17 (2H, m), 6.11 (1H, s), 6.03 (3H, d, *J* = 1.1 Hz), 5.54 (1H, d, *J* = 4.4 Hz), 5.39 (1H, d, *J* = 4.0 Hz), 4.34 (1H, d, *J* = 4.0 Hz), 4.23 (1H, d, *J* = 4.5 Hz); ^13^C NMR (DMSO, 151 MHz) *δ* 161.5, 161.3, 158.1, 158.0, 157.3, 157.3, 155.6, 154.6, 154.5, 145.6, 144.8, 139.8, 132.2, 128.2, 128.2, 127.9, 127.1, 126.8, 125.5, 118.3, 115.6, 115.3, 113.9, 113.8, 107.3, 106.9, 106.1, 105.6, 102.5, 100.7, 99.5, 97.8, 92.0, 87.9, 54.3, 52.7, 48.6 (NP-MRD ID: NP0028328 (https://np-mrd.org/natural_products/NP0028328)); HRESIMS *m*/*z* 697.2069 [M + H]^+^ (calcd for C_42_H_33_O_10_^+^ 697.2068, *Δ* = 0.14 ppm), MS/MS spectrum: CCMSLIB00012475005 (https://gnps.ucsd.edu/ProteoSAFe/gnpslibraryspectrum.jsp?SpectrumID=CCMSLIB00012475005), *m*/*z* 695.1917 [M − H]^−^ (calcd for C_42_H_31_O_10_^−^ 695.1923, *Δ* = −0.86 ppm).

(−)-Gnetin E (6) [*α*]^20^_D_ −2.06 (*c* 0.13, MeOH); UV (MeOH) *λ*_max_ (log *ε*) 226 (4.45), 287 (3.91), 310 (4.03), 328 (4.04), 347 (3.78) nm; ^1^H NMR (600 MHz, DMSO) *δ* 9.56 (1H, s), 9.49 (2H, s), 9.40 (1H, s), 9.23 (1H, s), 9.10 (2H, s), 7.41 (2H, d, *J* = 8.7 Hz), 7.15 (2H, d, *J* = 8.6 Hz), 7.10 (2H, d, *J* = 8.5 Hz), 7.03 (1H, d, *J* = 16.3 Hz), 6.91 (1H, d, *J* = 16.3 Hz), 6.75 (6H, t, *J* = 8.7 Hz), 6.68 (1H, s), 6.50 (1H, s), 6.16 (1H, s), 6.13 (1H, s), 6.03 (1H, t, *J* = 2.2 Hz), 5.97 (2H, d, *J* = 2.2 Hz), 5.40 (1H, d, *J* = 4.0 Hz), 5.27 (1H, d, *J* = 5.7 Hz), 4.34 (1H, d, *J* = 3.9 Hz), 4.22 (1H, d, *J* = 5.7 Hz); ^13^C NMR (151 MHz, DMSO) *δ* 161.5, 161.0, 158.3, 157.3, 157.2, 154.6, 154.5, 145.1, 144.7, 139.7, 132.2, 131.7, 128.2, 128.1, 127.9, 127.2, 126.8, 125.5, 115.5, 115.3, 115.3, 113.9, 113.1, 107.3, 107.1, 105.4, 100.9, 99.4, 97.8, 92.4, 91.9, 54.3, 54.2 (NP-MRD ID: NP0332864 (https://www.npmrd.org/natural_products/NP0332864)); HRESIMS *m*/*z* 681.2120 [M + H]^+^ (calcd for C_42_H_33_O_9_^+^ 681.2119, *Δ* = 0.15 ppm), MS/MS spectrum: CCMSLIB00012474987 (https://gnps.ucsd.edu/ProteoSAFe/gnpslibraryspectrum.jsp?SpectrumID=CCMSLIB00012474987), *m*/*z* 679.1970 [M − H]^−^ (calcd for C_42_H_31_O_9_^−^ 679.1974, *Δ* = −0.59 ppm).

Macrostachyol A (7) [*α*]^20^_D_ −3.87 (*c* 0.12, MeOH); UV (MeOH) *λ*_max_ (log *ε*) 226 (4.59), 287 (4.01), 310 (4.07), 328 (4.10), 347 (3.84) nm; ^1^H NMR (DMSO, 600 MHz) *δ* 9.56 (2H, s), 9.49 (2H, s), 9.40 (1H, s), 9.31 (1H, s), 9.26 (1H, s), 9.16 (1H, s), 9.04 (2H, s), 7.41 (2H, d, *J* = 8.7 Hz), 7.16 (2H, d, *J* = 8.7 Hz), 7.13 (2H, d, *J* = 8.6 Hz), 7.03 (1H, d, *J* = 16.3 Hz), 6.91 (1H, d, *J* = 16.2 Hz), 6.85 (1H, d, *J* = 8.4 Hz), 6.80–6.72 (6H, m), 6.68 (1H, m), 6.50 (1H, m), 6.31 (1H, d, *J* = 2.3 Hz), 6.16 (3H, ddd, *J* = 8.0, 6.5, 1.8 Hz), 6.10 (2H, dd, *J* = 11.9, 1.2 Hz), 6.01 (3H, s), 5.51 (1H, d, *J* = 4.6 Hz), 5.41 (1H, d, *J* = 4.0 Hz), 5.35 (1H, d, *J* = 5.1 Hz), 4.35 (1H, d, *J* = 4.0 Hz), 4.32 (1H, d, *J* = 5.1 Hz), 4.23 (1H, d, *J* = 4.7 Hz); ^13^C NMR (DMSO, 151 MHz) *δ* 161.5, 161.1, 161.0, 158.1, 158.0, 157.3, 157.2, 155.6, 154.6, 154.5, 154.5, 145.5, 145.2, 144.5, 139.8, 132.2, 131.8, 128.2, 128.1, 127.9, 127.2, 127.1, 126.8, 125.5, 118.2, 115.5, 115.3, 115.3, 113.9, 113.7, 113.0, 107.3, 107.2, 106.9, 106.0, 105.6, 102.5, 100.7, 99.7, 99.6, 97.8, 92.3, 91.9, 87.8, 54.2, 52.6 (NP-MRD ID: NP0332865 (https://np-mrd.org/natural_products/NP0332865)); HRESIMS *m*/*z* 923.2700 [M + H]^+^ (calcd for C_56_H_43_O_13_^+^ 923.2698, *Δ* = 0.22 ppm), MS/MS spectrum: CCMSLIB00012475007 (https://gnps.ucsd.edu/ProteoSAFe/gnpslibraryspectrum.jsp?SpectrumID=CCMSLIB00012475007), *m*/*z* 921.2527 [M − H]^−^ (calcd for C_56_H_41_O_13_^−^ 921.2553, *Δ* = −2.82 ppm).

Gnemonol B (8) [*α*]^20^_D_ −6.18 (*c* 0.10, MeOH); UV (MeOH) *λ*_max_ (log *ε*) 226 (4.58), 287 (4.04), 310 (4.09), 328 (4.10), 347 (3.90) nm; ^1^H NMR (DMSO, 600 MHz) *δ* 9.56 (2H, s), 9.48 (2H, s), 9.39 (1H, s), 9.31 (1H, s), 9.23 (1H, s), 9.09 (2H, s), 7.41 (2H, d, *J* = 8.7 Hz), 7.16–7.12 (4H, dd, *J* = 11.8, 8.7 Hz), 7.09 (2H, d, *J* = 8.6 Hz), 7.03 (1H, d, *J* = 16.3 Hz), 6.91 (1H, d, *J* = 16.3 Hz), 6.77–6.74 (8H, dd, *J* = 8.7, 2.8 Hz), 6.67 (1H, d, *J* = 1.2 Hz), 6.50 (1H, d, *J* = 1.3 Hz), 6.19–6.10 (4H, m), 6.02 (2H, t, *J* = 2.1 Hz), 5.96 (1H, d, *J* = 2.2 Hz), 5.41 (1H, d, *J* = 4.1 Hz), 5.37 (1H, d, *J* = 5.0 Hz), 5.25 (1H, d, *J* = 5.9 Hz), 4.33 (2H, dd, *J* = 9.5, 4.5 Hz), 4.22 (1H, d, *J* = 5.9 Hz); ^13^C NMR (DMSO, 151 MHz) *δ* 161.5, 161.0, 160.9, 158.3, 158.1, 157.3, 157.2, 154.6, 154.5, 154.5, 145.2, 144.9, 139.8, 132.2, 131.8, 131.6, 128.2, 128.1, 127.9, 127.3, 127.1, 126.8, 125.5, 115.5, 115.3, 115.3, 113.9, 113.1, 113.0, 107.3, 107.2, 107.2, 105.4, 100.8, 99.6, 97.7, 92.4, 92.2, 91.9, 54.4, 54.2, 54.2 (NP-MRD ID: NP0140175 (https://www.npmrd.org/natural_products/NP0140175)); HRESIMS *m*/*z* 907.2742 [M + H]^+^ (calcd for C_56_H_43_O_12_^+^ 907.2749, *Δ* = −0.77 ppm), MS/MS spectrum: CCMSLIB00012475004 (https://gnps.ucsd.edu/ProteoSAFe/gnpslibraryspectrum.jsp?SpectrumID=CCMSLIB00012475004), *m*/*z* 905.2579 [M − H]^−^ (calcd for C_56_H_41_O_12_^−^ 905.2604, *Δ* = −2.76 ppm).

(−)-Gnemontanin G (9) [*α*]^20^_D_ −16.8 (*c* 0.06, MeOH); UV (MeOH) *λ*_max_ (log *ε*) 226 (4.09), 287 (3.29), 310 (2.84), 328 (2.94), 347 (2.51) nm; ^1^H NMR (DMSO, 600 MHz) *δ* 9.44 (1H, s), 9.19 (1H, s), 8.97 (2H, s), 8.85 (1H, s), 8.62 (1H, s), 7.10 (2H, d, *J* = 8.6 Hz), 6.72 (2H, d, *J* = 8.6 Hz), 6.57 (1H, d, *J* = 8.5 Hz), 6.22 (1H, dd, *J* = 8.4, 2.4 Hz), 6.17 (1H, d, *J* = 2.4 Hz), 6.04–6.01 (3H, m), 5.99 (1H, d, *J* = 2.1 Hz), 5.53 (1H, d, *J* = 2.1 Hz), 4.72 (1H, d, *J* = 8.4 Hz), 3.95 (1H, d, *J* = 7.1 Hz), 3.54–3.49 (1H, m), 3.27 (1H, t, *J* = 7.1 Hz); ^13^C NMR (DMSO, 151 MHz) *δ* 157.9, 157.1, 157.0, 156.5, 154.7, 154.2, 147.0, 144.8, 130.1, 129.7, 129.0, 121.3, 115.4, 114.8, 108.4, 106.2, 104.3, 103.4, 102.6, 101.8, 100.3, 77.3, 56.1, 55.6, 48.4, 47.0 (NP-MRD ID: NP0332866 (https://np-mrd.org/natural_products/NP0332866)); HRESIMS *m*/*z* 471.1432 [M + H]^+^ (calcd for C_28_H_23_O_7_^+^ 471.1438, *Δ* = −1.27 ppm), MS/MS spectrum: CCMSLIB00012474989 (https://gnps.ucsd.edu/ProteoSAFe/gnpslibraryspectrum.jsp?SpectrumID=CCMSLIB00012474989), *m*/*z* 469.1289 [M − H]^−^ (calcd for C_28_H_21_O_7_^−^ 469.1293, *Δ* = −0.85 ppm).

(−)-Gnetumontanin A (10) [*α*]^20^_D_ −13.4 (*c* 0.05, MeOH); UV (MeOH) *λ*_max_ (log *ε*) 226 (3.88), 287 (3.38), 305 (3.41), 333 (4.57), 342 (3.54) nm; ^1^H NMR (DMSO, 600 MHz) *δ* 9.59 (1H, d, *J* = 8.0 Hz), 9.54 (1H, d, *J* = 7.2 Hz), 9.40 (1H, s), 9.24 (1H, s), 9.21 (1H, d, *J* = 5.2 Hz), 9.04 (2H, s), 7.35 (1H, d, *J* = 8.5 Hz), 7.19 (1H, d, *J* = 16.4 Hz), 6.85 (1H, d, *J* = 16.4 Hz), 6.84 (1H, d, *J* = 8.3 Hz), 6.54 (1H, s), 6.44 (1H, d, *J* = 1.3 Hz), 6.32 (2H, t, *J* = 2.7 Hz), 6.25 (1H, dd, *J* = 8.5, 2.4 Hz), 6.14 (1H, dd, *J* = 8.3, 2.4 Hz), 6.04 (2H, d, *J* = 2.2 Hz), 6.02 (1H, t, *J* = 2.3 Hz), 5.53 (1H, d, *J* = 3.4 Hz), 4.18 (1H, d, *J* = 3.4 Hz); ^13^C NMR (DMSO, 151 MHz) *δ* 161.7, 158.1, 157.9, 156.1, 155.3, 154.6, 145.7, 140.1, 127.2, 126.5, 124.7, 123.3, 118.7, 115.3, 114.3, 107.2, 106.1, 105.9, 105.5, 102.6, 102.5, 100.7, 97.8, 87.6, 52.8 (NP-MRD ID: NP0332867 (https://np-mrd.org/natural_products/NP0332867)); HRESIMS *m*/*z* 487.1382 [M + H]^+^ (calcd for C_28_H_23_O_8_^+^ 487.1387, *Δ* = −1.03 ppm), MS/MS spectrum: CCMSLIB00012474986 (https://gnps.ucsd.edu/ProteoSAFe/gnpslibraryspectrum.jsp?SpectrumID=CCMSLIB00012474986), *m*/*z* 485.1240 [M − H]^−^ (calcd for C_28_H_21_O_8_^−^ 485.1242, *Δ* = −0.41 ppm).

(−)-Gnetuhainin M (11) [*α*]^20^_D_ −44.9 (*c* 0.12, MeOH); UV (MeOH) *λ*_max_ (log *ε*) 226 (4.63), 287 (4.11), 310 (4.16), 328 (4.19), 347 (3.90) nm; ^1^H NMR (DMSO, 600 MHz) *δ* 9.57 (1H, s), 9.53 (1H, s), 9.37 (2H, s), 9.17 (1H, s), 9.15 (2H, s), 9.07 (2H, s), 7.09 (2H, d, *J* = 8.7 Hz), 6.83 (1H, d, *J* = 16.3 Hz), 6.68 (2H, d, *J* = 8.6 Hz), 6.59 (1H, d, *J* = 2.2 Hz), 6.57 (1H, d, *J* = 16.3 Hz), 6.55 (1H, d, *J* = 8.4 Hz), 6.33–6.29 (3H, m), 6.23 (1H, d, *J* = 2.0 Hz), 6.10 (2H, d, *J* = 2.2 Hz), 6.09–6.05 (4H, m), 6.04 (1H, t, *J* = 2.1 Hz), 5.61 (1H, d, *J* = 6.6 Hz), 5.54 (1H, d, *J* = 2.6 Hz), 4.65 (1H, d, *J* = 6.6 Hz), 4.10 (1H, d, *J* = 2.6 Hz); ^13^C NMR (DMSO, 151 MHz) *δ* 160.8, 158.9, 158.6, 158.4, 158.1, 157.6, 157.3, 154.9, 154.8, 145.6, 145.5, 134.7, 128.8, 128.0, 127.7, 127.6, 125.8, 122.0, 118.9, 118.6, 115.5, 115.1, 112.8, 108.6, 105.9, 105.7, 105.6, 102.9, 102.4, 101.1, 100.7, 96.0, 88.2, 88.2, 53.9, 53.3 (NP-MRD ID: NP0332868 (https://np-mrd.org/natural_products/NP0332868)); HRESIMS *m*/*z* 713.2018 [M + H]^+^ (calcd for C_42_H_33_O_11_^+^ 713.2017, *Δ* = 0.14 ppm), MS/MS spectrum: CCMSLIB00012474996 (https://gnps.ucsd.edu/ProteoSAFe/gnpslibraryspectrum.jsp?SpectrumID=CCMSLIB00012474996), *m*/*z* 711.1870 [M − H]^−^ (calcd for C_42_H_31_O_11_^−^ 711.1872, *Δ* = −0.28 ppm).

## Data availability

All data relative to the above-mentioned collection of 1600 NEs was described in Allard *et al.* (2023)^3^ at https://doi.org/10.1093/gigascience/giac124. The data supporting this article have been included as part of the ESI† associated as a .pdf file. Supplementary SPARQL scripts are available under the respective links provided. The raw biological data presented is available as a ESI Table file (.csv). The raw NMR data of all isolated compounds presented in this study is available through the NP-MRD platform *via* the NP-MRD IDs provided for each compound in the experimental section. The raw HRMS/MS data of all isolated compounds presented in this study is accessible through the GNPS platform *via* the spectrum IDs provided for each compound in the experimental section.

## Author contributions

OK: conceptualization, data curation, investigation, methodology, software, validation, visualization, writing – original draft, writing – review and editing. LQG: data curation, investigation, methodology, software, visualization, writing – original draft, writing – review and editing. JN: conceptualization, data curation, investigation, validation, visualization, writing – original draft. LFN: conceptualization, data curation, methodology, software, supervision, writing – review and editing. FB: data curation, investigation, methodology, software. LM: data curation, investigation, writing – review and editing. NH: data curation, methodology, supervision, writing – review and editing. FM: data curation, investigation, methodology, software. BD: resources. AG: resources, writing – review and editing. EFQ: investigation, methodology, supervision, writing – review and editing. MP: data curation, investigation, methodology, software. TS: conceptualization, funding acquisition, project administration, supervision, writing – review and editing. J-LW: conceptualization, funding acquisition, project administration, supervision, writing – original draft, writing – review and editing.

## Conflicts of interest

There are no conflicts to declare.

## Supplementary Material

RA-015-D4RA08421G-s001

RA-015-D4RA08421G-s002

RA-015-D4RA08421G-s003

RA-015-D4RA08421G-s004

RA-015-D4RA08421G-s005

RA-015-D4RA08421G-s006

RA-015-D4RA08421G-s007

RA-015-D4RA08421G-s008
