# Supplementary material for: Prioritization of novel anti-infective stilbene derivatives by combining metabolomic data organization and a stringent 3R-infection model in a knowledge graph
Source: RSC Adv. 2025 Apr 23;15(17):13010–30. doi: 10.1039/d4ra08421g (PMC12015462; doi:10.1039/d4ra08421g)
Supplement: RA-015-D4RA08421G-s008 [file RA-015-D4RA08421G-s008.pdf]

Supplementary Information (SI) for RSC Advances.
This journal is © The Royal Society of Chemistry 2025

Supplementary Material

Prioritization of Novel Anti-infective Stilbene derivatives by Combining Metabolomic Data Organization and a Stringent 3R-infection Model in a Knowledge Graph

Olivier Auguste Kirchhoffer[a,b], Luis Quirós-Guerrero[a,b], Jahn Nitschke[c], Louis-Félix Nothias[a,b,d], Frédéric Burdet[e], Laurence Marcourt[a,b], Nabil Hanna[c], Florence Mehl[e], Bruno David[f], Antonio Grondin[f], Emerson Ferreira Queiroz[a,b], Marco Pagni[e], Thierry Soldati[c] and Jean-Luc Wolfender[a,b*]

[a.] *Institute of Pharmaceutical Sciences of Western Switzerland, University of Geneva, CMU, 1211 Geneva, Switzerland.*
[b.] *School of Pharmaceutical Sciences, University of Geneva, CMU, 1211 Geneva, Switzerland.*
[c.] *Department of Biochemistry, Faculty of Sciences, University of Geneva, Quai Ernest-Ansermet 30, 1205 Geneva, Switzerland.*
[d.] *Université Côte d'Azur, CNRS, ICN, France.*
[e.] *Vital-IT, SIB Swiss Institute of Bioinformatics, 1015 Lausanne, Switzerland.*
[f.] *Green Mission Department, Herbal Products Laboratory, Pierre Fabre Research Institute, Toulouse, France.*

Table of content

Supplementary Figures 6

Supplementary Figure 1 - Chromatographic separations of the ethyl acetate extract of S. brunoniana with compounds A-D labeled (corresponding to those in Figure 1 of the manuscript) as well as corresponding micro-fractions (in Figure 3)........ 6

Supplementary Figure 2 – Representation of the Knowledge Graph (KG) with a focus on a document generated from the MS/MS spectrum of Macrostachyol A (compound D). 7

Supplementary Figure 3 – Distribution of JSI indices resulting from a query using the spectrum of compound A in the extract of S. brunoniana as proxy. 8

Supplementary Figure 4 - Dose-response curves for (-)-Gnetuhainin M (11) and Rapamycin with mean values. In red: bacterial growth in infection; in green: amoeba growth in infection; Concentrations recorded (from left to right): 0.41 µM, 1.23 µM, 3.70 µM, 11.1 µM, 33.3 µM and 100 µM ((-)-Gnetuhainin M); 0.0045 µM, 0.0135 µM, 0.0405 µM, 0.1215 µM, 0.3646 µM and 1.094 µM (Rapamycin). 9

Supplementary Data: NMR and HRMS 10

Nuclear Magnetic Resonance (NMR) measurements 10

Electronic Circular Dichroism calculations (ECD) 11

1. Gnetin D: 13

S1.1. ECD spectra (experimental and calculated) of Gnetin D in MeOH 14

S1.2. HRESIMS+ spectrum of Gnetin D in MeOH. 14

S1.3. HRESIMS- spectrum of Gnetin D in MeOH. 14

S1.4. ^{1}H NMR spectrum of Gnetin D in DMSO-d_6 at 600 MHz. 15

S1.5. ^{13}C NMR spectrum of Gnetin D in DMSO-d_6 at 151 MHz. 16

S1.6. HSQC NMR spectrum of Gnetin D in DMSO-d_6. 17

S1.7. HMBC NMR spectrum of Gnetin D in DMSO-d_6. 17

S1.8. COSY NMR spectrum of Gnetin D in DMSO-d_6. 18

S1.9. ROESY NMR spectrum of Gnetin D in DMSO-d_6. 18

2. (+)-Gnetupendin C: 19

S2.1. ECD spectra (experimental and calculated) of (+)-Gnetupendin C in MeOH. 19

S2.2. HRESIMS+ spectrum of (+)-Gnetupendin C in MeOH. 20

S2.3. HRESIMS- spectrum of (+)-Gnetupendin C in MeOH. 20

S2.4 ^{1}H NMR spectrum of (+)-Gnetupendin C in DMSO-d_6 at 600 MHz. 21

S2.5. ^{13}C NMR spectrum of (+)-Gnetupendin C in DMSO-d_6 at 151 MHz. 22

S2.6. HSQC NMR spectrum of (+)-Gnetupendin C in DMSO-d_6. 23

S2.7. HMBC NMR spectrum of (+)-Gnetupendin C in DMSO-d_6. 23

S2.8. COSY NMR spectrum of (+)-Gnetupendin C in DMSO-d_6. 24

S2.9. ROESY NMR spectrum of (+)-Gnetupendin C in DMSO-d_6. 24

3. Gnetin C: 25
S3.1. HRESIMS+ spectrum of Gnetin C in MeOH. 25
S3.2. HRESIMS- spectrum of Gnetin C in MeOH. 26
S3.3. ^{1}H NMR spectrum of Gnetin C in DMSO-d_6 at 600 MHz. 26
S3.5. HSQC NMR spectrum of Gnetin C in DMSO-d_6. 27
S3.6. HMBC NMR spectrum of Gnetin C in DMSO-d_6. 28
S3.7. COSY NMR spectrum of Gnetin C in DMSO-d_6. 28
S3.8. ROESY NMR spectrum of Gnetin C in DMSO-d_6. 29
4. Gnetoline A: 30
S4.1. ECD spectra (experimental and calculated) of Gnetoline A in MeOH. 31
S4.2. HRESIMS+ spectrum of Gnetoline A in MeOH. 32
S4.3. HRESIMS- spectrum of Gnetoline A in MeOH. 32
S4.4. ^{1}H NMR spectrum of Gnetoline A in DMSO-d_6 at 600 MHz. 33
S4.5. ^{13}C NMR spectrum of Gnetoline A in DMSO-d_6 at 151 MHz. 33
S4.6. HSQC NMR spectrum of Gnetoline A in DMSO-d_6. 34
S4.7. HMBC NMR spectrum of Gnetoline A in DMSO-d_6. 35
S4.8. COSY NMR spectrum of Gnetoline A in DMSO-d_6. 36
S4.9. ROESY NMR spectrum of Gnetoline A in DMSO-d_6. 36
5. Latifolol: 37
S5.1. ECD spectra (experimental and calculated) of Latifolol in MeOH. 38
S5.2. HRESIMS+ spectrum of Latifolol in MeOH. 38
S5.3. HRESIMS- spectrum of Latifolol in MeOH. 39
S5.4. ^{1}H NMR spectrum of Latifolol in DMSO-d_6 at 600 MHz. 40
S5.5. ^{13}C NMR spectrum of Latifolol in DMSO-d_6 at 151 MHz. 40
S5.6. HSQC NMR spectrum of Latifolol in DMSO-d_6. 41
S5.7. HMBC NMR spectrum of Latifolol in DMSO-d_6. 42
S5.8. COSY NMR spectrum of Latifolol in DMSO-d_6. 43
S5.9. ROESY NMR spectrum of Latifolol in DMSO-d_6. 43
6. (-)-Gnetin E: 44
S6.1. ECD spectra (experimental and calculated) of (-)-Gnetin E in MeOH. 45
S6.2. HRESIMS+ spectrum of (-)-Gnetin E in MeOH. 45
S6.3. HRESIMS- spectrum of (-)-Gnetin E in MeOH. 46
S6.4. ^{1}H NMR spectrum of (-)-Gnetin E in DMSO-d_6 at 600 MHz. 47
S6.5. ^{13}C NMR spectrum of (-)-Gnetin E in DMSO-d_6 at 151 MHz. 47
S6.6. HSQC NMR spectrum of (-)-Gnetin E in DMSO-d_6. 48

S6.7. HMBC NMR spectrum of (-)-Gnetin E in DMSO-d_6. 49
S6.8. COSY NMR spectrum of (-)-Gnetin E in DMSO-d_6. 50
S6.9. ROESY NMR spectrum of (-)-Gnetin E in DMSO-d_6. 50
7. Macrostachyol A: 51
S7.1. HRESIMS+ spectrum of Macrostachyol A in MeOH. 52
S7.2. HRESIMS- spectrum of Macrostachyol A in MeOH. 52
S7.3. ^{1}H NMR spectrum of Macrostachyol A in DMSO-d_6 at 600 MHz. 53
S7.4. ^{13}C NMR spectrum of Macrostachyol A in DMSO-d_6 at 151 MHz. 53
S7.5. HSQC NMR spectrum of Macrostachyol A in DMSO-d_6. 54
S7.6. HMBC NMR spectrum of Macrostachyol A in DMSO-d_6. 54
S7.7. COSY NMR spectrum of Macrostachyol A in DMSO-d_6. 55
S7.8. ROESY NMR spectrum of Macrostachyol A in DMSO-d_6. 55
8. Gnemonol B: 56
S8.1. HRESIMS+ spectrum of Gnemonol B in MeOH. 57
S8.2. HRESIMS- spectrum of Gnemonol B in MeOH. 57
S8.3. ^{1}H NMR spectrum of Gnemonol B in DMSO-d_6 at 600 MHz. 58
S8.4. ^{13}C NMR spectrum of Gnemonol B in DMSO-d_6 at 151 MHz. 58
S8.5. HSQC NMR spectrum of Gnemonol B in DMSO-d_6. 59
S8.6. HMBC NMR spectrum of Gnemonol B in DMSO-d_6. 59
S8.7. COSY NMR spectrum of Gnemonol B in DMSO-d_6. 60
S8.9. ROESY NMR spectrum of Gnemonol B in DMSO-d_6 60
9. Gnemontanin G: 61
S9.1. HRESIMS+ spectrum of Gnemontanin G in MeOH. 61
S9.2. HRESIMS- spectrum of Gnemontanin G in MeOH. 62
S9.3. ^{1}H NMR spectrum of Gnemontanin G in DMSO-d_6 at 600 MHz. 62
S9.4. ^{13}C NMR spectrum of Gnemontanin G in DMSO-d_6 at 151 MHz. 63
S9.5. HSQC NMR spectrum of Gnemontanin G in DMSO-d_6. 63
S9.6. HMBC NMR spectrum of Gnemontanin G in DMSO-d_6. 64
S9.7. COSY NMR spectrum of Gnemontanin G in DMSO-d_6. 64
S9.8. ROESY NMR spectrum of Gnemontanin G in DMSO-d_6. 65
10. (-)-Gnetumontanin A: 66
S10.1. HRESIMS+ spectrum of (-)-Gnetumontanin A in MeOH. 66
S10.2. HRESIMS- spectrum of (-)-Gnetumontanin A in MeOH. 67
S10.3. ^{1}H NMR spectrum of (-)-Gnetumontanin A in DMSO-d_6 at 600 MHz. 67
S10.4. ^{13}C NMR spectrum of (-)-Gnetumontanin A in DMSO-d_6 at 151 MHz. 68

S10.6. HMBC NMR spectrum of (-)-Gnetumontanin A in DMSO-d_6. 69
S10.7. COSY NMR spectrum of (-)-Gnetumontanin A in DMSO-d_6. 69
S10.9. ROESY NMR spectrum of (-)-Gnetumontanin A in DMSO-d_6. 70
11. (-)-Gnetuhainin M: 71
S11.1. ECD spectra (experimental and calculated) of (-)-Gnetuhainin M in MeOH........... 72
S11.2. HRESIMS+ spectrum of (-)-Gnetuhainin M in MeOH. 73
S11.3. HRESIMS- spectrum of (-)-Gnetuhainin M in MeOH. 73
S11.4. ^{1}H NMR spectrum of (-)-Gnetuhainin M in DMSO-d_6 at 600 MHz. 73
S11.5. ^{13}C NMR spectrum of (-)-Gnetuhainin M in DMSO-d_6 at 151 MHz. 74
S11.6. HSQC NMR spectrum of (-)-Gnetuhainin M in DMSO-d_6. 74
S11.7. HMBC NMR spectrum of (-)-Gnetuhainin M in DMSO-d_6. 75
S11.8. COSY NMR spectrum of (-)-Gnetuhainin M in DMSO-d_6. 75
S11.9. ROESY NMR spectrum of (-)-Gnetuhainin M in DMSO-d_6. 76

Supplementary Figures

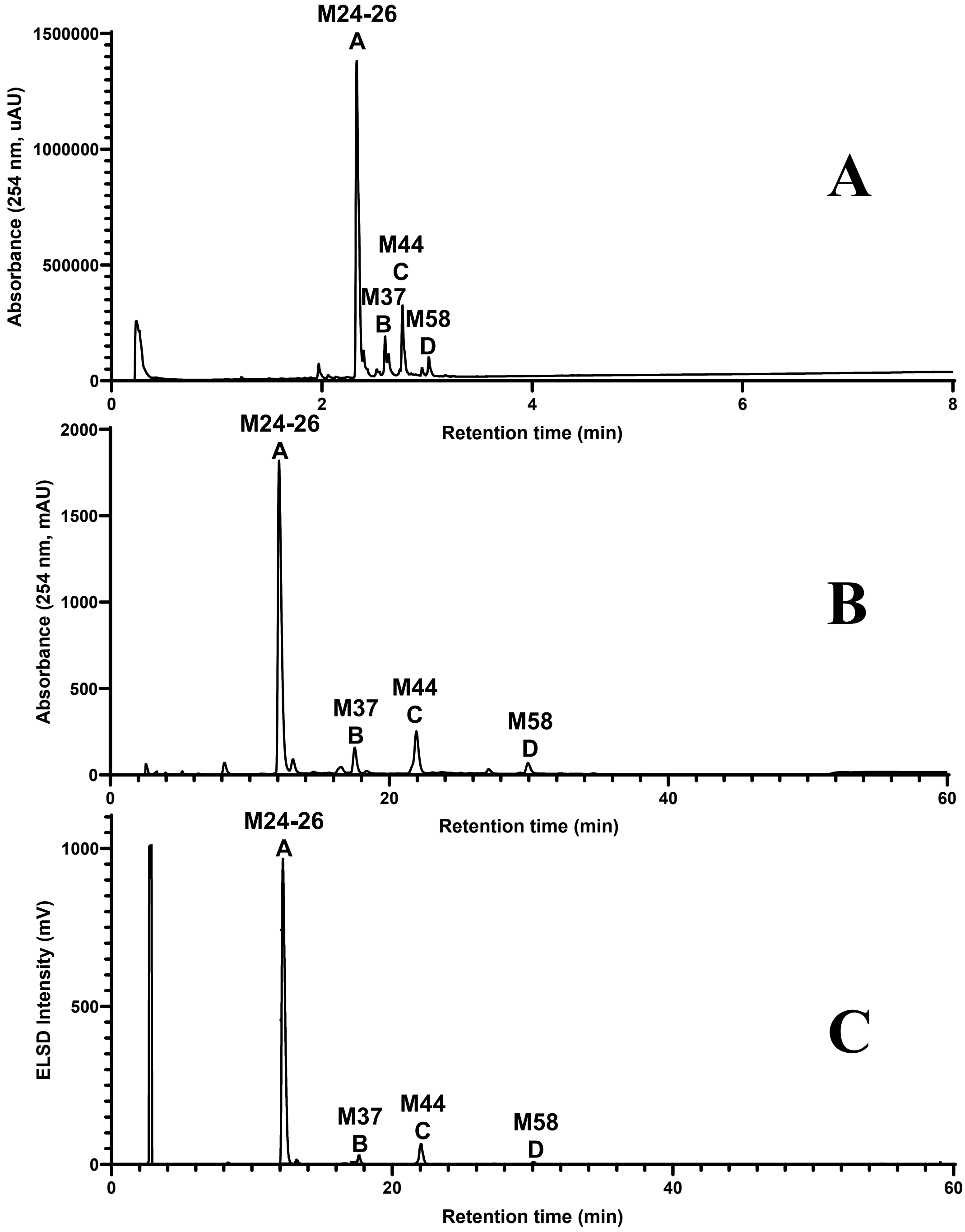

Supplementary Figure 1 - Chromatographic separations of the ethyl acetate extract of S. brunoniana with compounds A-D labeled (corresponding to those in Figure 1 of the manuscript) as well as corresponding micro-fractions (in Figure 3). A: UV chromatogram at 254 nm of the ethyl acetate extract of S. brunoniana using the standard gradient reported for the analysis of the 1600 plant set1. B: UV chromatogram at 254 nm of the ethyl acetate extract of S. brunoniana from newly extracted plant material using a gradient that has been optimized to improve the separation of different components. C: ELSD chromatogram of the ethyl acetate extract of S. brunoniana from newly extracted plant material.

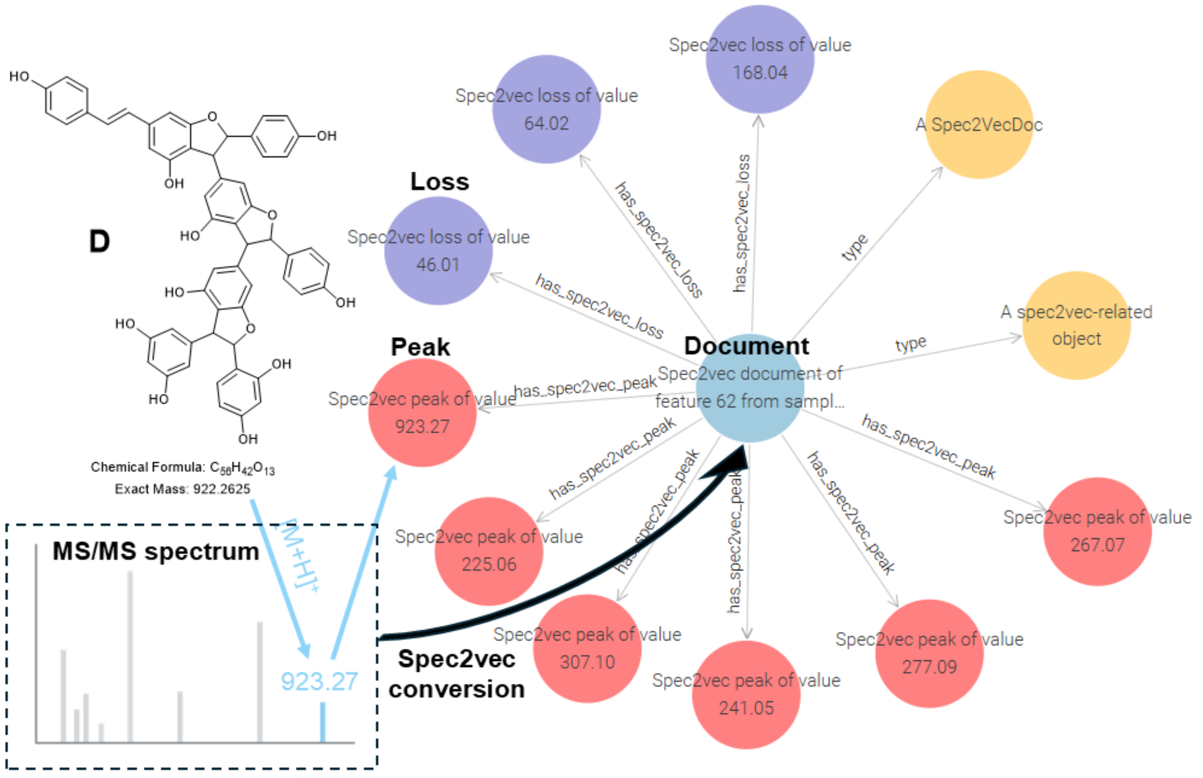

Supplementary Figure 2 – Representation of the Knowledge Graph (KG) with a focus on a document generated from the MS/MS spectrum of Macrostachyol A (compound D).

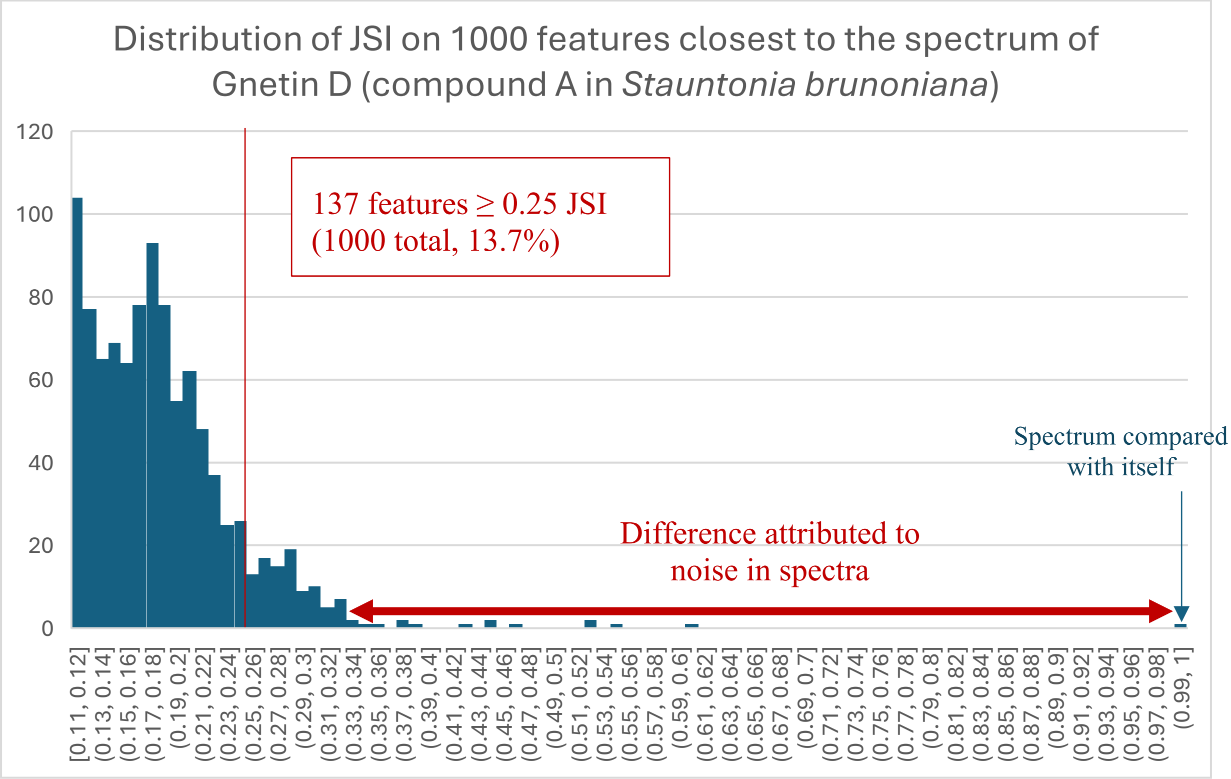

Supplementary Figure 3 – Distribution of JSI indices resulting from a query using the spectrum of compound A in the extract of S. brunoniana as proxy. The query provided the 1000 features with the highest corresponding JSI score. Among the 137 features having a JSI ≥ 0.25, 43 were annotated as "oligomeric stilbenes", 2 were annotated as "unknown" and 92 were unannotated.

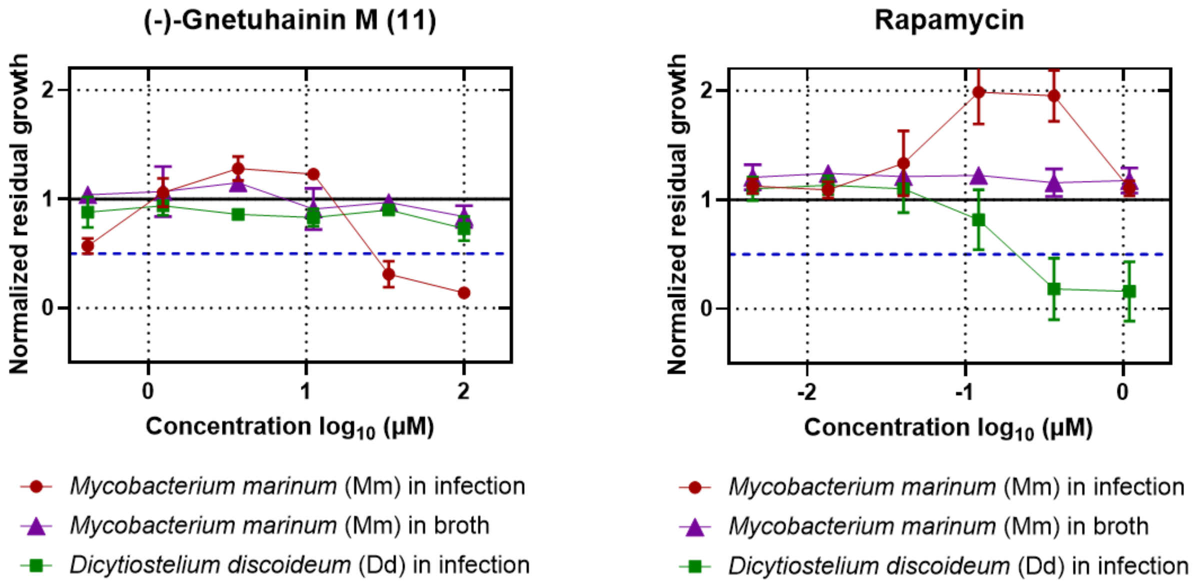

Supplementary Figure 4 - Dose-response curves for (-)-Gnetuhainin M (11) and Rapamycin with mean values. In red: bacterial growth in infection; in green: amoeba growth in infection; Concentrations recorded (from left to right): 0.41 µM, 1.23 µM, 3.70 µM, 11.1 µM, 33.3 µM and 100 µM ((-)-Gnetuhainin M); 0.0045 µM, 0.0135 µM, 0.0405 µM, 0.1215 µM, 0.3646 µM and 1.094 µM (Rapamycin).

Supplementary Data: NMR and HRMS

Nuclear Magnetic Resonance (NMR) measurements

All Nuclear Magnetic Resonance (NMR) measurements were recorded on a Bruker Avance Neo 600 MHz NMR spectrometer (Cryoprobe QCI 5-mm) equipped with a SampleJet automated sample changer (Bruker BioSpin, Rheinstetten, Germany). Chemical shifts are presented in parts per million (δ), referencing the residual DMSO-d_6 signal (δ_H 2.50; δ_C 39.5) as internal standards for ^{1}H and ^{13}C NMR, respectively, with coupling constants (*J*) reported in Hz. Additional 2D experiments (HSQC, HMBC, COSY and ROESY) as well as comparisons with literature were used when performing complete structural assignments.

UHPLC-DAD-HRMS/MS of extracts, fractions and pure compounds

Analyses were performed with a Waters Acquity UHPLC system coupled to a Corona Veo RS Charged Aerosol Detector (CAD, Thermo Scientific, Germany) and an Orbitrap Exploris 120 mass spectrometer (Thermo Scientific, Germany). The Orbitrap employed a heated electrospray ionization source (H-ESI) with the following parameters: spray voltage: +3.5 kV; ion transfer tube temperature: 320.00 °C; vaporizer temperature: 320.00 °C; S-lens RF: 45 (arb units); sheath gas flow rate: 35.00 (arb units); Sweep Gas (arb): 1 and auxiliary gas flow rate: 10.00 (arb. units). Control of the instruments was done using Thermo Scientific Xcalibur software v. 4.6.67.17. Full scans were acquired at a resolution of 30,000 fwhm (at m/ z 200) and MS2 scans at 15000 fwhm in the range of 100−1000 m/ z, with 1 microscan, time (ms): 200, an RF lens (%): 70; AGC target custom (Normalized AGC target (%): 300); maximum injection time (ms): 130; Microscans: 1; data type: profile; Use EASY-IC(TM): ON. The settings for dynamic exclusion mode were customized; Exclude after n times: 1; Exclusion duration (s): 5; Mass tolerance: ppm; low: 10, high: 10, Exclude isotopes: true. Appex detention: Desired Apex Window (%): 50. Isotope Exclusion: Assigned and unassigned with an exclusion window (m/z) for unassigned isotopes: 8. The Intensity threshold was set to 2.5E5 and a targeted mass exclusion list was used.

The centroid data-dependent MS2 (dd-MS2) scan acquisition events were performed in discovery mode, triggered by Apex detection with a trigger detection (%) of 300 with a maximum injection time of 120 ms, performing 1 microscan. The top 3 abundant precursors (charge states 1 and 2) within an isolation window of 1.2 m/z were considered for MS/MS analysis. For precursor fragmentation in the HCD mode, a normalized collision energy of 15, 30 and 45 % was used. Data was recorded in profile mode (Use EASY-IC(TM): ON).

The chromatographic separation was done on a Waters BEH C18 column (50 × 2.1 mm i.d., 1.7 μm, Waters, Milford, MA) using the following gradient (time (min), %B): 5%B from 0 to 0.5 min; from 5%B to 100%B between 0.5 and 7 min; 100%B from 7 to 8 min, from 100%B to 5%B from 8 to 8.10 min; 5%B from 8.10 to 10 min. The mobile phases were (A) water and (B) acetonitrile both containing 0.1% FA. The flow rate was set to 600 μL/min, the injection volume was 2 μL and the column was kept at 40 °C. The PDA detector was used from 210 to 400 nm with a resolution of 1.2 nm. The CAD detector was kept at 40 °C, with 5 bar N_2 and power function 1 for a data collection rate of 20 Hz.

All the raw NMR data has been submitted in the public repository of NP-MRD and will be publicly released upon publication of the article. Individual identifiers for each compound referring to this database were also provided in the present document.

Electronic Circular Dichroism calculations (ECD)

The absolute configuration assigned for all compounds was based on a comparison between the calculated and experimental ECD. The calculations were based on the relative configuration determined through NMR 2D ROESY experiments. The structures were used to find the conformers through a random rotor search algorithm (number of conformers, 100) employing the MMFF94s force field in Avogadro v1.2.0[2]. The conformers were further optimized using PM3 and B3LYP/6-31G (d,p) basis sets in Gaussian 16 software (© 2015-2022, Gaussian Inc., Wallingford, CT, United States of America) with the SCRF model in methanol[3,4]. All optimized conformers were checked for imaginary frequencies. The conformers were subjected to ECD calculations using TD-DFT B3LYP/def2svp as a basis set and an SCRFmodelinmethanolinGaussian16 software. The calculated ECD spectrum was generated in SpecDisv1.71 software (Berlin, Germany). The ECD calculations were performed on the HPC Baobab cluster at the University of Geneva.

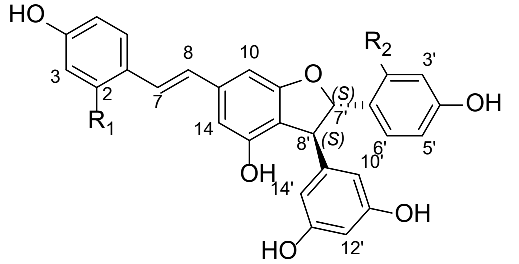

1. 7'S,8'S, R_1 = H, R_2 = OH, Gnetin D
2. 7'S,8'R, R_1 = H, R_2 = OH, (+)-Gnetupendin C
3. 7'S,8'S, R_1 = H, R_2 = H, Gnetin C
10. 7'S,8'S, R_1 = OH, R_2 = OH, (-)-Gnetumontanin A

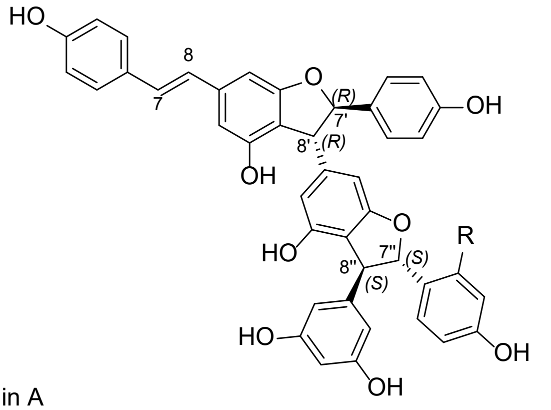

4. 7'R,8'R,7''R,8''S, R = OH
5. 7'R,8'R,7''S,8''S, R = OH, Latifolol
6. 7'R,8'R,7''S,8''S, R = H

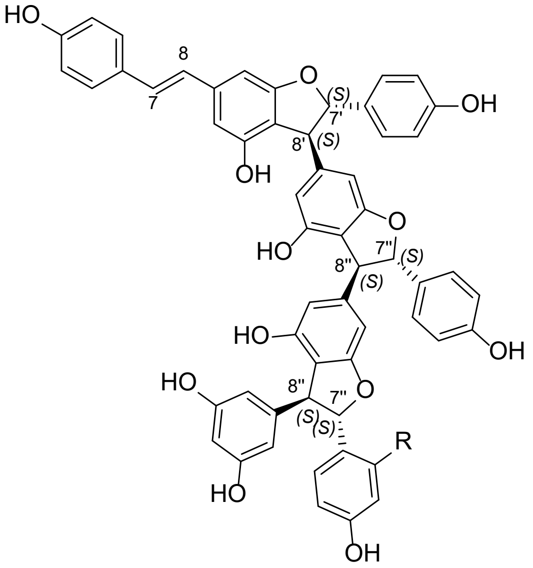

7. R = OH, Macrostachyol
8. R = H, Gnemonol B

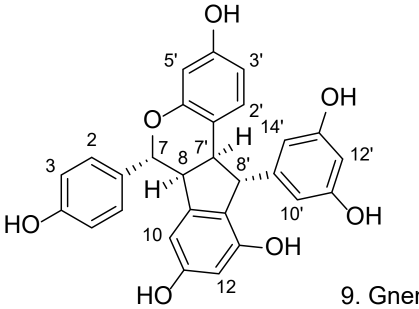

9. Gnemontanin G

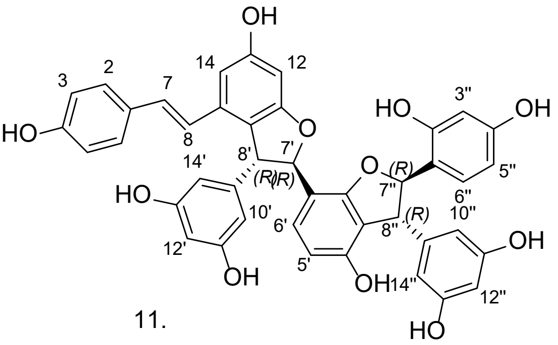

11.

1. Gnetin D:

Experimental:

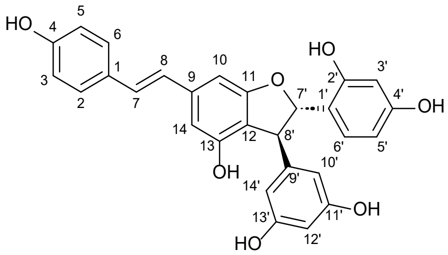

Gnetin D (1) $[\alpha]_D^{20}$ +15.7 (c 0.10, MeOH), Literature[5] : $[\alpha]_D^{20}$ +65.9 (c 0.13, MeOH); UV (MeOH) λ_{max} (log ε) 226 (4.27), 287 (3.89), 310 (4.02), 328 (4.04), 347 (3.76) nm;

^{1}H NMR (DMSO-d_6, 600 MHz) δ 9.54 (1H, s, 2'-OH), 9.24 (1H, s, 13-OH), 9.05 (2H, s, 11'-OH, 13'-OH), 7.42 (2H, d, J = 8.3 Hz, H-2, H-6), 7.02 (1H, d, J = 16.3 Hz, H-7), 6.90 (1H, d, J = 16.3 Hz, H-8), 6.85 (1H, d, J = 8.3 Hz, H-6'), 6.77 (3H, d, J = 8.3 Hz, H-3, H-5), 6.66 (1H, s, H-10), 6.45 (1H, s, H-14), 6.33 (1H, d, J = 2.3 Hz, H-3'), 6.15 (1H, dd, J = 8.3, 2.3 Hz, H-5'), 6.05 (2H, d, J = 2.3 Hz, H-10', H-14'), 6.04 (1H, t, J = 2.3 Hz, H-12'), 5.55 (1H, d, J = 3.4 Hz, H-7'), 4.21 (1H, d, J = 3.4 Hz, H-8'); ^{13}C NMR (DMSO-d_6, 151 MHz) δ 161.8 (C-11), 158.2 (C-11', C-13'), 157.9 (C-4'), 157.3 (C-4), 155.3 (C-2'), 154.6 (C-13), 145.7 (C-9'), 139.4 (C-9), 128.2 (C-1), 128.0 (CH-7), 127.9 (CH-2, CH-6), 126.6 (CH-6'), 125.6 (CH-8), 118.7 (C-1'), 115.6 (CH-3, CH-5), 114.8 (C-12), 107.1 (CH-14), 105.9 (CH-5'), 105.5 (CH-10', CH-14'), 102.5 (CH-3'), 100.7 (CH-12'), 97.7 (CH-10), 87.7 (CH-7'), 52.8 (CH-8'); ; (NP-MRD ID: NP0061299); HRESIMS m/z 471.1434 $[M+H]^+$ (calcd for $C_{28}H_{23}O_7^+$ 471.1438 Δ = -0.85 ppm), MS/MS spectrum: CCMSLIB00012474988, m/z 469.1291 $[M-H]^-$ (calcd for $C_{28}H_{21}O_7^-$ 469.1293, Δ = -0.43 ppm).

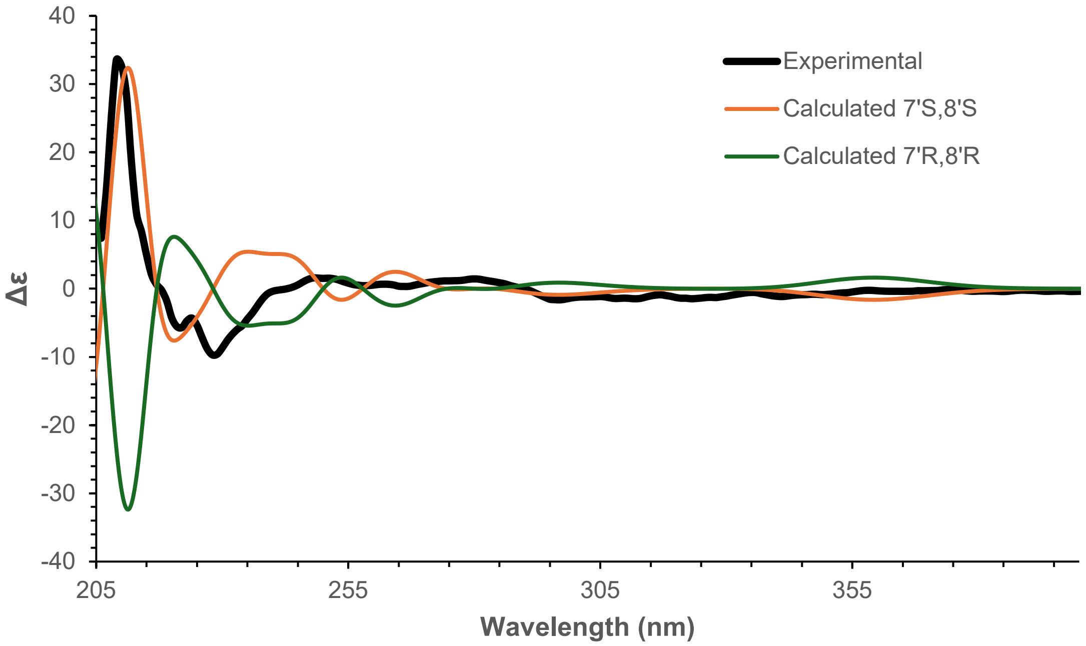

S1.1. ECD spectra (experimental and calculated) of Gnetin D in MeOH.

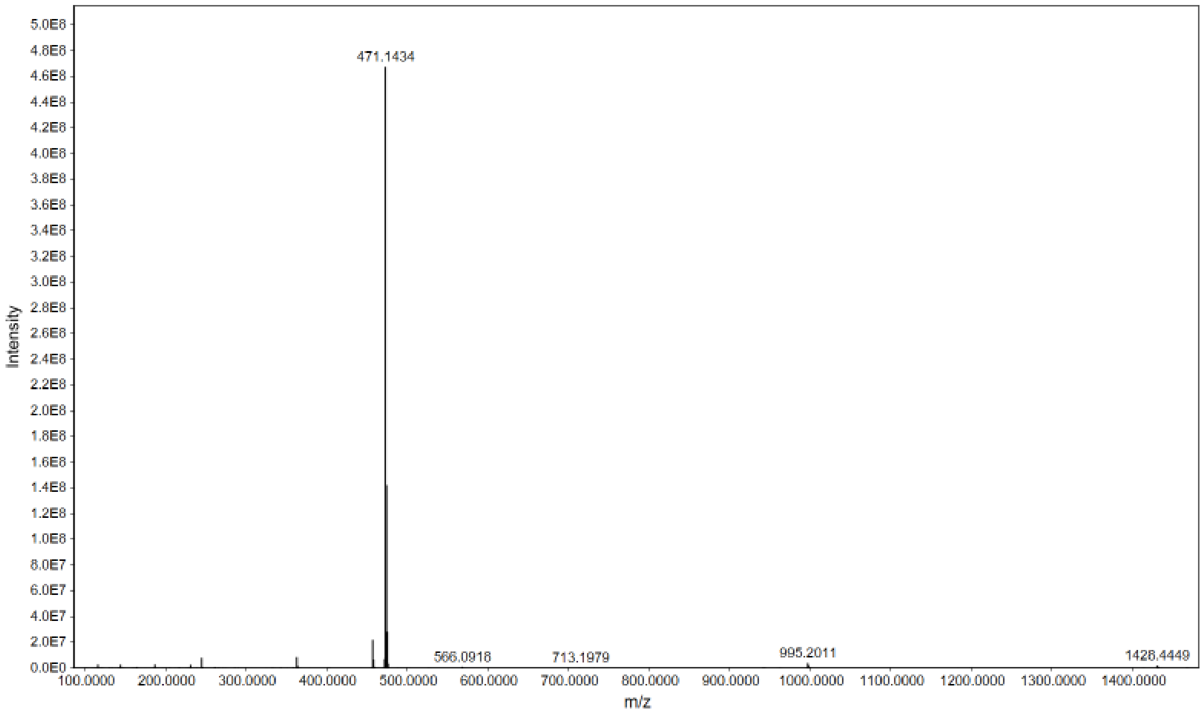

S1.2. HRESIMS+ spectrum of Gnetin D in MeOH.

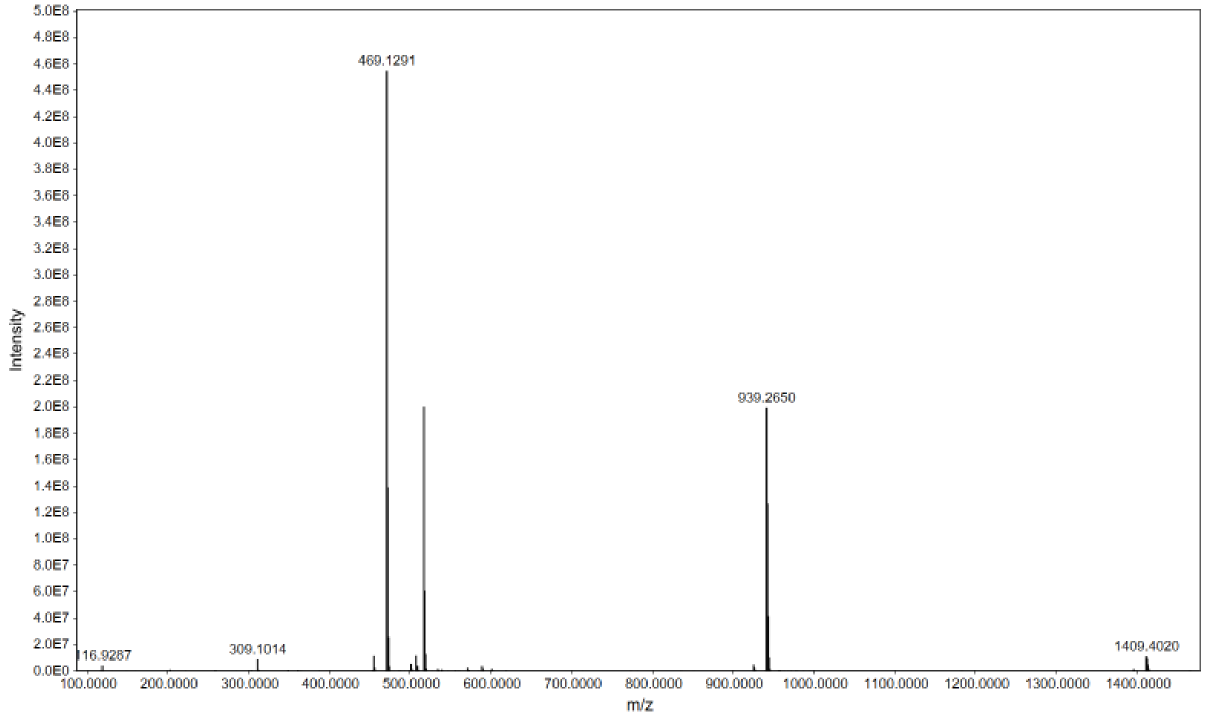

S1.3. HRESIMS- spectrum of Gnetin D in MeOH.

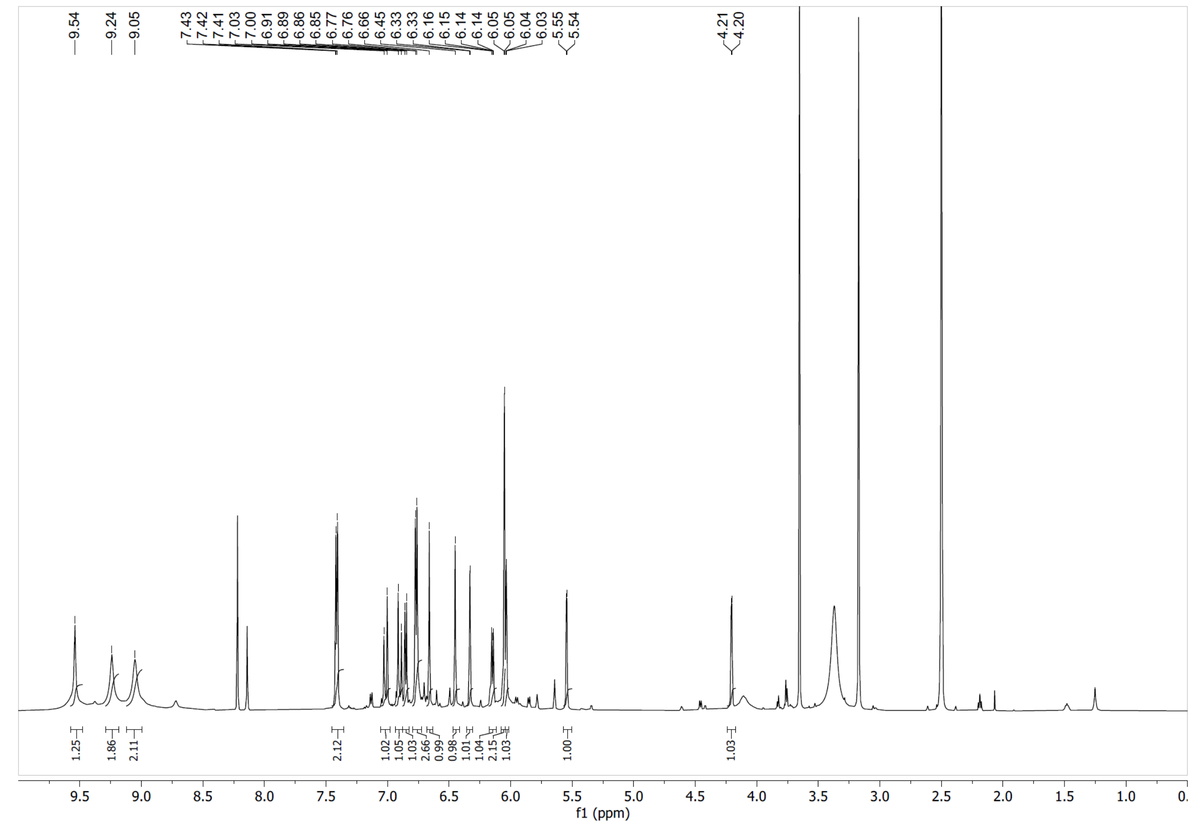

S1.4. ^{1}H NMR spectrum of Gnetin D in DMSO-d_6 at 600 MHz.

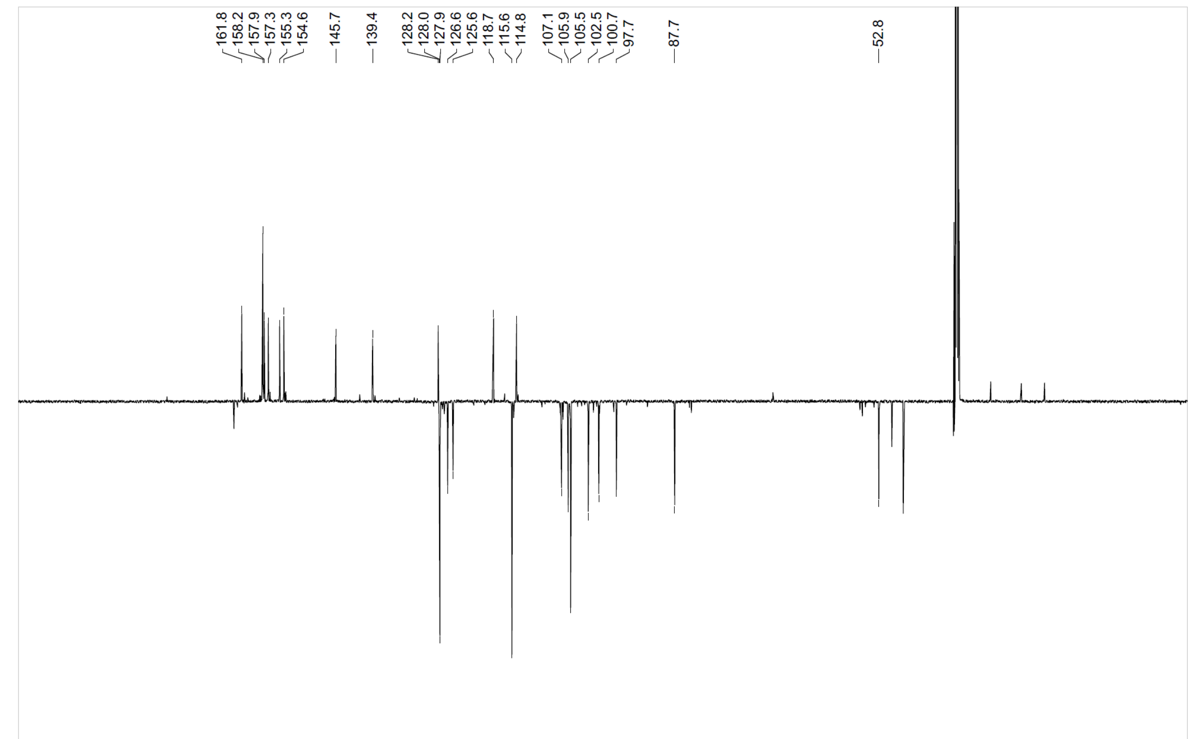

S1.5. ^{13}C NMR spectrum of Gnetin D in DMSO-d_6 at 151 MHz.

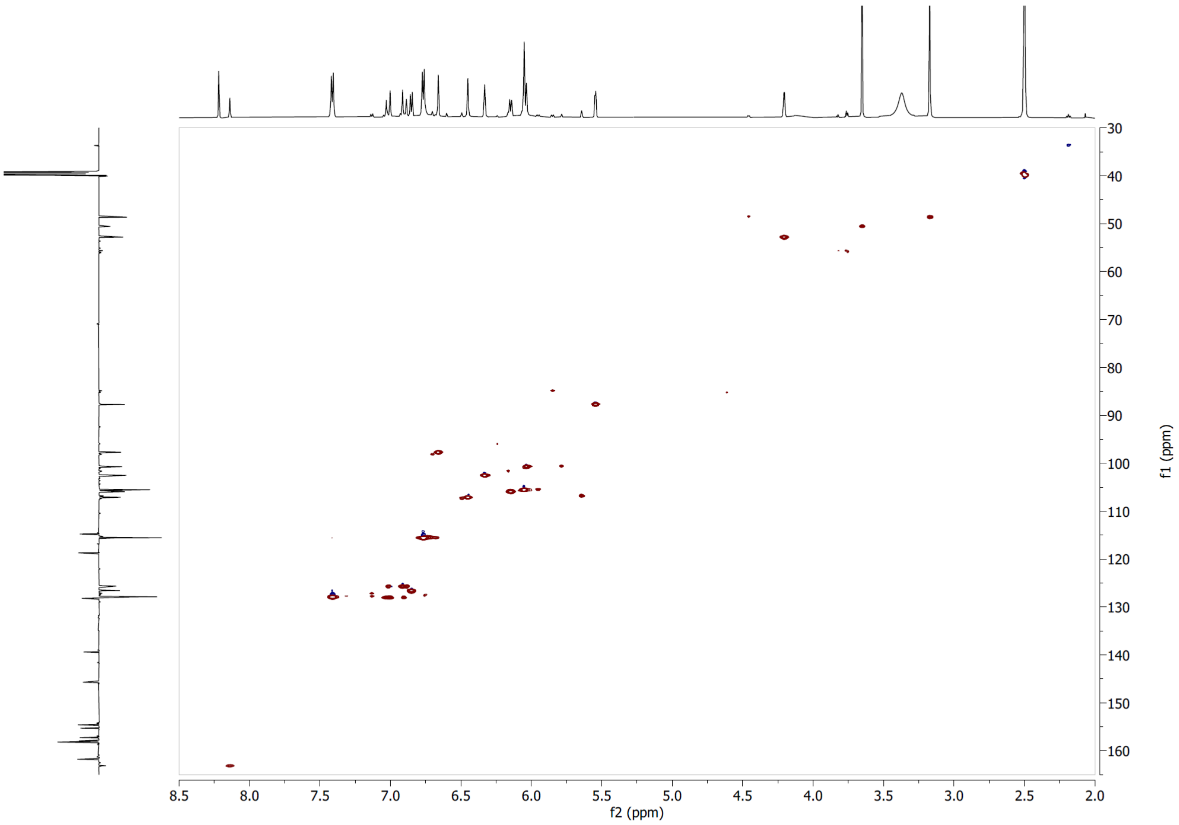

S1.6. HSQC NMR spectrum of Gnetin D in DMSO-d_6.

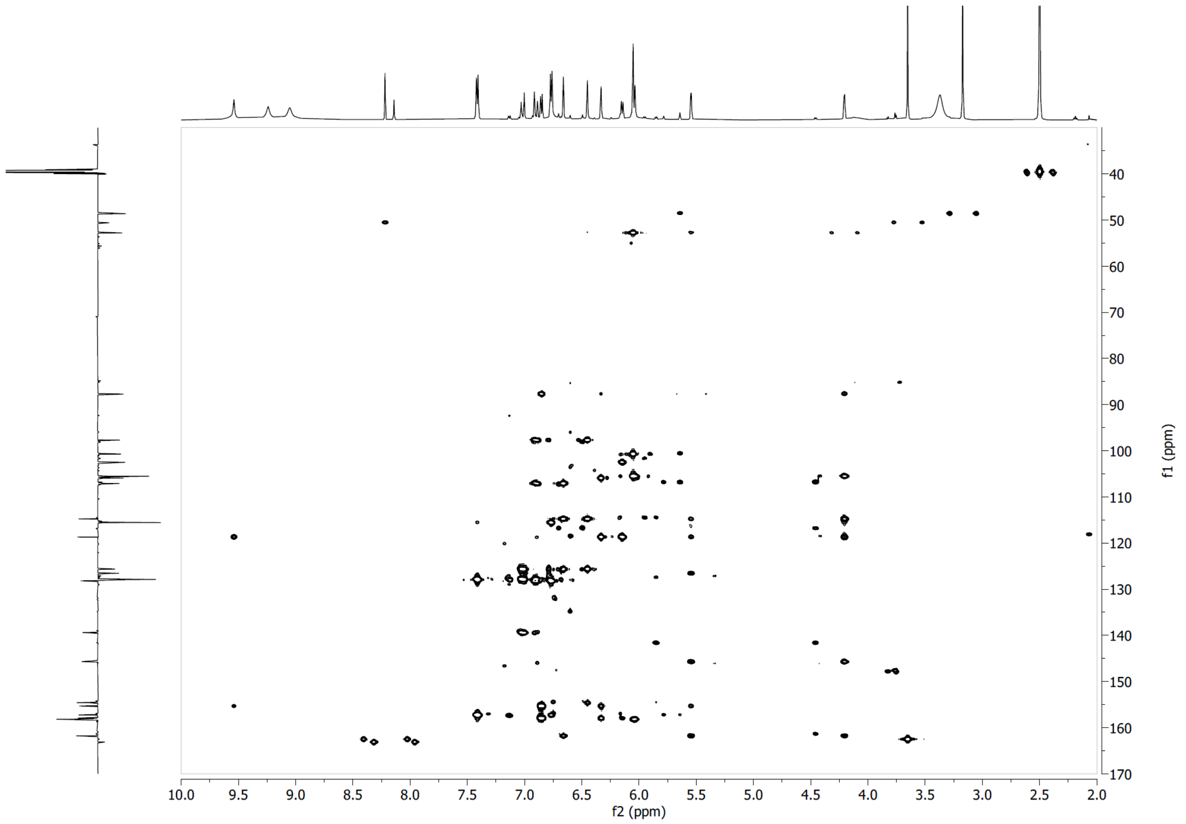

S1.7. HMBC NMR spectrum of Gnetin D in DMSO-d_6.

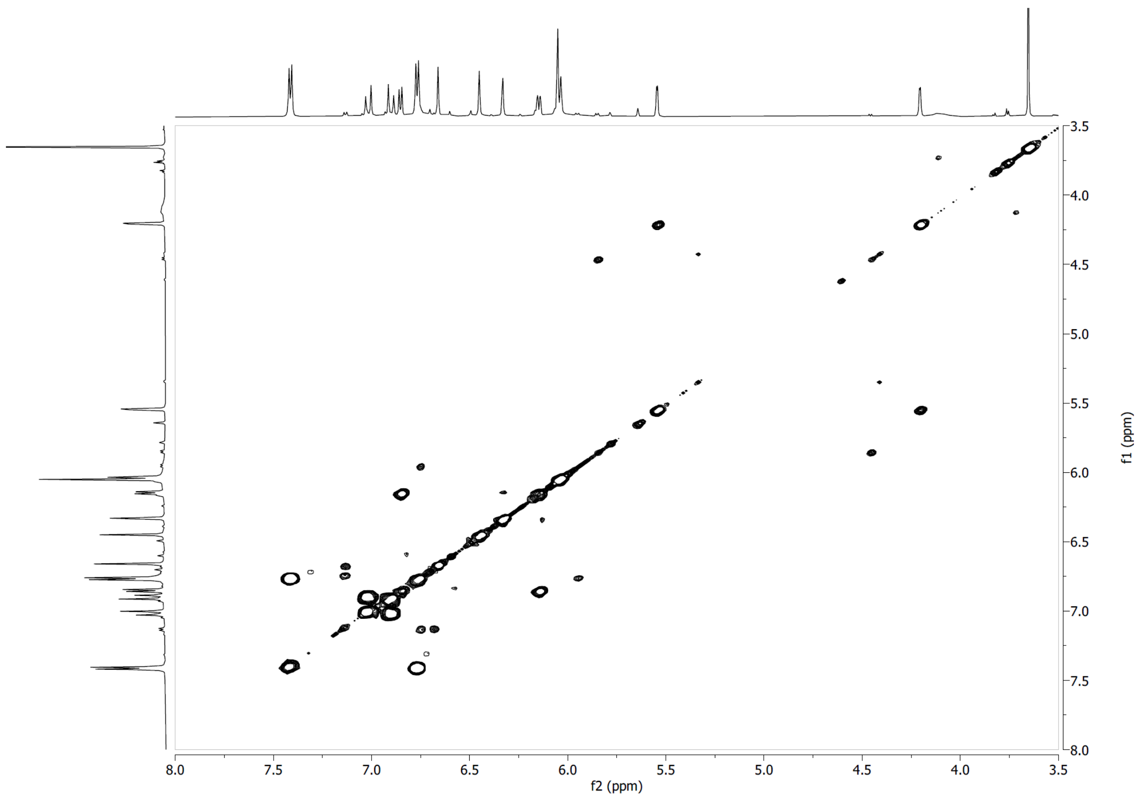

S1.8. COSY NMR spectrum of Gnetin D in DMSO-d_6.

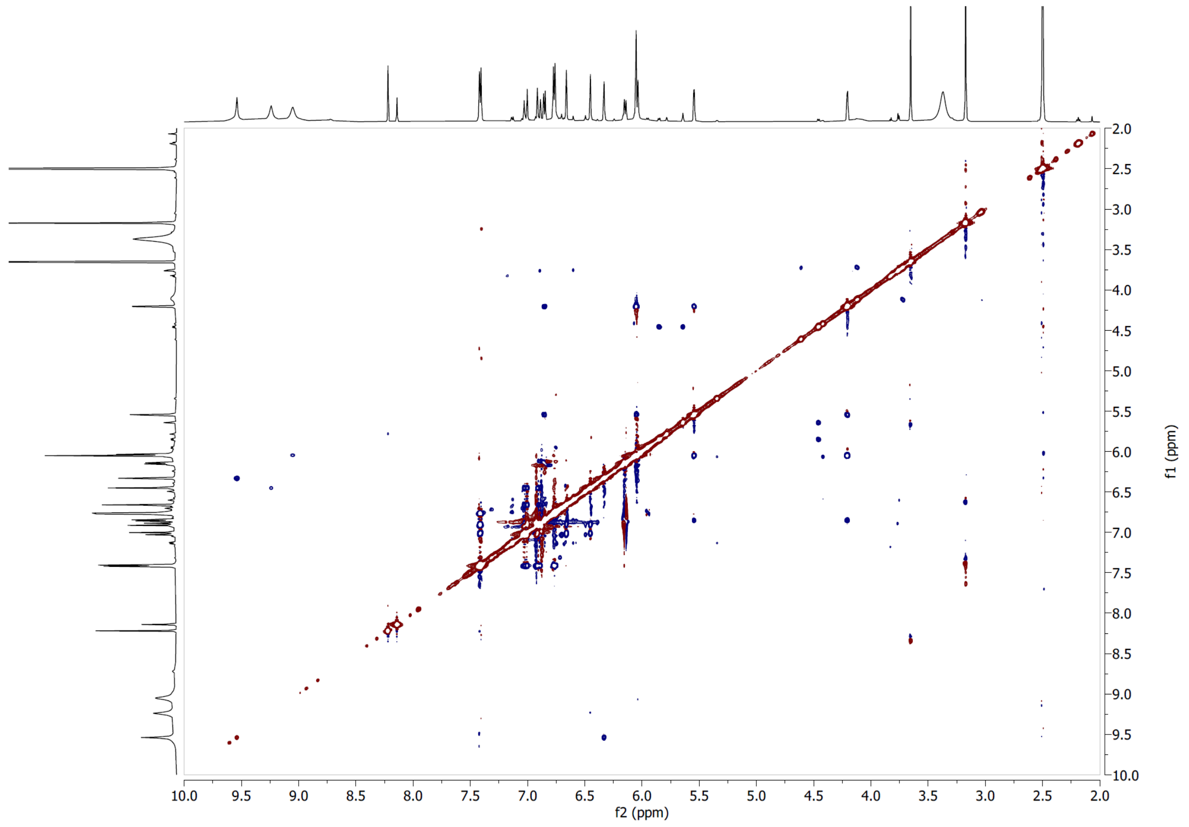

S1.9. ROESY NMR spectrum of Gnetin D in DMSO-d_6.

2. (+)-Gnetupendin C:

Experimental:

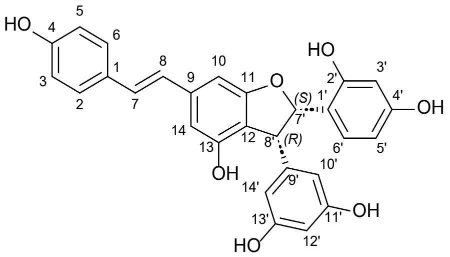

(+)-Gnetupendin C (2) $[\alpha]_D^{20}$ +69.6 (c 0.16, MeOH), Enantiomer of (-)-Gnetupendin C reported[5]: $[\alpha]_D^{20}$ -220.0 (c 0.10, MeOH); UV (MeOH) λ_{max} (log ε) 226 (4.57), 287 (4.19), 310 (4.32), 328 (4.34), 347 (4.06) nm; ^{1}H NMR (DMSO-d_6, 600 MHz) δ 9.55 (1H, s, 4-OH), 9.37 (1H, s, 2'-OH), 9.23 (1H, s, 13-OH), 8.97 (1H, s, 4'-OH), 8.71 (2H, s, 11'-OH, 13'-OH), 7.41 (2H, d, J = 8.6 Hz, H-2, H-6), 7.03 (1H, d, J = 16.3 Hz, H-7), 6.91 (1H, d, J = 16.3 Hz, H-8), 6.77 (2H, d, J = 8.6 Hz, H-3, H-5), 6.75 (1H, d, J = 8.3 Hz, H-6'), 6.70 (1H, d, J = 1.3 Hz, H-10), 6.49 (1H, d, J = 1.3 Hz, H-14), 6.16 (1H, d, J = 2.3 Hz, H-3'), 5.94 (1H, dd, J = 8.3, 2.3 Hz, H-5'), 5.84 (1H, d, J = 7.7 Hz, H-7'), 5.78 (1H, t, J = 2.2 Hz, H-12'), 5.63 (2H, d, J = 2.2 Hz, H-10', H-14'), 4.45 (1H, d, J = 7.7 Hz, H-8'); ^{13}C NMR (DMSO-d_6, 151 MHz) δ 161.3 (C-11), 157.2 (C-4), 157.1 (C-11', C-13'), 156.9 (C-4'), 154.4 (C-2'), 154.2 (C-13), 141.6 (C-9'), 139.0 (C-9), 128.2 (C-1), 127.9 (CH-7), 127.9 (CH-2, CH-6), 127.4 (CH-6'), 125.7 (CH-8), 116.8 (C-12), 115.5 (CH-3, CH-5), 114.5 (C-1'), 107.3 (CH-14), 106.8 (CH-10', CH-14'), 105.4 (CH-5'), 101.6 (CH-3'), 100.6 (CH-12'), 98.1 (CH-10), 84.8 (CH-7'), 48.6 (CH-8'); (NP-MRD ID: NP0332862); HRESIMS m/z 471.1433 $[M+H]^+$ (calcd for $C_{28}H_{23}O_7^+$ 471.1438, Δ = -1.06 ppm), MS/MS spectrum: CCMSLIB00012474991, m/z 469.1289 $[M-H]^-$ (calcd for $C_{28}H_{21}O_7^-$ 469.1293, Δ = -0.85 ppm).

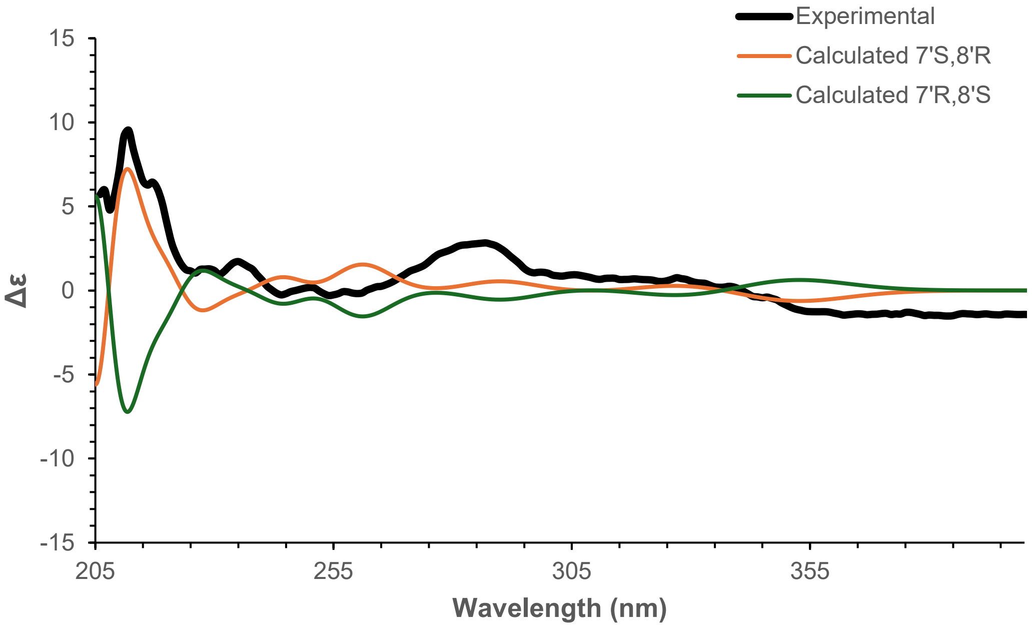

S2.1. ECD spectra (experimental and calculated) of (+)-Gnetupendin C in MeOH.

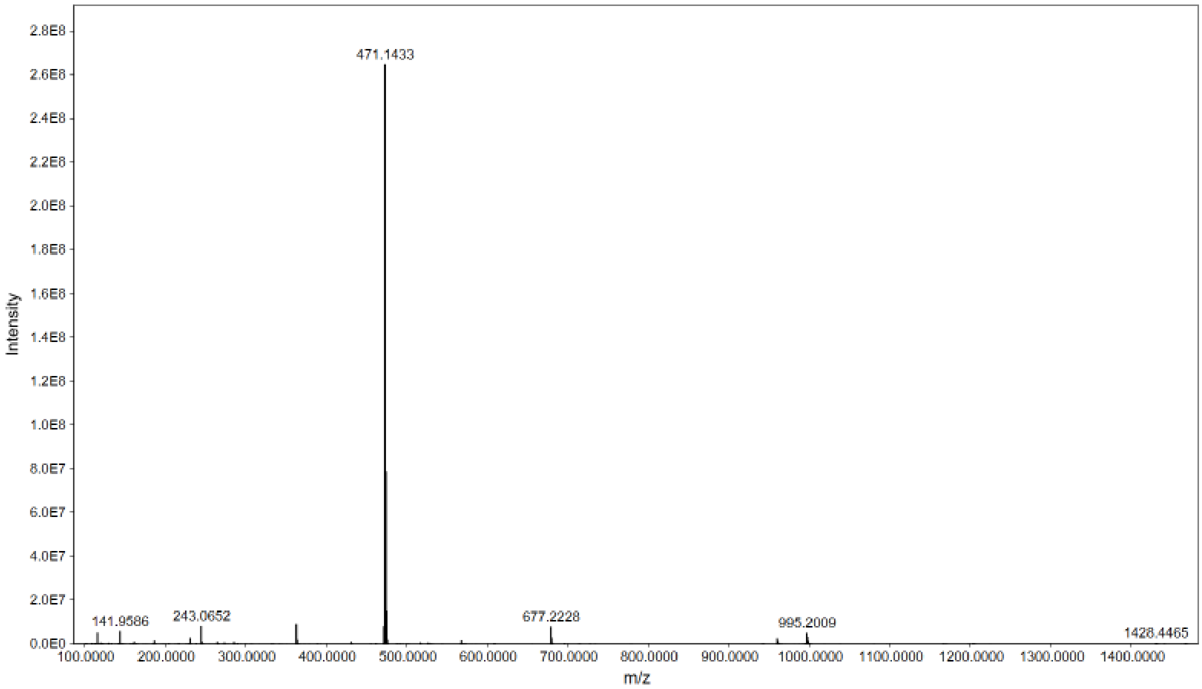

S2.2. HRESIMS+ spectrum of (+)-Gnetupendin C in MeOH.

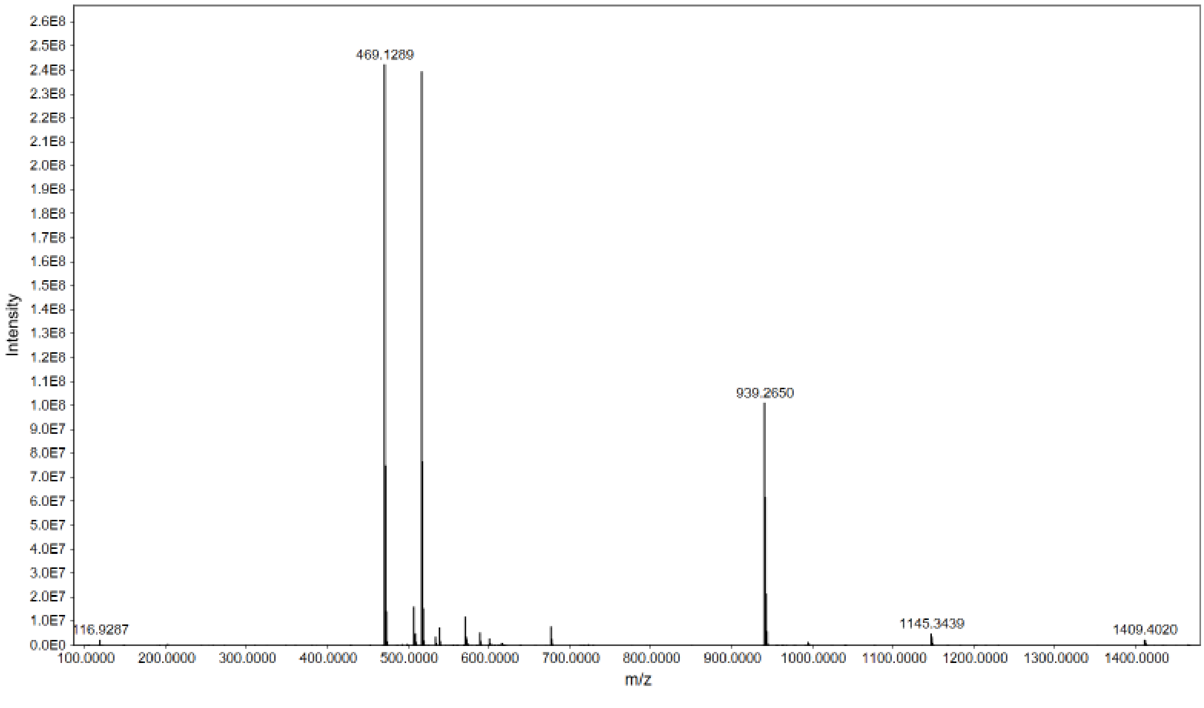

S2.3. HRESIMS- spectrum of (+)-Gnetupendin C in MeOH.

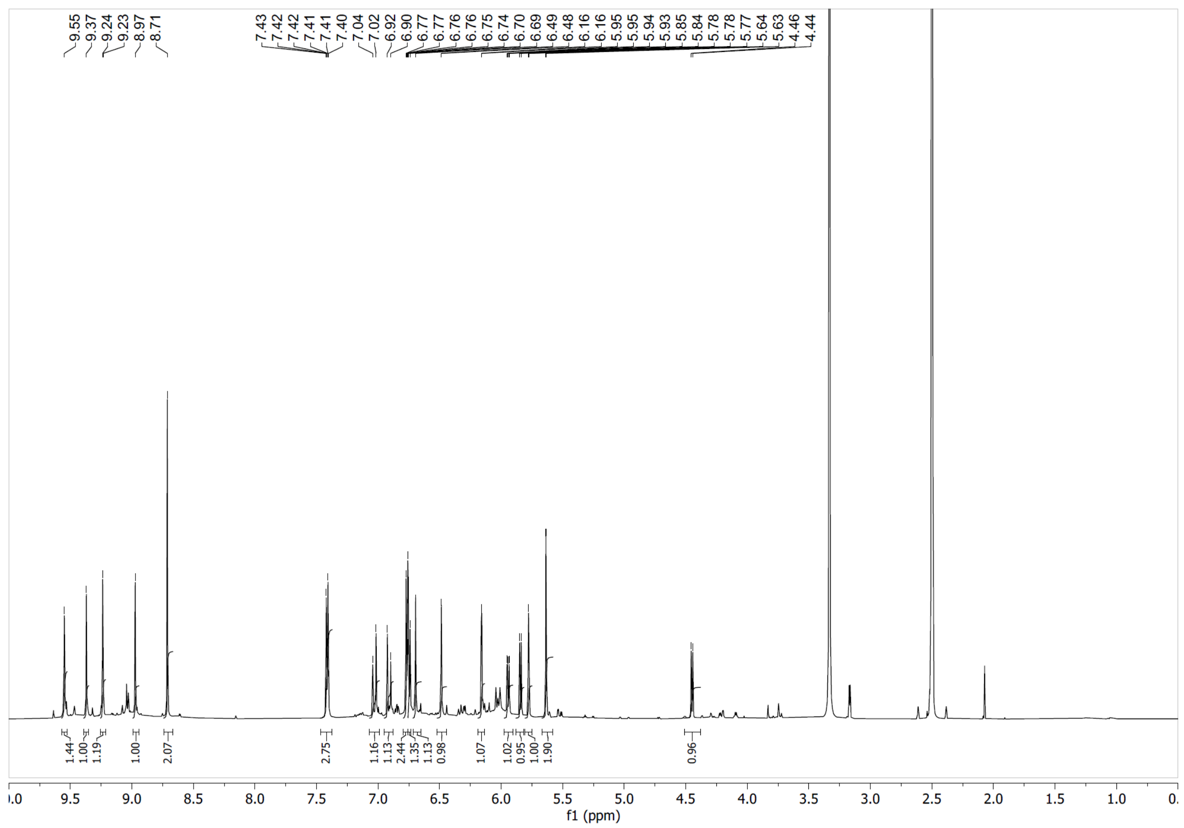

S2.4 ^{1}H NMR spectrum of (+)-Gnetupendin C in DMSO-d_6 at 600 MHz.

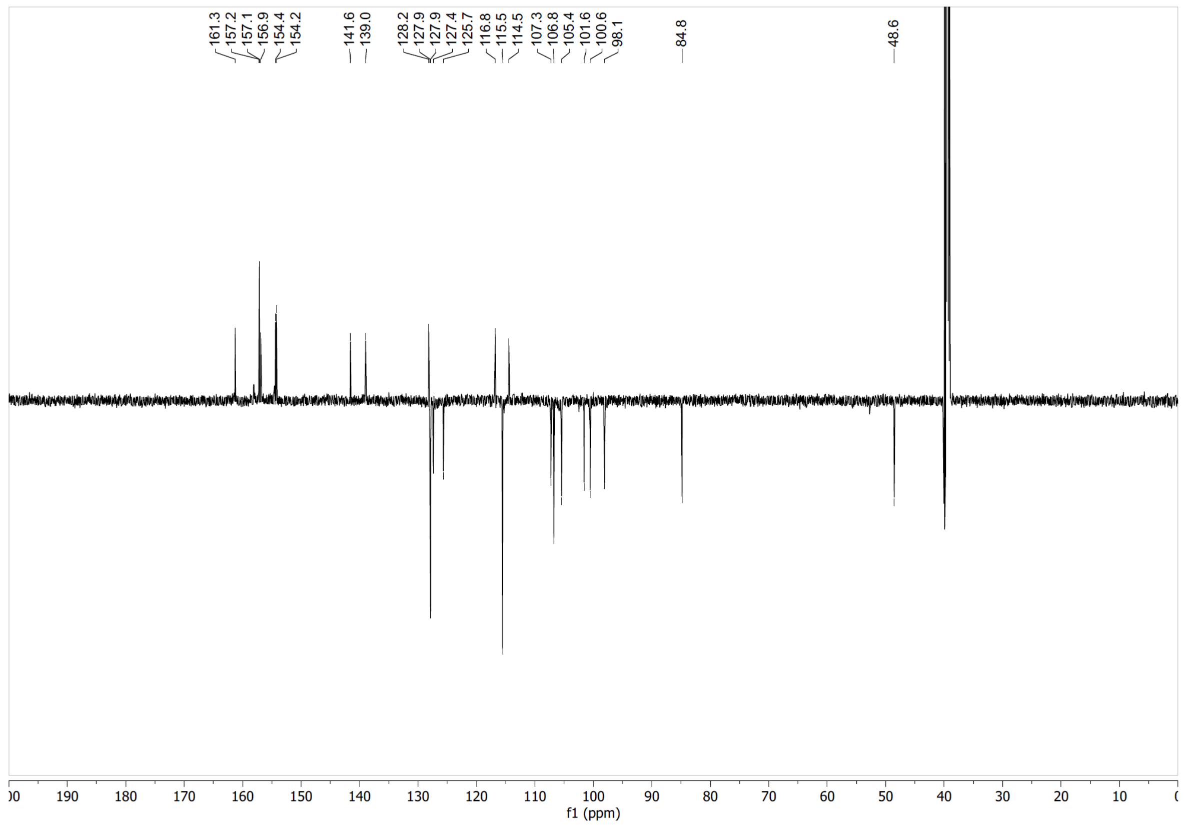

S2.5. ^{13}C NMR spectrum of (+)-Gnetupendin C in DMSO-d_6 at 151 MHz.

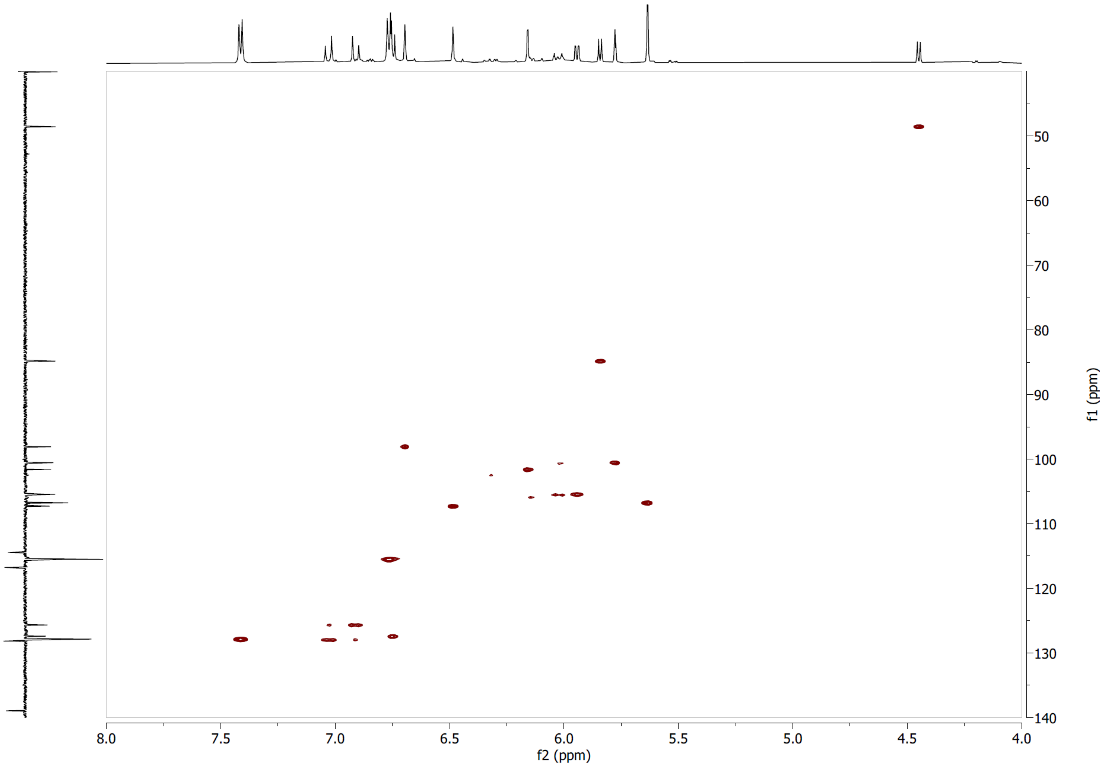

S2.6. HSQC NMR spectrum of (+)-Gnetupendin C in DMSO-d_6.

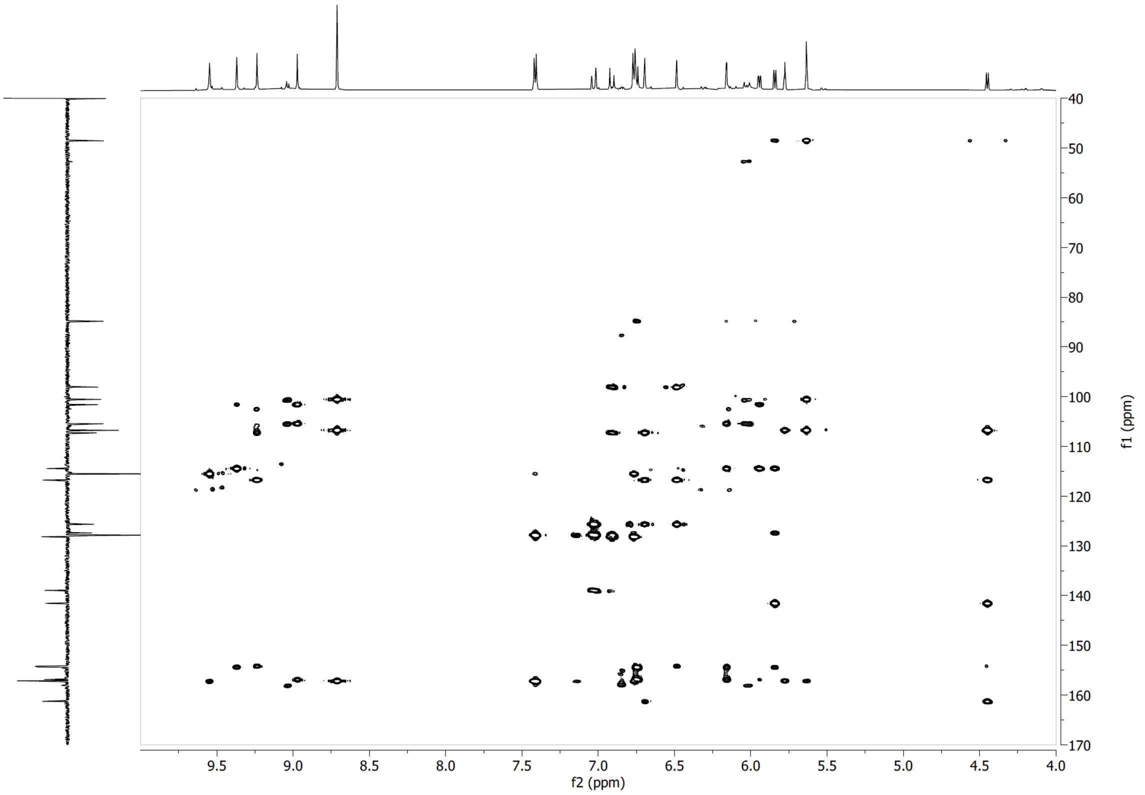

S2.7. HMBC NMR spectrum of (+)-Gnetupendin C in DMSO-d_6.

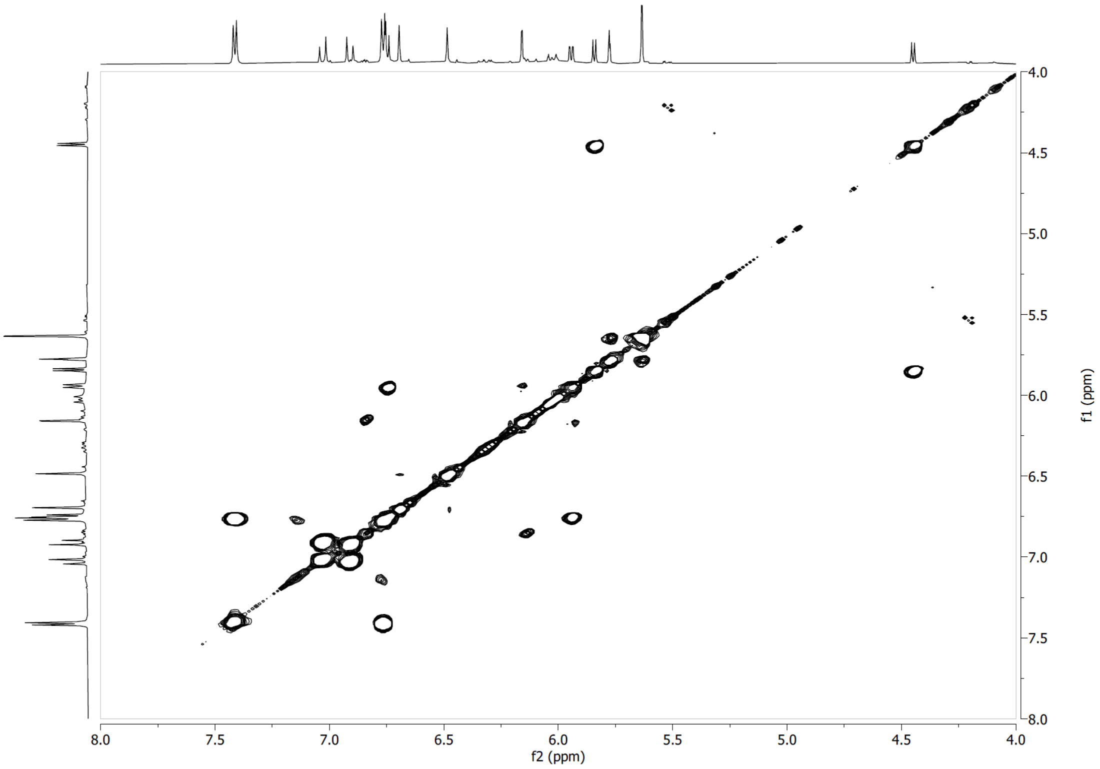

S2.8. COSY NMR spectrum of (+)-Gnetupendin C in DMSO-*d*$_6$.

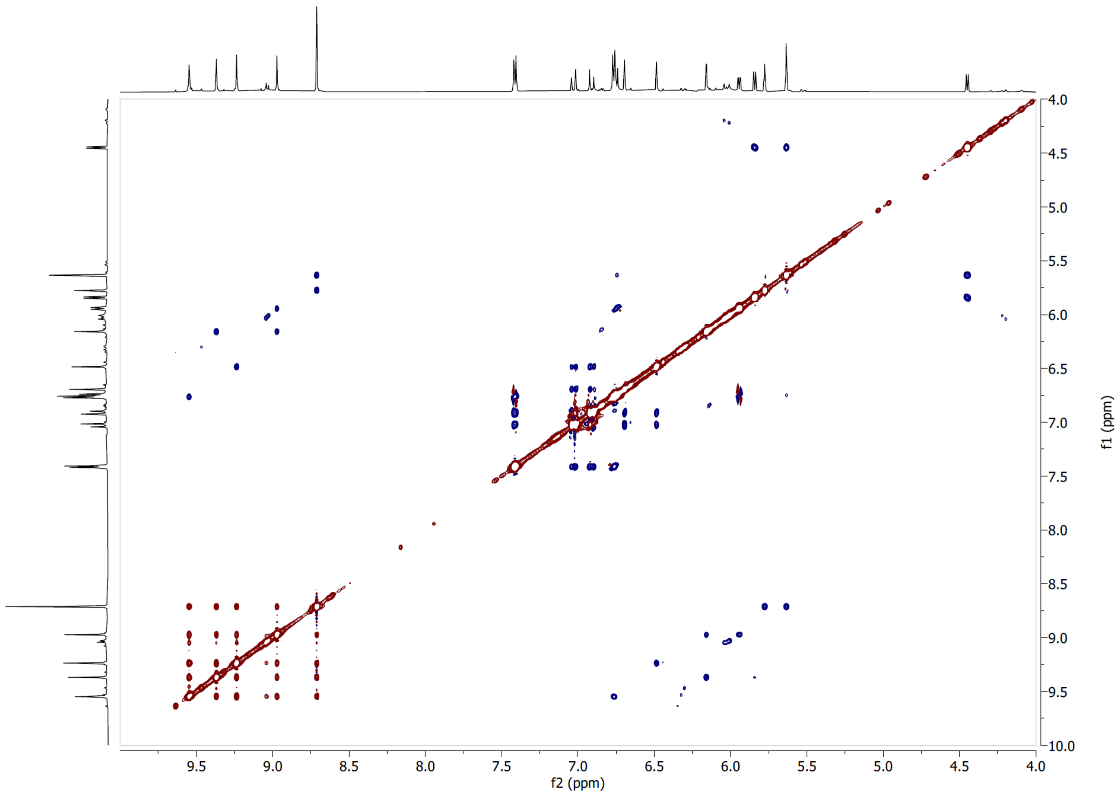

S2.9. ROESY NMR spectrum of (+)-Gnetupendin C in DMSO-*d*$_6$.

3. Gnetin C:

Experimental:

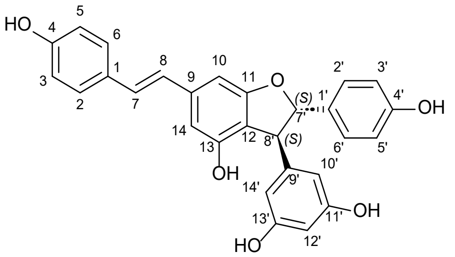

Gnetin C (3) $[\alpha]_D^{20}$ -4.61 (c 0.09, MeOH), Literature[6] : $[\alpha]_D^{20}$ -1 (c 0.1, MeOH); UV (MeOH) λ_{max} (log ε) 226 (4.23), 287 (3.74), 310 (3.86), 328 (3.88), 347 (3.61) nm; ^{1}H NMR (DMSO-d_6, 600 MHz) δ 7.41 (2H, d, *J* = 8.6 Hz, H-2, H-6), 7.11 (2H, d, *J* = 8.6 Hz, H-2', H-6'), 7.02 (1H, d, *J* = 16.3 Hz, H-7), 6.91 (1H, d, *J* = 16.3 Hz, H-8), 6.77 (2H, d, *J* = 8.6 Hz, H-3, H-5), 6.75 (2H, d, *J* = 8.6 Hz, H-3', H-5'), 6.65 (1H, s, H-10), 6.48 (1H, s, H-14), 6.05 (1H, t, *J* = 2.1 Hz, H-12'), 5.99 (2H, d, *J* = 2.1 Hz, H-10', H-14'), 5.31 (1H, d, *J* = 4.5 Hz, H-7'), 4.23 (1H, d, *J* = 4.5 Hz, H-8'); ^{13}C NMR (DMSO-d_6, 151 MHz) δ 161.5 (C-11), 158.4 (C-11', C-13'), 157.3 (C-4), 157.2 (C-4'), 154.6 (C-13), 145.0 (C-9'), 139.6 (C-9), 132.2 (C-1'), 128.1 (C-1), 128.1 (CH-7), 127.9 (CH-2, CH-6), 126.8 (CH-2', CH-6'), 125.5 (CH-8), 115.5 (CH-3, CH-5), 115.3 (CH-3', CH-5'), 114.0 (C-12), 107.2 (CH-14), 105.3 (CH-10', CH-14'), 100.9 (CH-12'), 97.7 (CH-10), 92.0 (CH-7'), 54.3 (CH-8'); (NP-MRD ID: NP0061298); HRESIMS *m/z* 455.1487 [M+H]$^+$ (calcd for $C_{28}H_{23}O_6^+$ 455.1489, Δ = -0.44 ppm), MS/MS spectrum: CCMSLIB00012475006, *m/z* 453.1338 [M-H]$^-$ (calcd for $C_{28}H_{21}O_6^-$ 453.1344, Δ = -1.32 ppm).

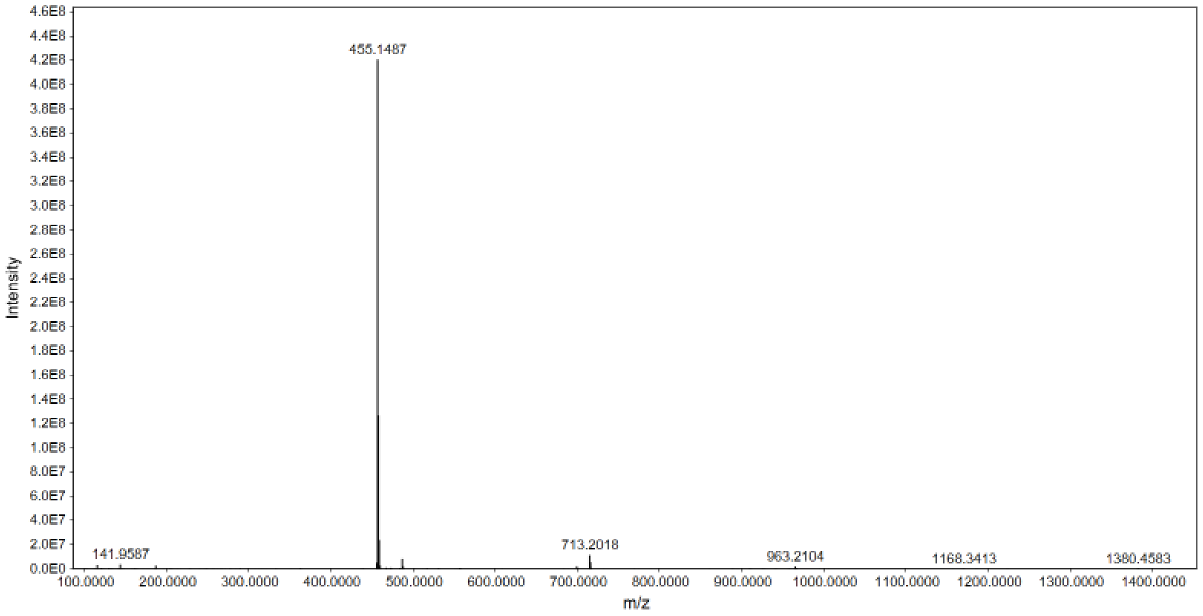

S3.1. HRESIMS+ spectrum of Gnetin C in MeOH.

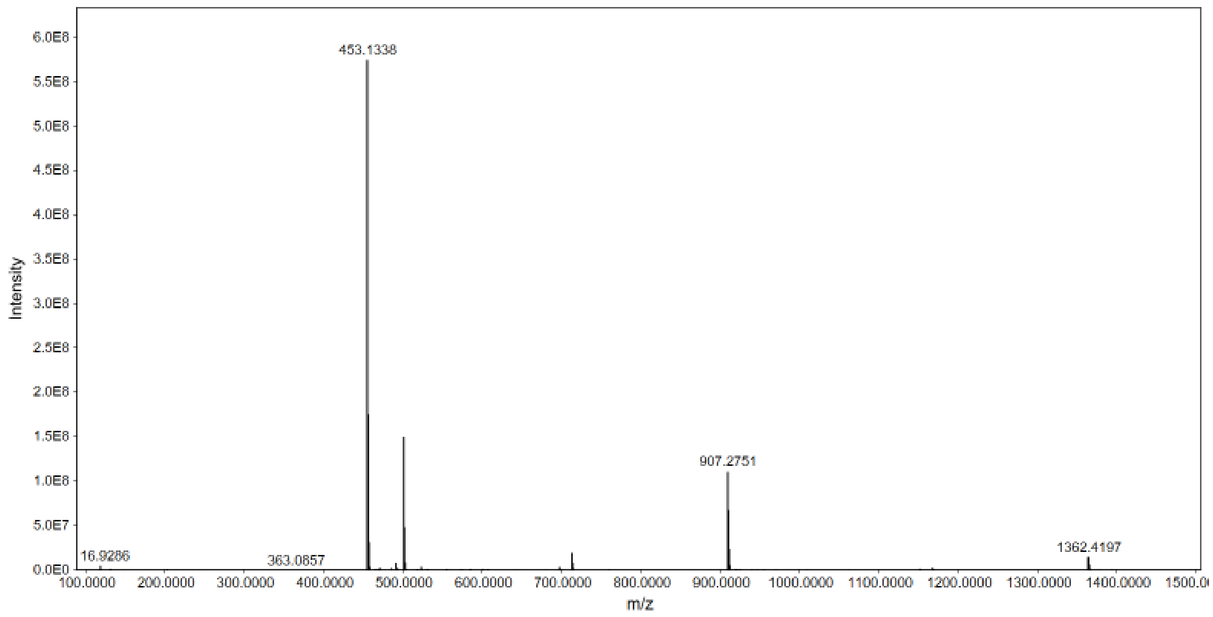

S3.2. HRESIMS- spectrum of Gnetin C in MeOH.

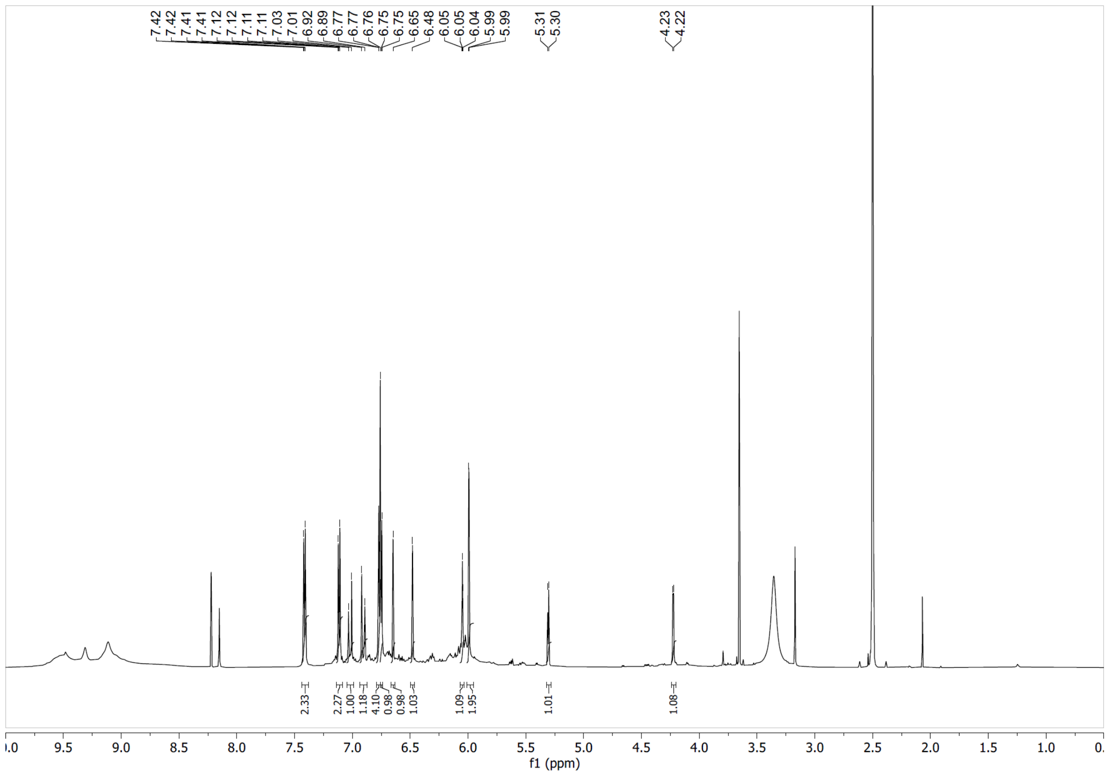

S3.3. ^{1}H NMR spectrum of Gnetin C in DMSO-d_6 at 600 MHz.

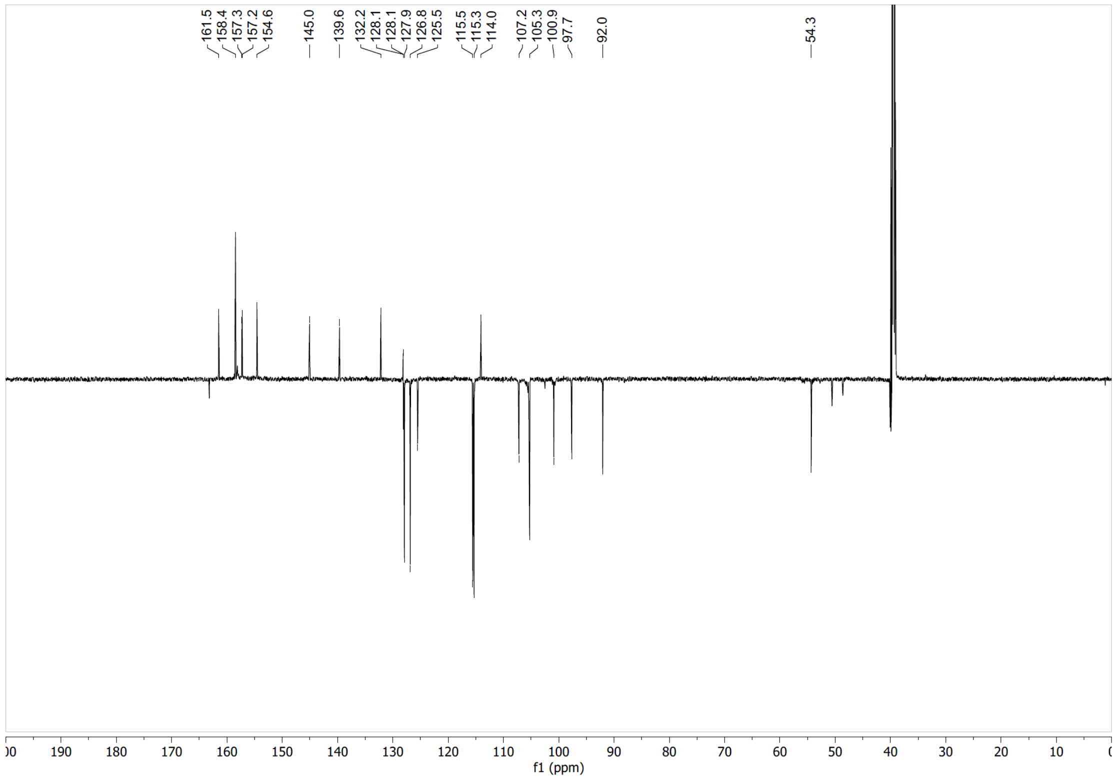

S3.4. 13C NMR spectrum of Gnetin C in DMSO-d6 at 151 MHz.

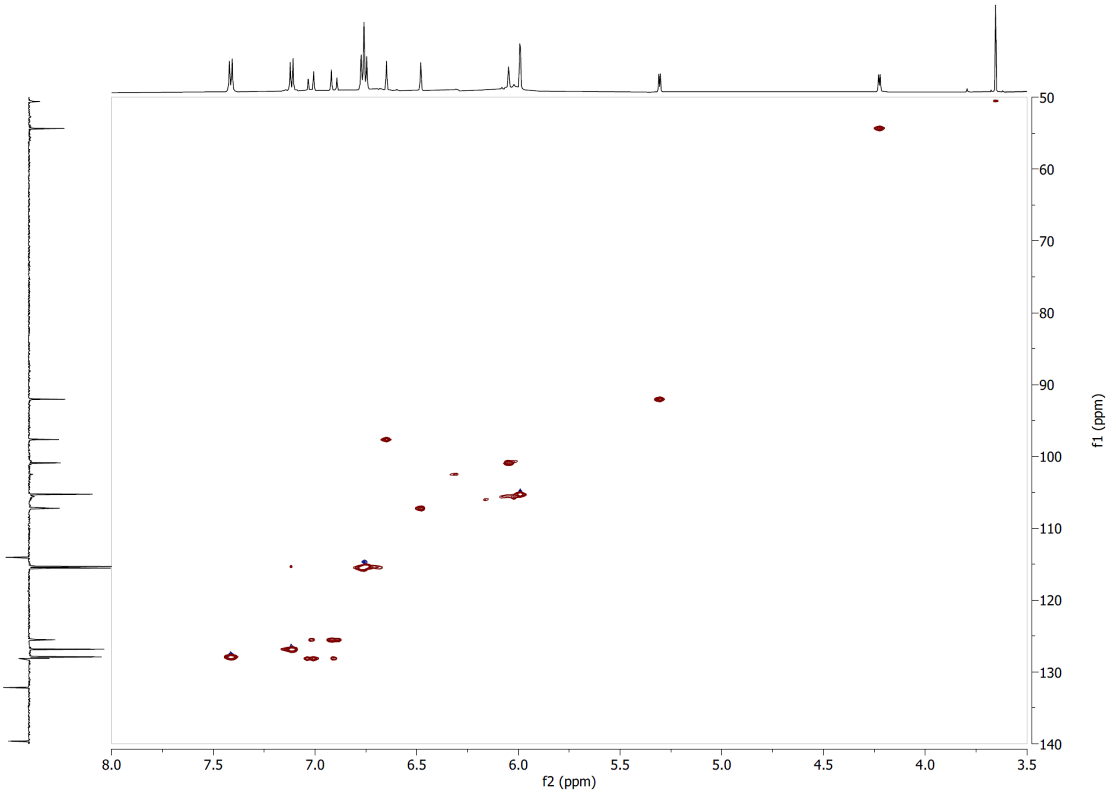

S3.5. HSQC NMR spectrum of Gnetin C in DMSO-*d*$_6$.

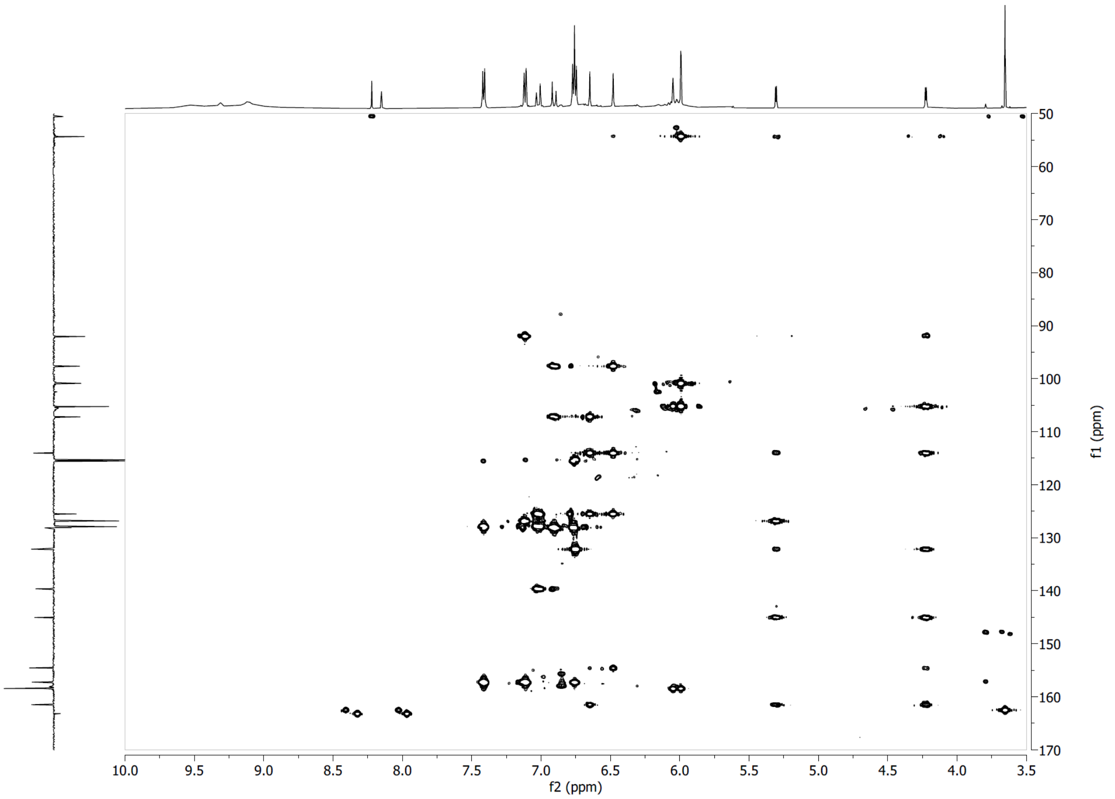

S3.6. HMBC NMR spectrum of Gnetin C in DMSO-d_6.

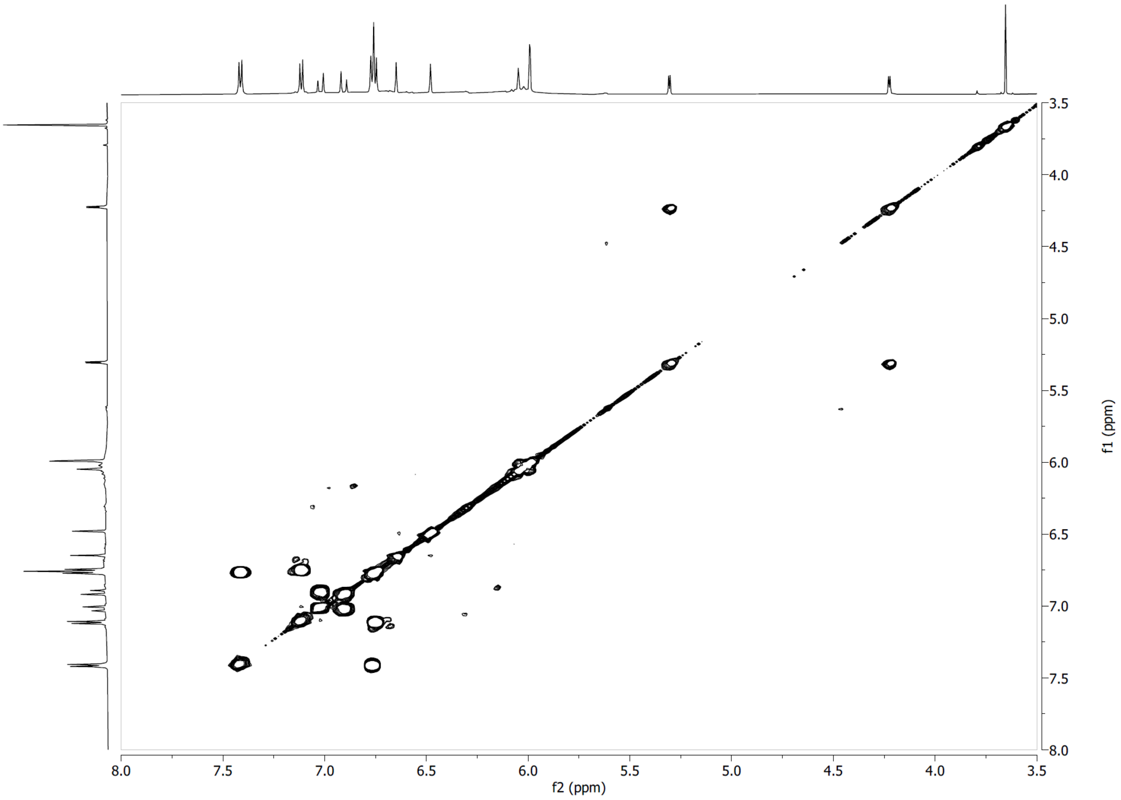

S3.7. COSY NMR spectrum of Gnetin C in DMSO-d_6.

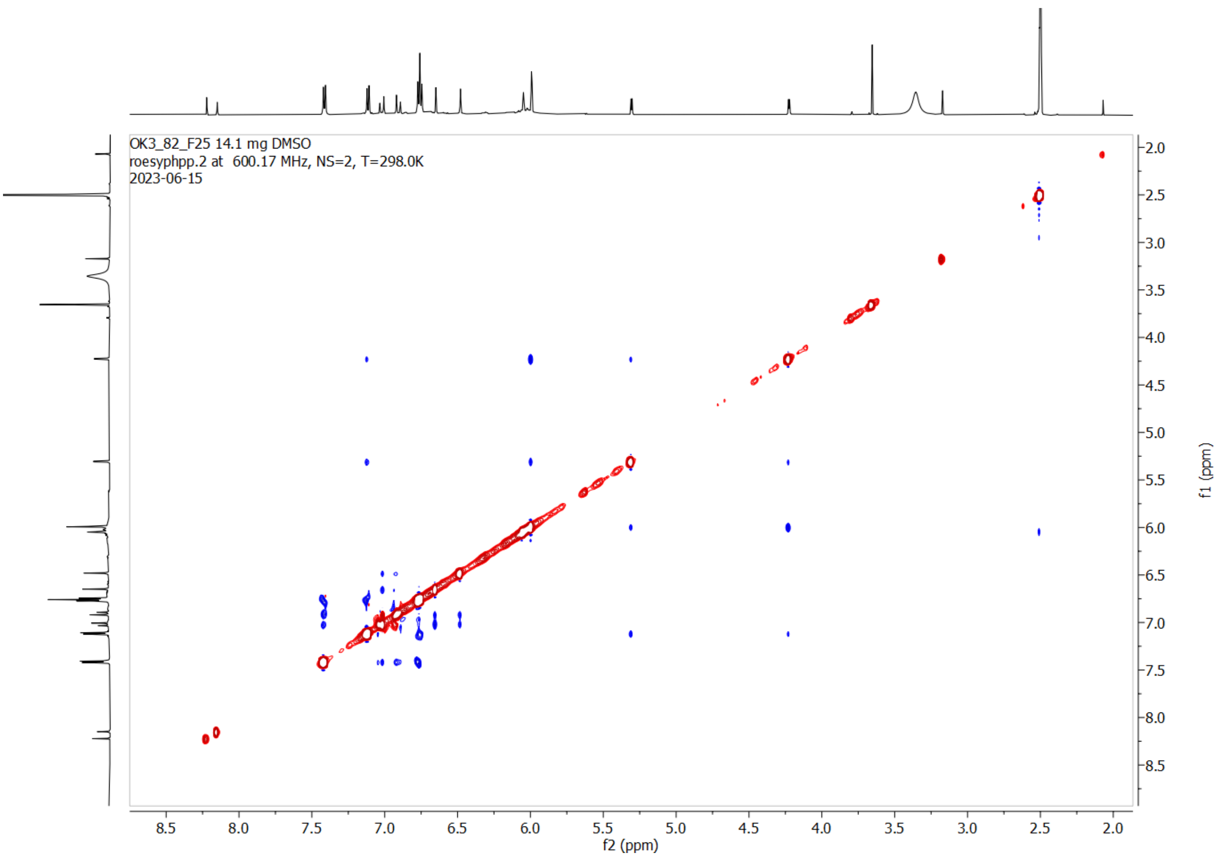

S3.8. ROESY NMR spectrum of Gnetin C in DMSO-d_6.

4. Gnetoline A:

Experimental:

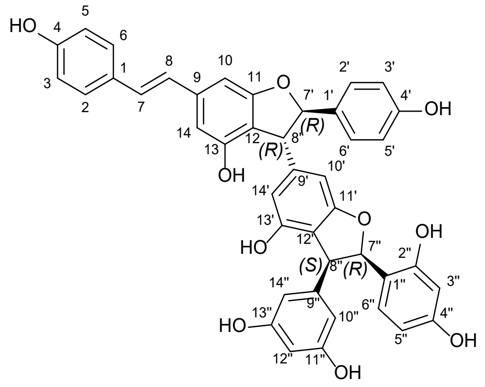

Gnetoline A (4) $[\alpha]_D^{20}$ +19.7 (c 0.17, MeOH); UV (MeOH) λ_{max} (log ε) 226 (4.47), 287 (3.94), 310 (4.02), 328 (4.05), 347 (3.80) nm; ^{1}H NMR (DMSO-d_6, 600 MHz) δ 9.55 (1H, s, 4-OH), 9.48 (1H, s, 4'-OH), 9.40 (1H, s, 13-OH), 9.33 (1H, s, 2''-OH), 9.18 (1H, s, 11'-OH), 8.95 (1H, s, 4''-OH), 8.72 (2H, s, 11''-OH, 13''-OH), 7.42 (2H, d, *J* = 8.6 Hz, H-2, H-6), 7.16 (2H, d, *J* = 8.6 Hz, H-2', H-6'), 7.03 (1H, d, *J* = 16.3 Hz, H-7), 6.92 (1H, d, *J* = 16.3 Hz, H-8), 6.76 (4H, d, *J* = 8.6 Hz, H-3, H-3', H-5, H-5'), 6.75 (1H, d, *J* = 8.4 Hz, H-6''), 6.68 (1H, d, *J* = 1.1 Hz, H-10), 6.50 (1H, d, *J* = 1.1 Hz, H-14), 6.19 (1H, s, H-10'), 6.15 (1H, s, H-14'), 6.13 (1H, d, *J* = 2.3 Hz, H-3''), 5.93 (1H, dd, *J* = 8.4, 2.3 Hz, H-5''), 5.82 (1H, d, *J* = 7.9 Hz, H-7''), 5.78 (1H, t, *J* = 2.2 Hz, H-12''), 5.63 (2H, d, *J* = 2.2 Hz, H-10'', H-14''), 5.40 (1H, d, *J* = 3.9 Hz, H-7'), 4.42 (1H, d, *J* = 7.9 Hz, H-8''), 4.35 (1H, d, *J* = 3.9 Hz, H-8'); ^{13}C NMR (DMSO-d_6, 151 MHz) δ 161.5 (C-11), 160.9 (C-11'), 157.3 (C-4), 157.2 (C-4'), 157.1 (C-11'', C-13''), 156.8 (C-4''), 154.6 (C-13), 154.3 (C-2''), 154.2 (C-13'), 144.5 (C-9'), 141.6 (C-9''), 139.7 (C-9), 132.3 (C-1'), 128.2 (CH-7), 128.1 (C-1), 127.9 (CH-2, CH-6), 127.4 (CH-6''), 126.7 (CH-2', CH-6'), 125.5 (CH-8), 115.8 (C-12'), 115.5 (CH-3, CH-5), 115.3 (CH-3', CH-5'), 114.6 (C-1''), 113.9 (C-12), 107.3 (CH-14'), 106.8 (CH-10'', CH-14''), 105.4 (CH-5''), 101.6 (CH-3''), 100.5 (CH-12''), 99.7 (CH-10'), 97.7 (CH-10), 91.9 (CH-7'), 85.0 (CH-7''), 54.2 (CH-8'), 48.5 (CH-8''); (NP-MRD ID: NP0332863); HRESIMS *m/z* 697.2068 $[M+H]^+$ (calcd for $C_{42}H_{33}O_{10}^+$ 697.2068, Δ = 0 ppm), MS/MS spectrum: CCMSLIB00012475011, *m/z* 695.1917 $[M-H]^-$ (calcd for $C_{42}H_{31}O_{10}^-$ 695.1923, Δ = -0.86 ppm).

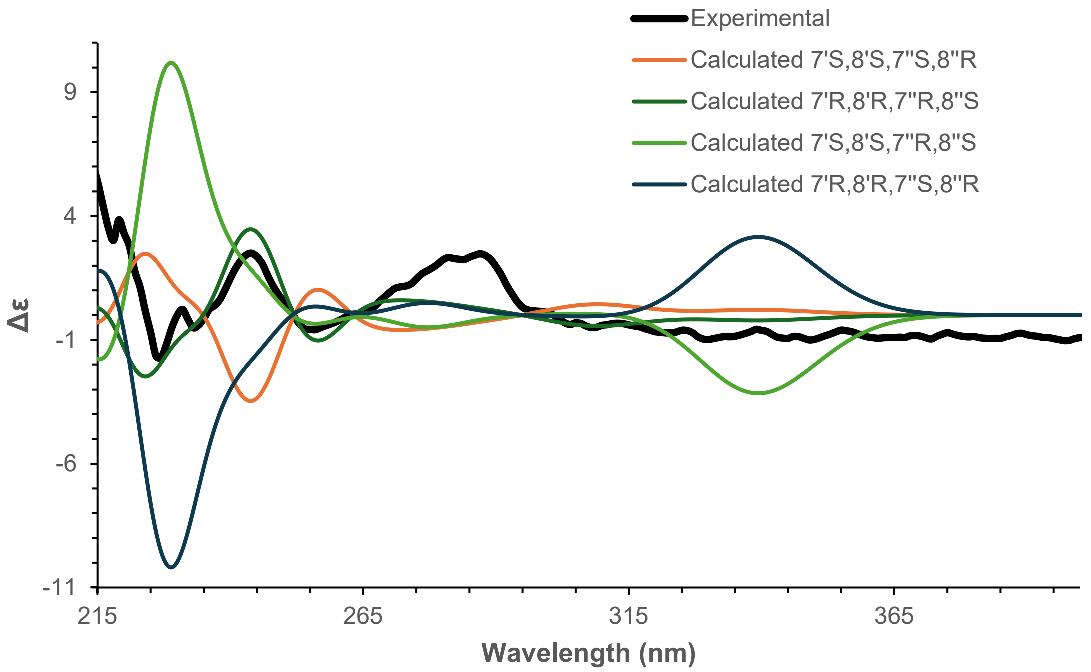

S4.1. ECD spectra (experimental and calculated) of Gnetoline A in MeOH.

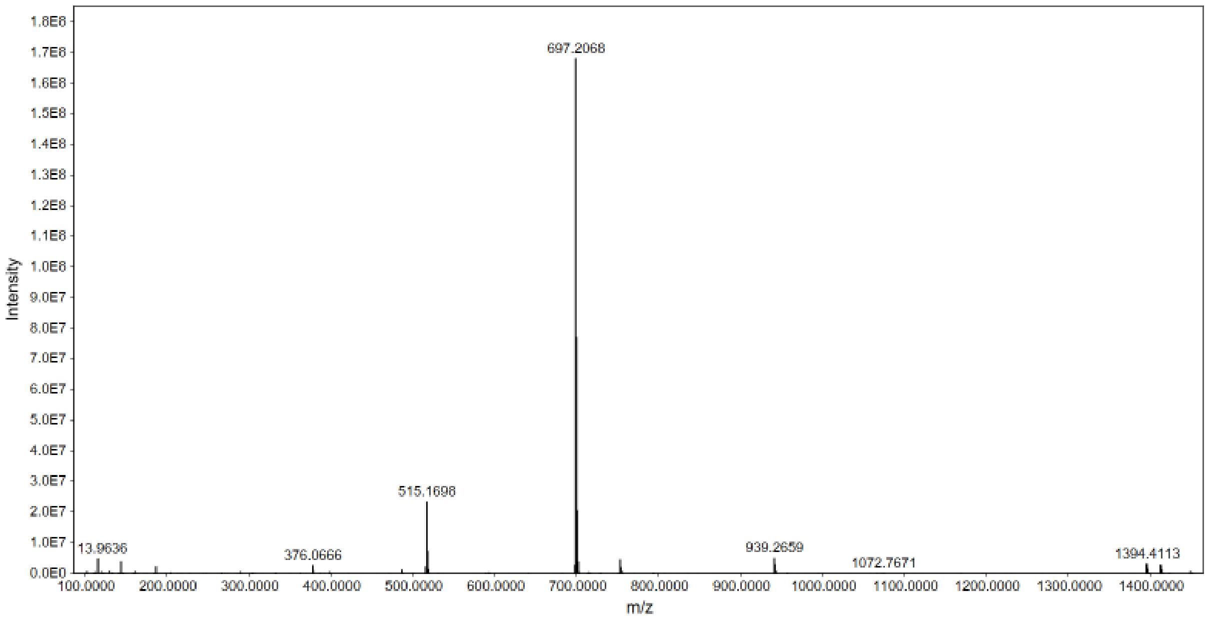

S4.2. HRESIMS+ spectrum of Gnetoline A in MeOH.

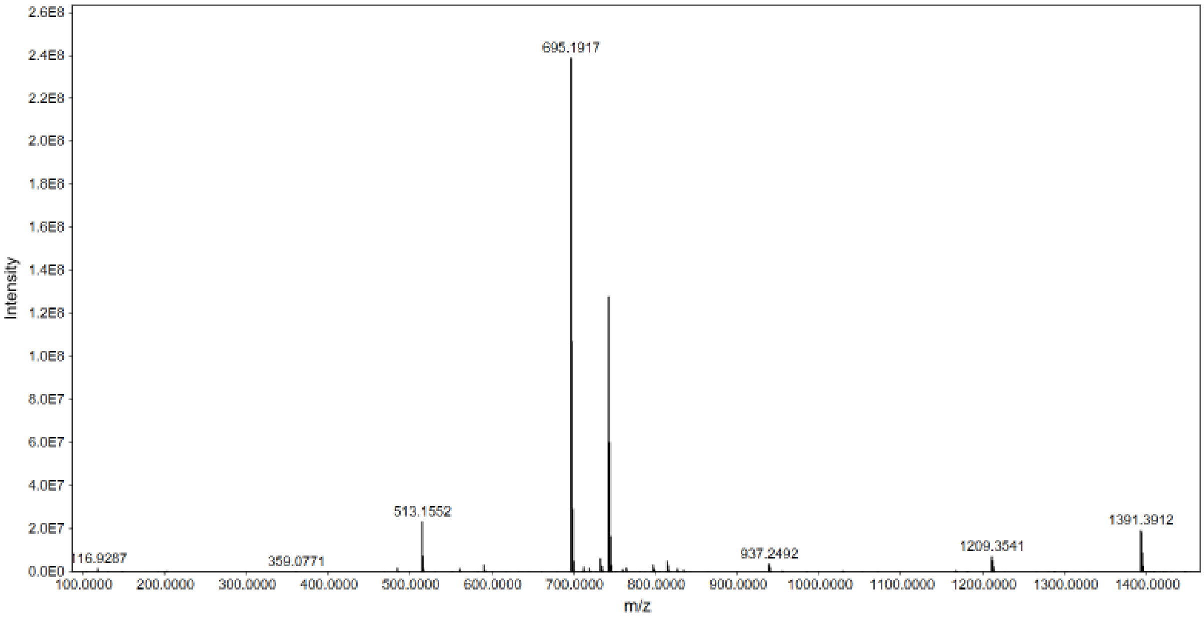

S4.3. HRESIMS- spectrum of Gnetoline A in MeOH.

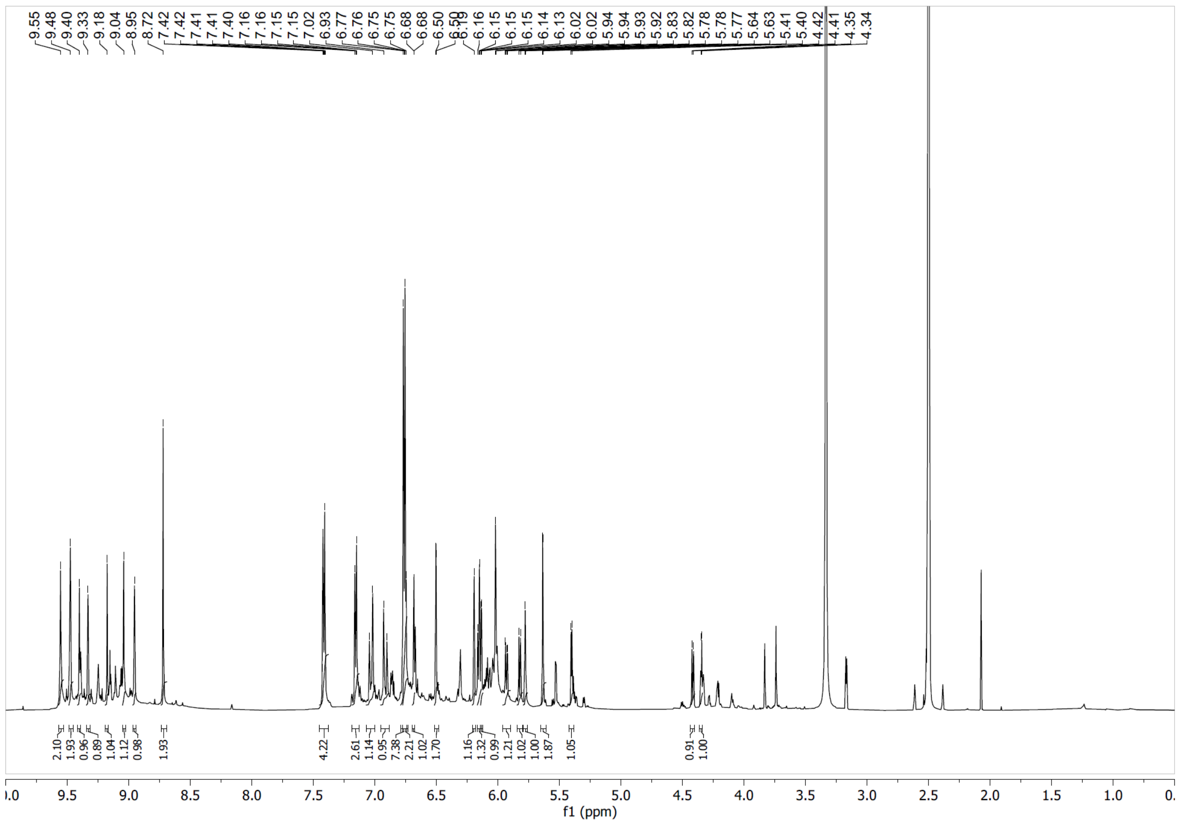

S4.4. ^{1}H NMR spectrum of Gnetoline A in DMSO-d_6 at 600 MHz.

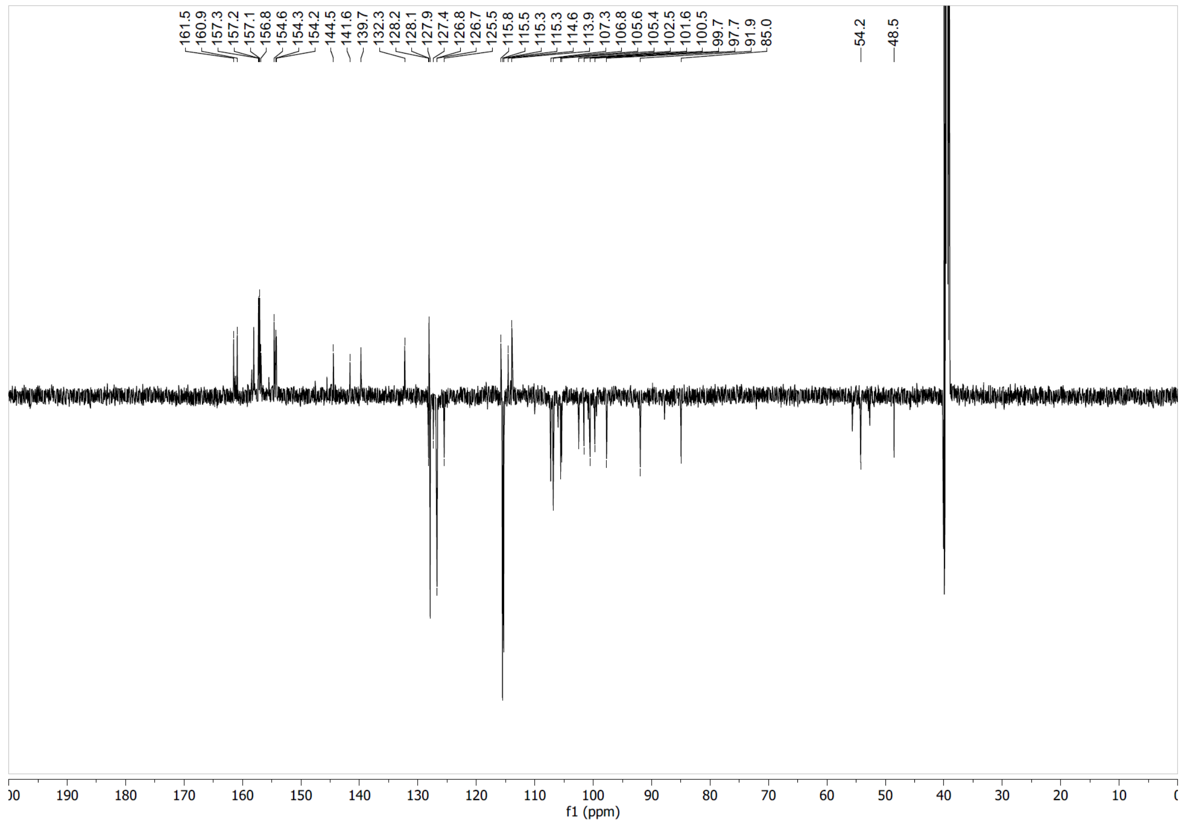

S4.5. ^{13}C NMR spectrum of Gnetoline A in DMSO-d_6 at 151 MHz.

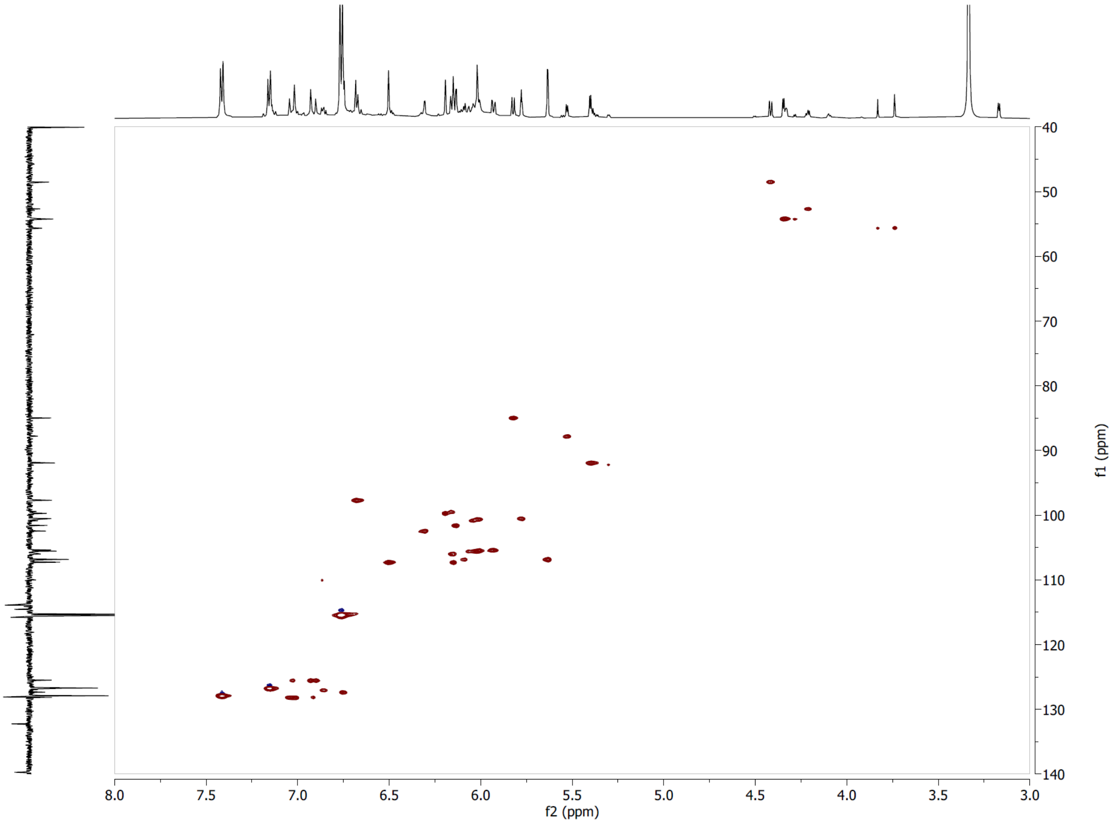

S4.6. HSQC NMR spectrum of Gnetoline A in DMSO-d_6.

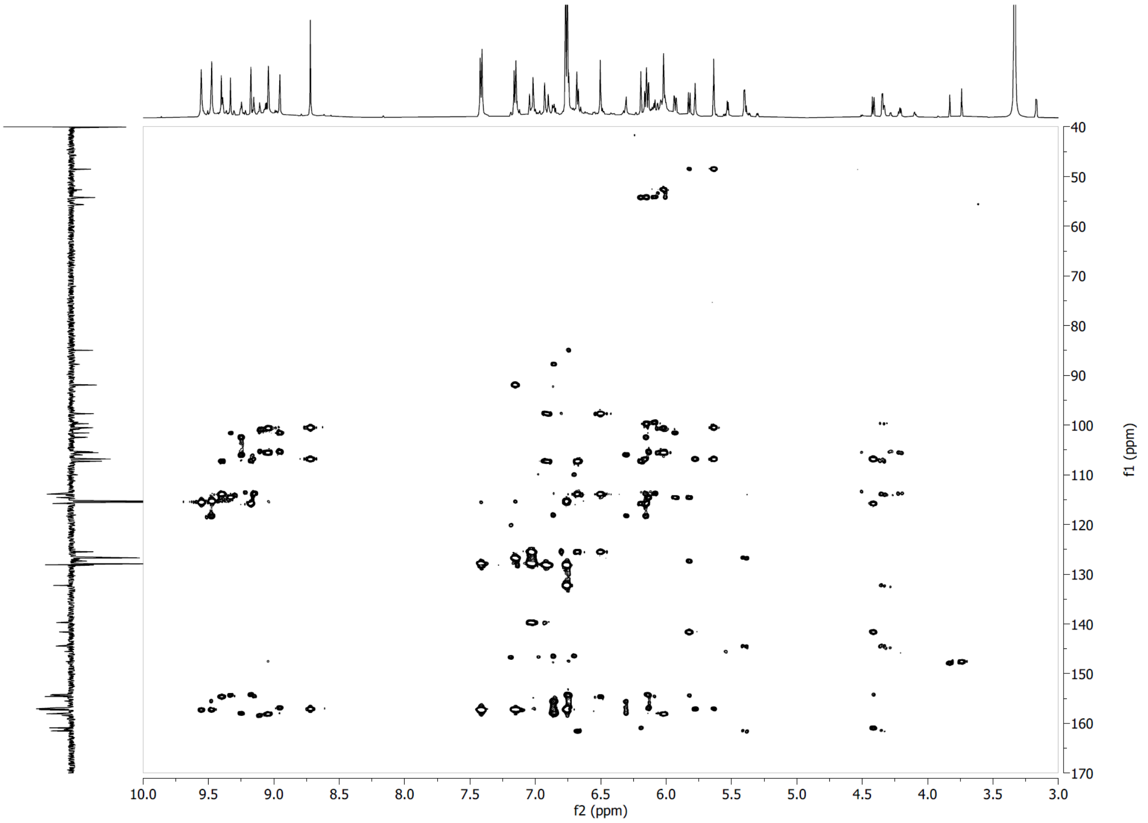

S4.7. HMBC NMR spectrum of Gnetoline A in DMSO-d_6.

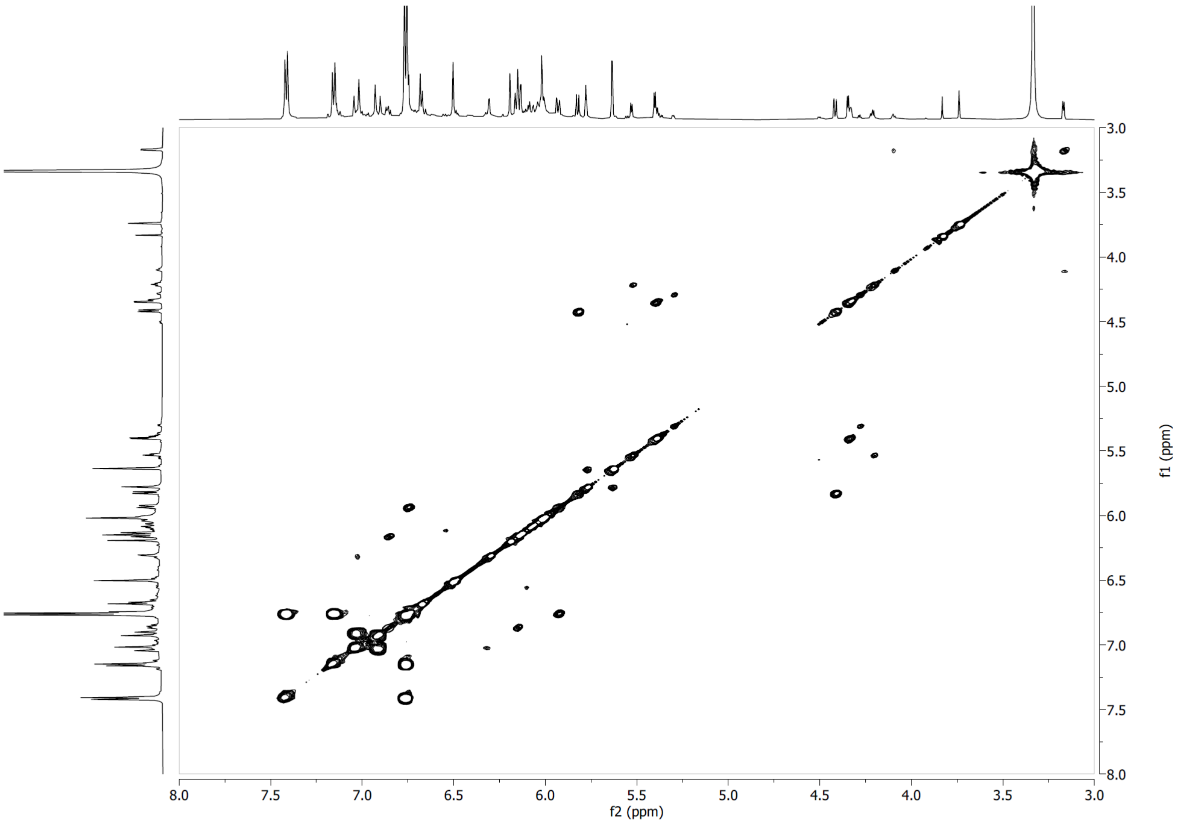

S4.8. COSY NMR spectrum of Gnetoline A in DMSO-d_6.

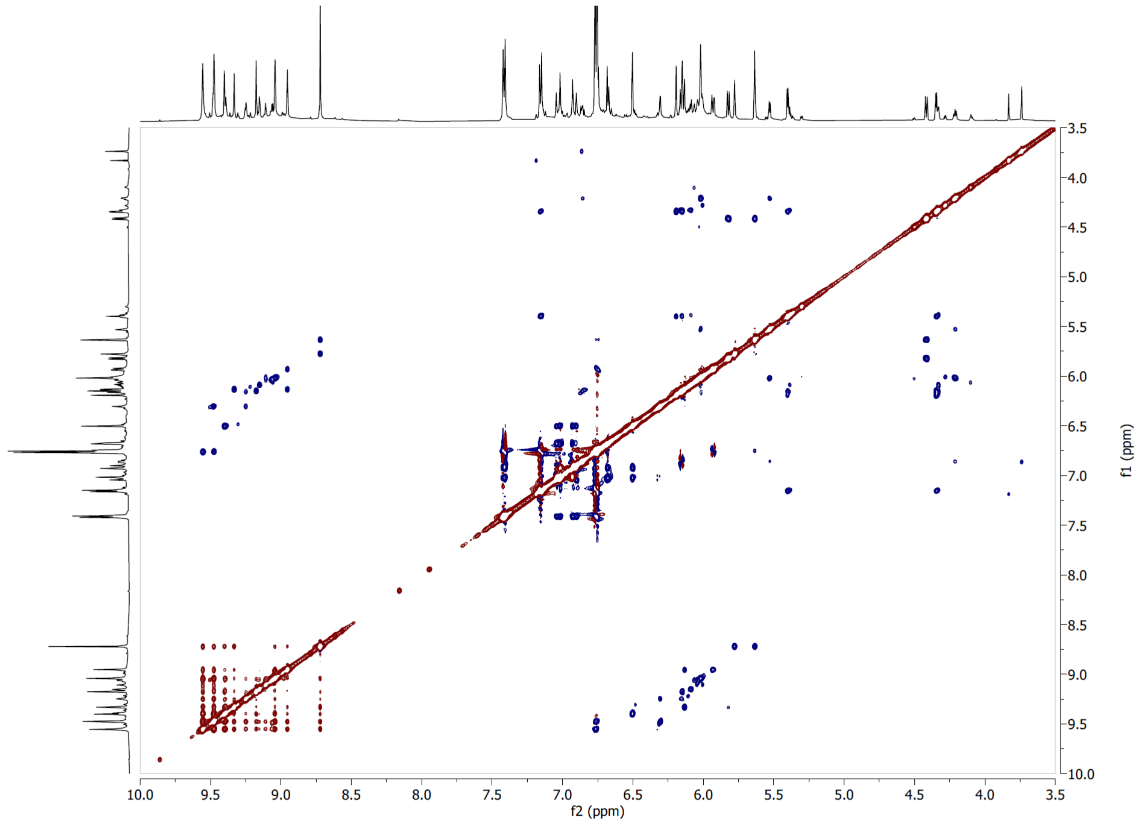

S4.9. ROESY NMR spectrum of Gnetoline A in DMSO-d_6.

5. Latifolol:

Experimental:

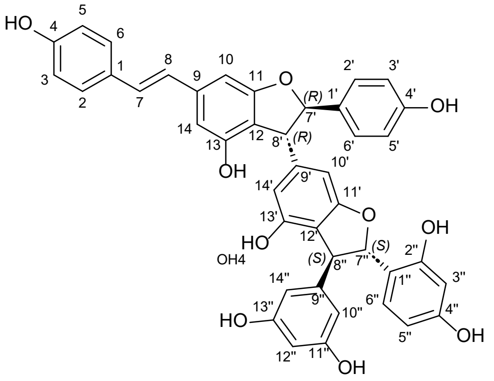

Latifolol (5) $[\alpha]_D^{20}$ -12.4 (c 0.12, MeOH), Literature[7] : $[\alpha]_D^{20}$ -42 (c 0.15, MeOH); UV (MeOH) λ_{max} (log ε) 226 (4.43), 287 (3.93), 310 (4.04), 328 (4.07), 347 (3.80) nm; ^{1}H NMR (DMSO-d_6, 600 MHz) δ 9.57 (1H, s, 4-OH or 4'-OH), 9.49 (2H, s, 2''-OH, 4-OH or 4'-OH), 9.40 (1H, s, 13-OH), 9.27 (1H, s, 4''-OH), 9.16 (1H, s, 11'-OH), 9.06 (2H, s, 11''-OH, 13''-OH), 7.42 (2H, d, *J* = 8.3 Hz, H-2, H-6), 7.16 (2H, d, *J* = 8.3 Hz, H-2', H-6'), 7.04 (1H, d, *J* = 16.2 Hz, H-7), 6.92 (1H, d, *J* = 16.2 Hz, H-8), 6.87 (1H, d, *J* = 8.5 Hz, H-6''), 6.77 (5H, d, *J* = 8.3 Hz, H-3, H-3', H-5, H-5'), 6.68 (1H, s, H-10), 6.52 (1H, s, H-14), 6.32 (1H, d, *J* = 1.8 Hz, H-3''), 6.17 (1H, dd, *J* = 8.5, 1.8 Hz, H-5''), 6.16 (1H, s, H-14'), 6.11 (1H, s, H-10'), 6.03 (3H, s, H-10'', H-12'', H-14''), 5.54 (1H, d, *J* = 4.2 Hz, H-7''), 5.39 (1H, d, *J* = 4.0 Hz, H-7'), 4.34 (1H, d, *J* = 4.0 Hz, H-8'), 4.23 (1H, d, *J* = 4.2 Hz, H-8''); ^{13}C NMR (DMSO, 151 MHz) δ 161.6 (C-11), 161.3 (C-13'), 158.1 (C-11'', C-13''), 158.0 (C-4''), 157.3 (C-4'), 157.3 (C-4), 155.6 (C-2''), 154.7 (C-13), 154.5 (C-11'), 145.6 (C-9''), 144.8 (C-9'), 139.8 (C-9), 132.2 (C-1'), 128.2 (CH-7), 128.2 (C-1), 127.9 (CH-3, CH-5), 127.1 (CH-6''), 126.8 (CH-2', CH-6'), 125.5 (CH-8), 118.3 (C-1''), 115.6 (CH-2, CH-6), 115.3 (CH-3', CH-5'), 113.9 (C-12), 113.8 (C-12'), 107.3 (CH-14), 106.9 (CH-10'), 106.1 (CH-5''), 105.6 (CH-10'', CH-14''), 102.5 (CH-3''), 100.7 (CH-12''), 99.5 (CH-14'), 97.8 (CH-10), 92.0 (CH-7'), 87.9 (CH-7''), 54.3 (CH-8'), 52.7 (CH-8''); (NP-MRD ID: NP0028328); HRESIMS *m/z* 697.2069 $[M+H]^+$ (calcd for $C_{42}H_{33}O_{10}^+$ 697.2068, Δ = 0.14 ppm), MS/MS spectrum: CCMSLIB00012475005, *m/z* 695.1917 $[M-H]^-$ (calcd for $C_{42}H_{31}O_{10}^-$ 695.1923, Δ = -0.86 ppm)

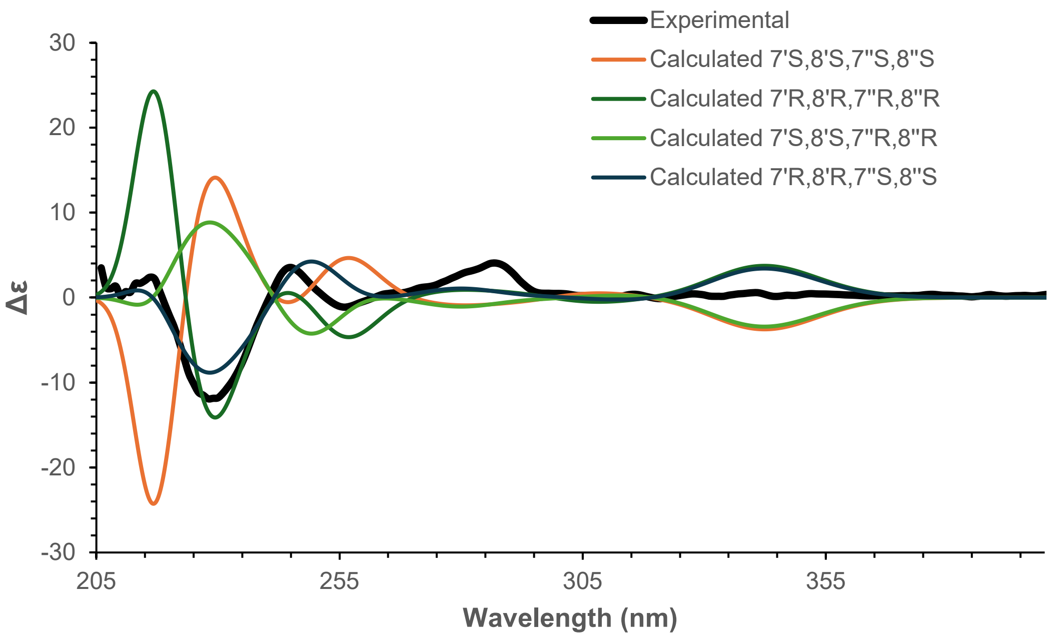

S5.1. ECD spectra (experimental and calculated) of Latifolol in MeOH.

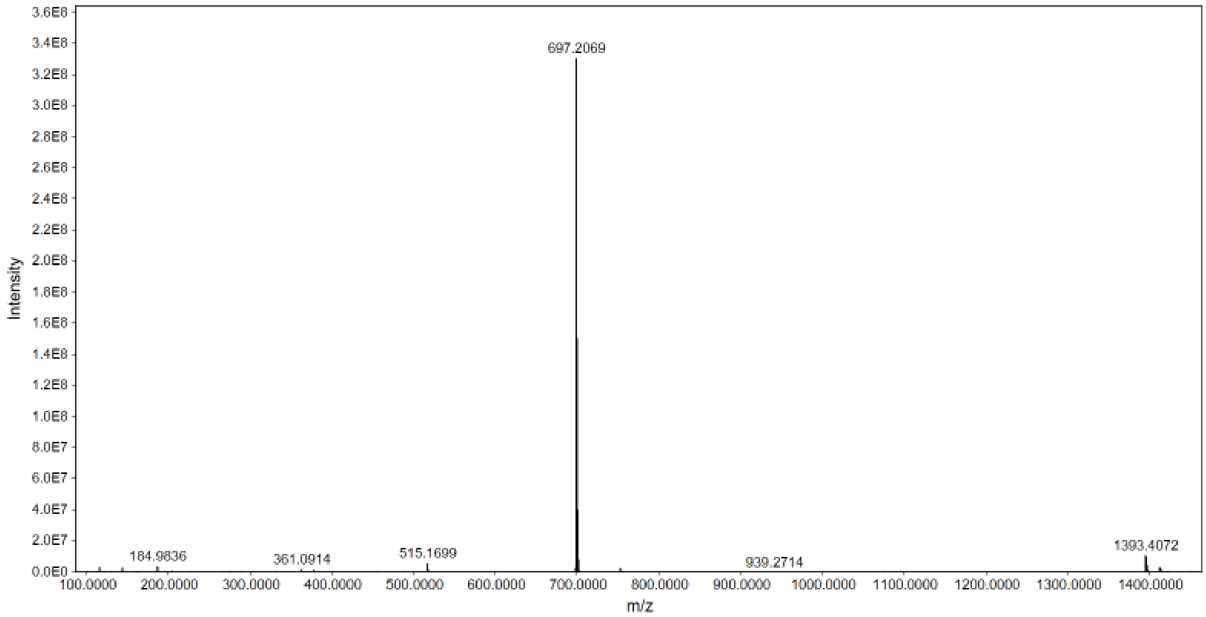

S5.2. HRESIMS+ spectrum of Latifolol in MeOH.

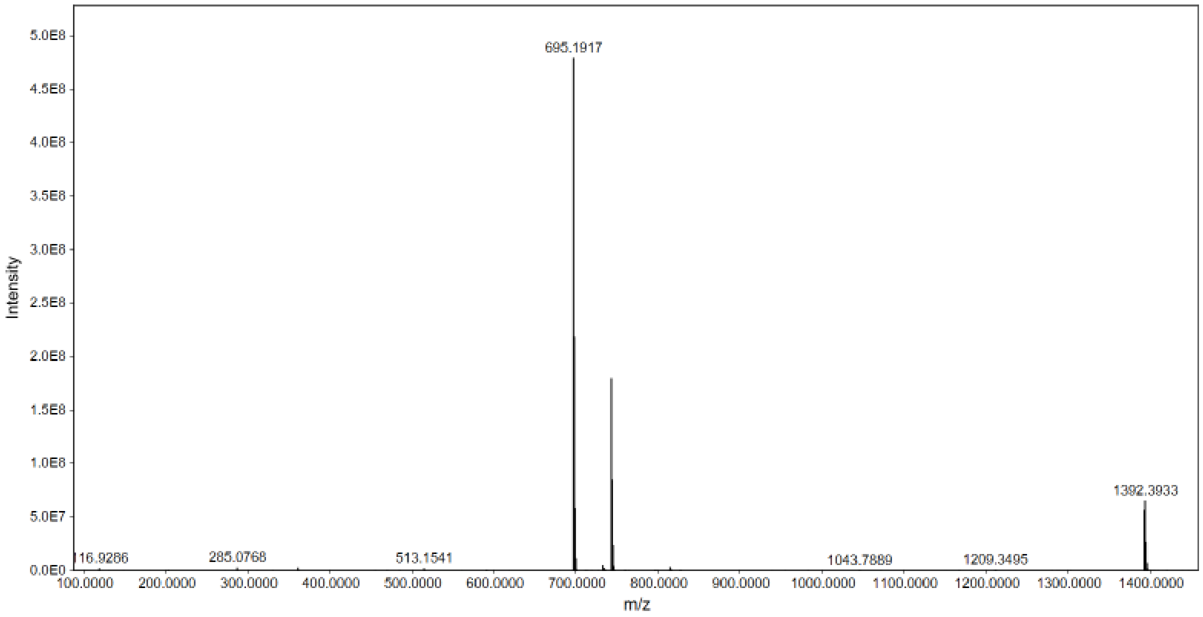

S5.3. HRESIMS- spectrum of Latifolol in MeOH.

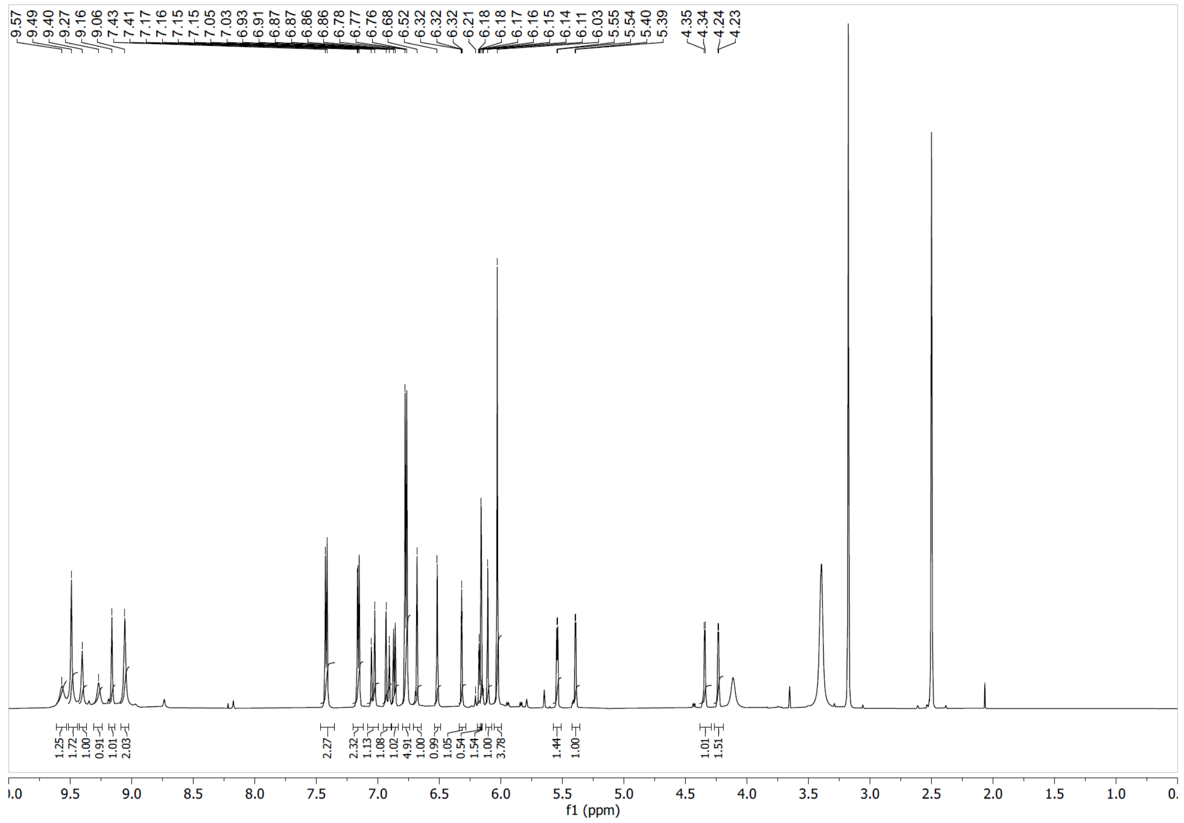

S5.4. ^{1}H NMR spectrum of Latifolol in DMSO-*d*$_6$ at 600 MHz.

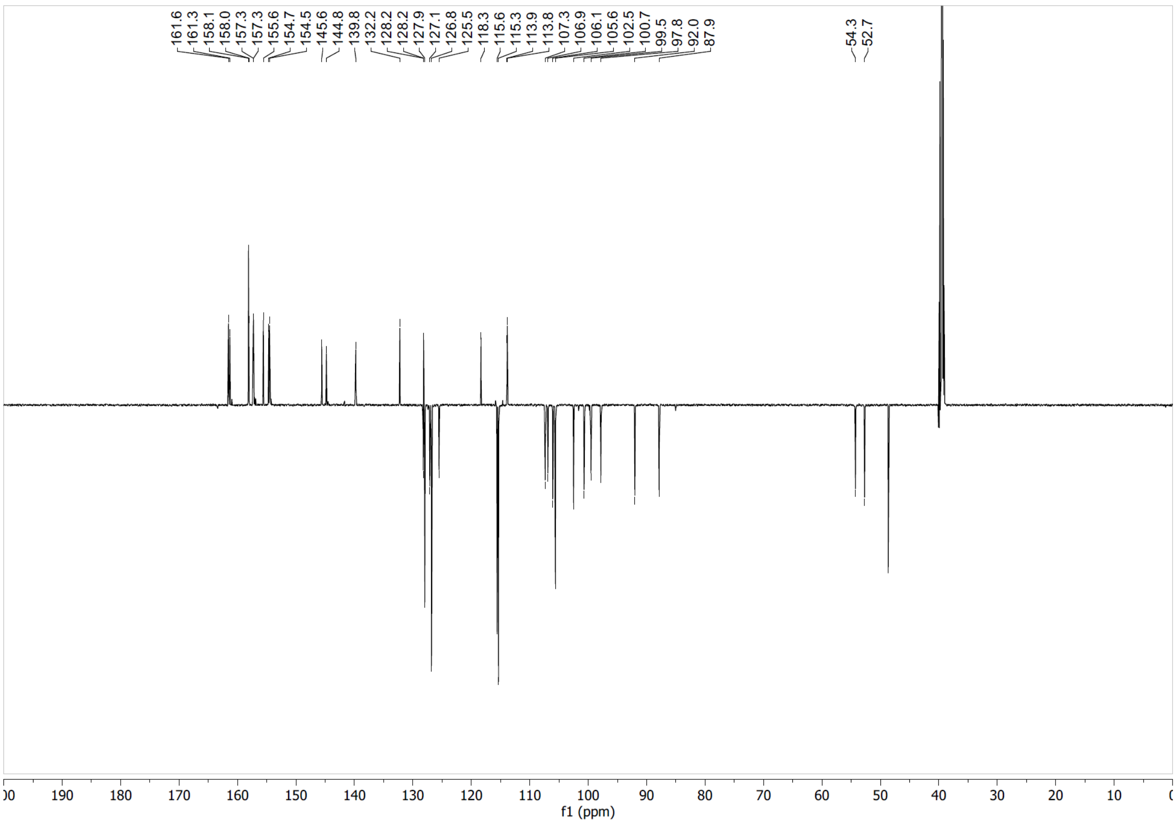

S5.5. ^{13}C NMR spectrum of Latifolol in DMSO-*d*$_6$ at 151 MHz.

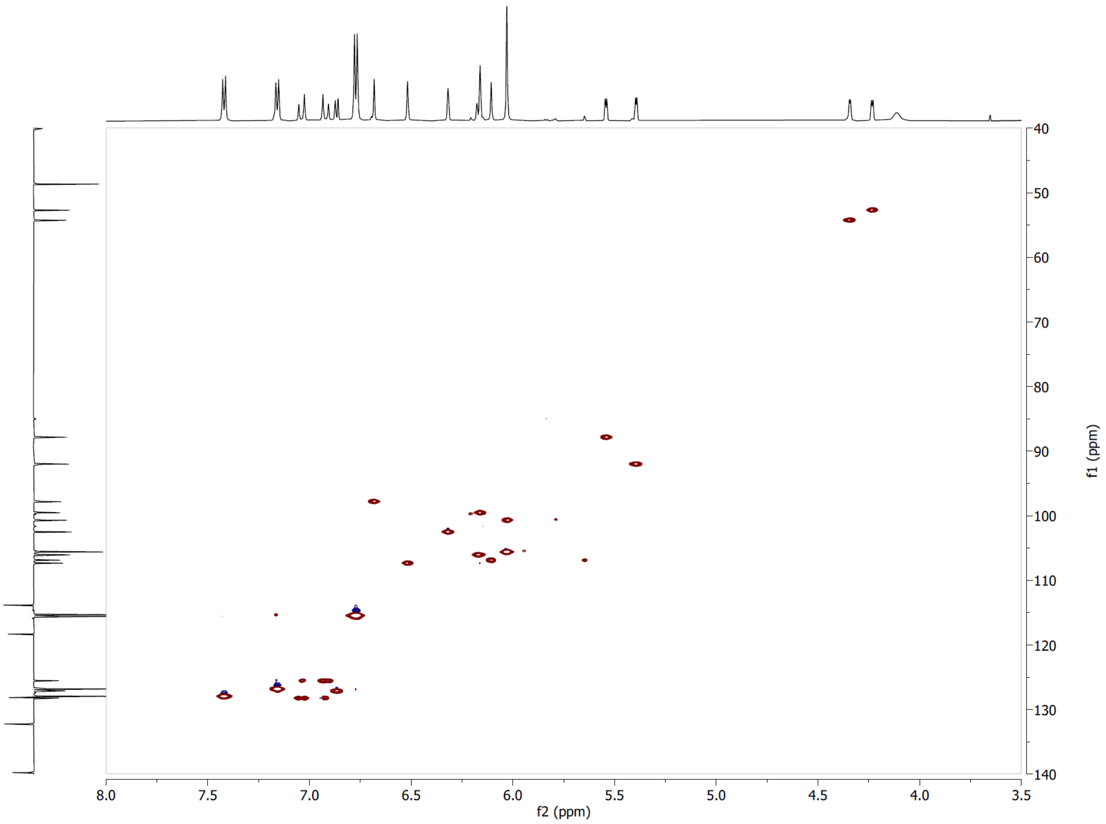

S5.6. HSQC NMR spectrum of Latifolol in DMSO-d_6.

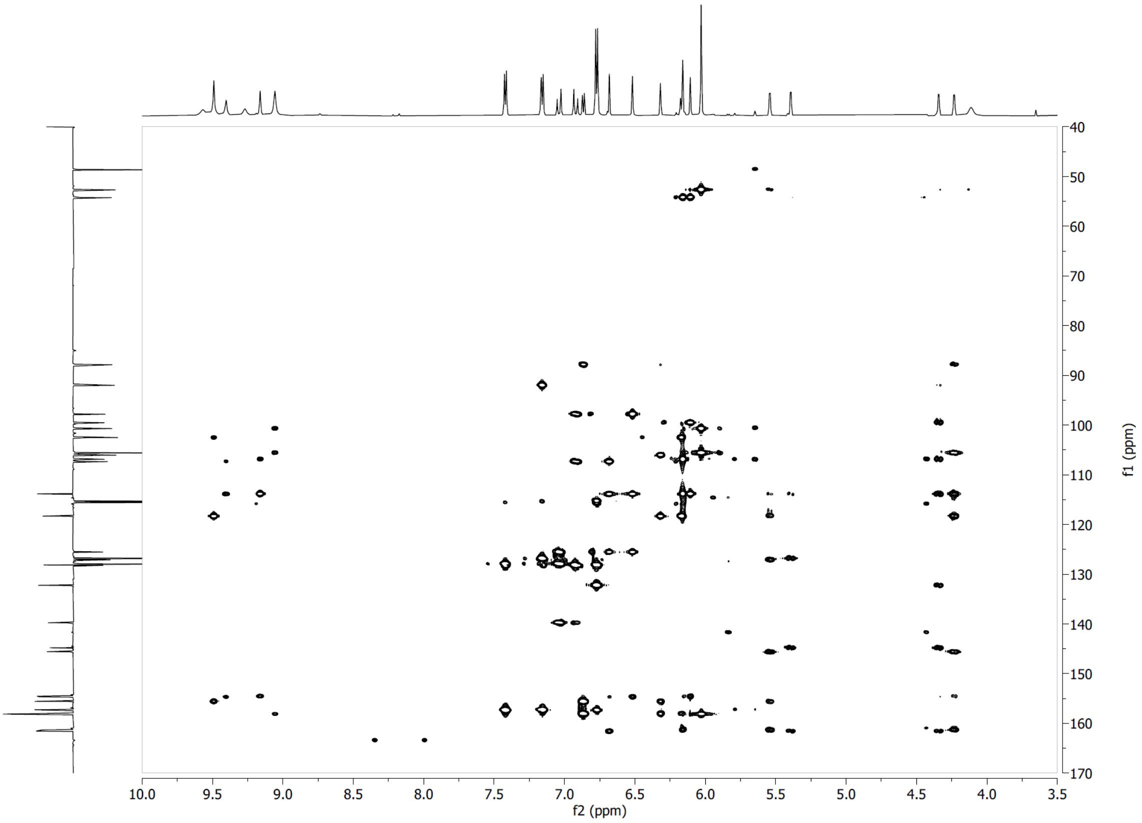

S5.7. HMBC NMR spectrum of Latifolol in DMSO-d_6.

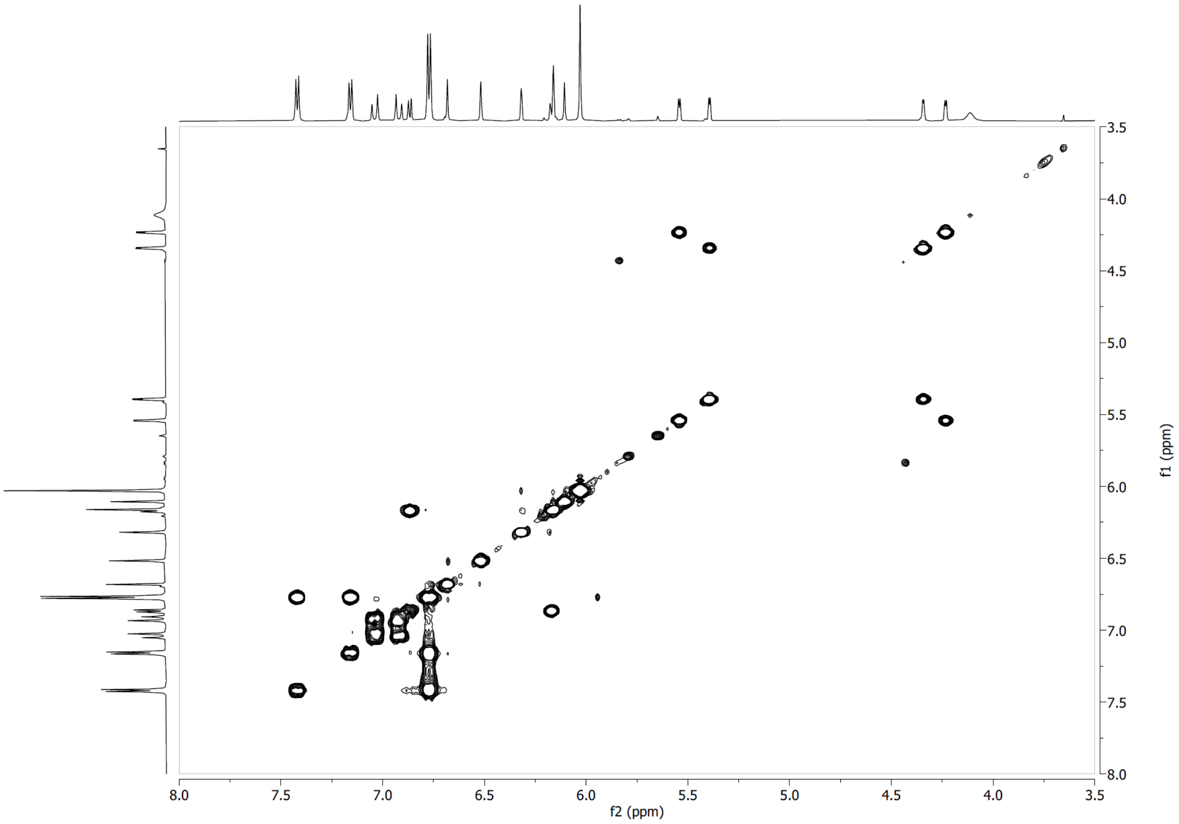

S5.8. COSY NMR spectrum of Latifolol in DMSO-*d*6.

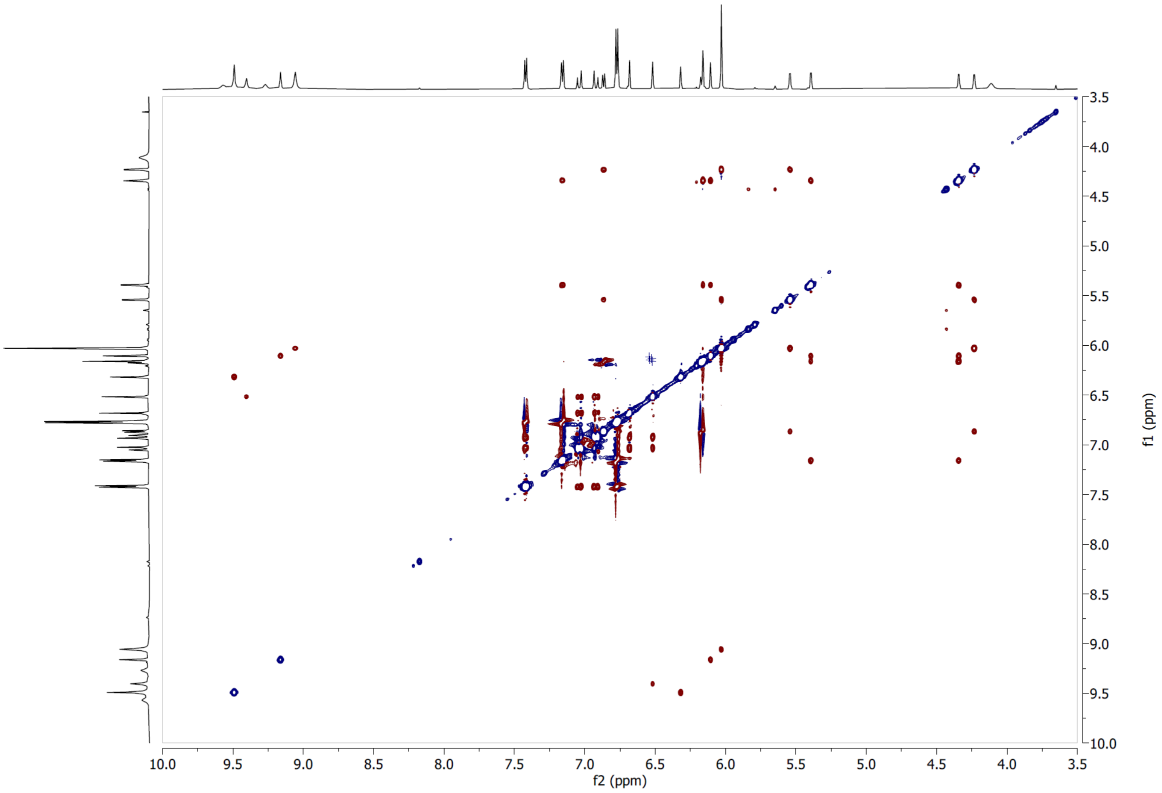

S5.9. ROESY NMR spectrum of Latifolol in DMSO-*d*6.

6. (-)-Gnetin E:

Experimental:

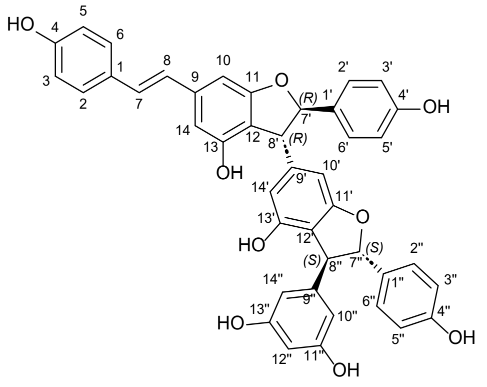

(-)-Gnetin E (6) $[\alpha]_D^{20}$ -2.06 (c 0.13, MeOH); UV (MeOH) λ_{max} (log ε) 226 (4.45), 287 (3.91), 310 (4.03), 328 (4.04), 347 (3.78) nm; ^{1}H NMR (DMSO-d_6, 600 MHz) δ 9.56 and 9.49 (1H and 2H, s, 4-OH, 4-OH, 4''-OH), 9.40 (1H, s, 13-OH), 9.23 (1H, s, 13'-OH), 9.10 (2H, s, 11''-OH, 13''-OH), 7.41 (2H, d, *J* = 8.7 Hz, H-2, H-6), 7.15 (2H, d, *J* = 8.6 Hz, H-2', H-6'), 7.10 (2H, d, *J* = 8.6 Hz, H-2'', H-6''), 7.03 (1H, d, *J* = 16.3 Hz, H-7), 6.91 (1H, d, *J* = 16.3 Hz, H-8), 6.76 (6H, m, H-3, H-3', H-3'', H-5, H-5', H-5''), 6.68 (1H, d, *J* = 0.9 Hz, H-10), 6.50 (1H, d, *J* = 0.9 Hz, H-14), 6.16 (1H, d, *J* = 1.3 Hz, H-10'), 6.13 (1H, d, *J* = 1.3 Hz, H-14'), 6.03 (1H, t, *J* = 2.2 Hz, H-12''), 5.97 (2H, d, *J* = 2.2 Hz, H-10'', H-14''), 5.40 (1H, d, *J* = 4.0 Hz, H-7'), 5.27 (1H, d, *J* = 5.7 Hz, H-7''), 4.34 (1H, d, *J* = 4.0 Hz, H-8'), 4.22 (1H, d, *J* = 5.7 Hz, H-8''); ^{13}C NMR (DMSO-d_6, 151 MHz) δ 161.5 (C-11), 161.0 (C-11'), 158.3 (C-11'', C-13''), 157.3 (C-4, C-4'), 157.2 (C-4''), 154.6 (C-13), 154.5 (C-13'), 145.1 (C-9'), 144.7 (C-9''), 139.7 (C-9), 132.2 (C-1'), 131.7 (C-1''), 128.2 (CH-7), 128.1 (C-1), 127.9 (CH-2, CH-6), 127.2 (CH-2'', CH-6''), 126.8 (CH-2', CH-6'), 125.5 (CH-8), 115.5 (CH-3, CH-5), 115.3 (CH-3'', CH-5'', CH-3', CH-5'), 113.9 (C-12), 113.1 (C-12'), 107.3 (CH-14), 107.1 (CH-14'), 105.4 (CH-10'', CH-14''), 100.9 (CH-12''), 99.4 (CH-10'), 97.8 (CH-10), 92.4 (CH-7''), 91.9 (CH-7'), 54.3 (CH-8''), 54.2 (CH-8'); (NP-MRD ID: NP0332864); HRESIMS *m/z* 681.2120 $[M+H]^+$ (calcd for $C_{42}H_{33}O_9^+$ 681.2119, Δ = 0.15 ppm), MS/MS spectrum: CCMSLIB00012474987, *m/z* 679.1970 $[M-H]^-$ (calcd for $C_{42}H_{31}O_9^-$ 679.1974, Δ = -0.59 ppm).

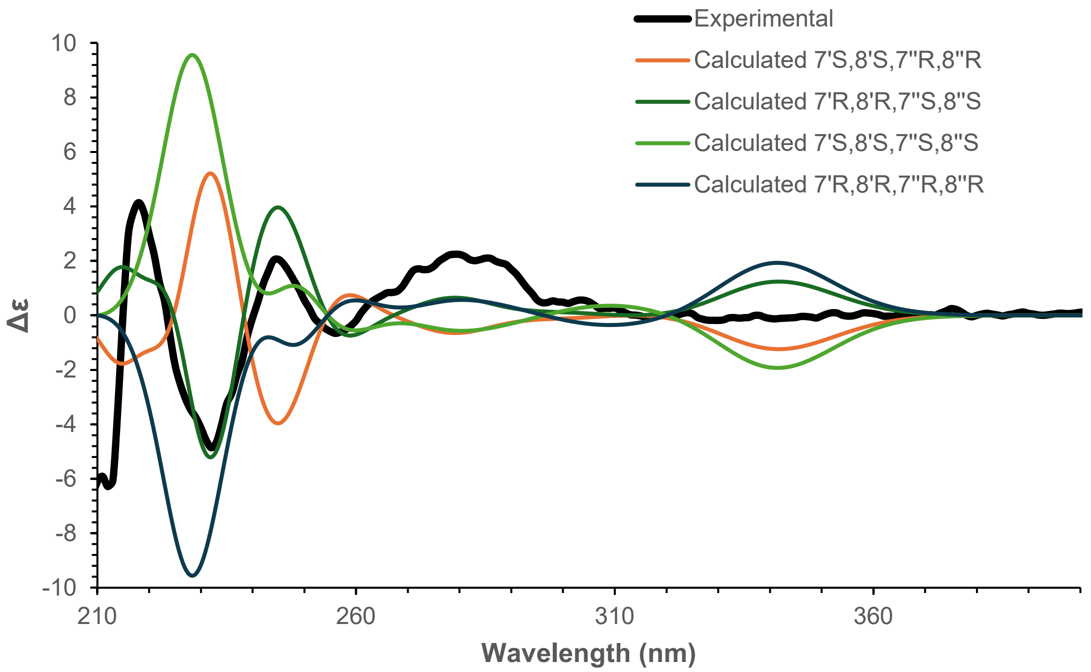

S6.1. ECD spectra (experimental and calculated) of (-)-Gnetin E in MeOH.

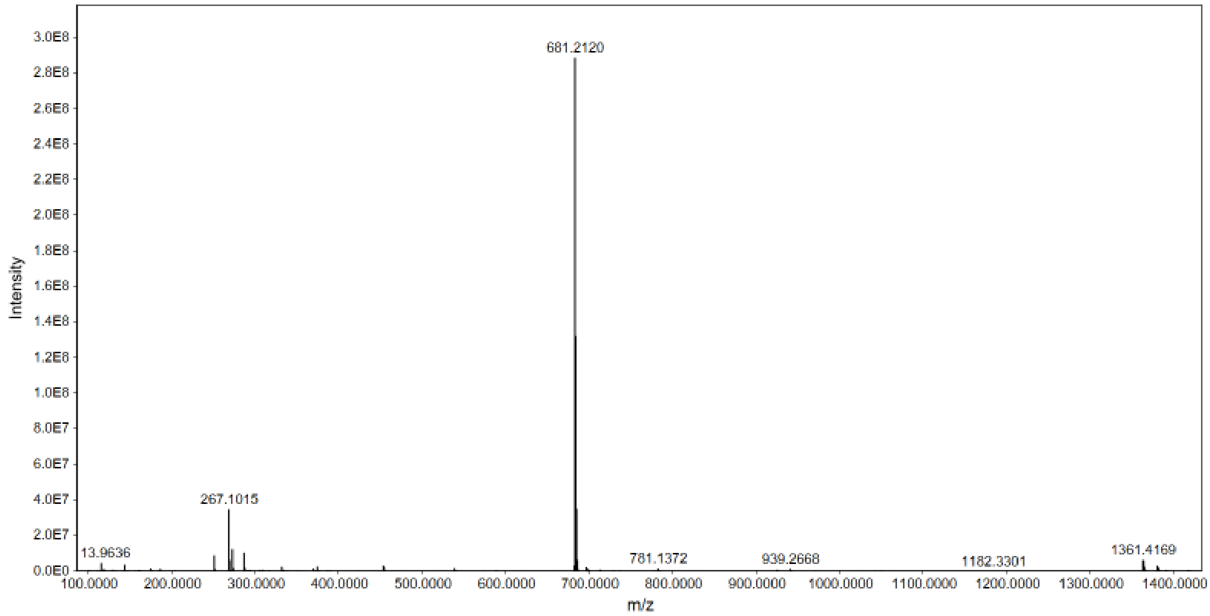

S6.2. HRESIMS+ spectrum of (-)-Gnetin E in MeOH.

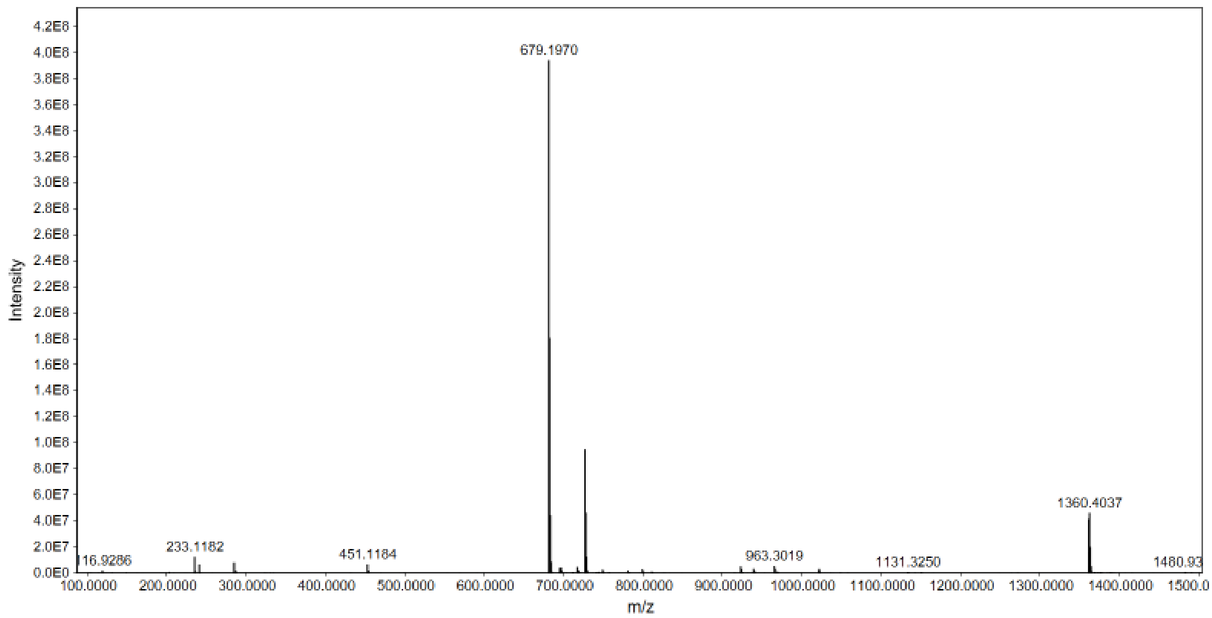

S6.3. HRESIMS- spectrum of (-)-Gnetin E in MeOH.

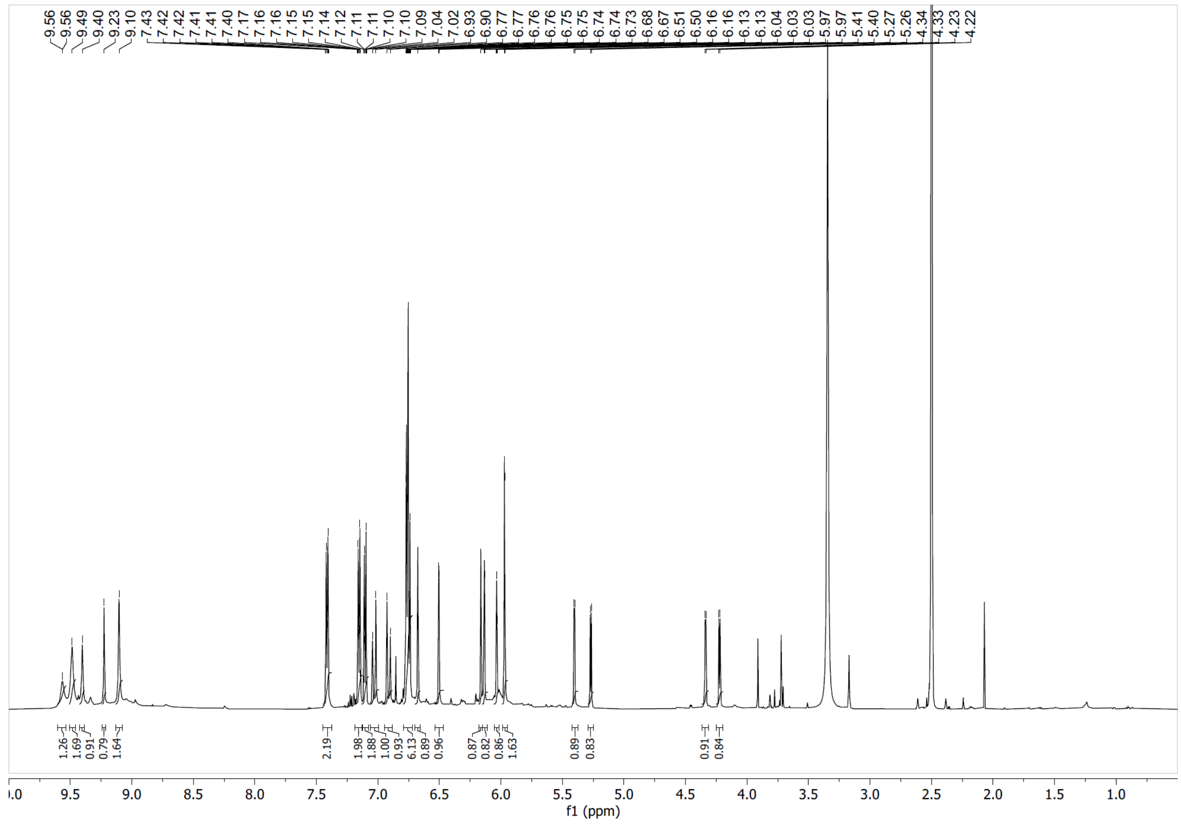

S6.4. ^{1}H NMR spectrum of (-)-Gnetin E in DMSO-d_6 at 600 MHz.

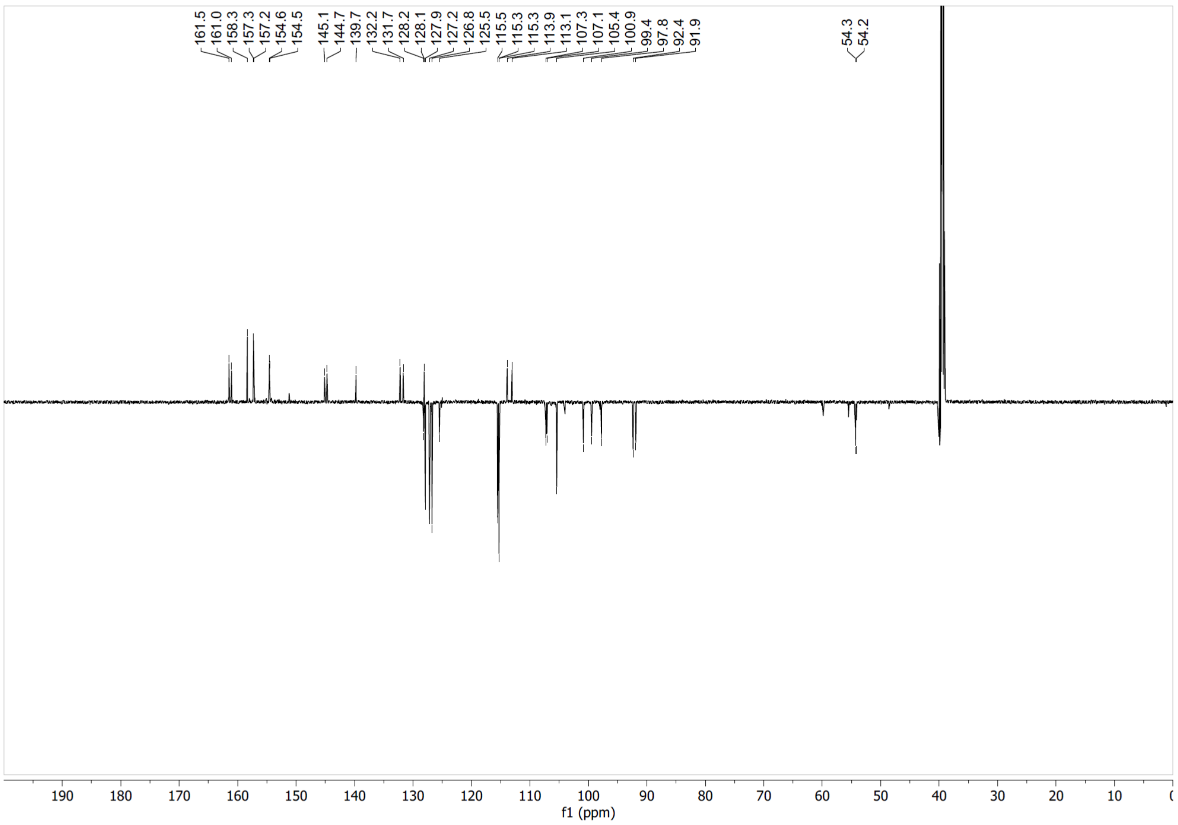

S6.5. ^{13}C NMR spectrum of (-)-Gnetin E in DMSO-d_6 at 151 MHz.

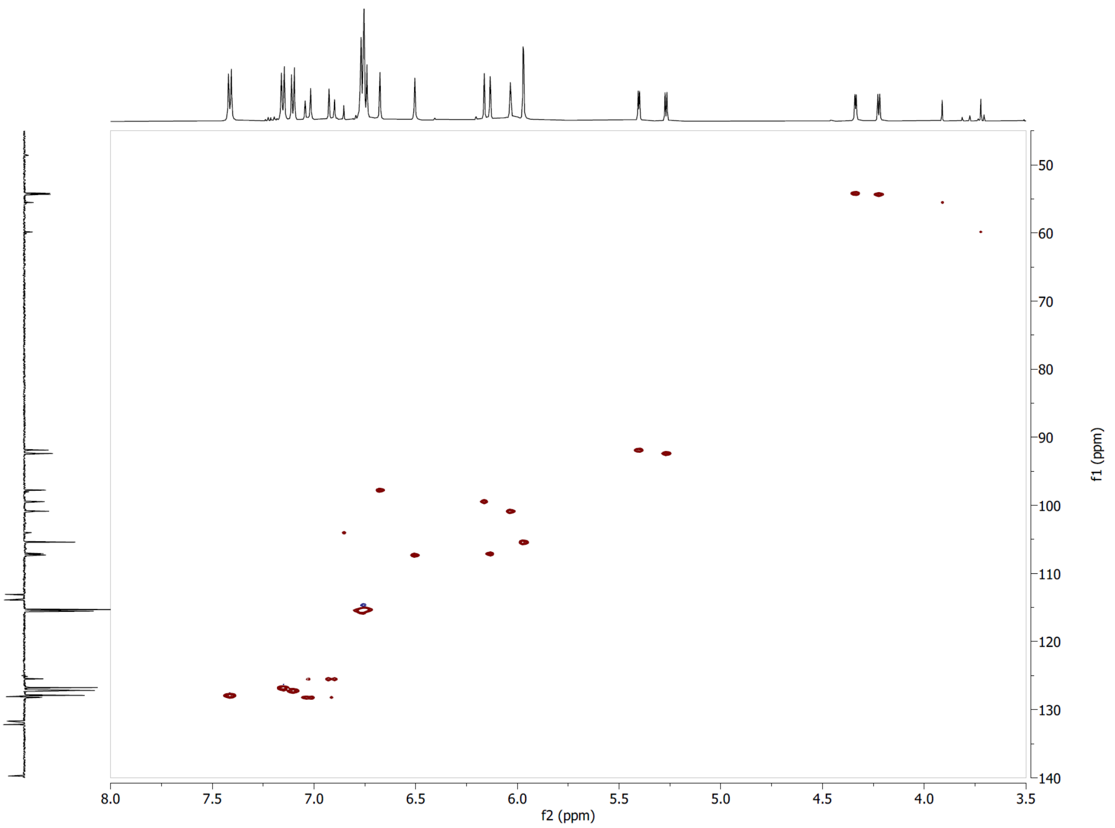

S6.6. HSQC NMR spectrum of (-)-Gnetin E in DMSO-d_6.

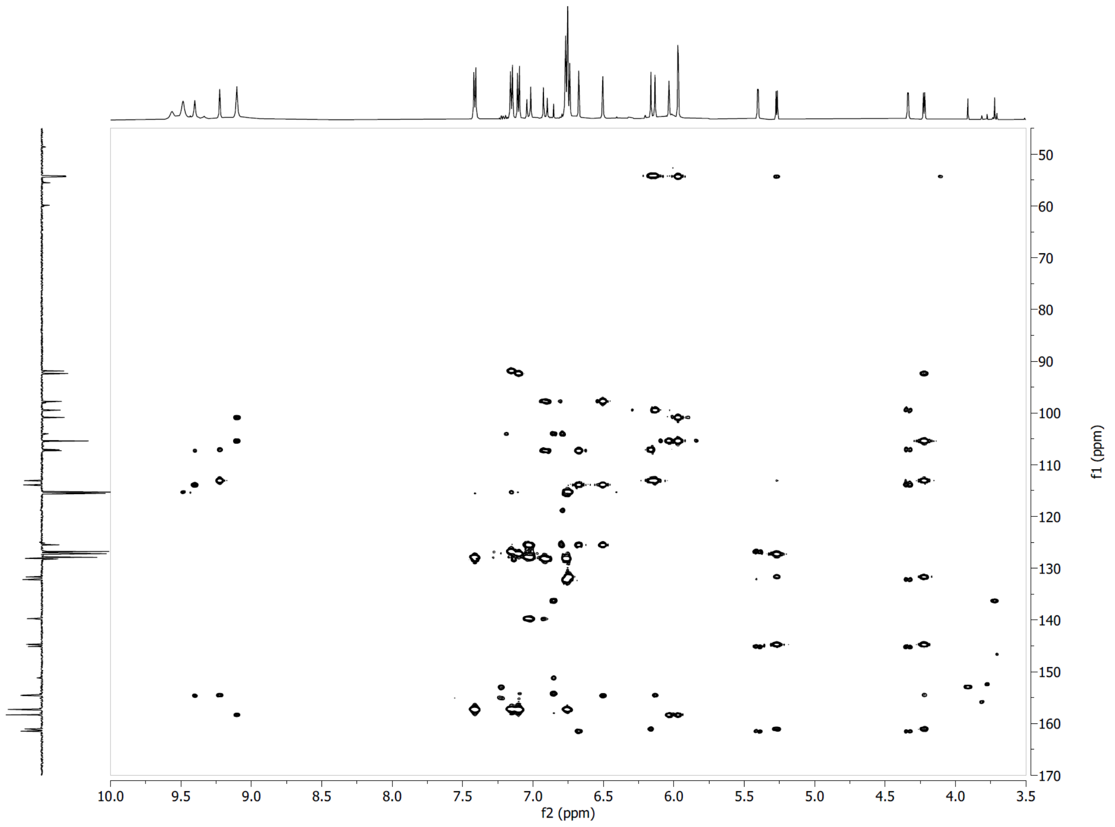

S6.7. HMBC NMR spectrum of (-)-Gnetin E in DMSO-d_6.

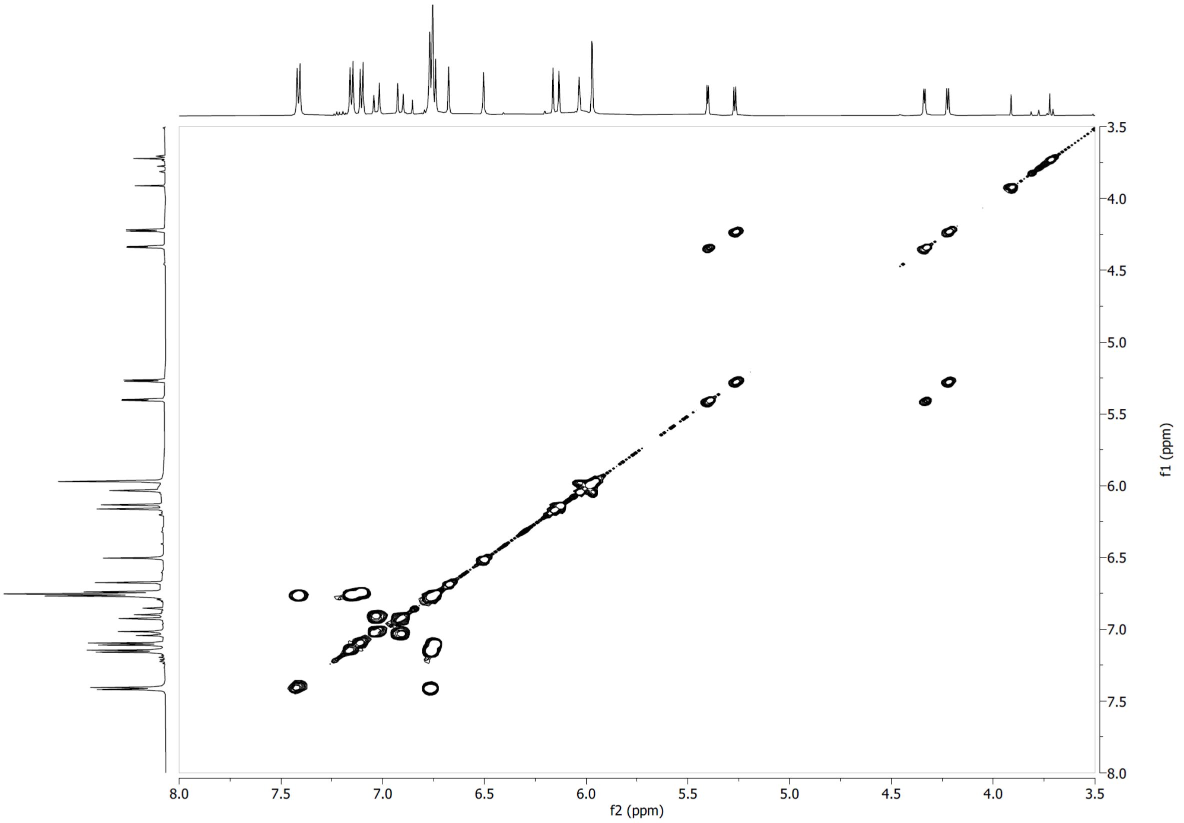

S6.8. COSY NMR spectrum of (-)-Gnetin E in DMSO-d_6.

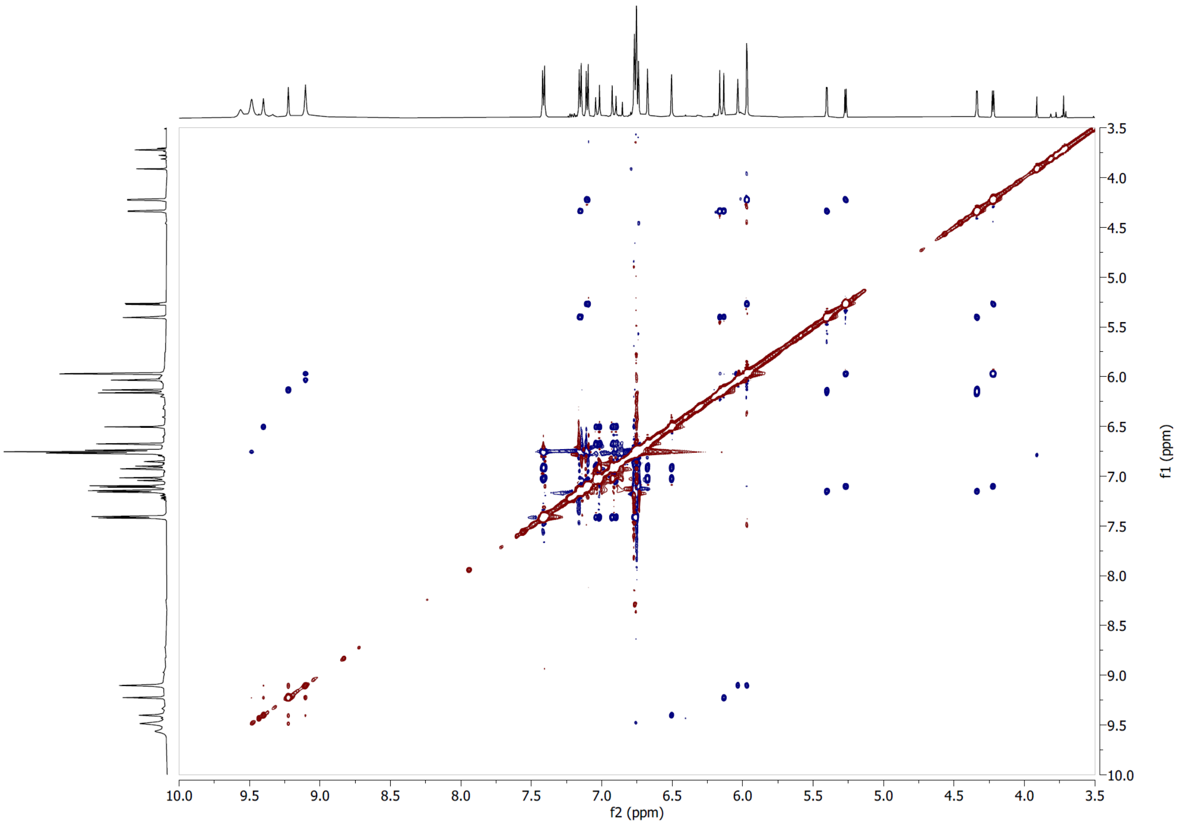

S6.9. ROESY NMR spectrum of (-)-Gnetin E in DMSO-d_6.

7. Macrostachyol A:

Experimental:

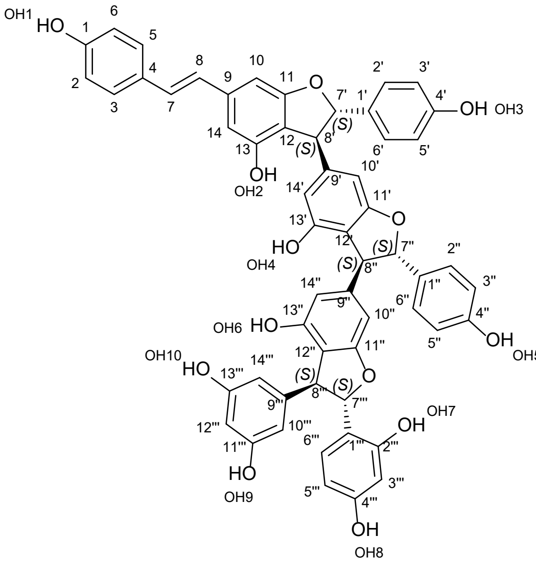

Macrostachyol A (+enantiomer?) (7) $[\alpha]_D^{20}$ -3.87 (c 0.12, MeOH), Literature[8] : $[\alpha]_D^{26}$ -29 (c 0.10, MeOH); UV (MeOH) λ_{max} (log ε) 226 (4.59), 287 (4.01), 310 (4.07), 328 (4.10), 347 (3.84) nm; ^{1}H NMR (DMSO-d_6, 600 MHz) δ 9.56 (1H, s, 4-OH), 9.49 (2H, s, 4'-OH, 4''-OH), 9.48 (1H, s, 2'''-OH), 9.40 (1H, s, 13-OH), 9.31 (1H, s, 13'-OH), 9.26 (1H, s, 4'''-OH), 9.16 (1H, s, 13''-OH), 9.04 (2H, s, 11'''-OH, 13'''-OH), 7.41 (2H, d, J = 8.4 Hz, H-2, H-6), 7.16 (2H, d, J = 8.4 Hz, H-2', H-6'), 7.13 (2H, d, J = 8.3 Hz, H-2'', H-6''), 7.03 (1H, d, J = 16.3 Hz, H-7), 6.91 (1H, d, J = 16.3 Hz, H-8), 6.85 (1H, d, J = 8.4 Hz, H-6'''), 6.76 (6H, m, H-3, H-3', H-3'', H-5, H-5', H-5''), 6.68 (1H, s, H-10), 6.50 (1H, s, H-14), 6.31 (1H, d, J = 2.3 Hz, H-3'''), 6.18 (1H, s, H-10'), 6.16 (1H, s, H-14'), 6.16 (1H, dd, J = 8.4, 2.3 Hz, H-5'''), 6.11 (1H, s, H-14''), 6.09 (1H, s, H-10''), 6.01 (3H, s, H-10''', H-12''', H-14'''), 5.51 (1H, d, J = 4.6 Hz, H-7'''), 5.41 (1H, d, J = 4.0 Hz, H-7'), 5.35 (1H, d, J = 5.0 Hz, H-7''), 4.35 (1H, d, J = 4.0 Hz, H-8'), 4.32 (1H, d, J = 5.0 Hz, H-8''), 4.23 (1H, d, J = 4.6 Hz, H-8'''); ^{13}C NMR (DMSO-d_6, 151 MHz) δ 161.5 (C-11), 161.1 (C-11''), 161.1 (C-11'), 158.1 (C-11''', C-13'''), 158.0 (C-4'''), 157.3 (C-4', C-4''), 157.2 (C-4), 155.6 (C-2'''), 154.6 (C-13), 154.5 (C-13'), 154.5 (C-13''), 145.5 (C-9'''), 145.2 (C-9'), 144.5 (C-9''), 139.8 (C-9), 132.2 (C-1'), 131.8 (C-1''), 128.2 (CH-7), 128.1 (C-1), 127.9 (CH-2, CH-6), 127.2 (CH-6'''), 127.1 (CH-2'', CH-6''), 126.8 (CH-2', CH-6'), 125.5 (CH-8), 118.2 (C-1'''), 115.5 (CH-3, CH-5), 115.3 (CH-3', CH-5'), 115.3 (CH-3'', CH-5''), 113.9 (C-12), 113.7 (C-12''), 113.0 (C-12'), 107.3 (CH-14), 107.2 (CH-14'), 106.9 (CH-14''), 106.0 (CH-5'''), 105.6 (CH-10''', CH-14'''), 102.5 (CH-3'''), 100.7 (CH-12'''), 99.7 (CH-10''), 99.6 (CH-10'), 97.8 (CH-10), 92.3 (CH-7''), 91.9 (CH-7'), 87.8 (CH-7'''), 54.2 (CH-8', CH-8''), 52.6 (CH-8'''); (NP-MRD ID: NP0332865); HRESIMS m/z 923.2700 $[M+H]^+$ (calcd for $C_{56}H_{43}O_{13}^+$ 923.2698, Δ = 0.22 ppm), MS/MS spectrum: CCMSLIB00012475007, m/z 921.2527 $[M-H]^-$ (calcd for $C_{56}H_{41}O_{13}^-$ 921.2553, Δ = -2.82 ppm).

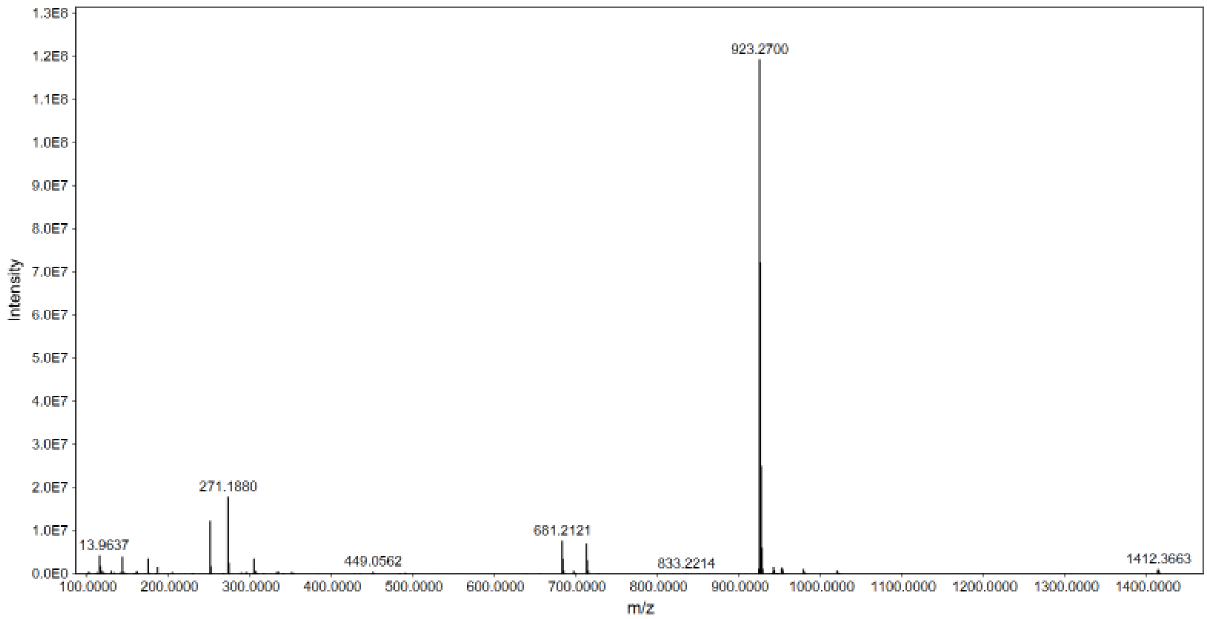

S7.1. HRESIMS+ spectrum of Macrostachyol A in MeOH.

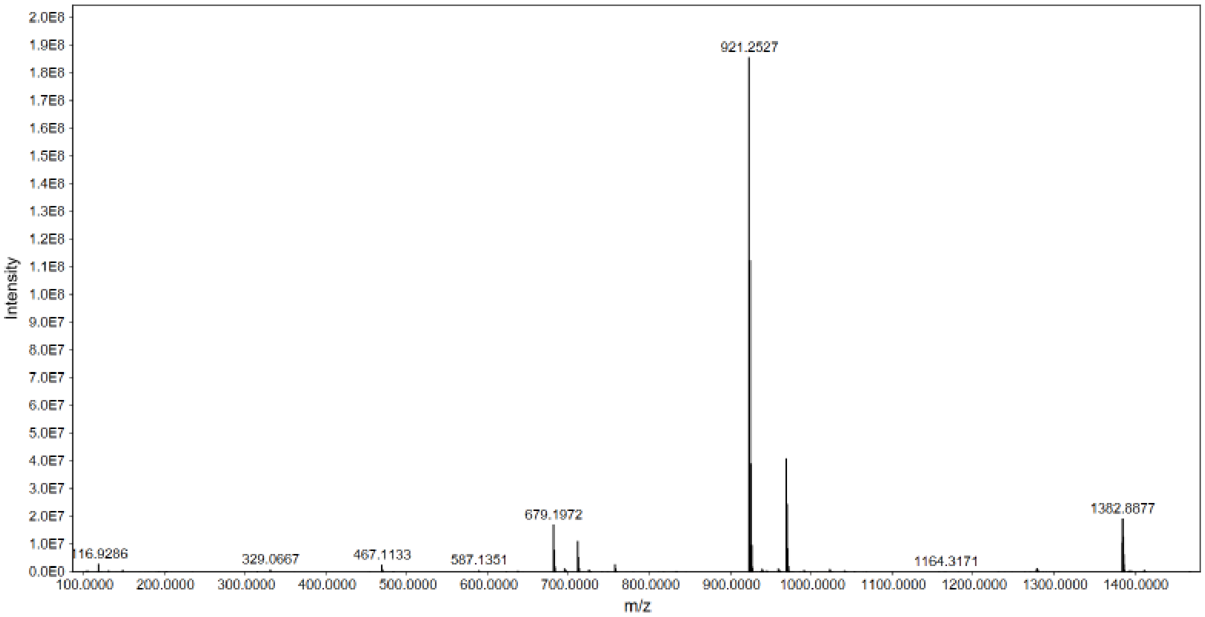

S7.2. HRESIMS- spectrum of Macrostachyol A in MeOH.

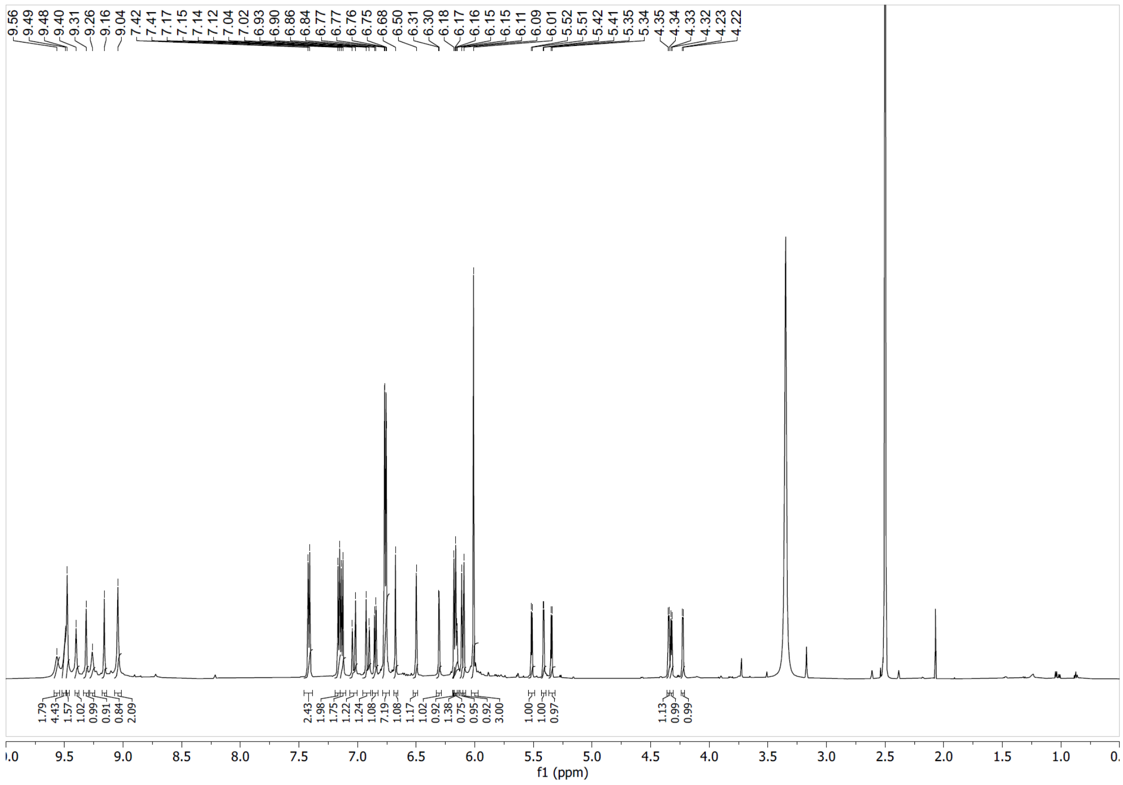

S7.3. ^{1}H NMR spectrum of Macrostachyol A in DMSO-d_6 at 600 MHz.

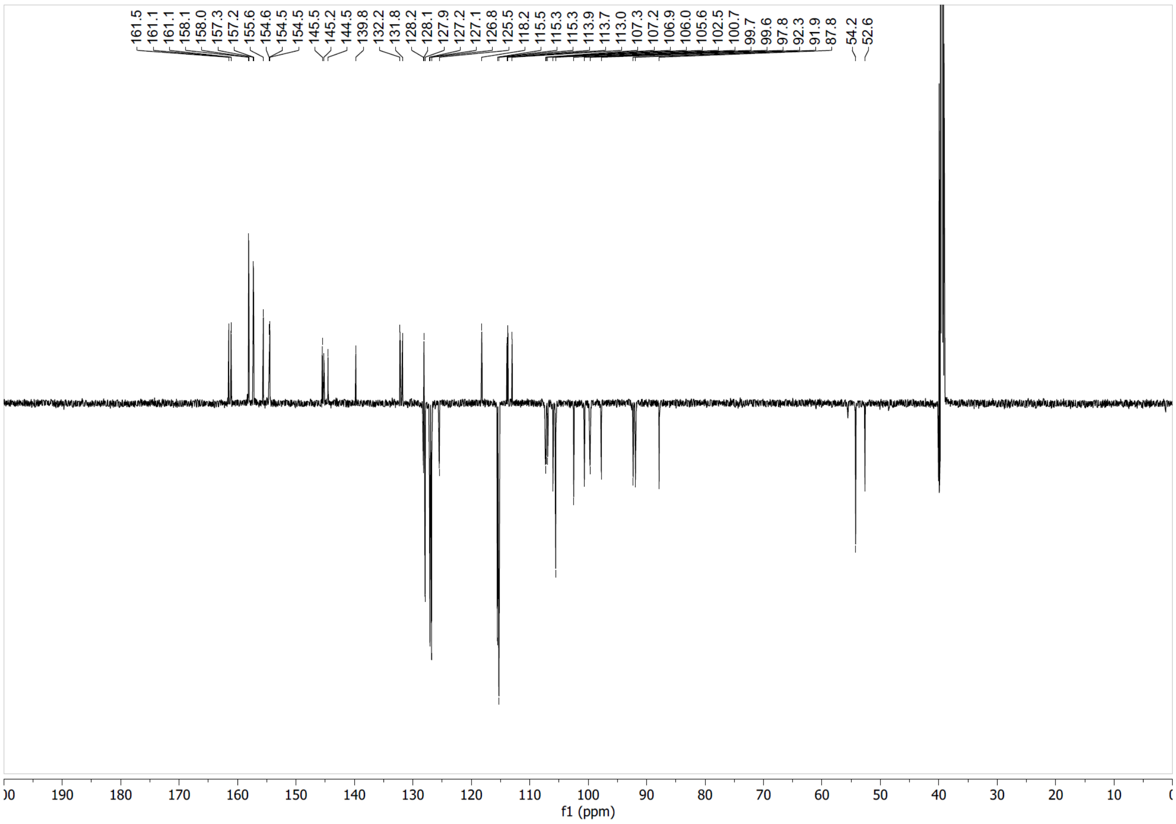

S7.4. ^{13}C NMR spectrum of Macrostachyol A in DMSO-d_6 at 151 MHz.

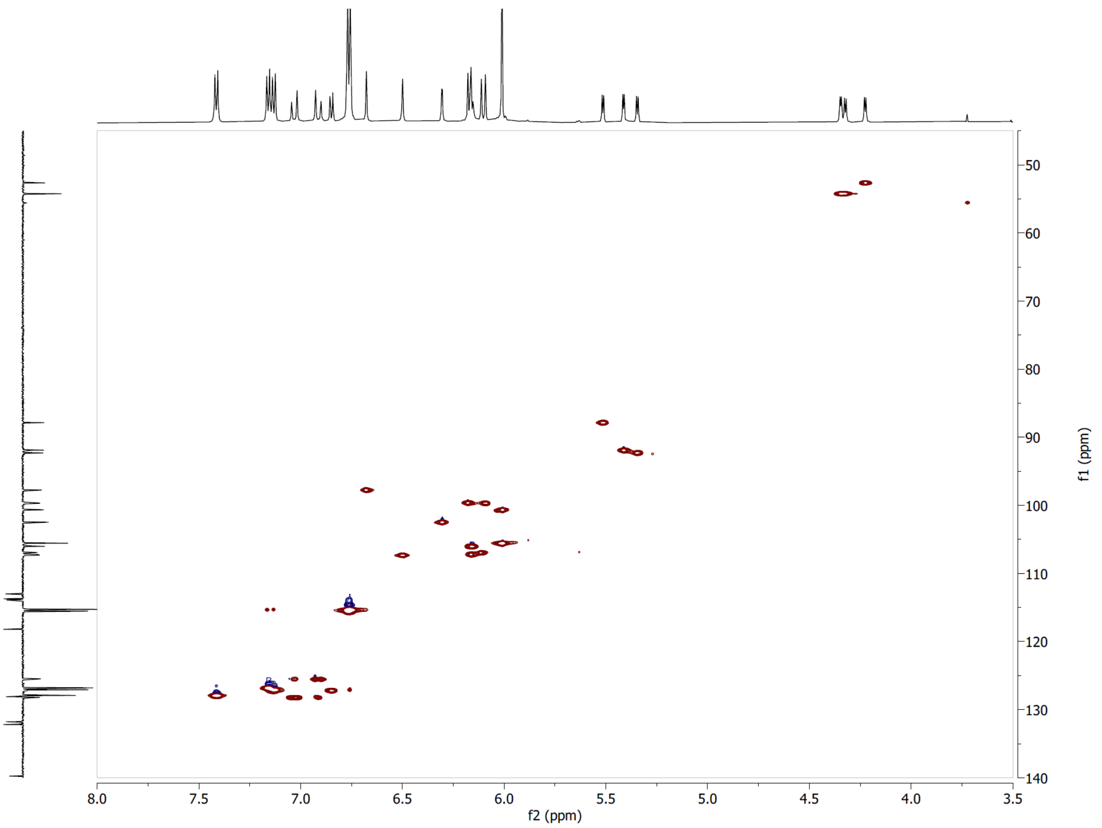

S7.5. HSQC NMR spectrum of Macrostachyol A in DMSO-*d*6.

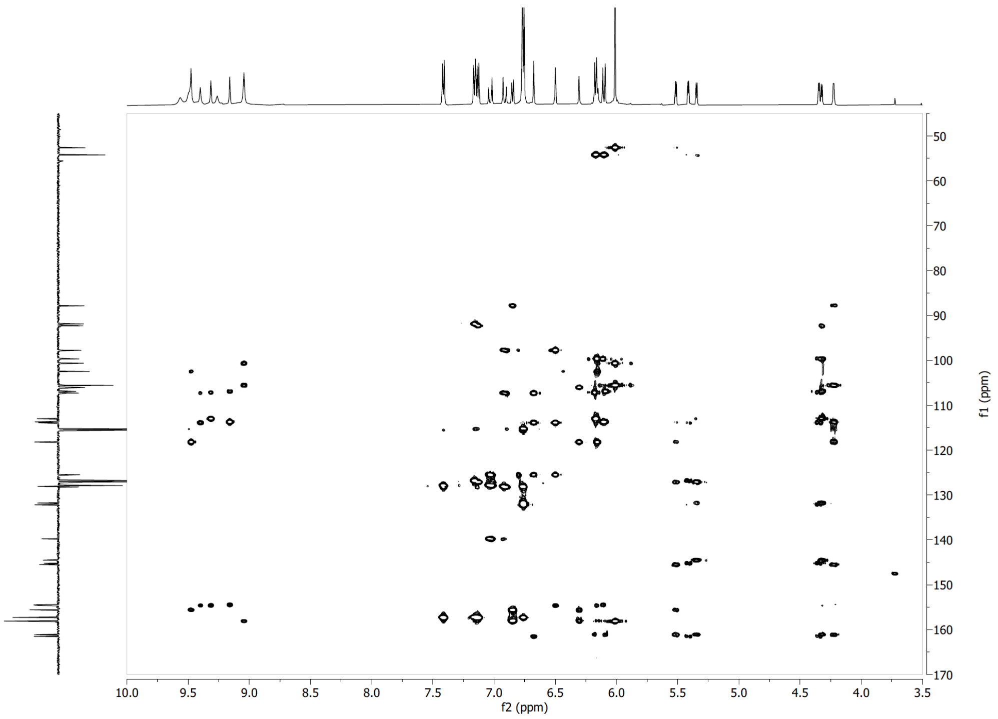

S7.6. HMBC NMR spectrum of Macrostachyol A in DMSO-*d*6.

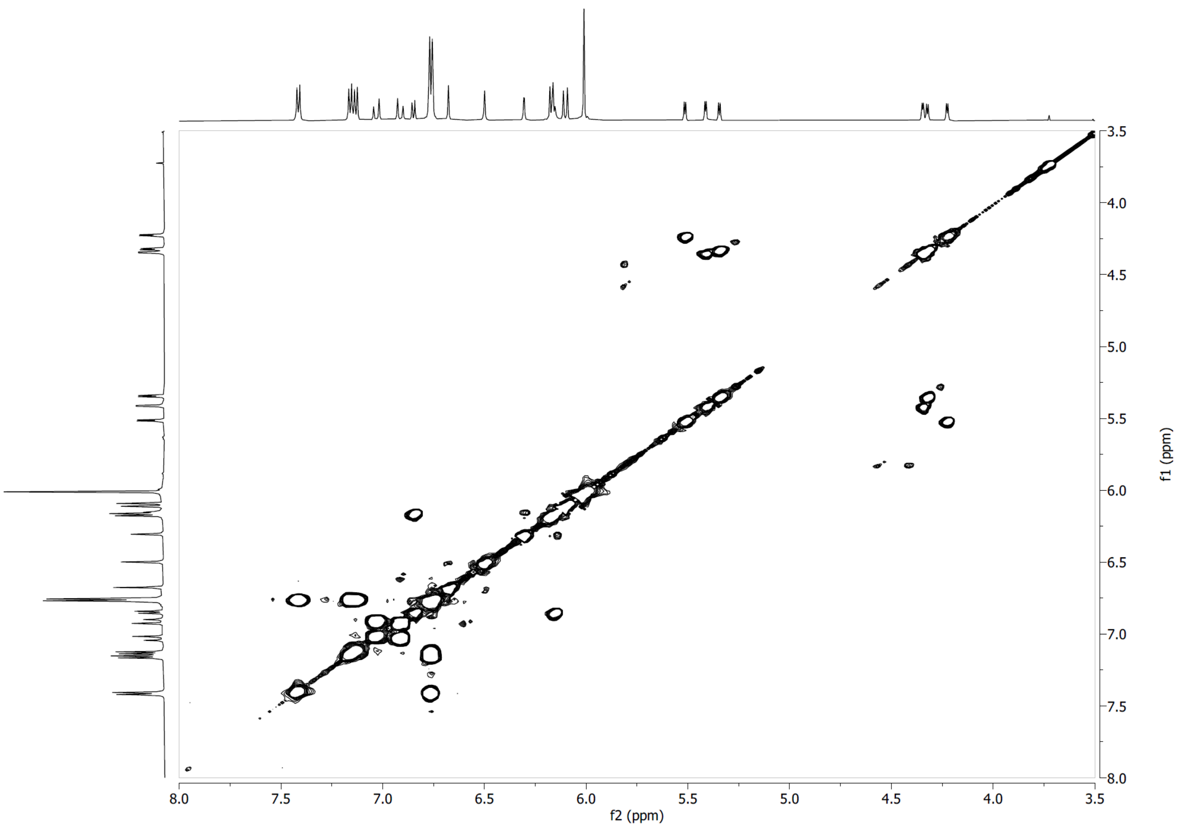

S7.7. COSY NMR spectrum of Macrostachyol A in DMSO-*d*$_6$.

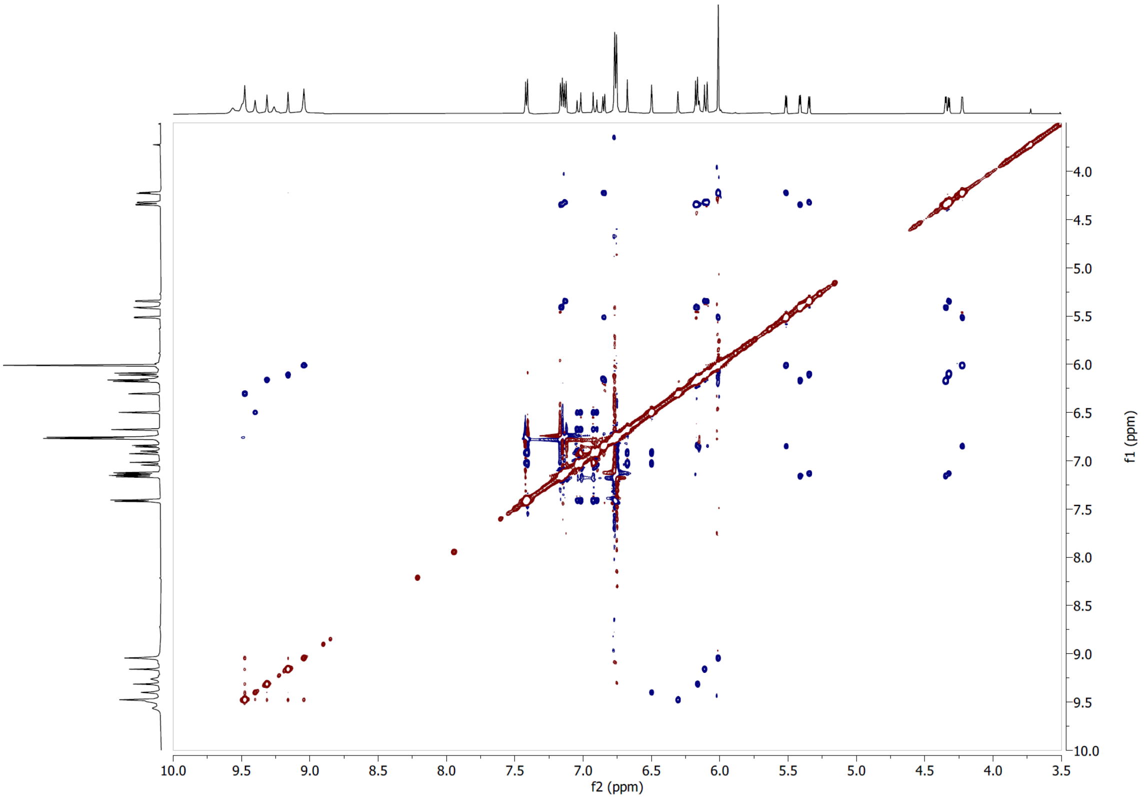

S7.8. ROESY NMR spectrum of Macrostachyol A in DMSO-*d*$_6$.

8. Gnemonol B:

Experimental:

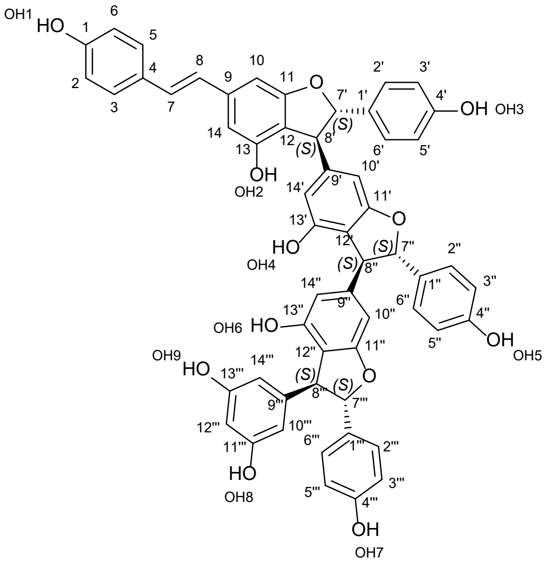

Gnemonol B (8) $[\alpha]_D^{20}$ -6.18 (c 0.10, MeOH), Literature[9] : $[\alpha]_D^{20}$ +8 (c 0.34, MeOH); UV (MeOH) λ_{max} (log ε) 226 (4.58), 287 (4.04), 310 (4.09), 328 (4.10), 347 (3.90) nm; ^{1}H NMR (DMSO-d_6, 600 MHz) δ 9.39 (1H, s, 13-OH), 9.31 (1H, s, 13'-OH), 9.23 (1H, s, 13"-OH), 9.09 (2H, s, 11'''-OH, 13'''-OH), 7.41 (2H, d, *J* = 8.6 Hz, H-2, H-6), 7.15 (2H, d, *J* = 8.7 Hz, H-2', H-6'), 7.13 (2H, d, *J* = 8.6 Hz, H-2", H-6"), 7.09 (1H, d, *J* = 8.6 Hz, H-2''', H-6'''), 7.03 (1H, d, *J* = 16.2 Hz, H-7), 6.90 (1H, d, *J* = 16.2 Hz, H-8), 6.75 (8H, m, H-3, H-3', H-3", H-3''', H-5, H-5', H-5", H-5'''), 6.67 (1H, d, *J* = 1.2 Hz, H-10), 6.50 (1H, d, *J* = 1.2 Hz, H-14), 6.18 (1H, d, *J* = 1.2 Hz, H-10'), 6.15 (1H, d, *J* = 1.2 Hz, H-14'), 6.14 (1H, d, *J* = 1.3 Hz, H-14"), 6.11 (1H, d, *J* = 1.3 Hz, H-10"), 6.02 (1H, t, *J* = 2.2 Hz, H-12'''), 5.96 (2H, d, *J* = 2.2 Hz, H-10''', H-14'''), 5.41 (1H, d, *J* = 4.1 Hz, H-7'), 5.37 (1H, d, *J* = 5.0 Hz, H-7"), 5.25 (1H, d, *J* = 5.9 Hz, H-7'''), 4.34 (1H, d, *J* = 4.1 Hz, H-8'), 4.33 (1H, d, *J* = 5.0 Hz, H-8"), 4.22 (1H, d, *J* = 5.9 Hz, H-8'''); ^{13}C NMR (DMSO-d_6, 151 MHz) δ 161.5 (C-11), 161.0 (C-11'), 160.9 (C-11"), 158.3 (C-11''', C-13'''), 157.3 (C-4, C-4", C-4'''), 157.2 (C-4'), 154.6 (C-13), 154.5 (C-13'), 154.5 (C-13"), 145.2 (C-9'), 144.9 (C-9"), 144.6 (C-9'''), 139.8 (C-9), 132.2 (C-1'), 131.8 (C-1"), 131.6 (C-1'''), 128.2 (CH-7), 128.1 (C-1), 127.9 (CH-2, CH-6), 127.3 (CH-2''', CH-6'''), 127.1 (CH-2", CH-6"), 126.8 (CH-2', CH-6'), 125.5 (CH-8), 115.5 (CH-3, CH-5), 115.3 (CH-3', CH-3", CH-3''', CH-5', CH-5", CH-5'''), 113.9 (C-12), 113.1 (C-12'), 113.0 (C-12"), 107.3 (CH-14), 107.2 (CH-14"), 107.2 (CH-14'), 105.4 (CH-10''', CH-14'''), 100.8 (CH-12'''), 99.6 (CH-10', CH-10"), 97.7 (CH-10), 92.4 (CH-7'''), 92.2 (CH-7"), 91.9 (CH-7'), 54.4 (CH-8'''), 54.2 (CH-8"), 54.2 (CH-8'); (NP-MRD ID: NP0140175); HRESIMS *m/z* 907.2742 $[M+H]^+$ (calcd for $C_{56}H_{43}O_{12}^+$ 907.2749, Δ = -0.77 ppm), MS/MS spectrum: CCMSLIB00012475004, *m/z* 905.2579 $[M-H]^-$ (calcd for $C_{56}H_{41}O_{12}^-$ 905.2604, Δ = -2.76 ppm).

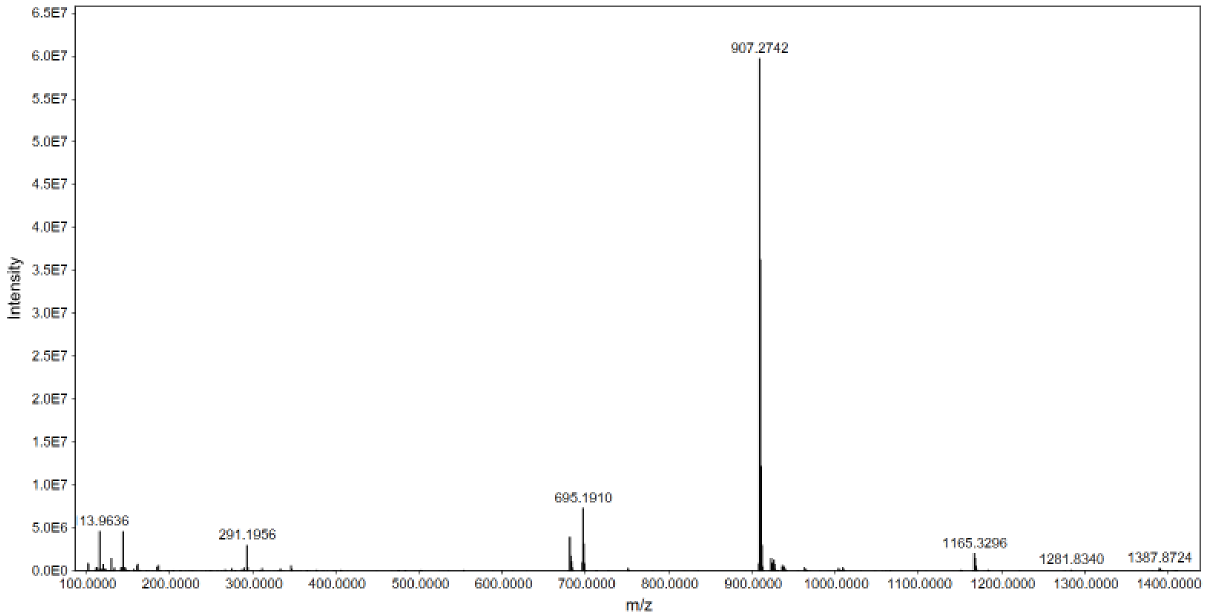

S8.1. HRESIMS+ spectrum of Gnemonol B in MeOH.

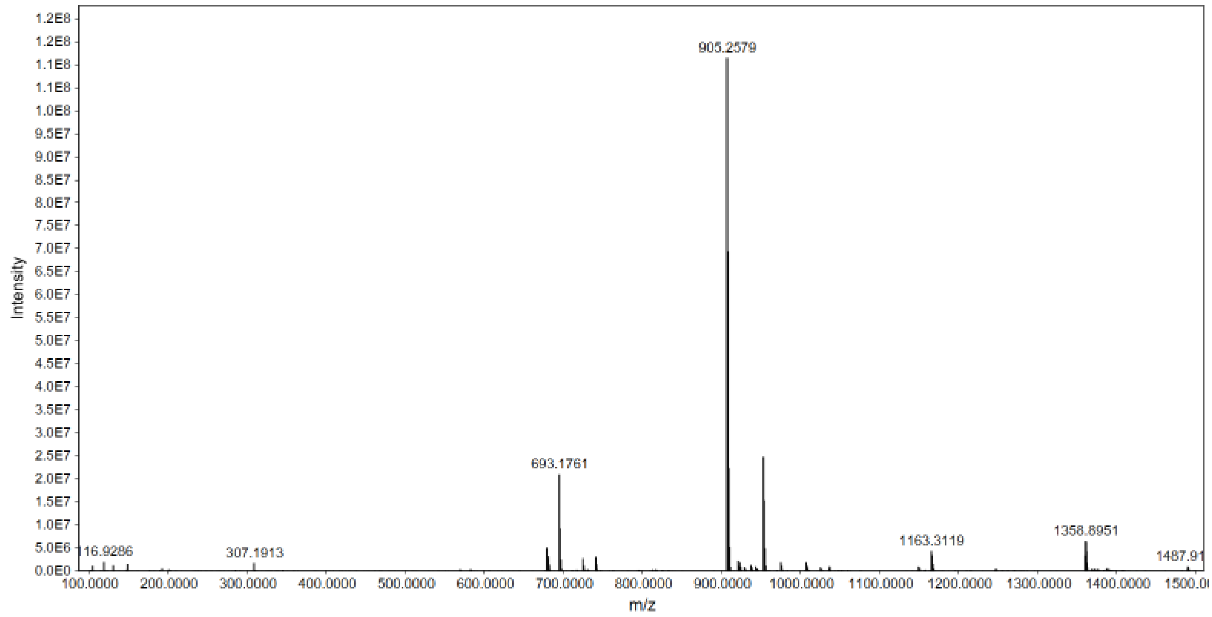

S8.2. HRESIMS- spectrum of Gnemonol B in MeOH.

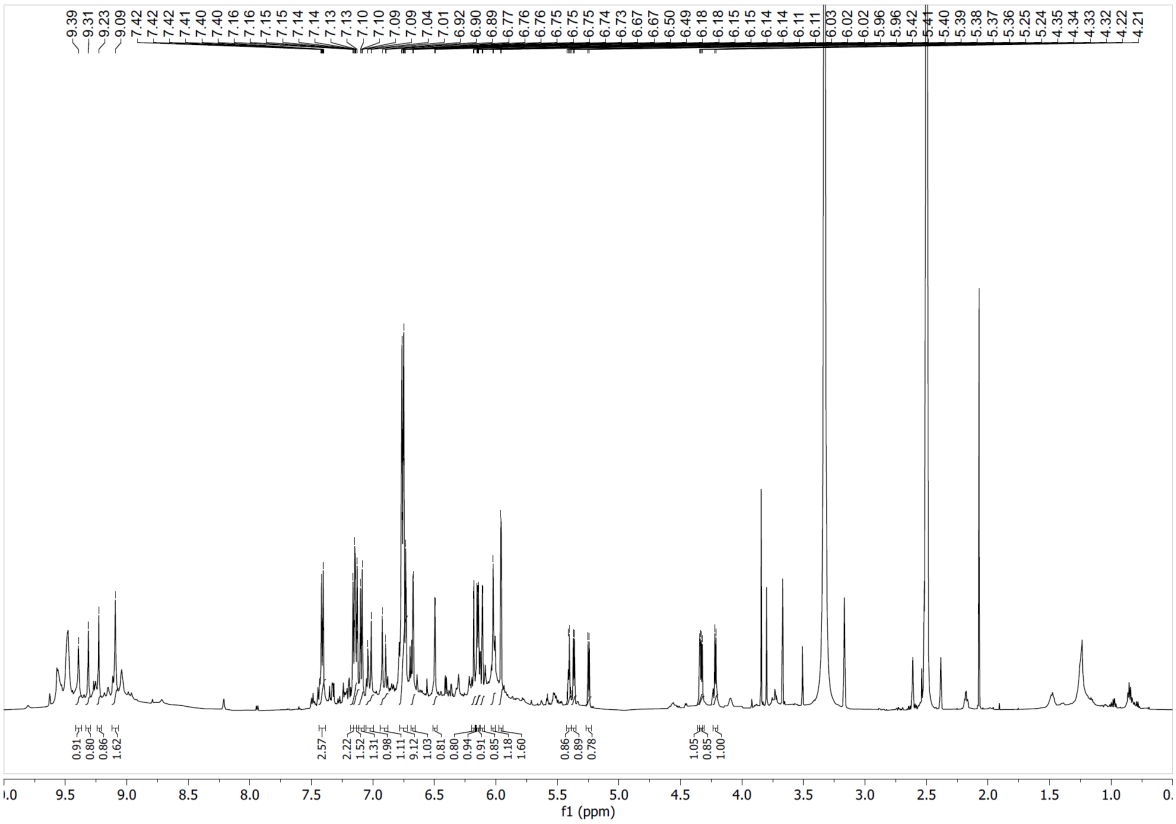

S8.3. ^{1}H NMR spectrum of Gnemonol B in DMSO-d_6 at 600 MHz.

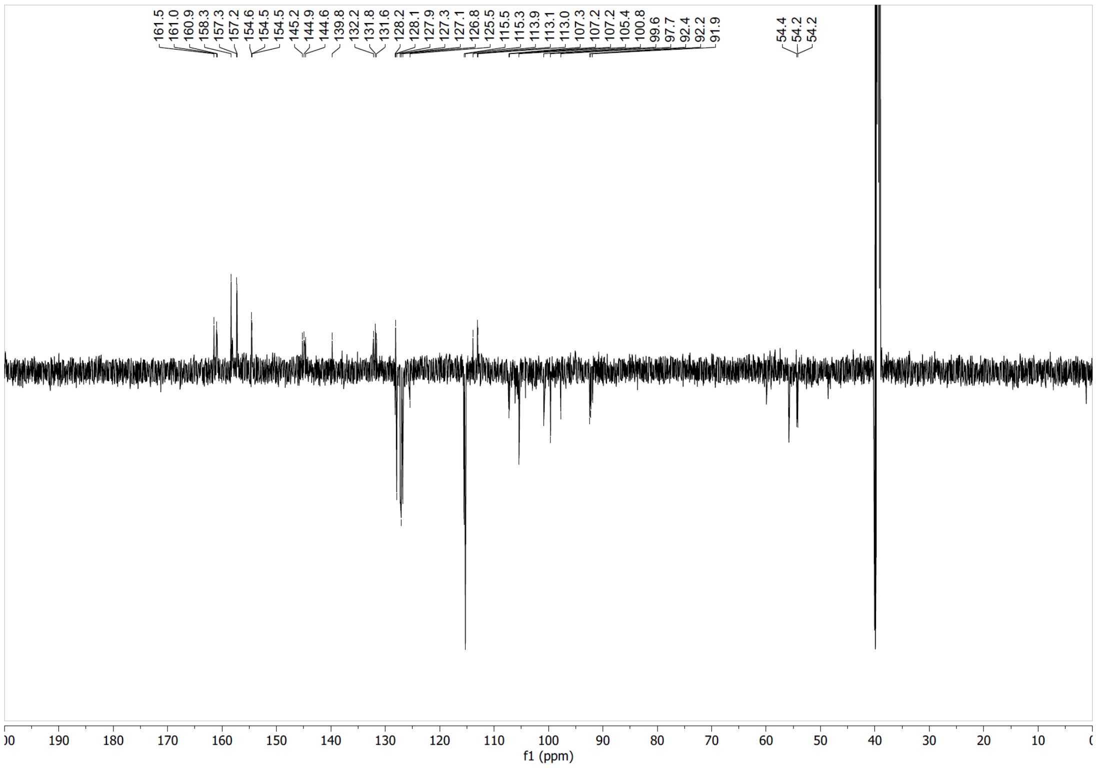

S8.4. ^{13}C NMR spectrum of Gnemonol B in DMSO-d_6 at 151 MHz.

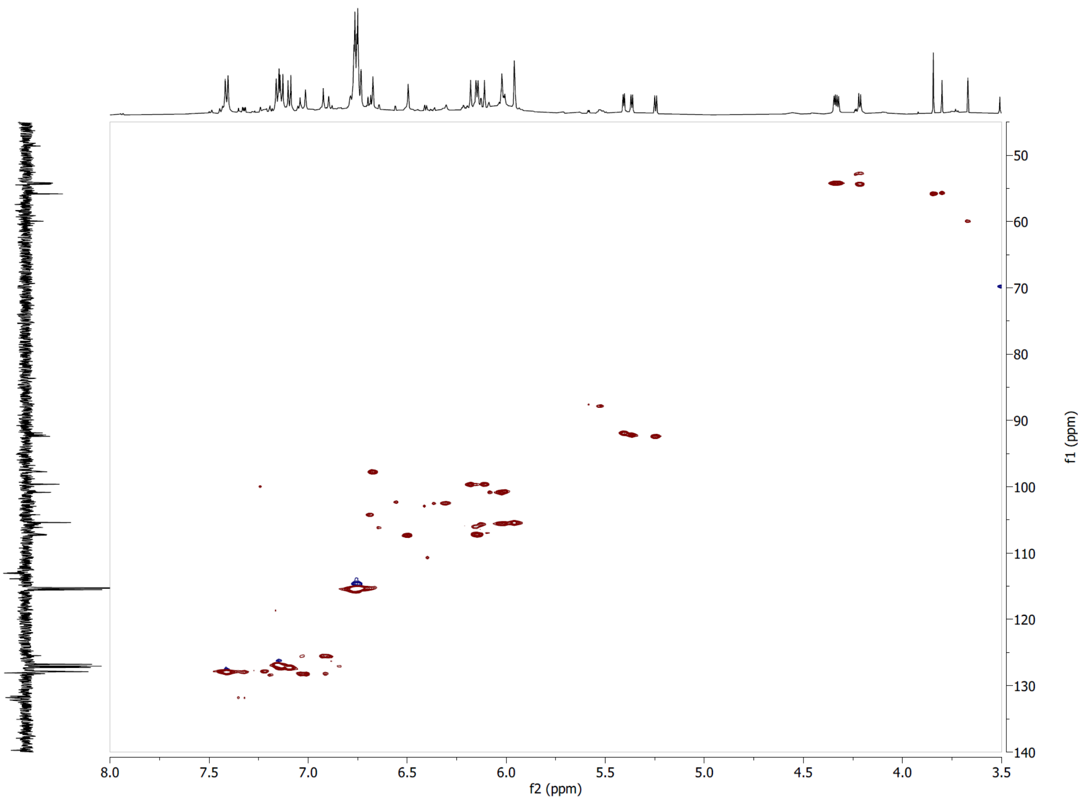

S8.5. HSQC NMR spectrum of Gnemonol B in DMSO-*d*6.

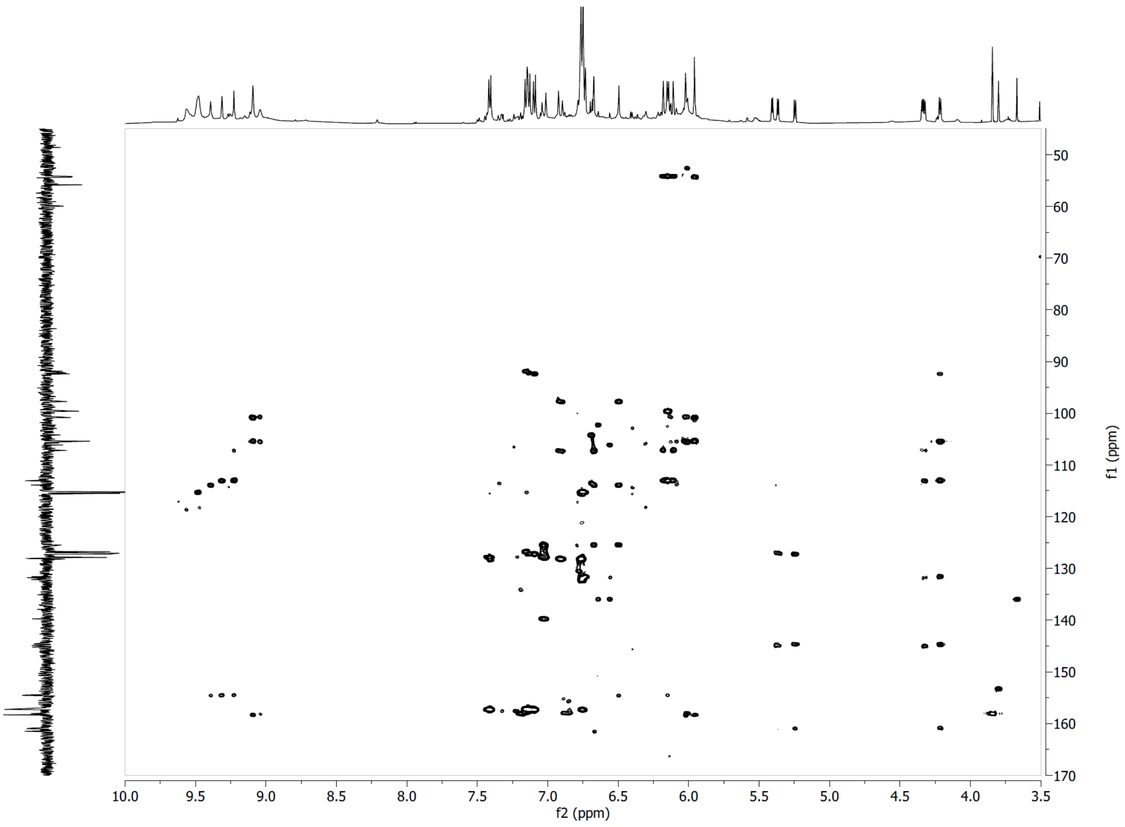

S8.6. HMBC NMR spectrum of Gnemonol B in DMSO-*d*6.

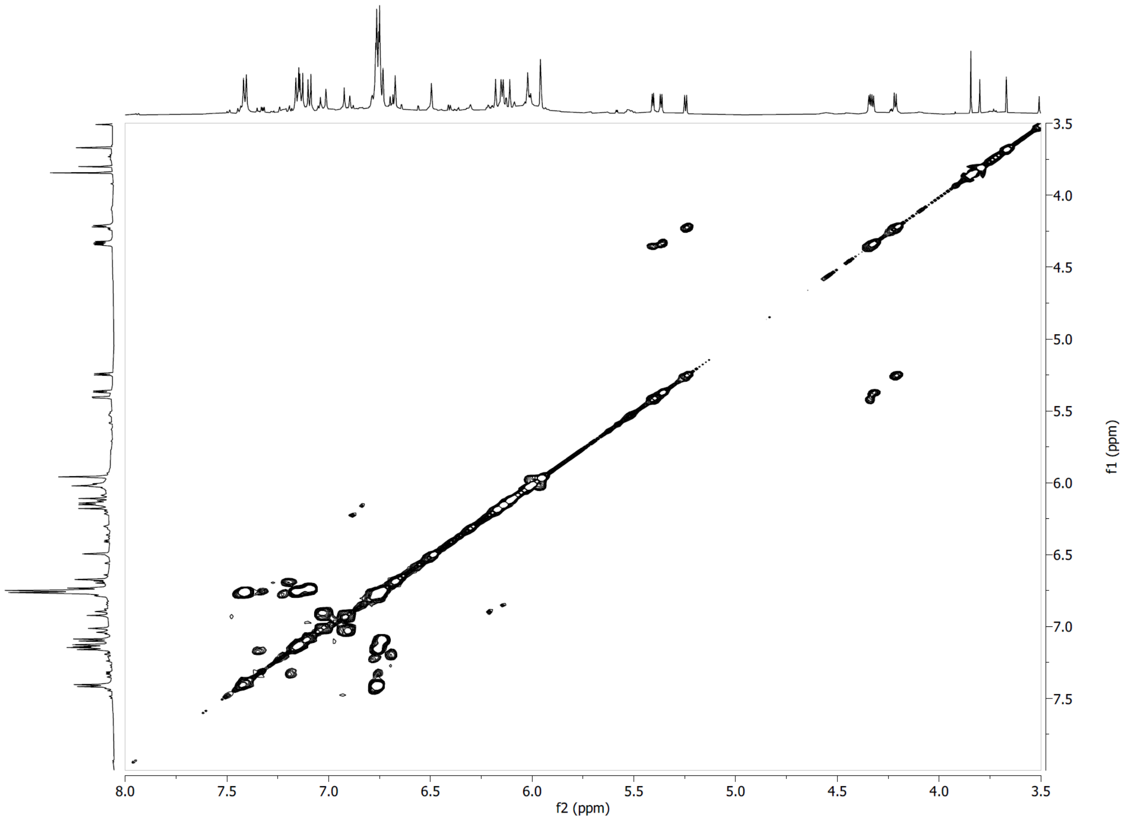

S8.7. COSY NMR spectrum of Gnemonol B in DMSO-d_6.

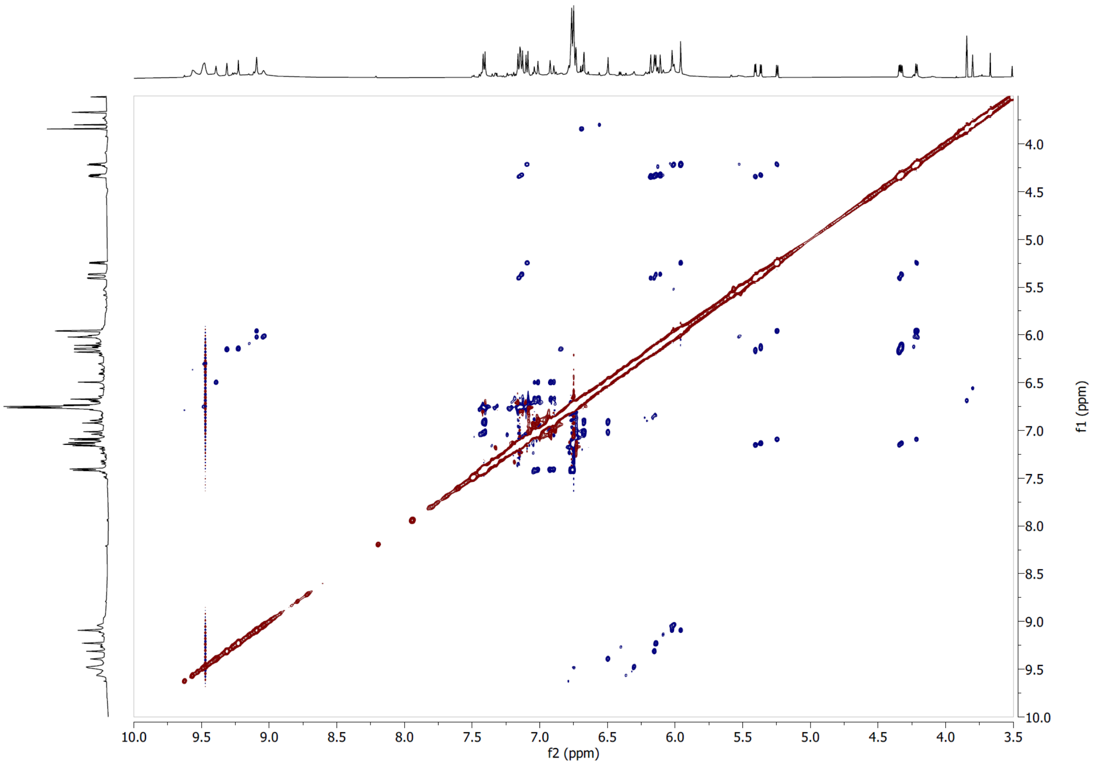

S8.9. ROESY NMR spectrum of Gnemonol B in DMSO-d_6.

9. Gnemontanin G:

Experimental:

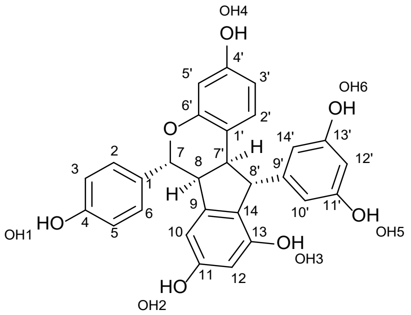

Gnemontanin G (9) $[\alpha]_D^{20}$ -16.8 (c 0.06, MeOH), Literature[10] : $[\alpha]_D^{20}$ -19.6 (c 0.05, MeOH); UV (MeOH) λ_{max} (log ε) 226 (4.09), 287 (3.29), 310 (2.84), 328 (2.94), 347 (2.51) nm; ^{1}H NMR (DMSO-d_6, 600 MHz) δ 9.44 (1H, s, 4-OH), 9.19 (1H, s, 4'-OH), 8.97 (2H, s, 11'-OH, 13'-OH), 8.85 (1H, s, 11-OH), 8.62 (1H, s, 13-OH), 7.10 (2H, d, *J* = 8.5 Hz, H-2, H-6), 6.72 (2H, d, *J* = 8.5 Hz, H-3, H-5), 6.57 (1H, d, *J* = 8.4 Hz, H-2'), 6.22 (1H, dd, *J* = 8.4, 2.4 Hz, H-3'), 6.17 (1H, d, *J* = 2.4 Hz, H-5'), 6.03 (1H, t, *J* = 2.1 Hz, H-12'), 6.02 (2H, d, *J* = 2.1 Hz, H-10', H-14'), 5.99 (1H, d, *J* = 2.0 Hz, H-12), 5.53 (1H, d, *J* = 2.0 Hz, H-10), 4.72 (1H, d, *J* = 8.5 Hz, H-7), 3.95 (1H, d, *J* = 7.1 Hz, H-8'), 3.51 (1H, t, *J* = 8.5, 7.1 Hz, H-8), 3.27 (1H, t, *J* = 7.1, 7.1 Hz, H-7'); ^{13}C NMR (DMSO-d_6, 151 MHz) δ 157.9 (C-11', C-13'), 157.1 (C-11), 157.0 (C-4), 156.5 (C-4'), 154.7 (C-6'), 154.2 (C-13), 147.0 (C-9'), 144.8 (C-9), 130.1 (C-1), 129.7 (CH-2'), 129.0 (CH-2, CH-6), 121.3 (C-14), 115.4 (C-1'), 114.8 (CH-3, CH-5), 108.4 (CH-3'), 106.2 (CH-10', CH-14'), 103.4 (CH-10), 102.6 (CH-5'), 101.8 (CH-12), 100.3 (CH-12'), 77.3 (CH-7), 56.1 (CH-8'), 48.4 (CH-7'), 47.0 (CH-8); (NP-MRD ID: NP0332866); HRESIMS *m/z* 471.1432 $[M+H]^+$ (calcd for $C_{28}H_{23}O_7^+$ 471.1438, Δ = -1.27 ppm), MS/MS spectrum: CCMSLIB00012474989, *m/z* 469.1289 $[M-H]^-$ (calcd for $C_{28}H_{21}O_7^-$ 469.1293, Δ = -0.85 ppm).

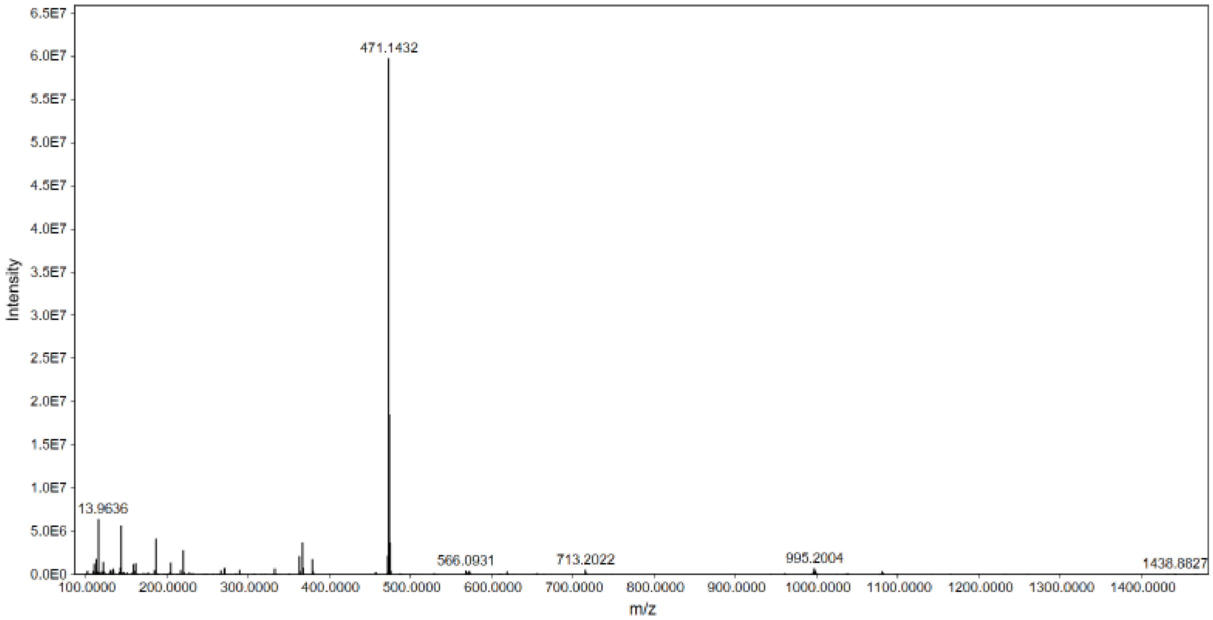

S9.1. HRESIMS+ spectrum of Gnemontanin G in MeOH.

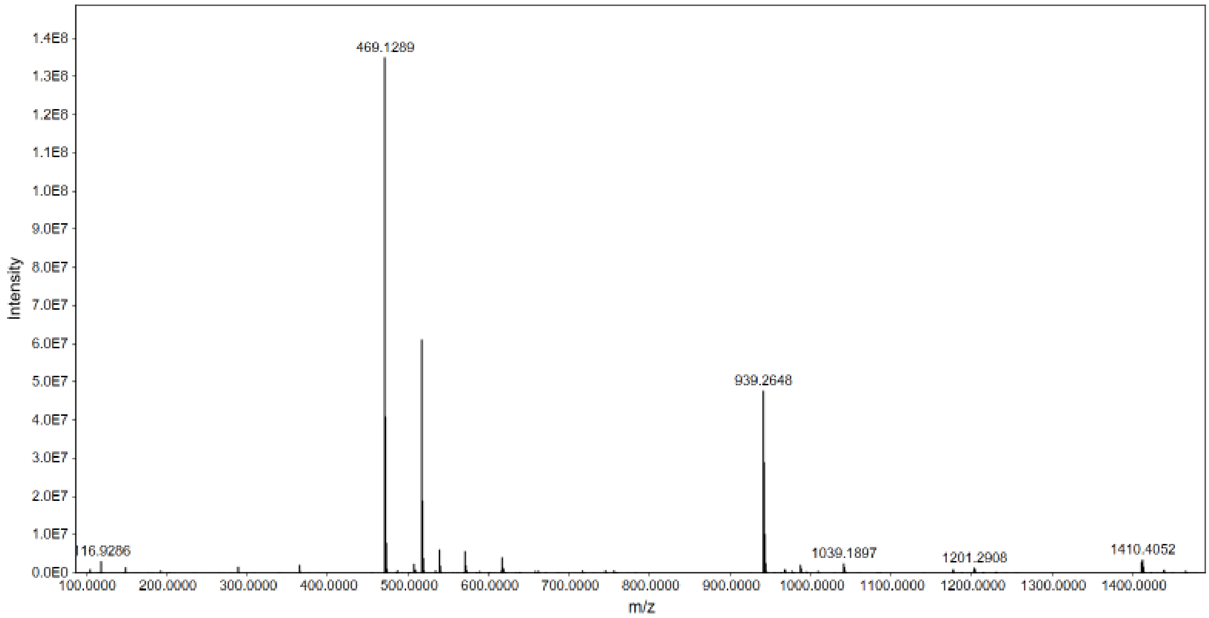

S9.2. HRESIMS- spectrum of Gnemontanin G in MeOH.

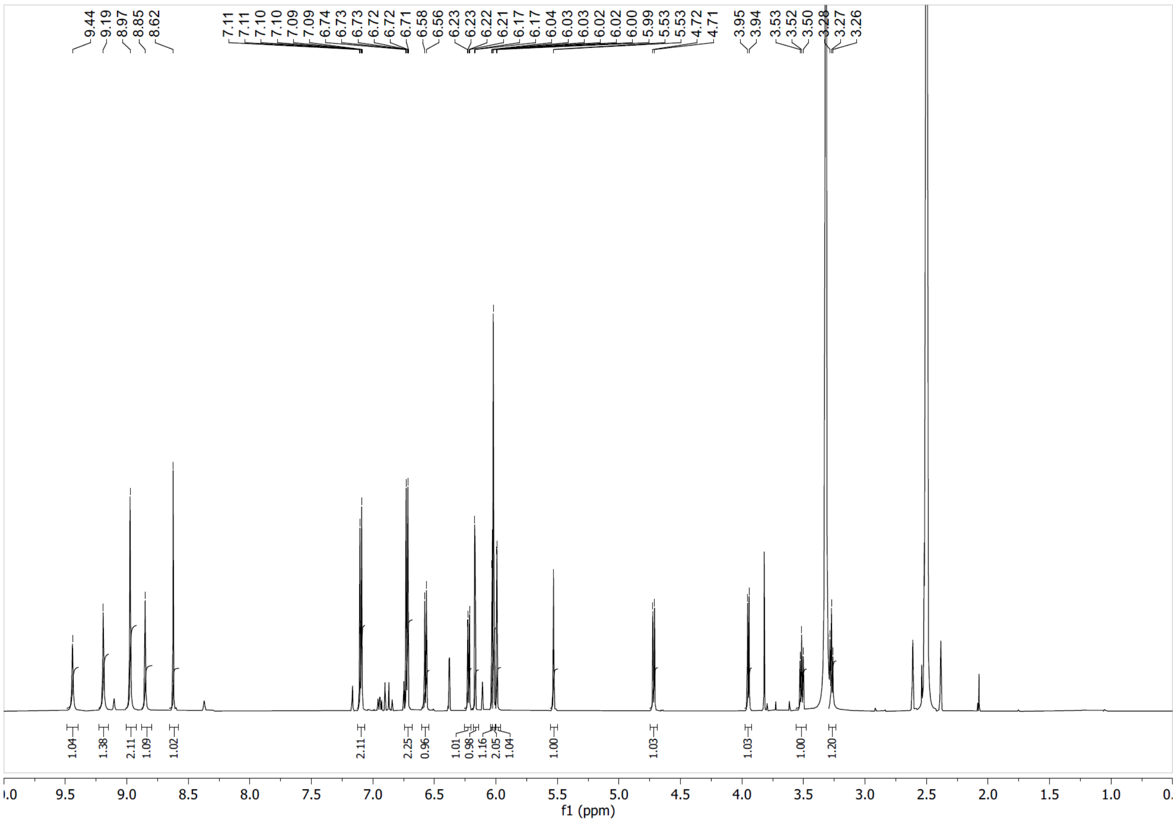

S9.3. ^{1}H NMR spectrum of Gnemontanin G in DMSO-d_6 at 600 MHz.

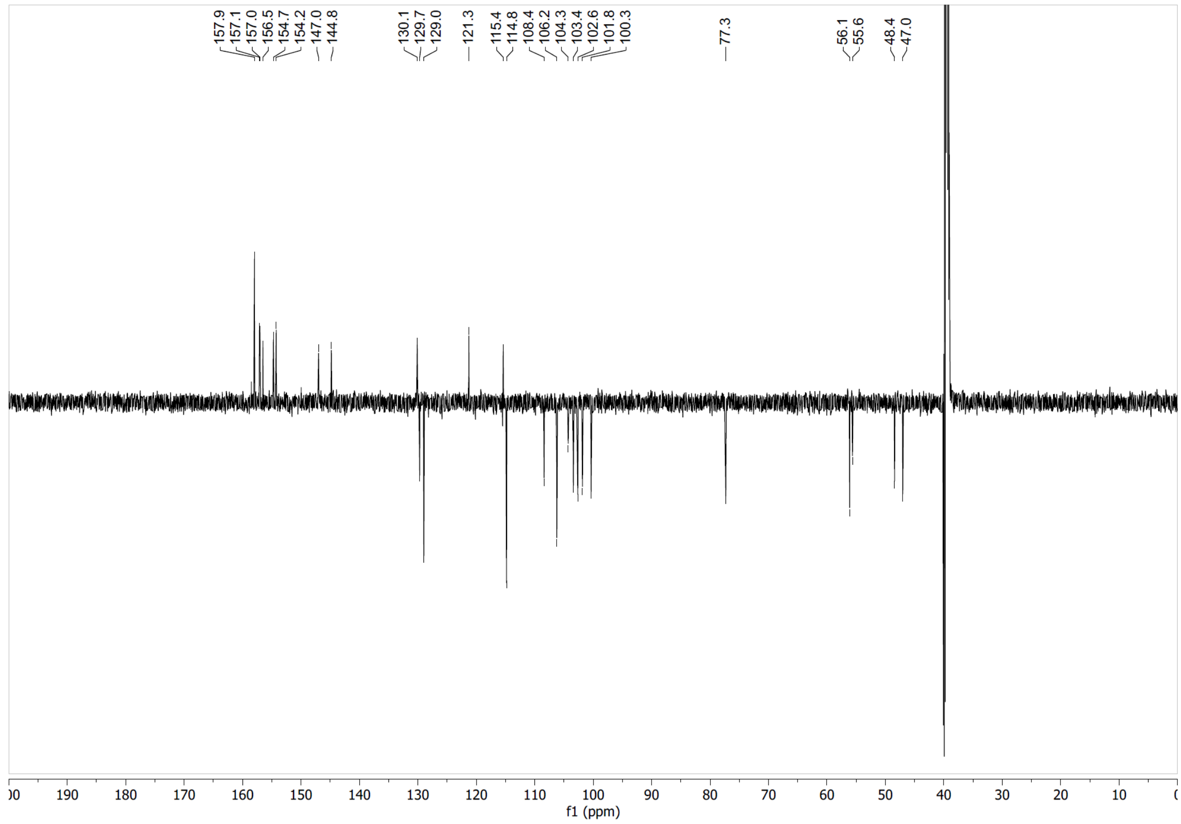

S9.4. ^{13}C NMR spectrum of Gnemontanin G in DMSO-d_6 at 151 MHz.

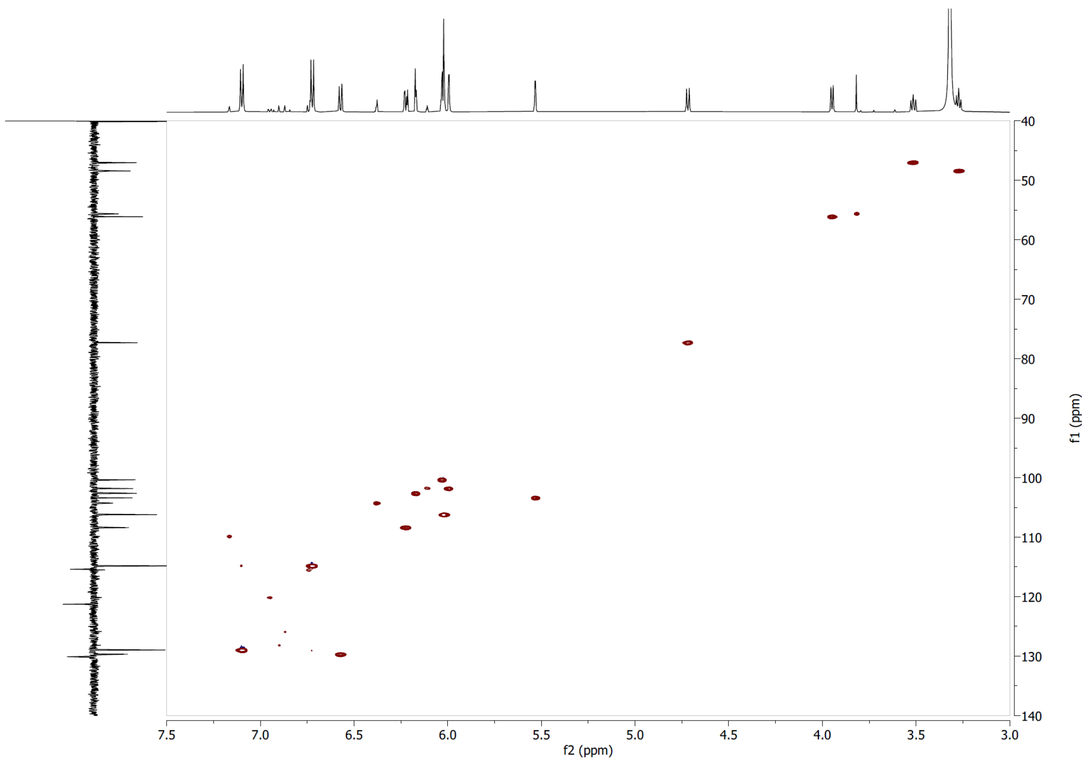

S9.5. HSQC NMR spectrum of Gnemontanin G in DMSO-d_6.

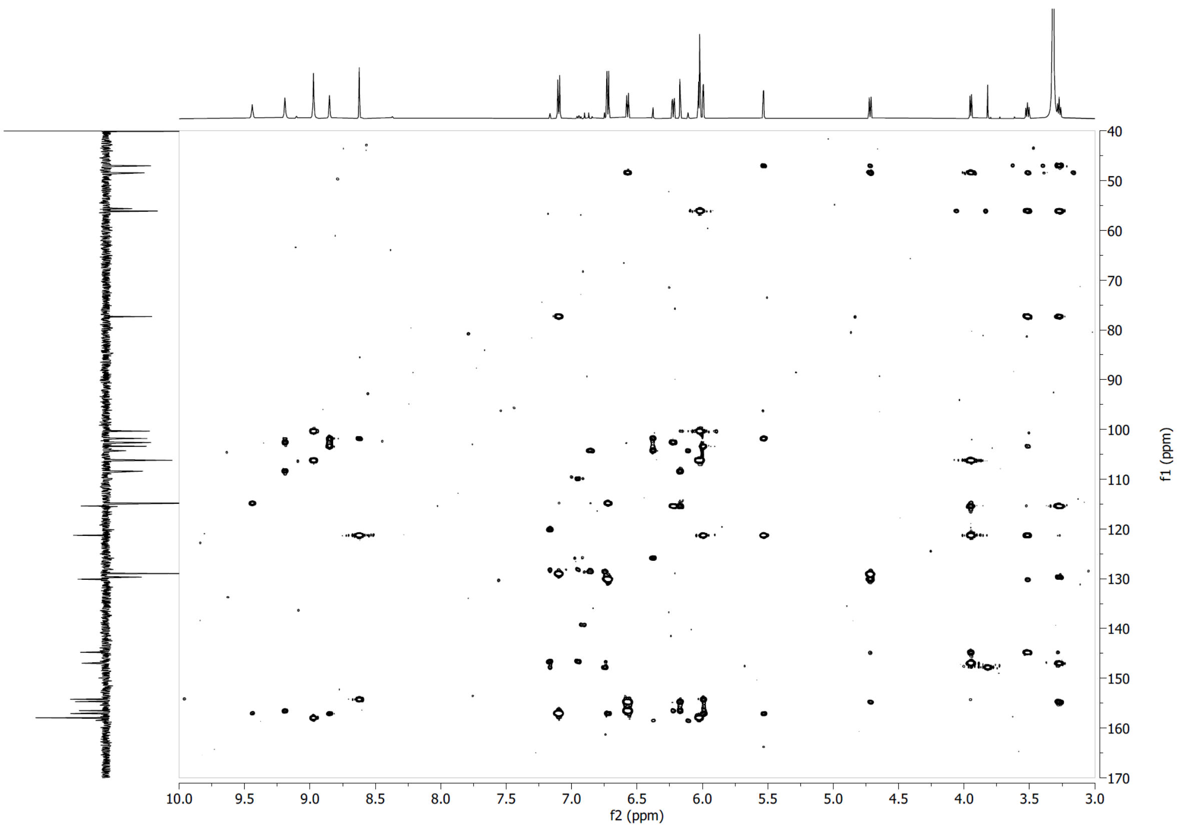

S9.6. HMBC NMR spectrum of Gnemontanin G in DMSO-d_6.

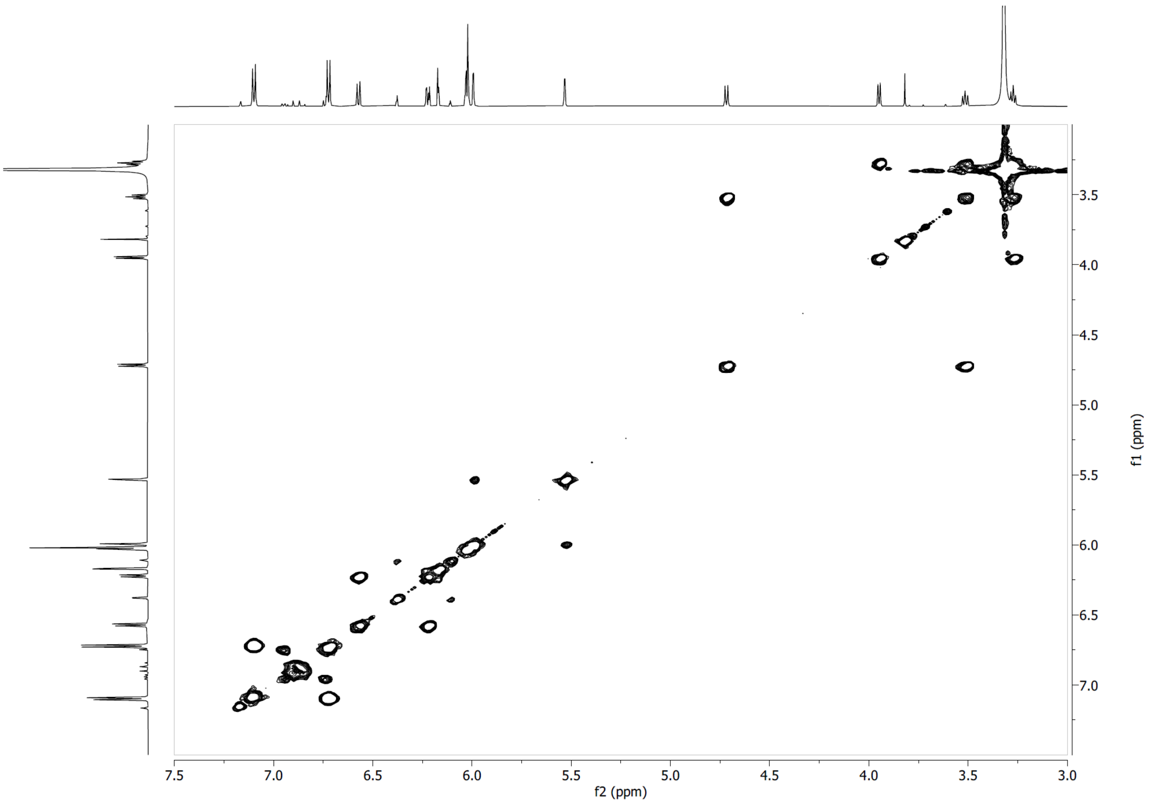

S9.7. COSY NMR spectrum of Gnemontanin G in DMSO-d_6.

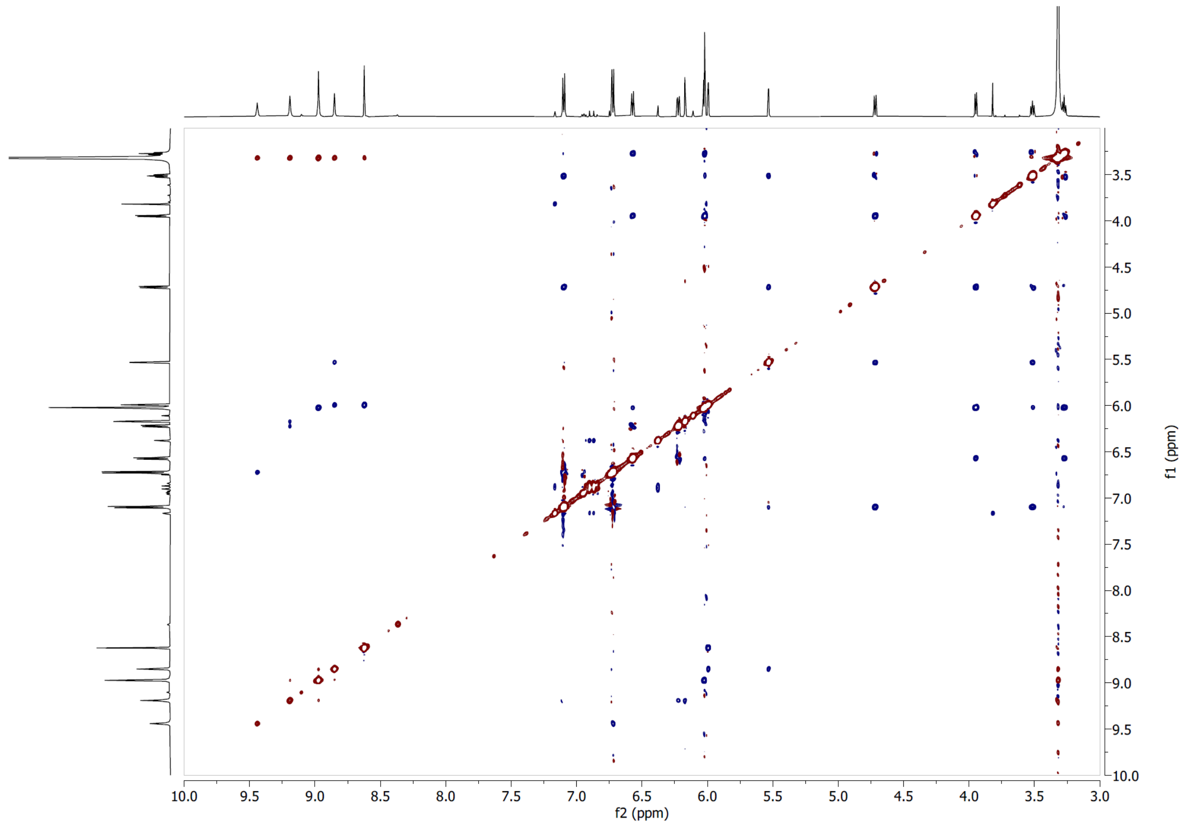

S9.8. ROESY NMR spectrum of Gnemontanin G in DMSO-*d*$_6$.

10. (-)-Gnetumontanin A:

Experimental:

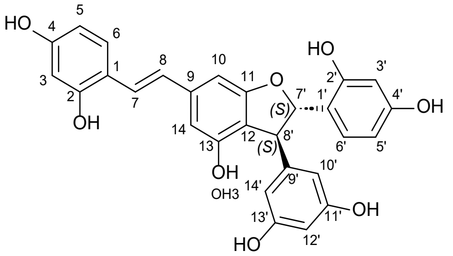

(-)-Gnetumontanin A (10) $[\alpha]_D^{20}$ -13.4 (c 0.05, MeOH), Enantiomer of (+)-Gnetumontanin A reported[11]: $[\alpha]_D^{20}$ +17 (c 0.1, MeOH); UV (MeOH) λ_{max} (log ε) 226 (3.88), 287 (3.38), 305 (3.41), 333 (4.57), 342 (3.54) nm; ^{1}H NMR (DMSO-d_6, 600 MHz) δ 9.59 (1H, s, 2-OH), 9.54 (1H, s, 2'-OH), 9.40 (1H, s, 4-OH), 9.24 (1H, s, 4'-OH), 9.20 (1H, s, 13-OH), 9.04 (2H, s, 11'-OH, 13'-OH), 7.35 (1H, d, *J* = 8.5 Hz, H-6), 7.19 (1H, d, *J* = 16.4 Hz, H-7), 6.85 (1H, d, *J* = 16.4 Hz, H-8), 6.84 (1H, d, *J* = 8.4 Hz, H-6'), 6.54 (1H, d, *J* = 1.3 Hz, H-10), 6.44 (1H, d, *J* = 1.3 Hz, H-14), 6.33 (1H, d, *J* = 2.5 Hz, H-3), 6.32 (1H, d, *J* = 2.4 Hz, H-3'), 6.25 (1H, dd, *J* = 8.5, 2.5 Hz, H-5), 6.14 (1H, dd, *J* = 8.4, 2.4 Hz, H-5'), 6.04 (2H, d, *J* = 2.2 Hz, H-10', H-14'), 6.02 (1H, t, *J* = 2.2 Hz, H-12'), 5.53 (1H, d, *J* = 3.4 Hz, H-7'), 4.18 (1H, d, *J* = 3.4 Hz, H-8'); ^{13}C NMR (DMSO-d_6, 151 MHz) δ 161.7 (C-11), 158.1 (C-4), 158.1 (C-11', C-13'), 157.9 (C-4'), 156.1 (C-2), 155.3 (C-2'), 154.6 (C-13), 145.7 (C-9'), 140.1 (C-9), 127.2 (CH-6), 126.5 (CH-6'), 124.7 (CH-8), 123.3 (CH-7), 118.7 (C-1'), 115.3 (C-1), 114.3 (C-12), 107.2 (CH-5), 106.1 (CH-14), 105.9 (CH-5'), 105.5 (CH-10', CH-14'), 102.6 (CH-3), 102.5 (CH-3'), 100.6 (CH-12'), 97.8 (CH-10), 87.6 (CH-7'), 52.8 (CH-8'); (NP-MRD ID: NP0332867); HRESIMS *m/z* 487.1382 $[M+H]^+$ (calcd for $C_{28}H_{23}O_8^+$ 487.1387, Δ = -1.03 ppm), MS/MS spectrum: CCMSLIB00012474986, *m/z* 485.1240 $[M-H]^-$ (calcd for $C_{28}H_{21}O_8^-$ 485.1242, Δ = -0.41 ppm).

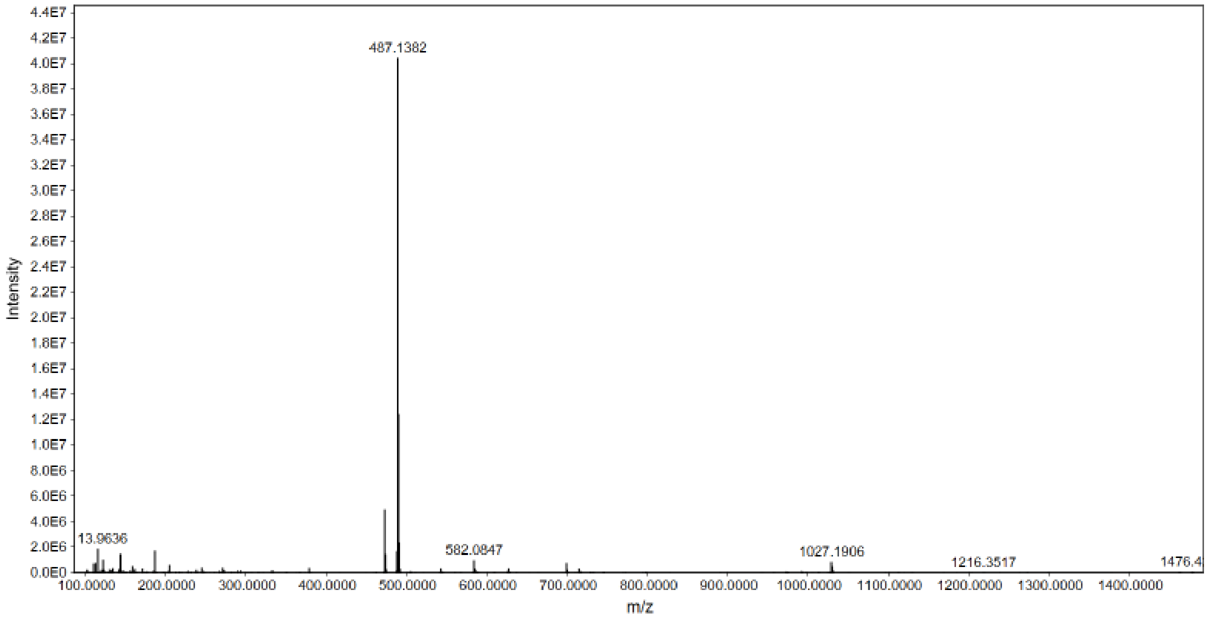

S10.1. HRESIMS+ spectrum of (-)-Gnetumontanin A in MeOH.

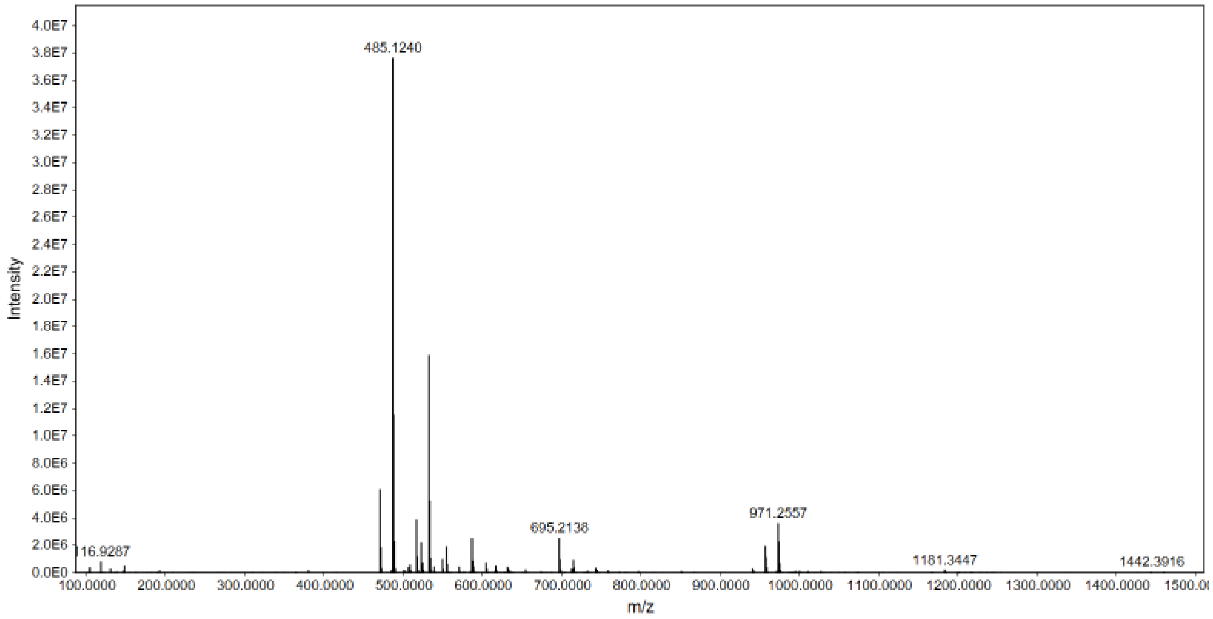

S10.2. HRESIMS- spectrum of (-)-Gnetumontanin A in MeOH.

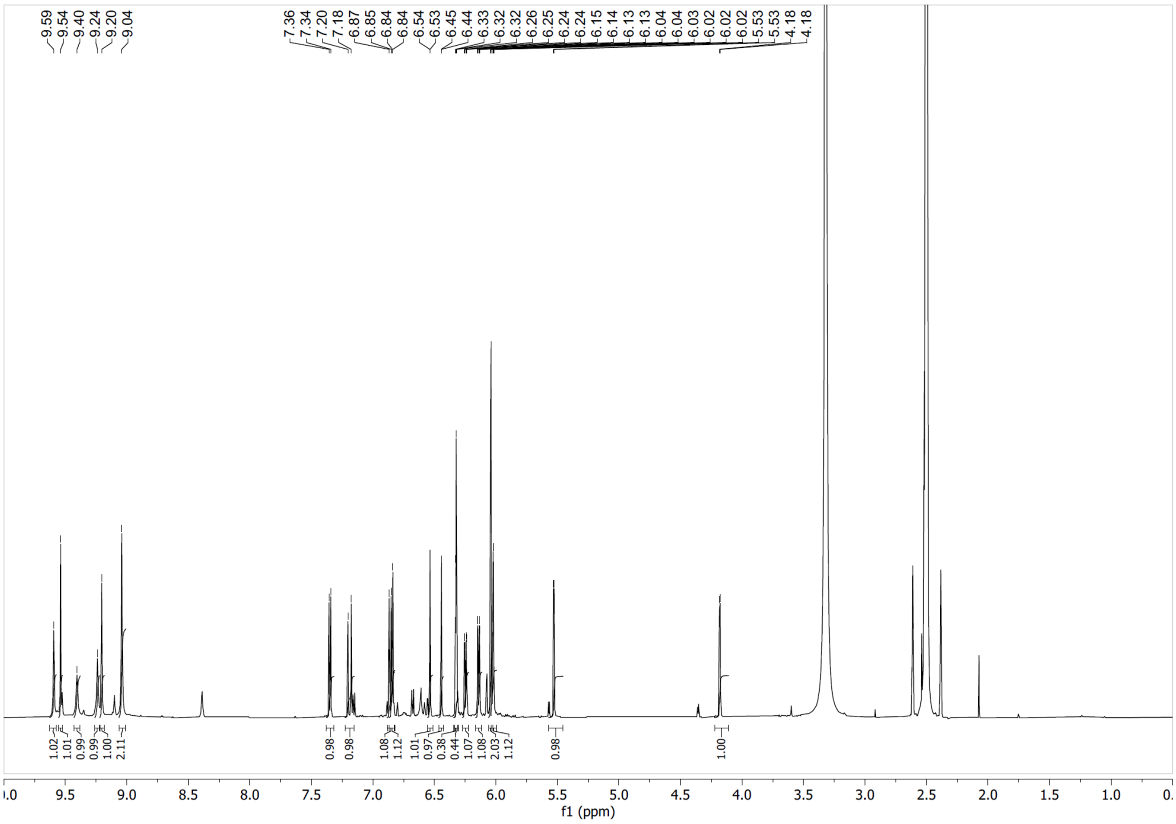

S10.3. ^{1}H NMR spectrum of (-)-Gnetumontanin A in DMSO-d_6 at 600 MHz.

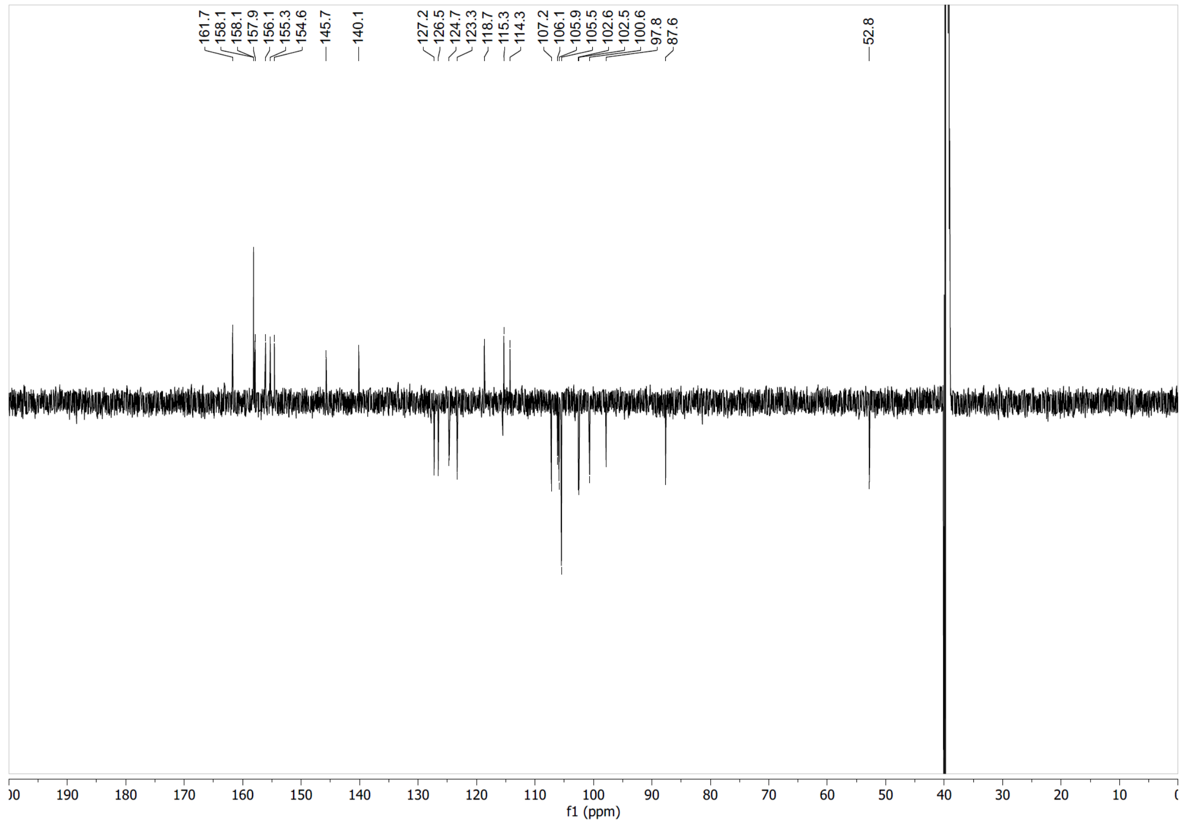

S10.4. ^{13}C NMR spectrum of (-)-Gnetumontanin A in DMSO-d_6 at 151 MHz.

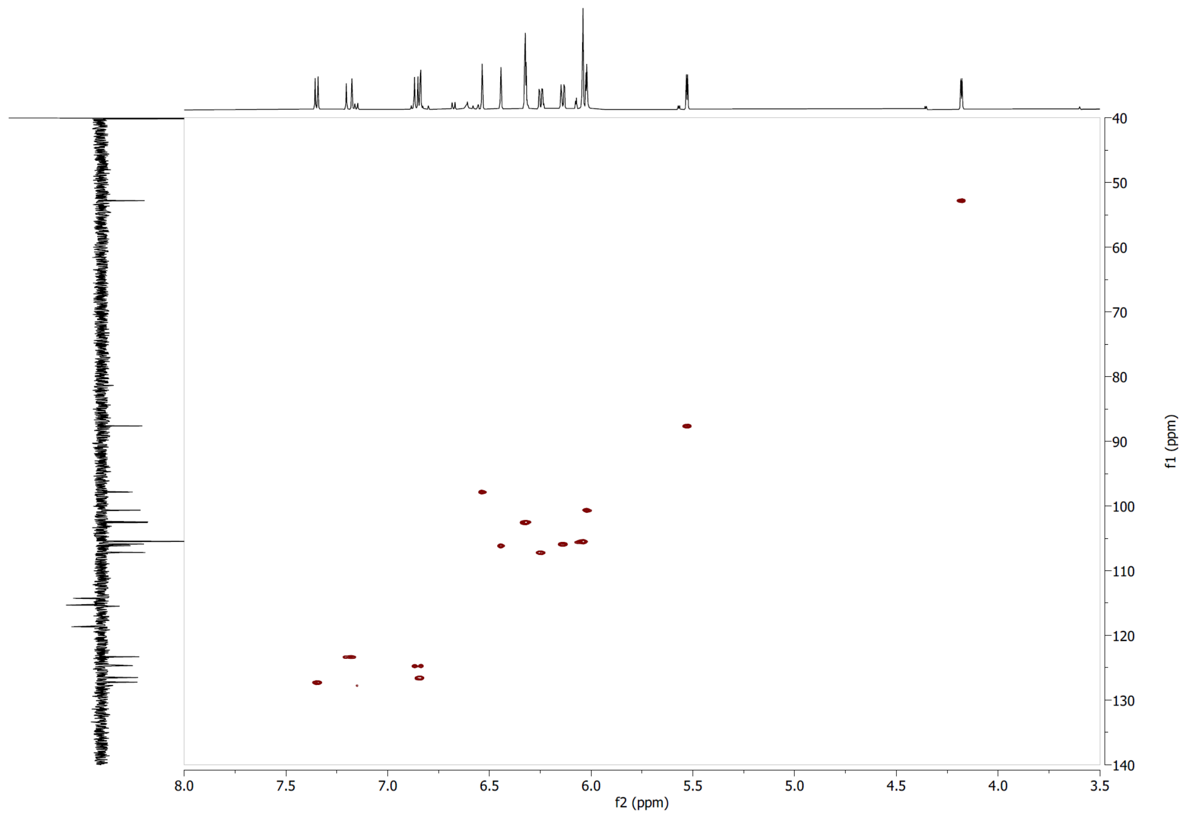

S10.5. HSQC NMR spectrum of (-)-Gnetumontanin A in DMSO-d_6.

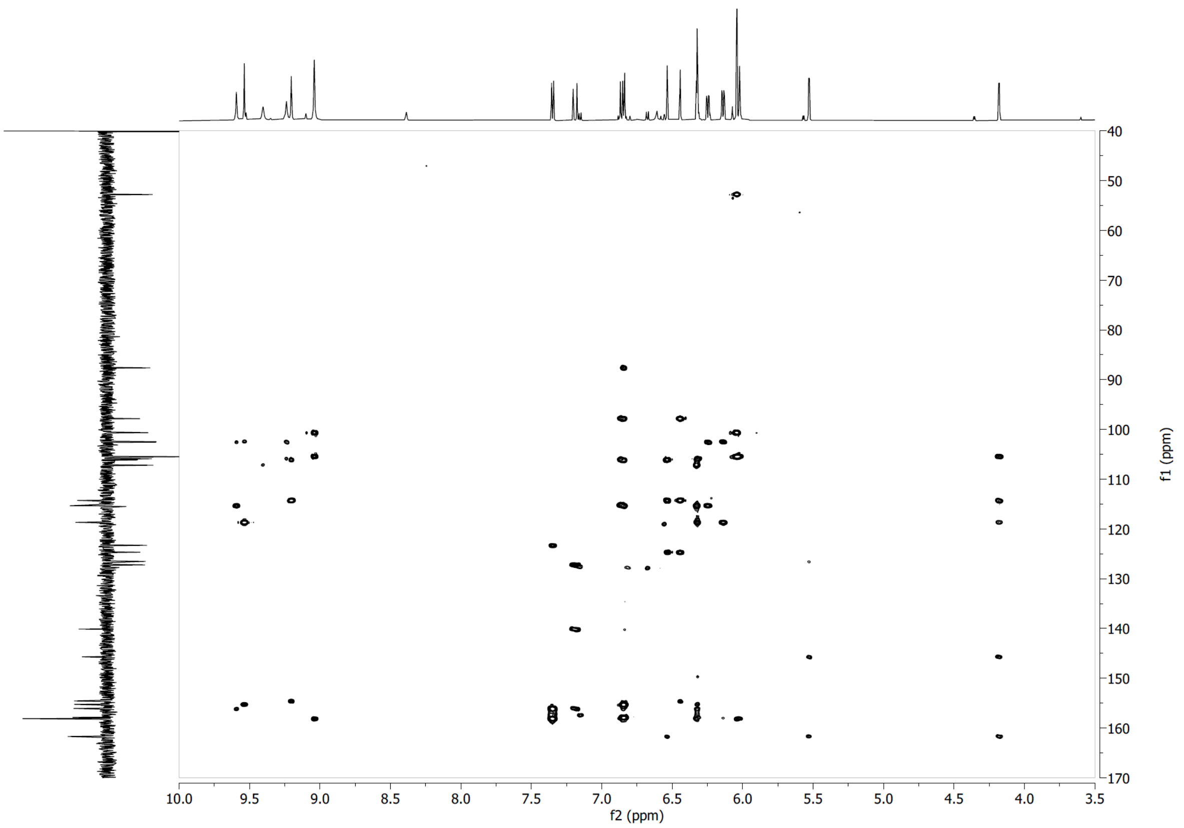

S10.6. HMBC NMR spectrum of (-)-Gnetumontanin A in DMSO-*d*6.

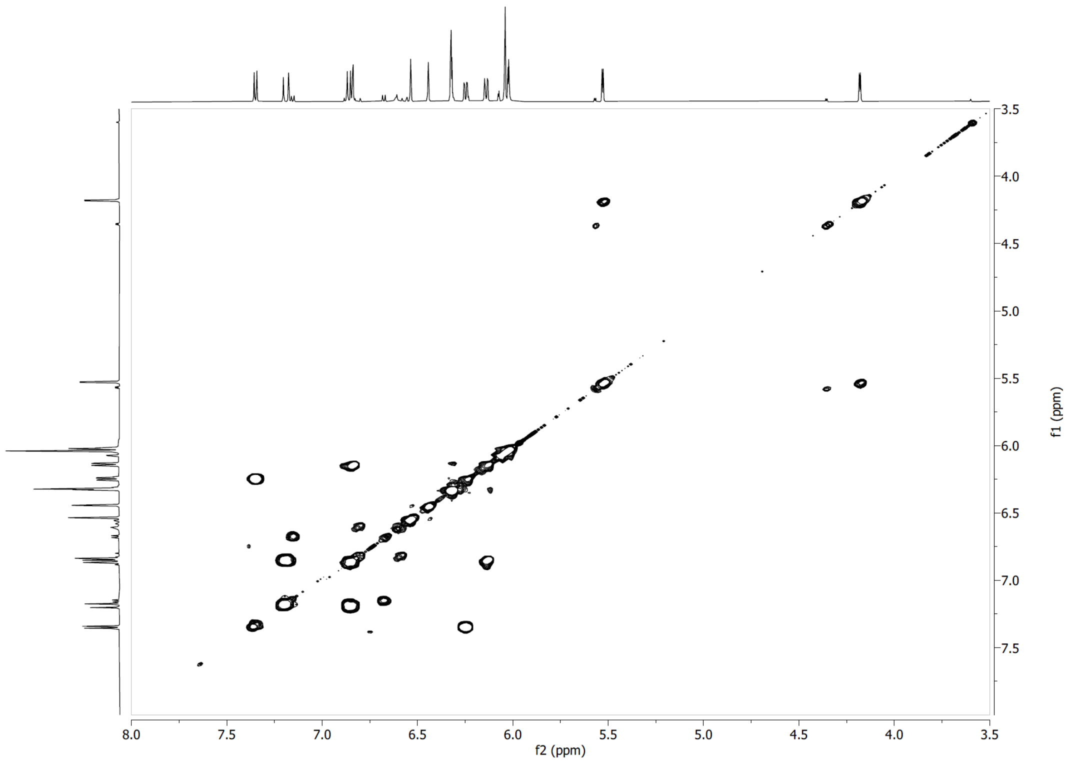

S10.7. COSY NMR spectrum of (-)-Gnetumontanin A in DMSO-*d*6.

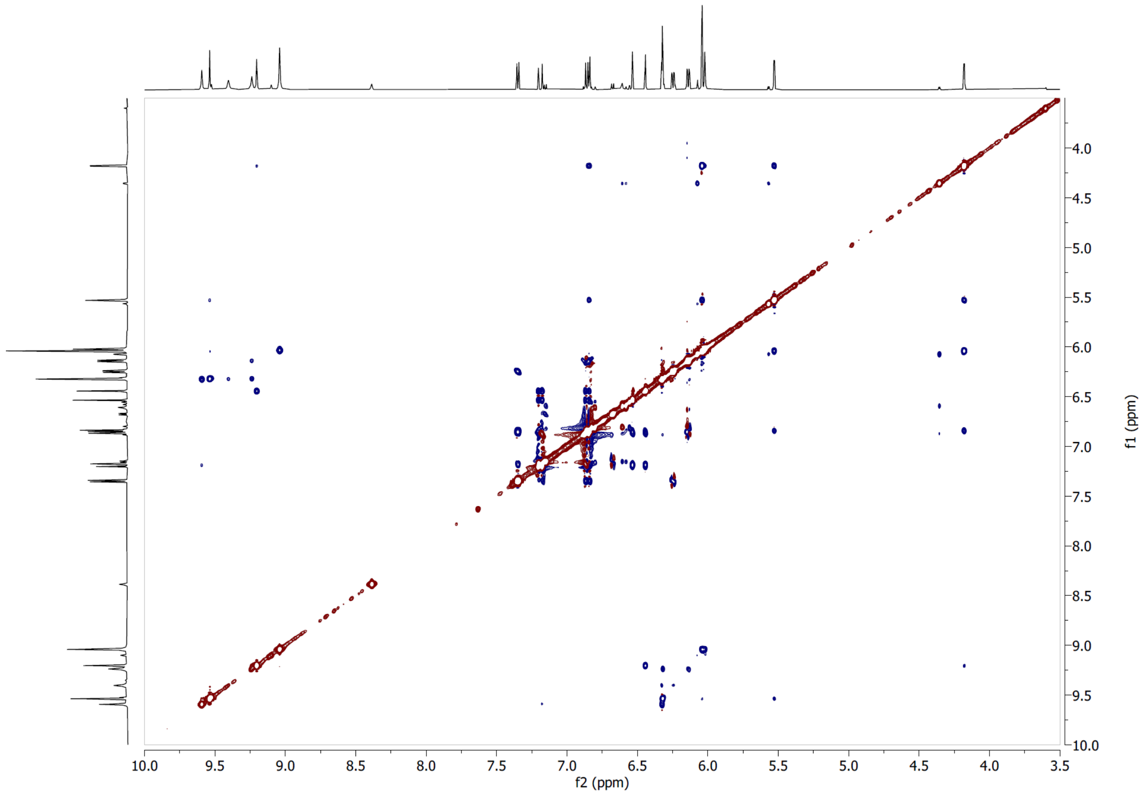

S10.9. ROESY NMR spectrum of (-)-Gnetumontanin A in DMSO-d_6.

11. (-)-Gnetuhainin M:

Experimental:

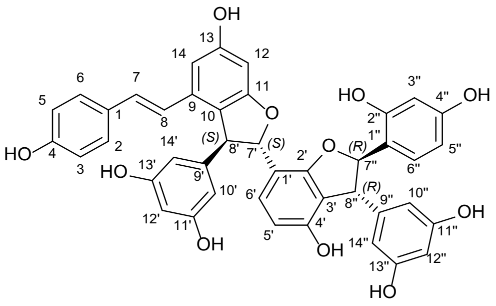

(-)-Gnetuhainin M (11) $[\alpha]_D^{20}$ -44.9 (c 0.12, MeOH), Literature[12]: $[\alpha]_D^{20}$ -32.9 (c 0.11, MeOH); UV (MeOH) λ_{max} (log ε) 226 (4.63), 287 (4.11), 310 (4.16), 328 (4.19), 347 (3.90) nm; ^{1}H NMR (DMSO-d_6, 600 MHz) δ 9.57 (1H, s, 4-OH), 9.53 (1H, s, 2''-OH), 9.37 (2H, s, 4'-OH, 13-OH), 9.17 (1H, s, 4''-OH), 9.15 (2H, s, 11'-OH, 13'-OH), 9.07 (2H, s, 11''-OH, 13''-OH), 7.09 (2H, d, *J* = 8.7 Hz, H-2, H-6), 7.05 (1H, d, *J* = 8.5 Hz, H-6'), 6.83 (1H, d, *J* = 16.3 Hz, H-7), 6.68 (2H, d, *J* = 8.7 Hz, H-3, H-5), 6.59 (1H, d, *J* = 2.0 Hz, H-14), 6.58 (1H, d, *J* = 16.3 Hz, H-8), 6.55 (1H, d, *J* = 8.4 Hz, H-6''), 6.32 (1H, d, *J* = 2.5 Hz, H-3''), 6.31 (1H, d, *J* = 8.5 Hz, H-5'), 6.23 (1H, d, *J* = 2.0 Hz, H-12), 6.10 (2H, d, *J* = 2.2 Hz, H-10', H-14'), 6.08 (2H, d, *J* = 2.2 Hz, H-10'', H-14''), 6.07 (1H, t, *J* = 2.2 Hz, H-12'), 6.07 (1H, dd, *J* = 8.4, 2.5 Hz, H-5''), 6.04 (1H, t, *J* = 2.2 Hz, H-12''), 5.61 (1H, d, *J* = 6.6 Hz, H-7'), 5.54 (1H, d, *J* = 2.7 Hz, H-7''), 4.65 (1H, d, *J* = 6.6 Hz, H-8'), 4.10 (1H, d, *J* = 2.7 Hz, H-8''); ^{13}C NMR (DMSO-d_6, 151 MHz) δ 160.8 (C-11), 158.9 (C-2'), 158.6 (C-11', C-13'), 158.4 (C-13), 158.1 (C-11'', C-13''), 157.6 (C-4''), 157.3 (C-4), 154.9 (C-4'), 154.8 (C-2''), 145.6 (C-9'), 145.5 (C-9''), 134.7 (C-9), 128.8 (CH-7), 128.0 (C-1), 127.7 (CH-2, CH-6), 127.6 (CH-6'), 125.8 (CH-6''), 122.0 (CH-8), 118.9 (C-10), 118.6 (C-1''), 115.5 (CH-3, CH-5), 115.1 (C-3'), 112.8 (C-1'), 108.6 (CH-5'), 105.9 (CH-5''), 105.7 (CH-10', CH-14'), 105.6 (CH-10'', CH-14''), 102.9 (CH-14), 102.4 (CH-3''), 101.1 (CH-12'), 100.7 (CH-12''), 96.0 (CH-12), 88.2 (CH-7''), 88.2 (CH-7'), 53.9 (CH-8'), 53.3 (CH-8''); (NP-MRD ID: NP0332868); HRESIMS *m/z* 713.2018 $[M+H]^+$ (calcd for $C_{42}H_{33}O_{11}^+$ 713.2017, Δ = 0.14 ppm), MS/MS spectrum: CCMSLIB00012474996, *m/z* 711.1870 $[M-H]^-$ (calcd for $C_{42}H_{31}O_{11}^-$ 711.1872, Δ = -0.28 ppm).

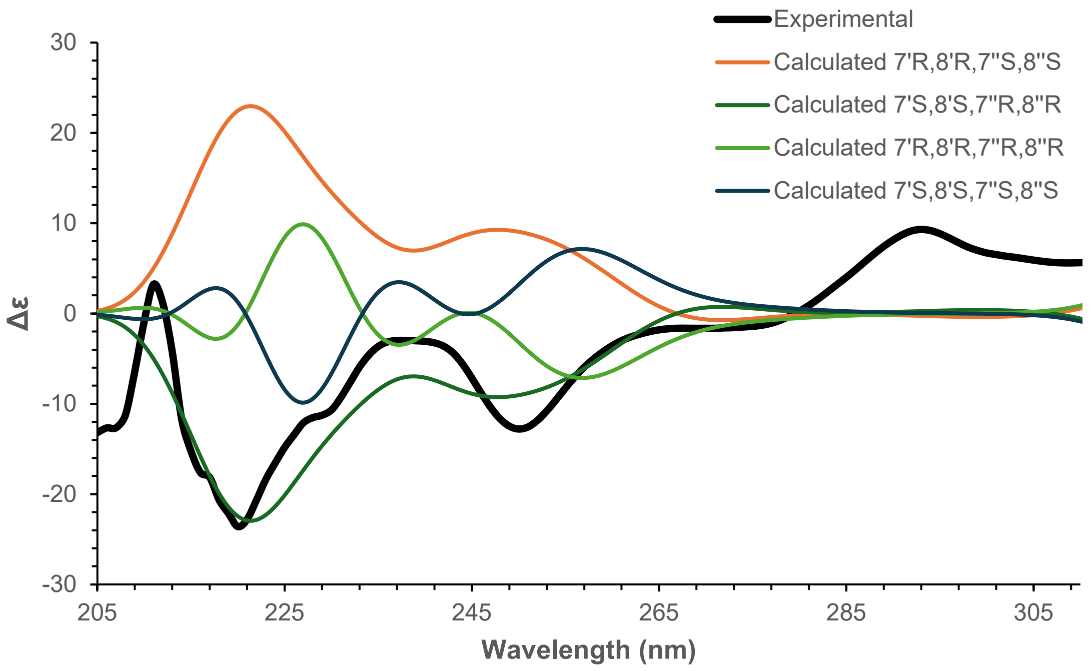

S11.1. ECD spectra (experimental and calculated) of (-)-Gnetuhainin M in MeOH.

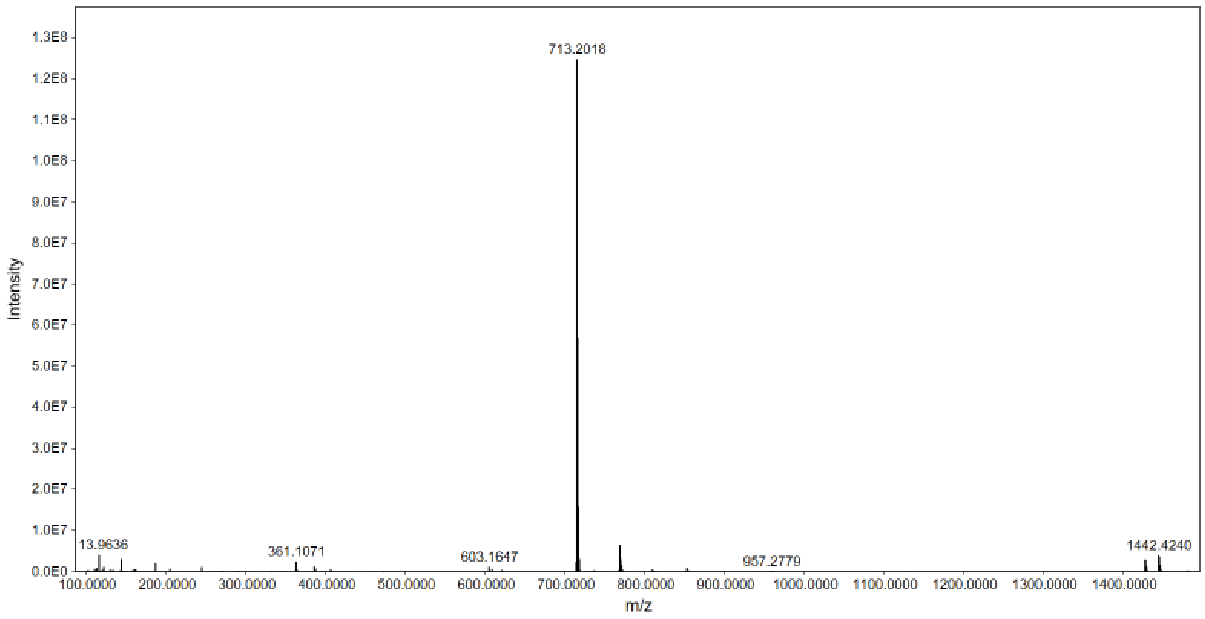

S11.2. HRESIMS+ spectrum of (-)-Gnetuhainin M in MeOH.

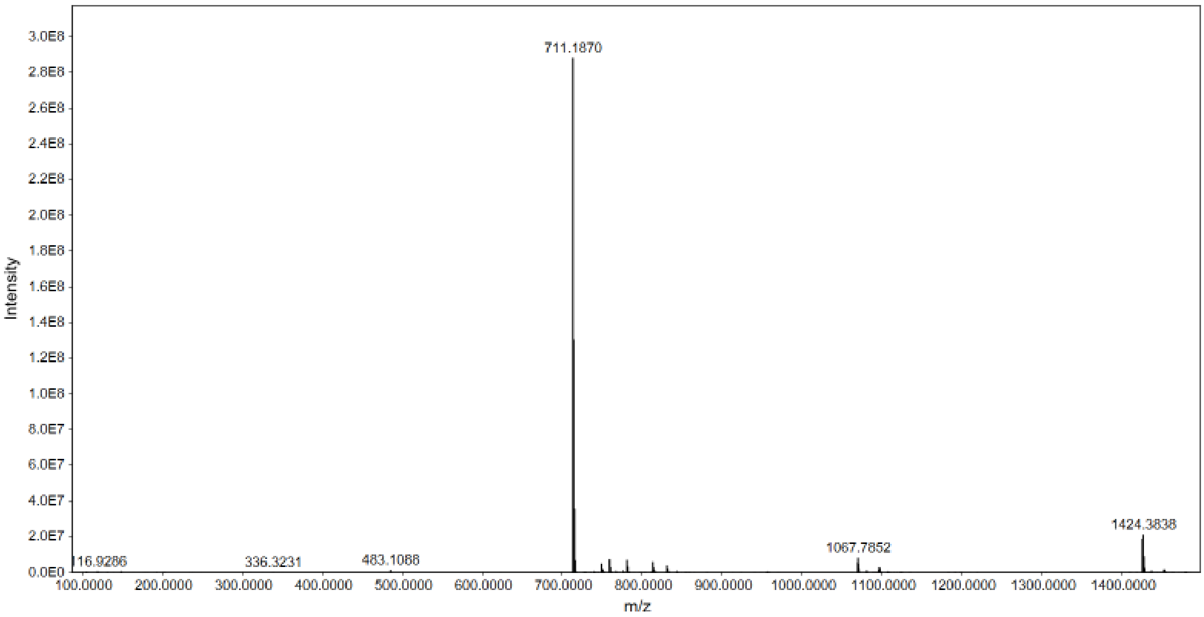

S11.3. HRESIMS- spectrum of (-)-Gnetuhainin M in MeOH.

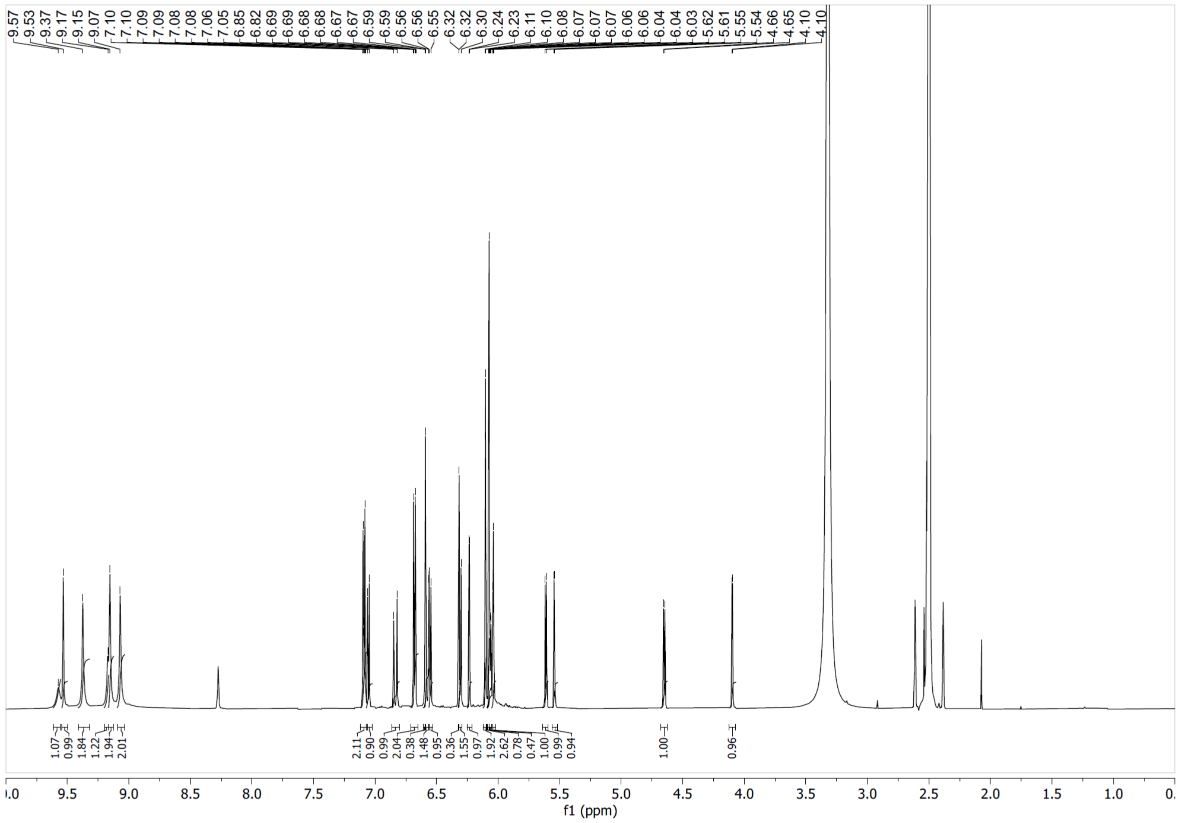

S11.4. ^{1}H NMR spectrum of (-)-Gnetuhainin M in DMSO-d_6 at 600 MHz.

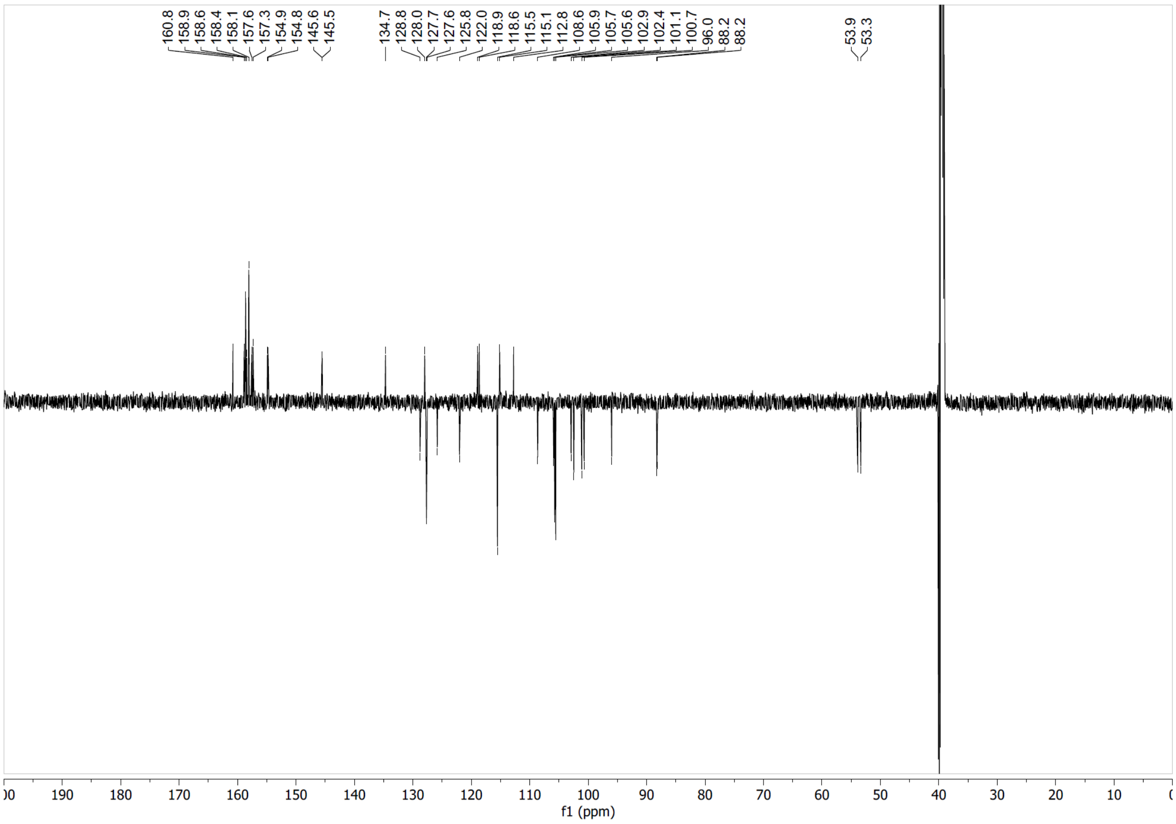

S11.5. ^{13}C NMR spectrum of (-)-Gnetuhainin M in DMSO-d_6 at 151 MHz.

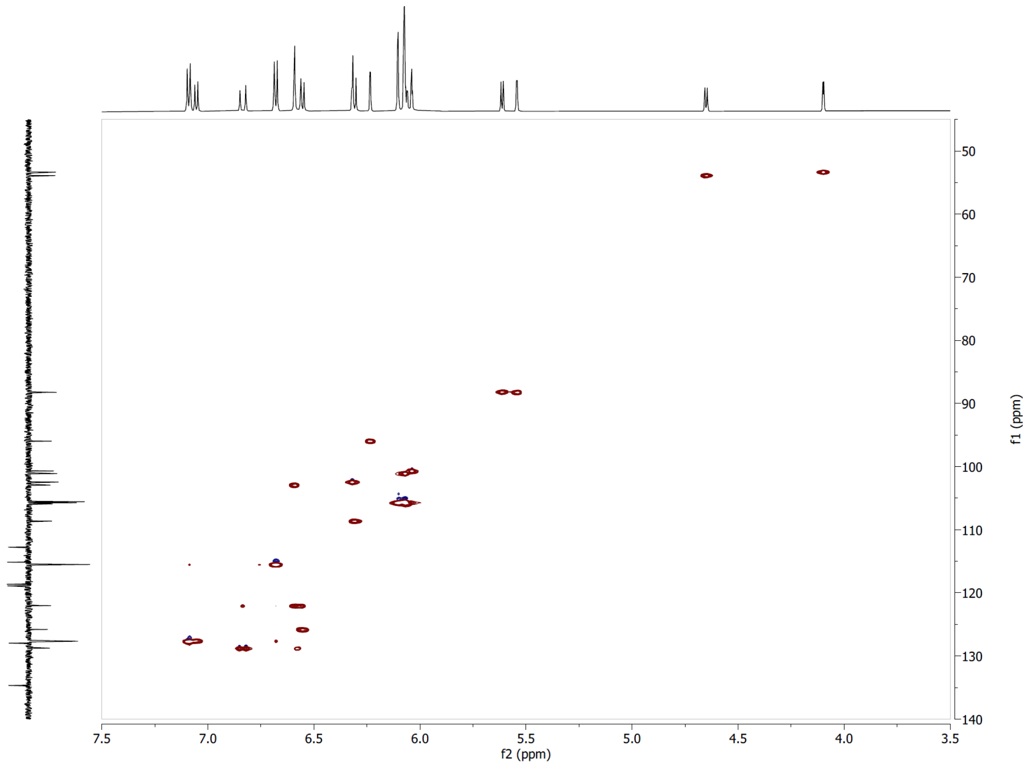

S11.6. HSQC NMR spectrum of (-)-Gnetuhainin M in DMSO-d_6.

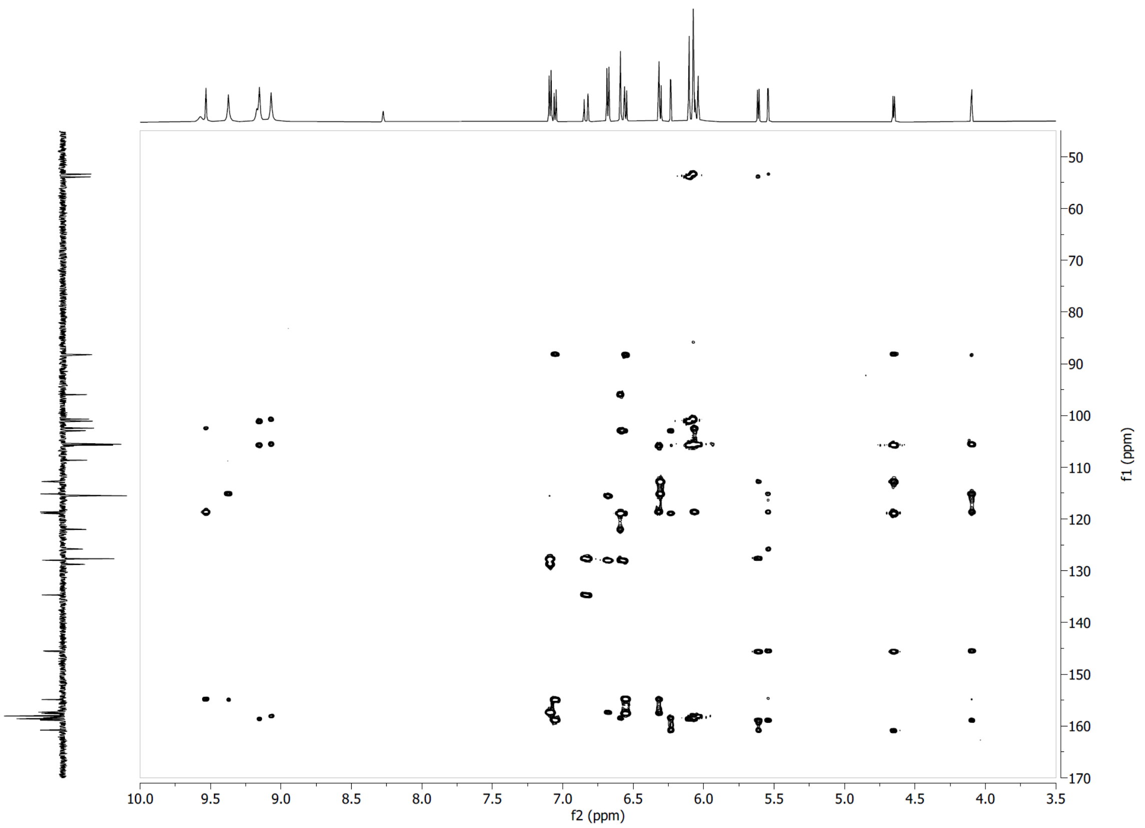

S11.7. HMBC NMR spectrum of (-)-Gnetuhainin M in DMSO-d_6.

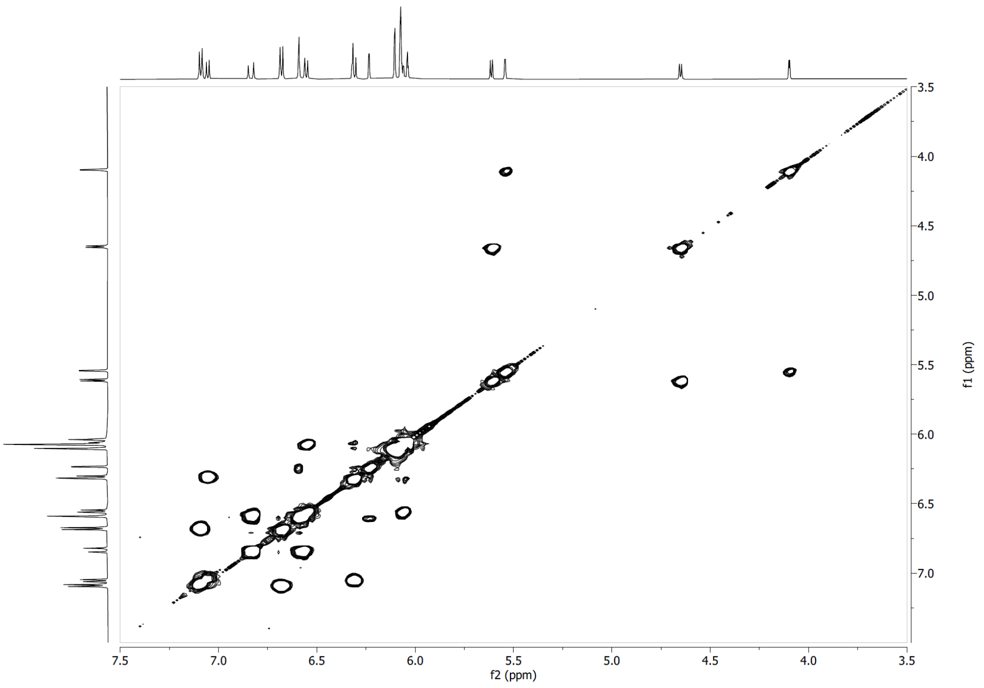

S11.8. COSY NMR spectrum of (-)-Gnetuhainin M in DMSO-d_6.

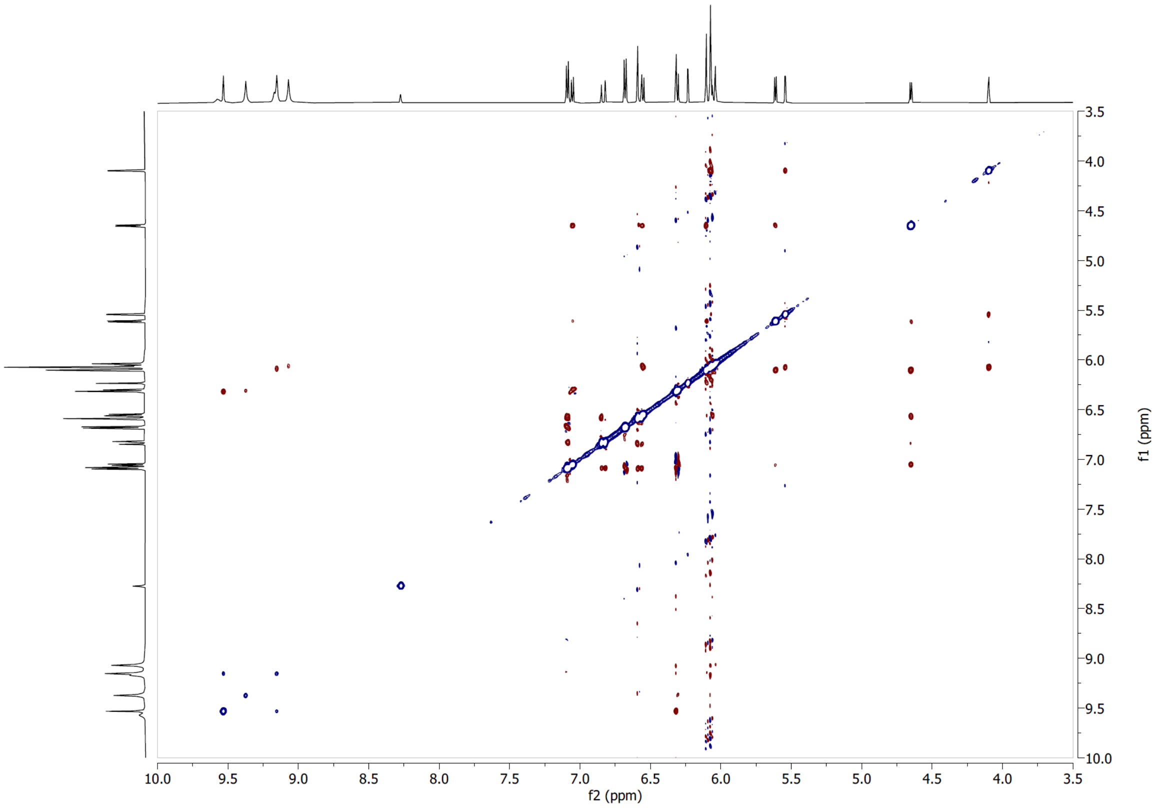

S11.9. ROESY NMR spectrum of (-)-Gnetuhainin M in DMSO-d_6.

REFERENCES

(1) Allard, P.-M.; Gaudry, A.; Quirós-Guerrero, L.-M.; Rutz, A.; Dounoue-Kubo, M.; Walker, T. W. N.; Defossez, E.; Long, C.; Grondin, A.; David, B.; Wolfender, J.-L. Open and Reusable Annotated Mass Spectrometry Dataset of a Chemodiverse Collection of 1,600 Plant Extracts. *GigaScience* **2023**, *12*, giac124. https://doi.org/10.1093/gigascience/giac124.

(2) Hanwell, M. D.; Curtis, D. E.; Lonie, D. C.; Vandermeersch, T.; Zurek, E.; Hutchison, G. R. Avogadro: An Advanced Semantic Chemical Editor, Visualization, and Analysis Platform. *Journal of Cheminformatics* **2012**, *4* (1), 17. https://doi.org/10.1186/1758-2946-4-17.

(3) Nugroho, A. E.; Morita, H. Circular Dichroism Calculation for Natural Products. *Journal of Natural Medicines* **2014**, *68* (1), 1–10. https://doi.org/10.1007/s11418-013-0768-x.

(4) Mándi, A.; Kurtán, T. Applications of OR/ECD/VCD to the Structure Elucidation of Natural Products. *Nat. Prod. Rep.* **2019**, *36* (6), 889–918. https://doi.org/10.1039/C9NP00002J.

(5) Li, X.-M.; Lin, M.; Wang, Y.-H. Stilbenoids from the Lianas of Gnetum Pendulum. *Journal of Asian Natural Products Research* **2003**, *5* (2), 113–119. https://doi.org/10.1080/1028602021000054964.

(6) Buffeteau, T.; Cavagnat, D.; Bisson, J.; Marchal, A.; Kapche, G. D.; Battistini, I.; Da Costa, G.; Badoc, A.; Monti, J.-P.; Mérillon, J.-M.; Waffo-Téguo, P. Unambiguous

Determination of the Absolute Configuration of Dimeric Stilbene Glucosides from the Rhizomes of Gnetum Africanum. *J. Nat. Prod.* **2014**, *77* (8), 1981–1985. https://doi.org/10.1021/np500427v.

(7) Iliya, I.; Ali, Z.; Tanaka, T.; Iinuma, M.; Furusawa, M.; Nakaya, K.; Murata, J.; Darnaedi, D. Stilbenoids from the Stem of Gnetum Latifolium (Gnetaceae). *Phytochemistry* **2002**, *61* (8), 959–961. https://doi.org/10.1016/S0031-9422(02)00289-3.

(8) Sri-in, P.; Sichaem, J.; Siripong, P.; Tip-pyang, S. Macrostachyols A–D, New Oligostilbenoids from the Roots of Gnetum Macrostachyum. *Fitoterapia* **2011**, *82* (3), 460–465. https://doi.org/10.1016/j.fitote.2010.12.008.

(9) Iliya, I.; Tanaka, T.; Iinuma, M.; Ali, Z.; Furasawa, M.; Nakaya, K.; Shirataki, Y.; Murata, J.; Darnaedi, D. Stilbene Derivatives from Two Species of Gnetaceae. *Chemical and Pharmaceutical Bulletin* **2002**, *50* (6), 796–801. https://doi.org/10.1248/cpb.50.796.

(10) Zhai, Y.-M.; Jiang, K.; Qu, S.-J.; Luo, H.-F.; Tan, J.-J.; Tan, C.-H. Structurally Diverse Stilbene Dimers from Gnetum Montanum Markgr.: Studies on the 1H Chemical Shift Differences between Dimeric Stilbene Epimers Correlating to the Relative Configurations. *RSC Adv.* **2016**, *6* (55), 50083–50090. https://doi.org/10.1039/C6RA08238F.

(11) Li, X. L., Mao; Wang, Ying-hong; Liu, Xin. Four New Stilbenoids from the Lianas of Gnetum Montanum f. Megalocarpum. *Planta Med* **2004**, *70* (02), 160–165. https://doi.org/10.1055/s-2004-815494.

(12) Huang, K.-S. L., Rong-Li; Wang, Ying-Hong; Lin, Mao. Three New Stilbene Trimers from the Lianas of Gnetum Hainanense. *Planta Med* **2001**, *67* (01), 61–64. https://doi.org/10.1055/s-2001-10875.
